# Exploration of Autophagy Families in Legumes and Dissection of the ATG18 Family with a Special Focus on *Phaseolus vulgaris*

**DOI:** 10.3390/plants10122619

**Published:** 2021-11-29

**Authors:** Elsa-Herminia Quezada-Rodríguez, Homero Gómez-Velasco, Manoj-Kumar Arthikala, Miguel Lara, Antonio Hernández-López, Kalpana Nanjareddy

**Affiliations:** 1 Ciencias Agrogenómicas, Escuela Nacional de Estudios Superiores Unidad León, Universidad Nacional Autónoma de México (UNAM), León C.P. 37684, Mexico; qrelsa@gmail.com (E.-H.Q.-R.); manoj@enes.unam.mx (M.-K.A.); ahernandez@enes.unam.mx (A.H.-L.); 2 Instituto de Química, Universidad Nacional Autónoma de México (UNAM), Cuidad Universitaria, Cuidad de Mexico C.P. 04510, Mexico; antropofagomer@hotmail.com; 3 Departamento de Biología Molecular de Plantas, Instituto de Biotecnología, Universidad Nacional Autónoma de México (UNAM), Cuernavaca C.P. 62271, Mexico; mflara@ibt.unam.mx

**Keywords:** homologs, phylogeny, *ATG18*, FRRG motif, principal component, 3D model, expression profile

## Abstract

Macroautophagy/autophagy is a fundamental catabolic pathway that maintains cellular homeostasis in eukaryotic cells by forming double-membrane-bound vesicles named autophagosomes. The autophagy family genes remain largely unexplored except in some model organisms. Legumes are a large family of economically important crops, and knowledge of their important cellular processes is essential. Here, to first address the knowledge gaps, we identified 17 *ATG* families in *Phaseolus vulgaris*, *Medicago truncatula* and *Glycine max* based on *Arabidopsis* sequences and elucidated their phylogenetic relationships. Second, we dissected *ATG18* in subfamilies from early plant lineages, chlorophytes to higher plants, legumes, which included a total of 27 photosynthetic organisms. Third, we focused on the ATG18 family in *P. vulgaris* to understand the protein structure and developed a 3D model for PvATG18b. Our results identified ATG homologs in the chosen legumes and differential expression data revealed the nitrate-responsive nature of ATG genes. A multidimensional scaling analysis of 280 protein sequences from 27 photosynthetic organisms classified ATG18 homologs into three subfamilies that were not based on the BCAS3 domain alone. The domain structure, protein motifs (FRRG) and the stable folding conformation structure of PvATG18b revealing the possible lipid-binding sites and transmembrane helices led us to propose PvATG18b as the functional homolog of AtATG18b. The findings of this study contribute to an in-depth understanding of the autophagy process in legumes and improve our knowledge of ATG18 subfamilies.

## 1. Introduction

Autophagy is a degradation process essential in the maintenance of homeostasis in eukaryotic cells and is related to a wide variety of physiological and pathophysiological roles, such as host defense, development, infection, and tumorigenesis [1,2]. Autophagy/macroautophagy is a process in which cytosolic components are sequestered within double-membrane vesicles called autophagosomes, which fuse with lysosomes or vacuoles for degradation/recycling [3]. This process is mediated by evolutionarily conserved genes known as autophagy genes (*ATG*s) [4], which were originally discovered in and isolated from *Saccharomyces cerevisiae* [5,6,7,8]. Three major intracellular autophagy pathways, namely, macroautophagy, microautophagy and chaperone-mediated autophagy (CMA), have been elucidated, and these differ in the mode of cargo delivery to the lysosome or vacuole [9,10]. Macroautophagy can be nonselective or selective: Nonselective autophagy is a cellular response to nutrient deprivation that involves the random uptake of cytoplasm into phagophores (precursors to autophagosomes) [11], and selective autophagy is responsible for the specific removal of certain components, such as protein aggregates and damaged or superfluous organelles [12,13]. Selective autophagic degradation has been observed with several organelles, such as mitochondria [14], peroxisomes [15], lysosomes [16], endoplasmic reticulum and nucleus [17]. In contrast, microautophagy is the least characterized type of autophagy; during this nonselective process, smaller molecules acting as substrates and the cargo for degradation are transferred into vacuole via invagination of the tonoplast membrane. CMA involves molecular chaperones in the cytosol that unfold proteins and translocate them through the lysosomal membrane [18].

Research on plant autophagy has improved enormously since the first genetic analysis of plant autophagy was performed [19,20,21,22,23,24]. During the process of autophagy, *ATG* genes play a key role and are classified into several functional groups: The ATG1 kinase complex, the ATG9 recycling complex, the phosphatidylinositol 3-kinase (PI3K) complex and the ATG8 and ATG12 conjugation systems [12].

Autophagy/macroautophagy can be activated under nutrient-depletion conditions via the inhibition of mammalian target of rapamycin (mTOR) or the activation of AMPK. Under TOR-inhibiting conditions, ATG13 is rapidly dephosphorylated, which results in its association with ATG1 and the additional proteins ATG11 and ATG101 and thus stimulation of the autophagy process [25,26]. Phagophore expansion is driven by the transmembrane protein ATG9 along with its cycling factors ATG2 and ATG18 [27,28]. Furthermore, assembly of the phagophore is completed with phosphatidylinositol-3-phosphate (PI3P) by a complex containing class III phosphatidylinositol-3-kinase (PI3K), vacuolar protein sorting 34 (VPS34), ATG/VPS30/beclin-1, VPS38, ATG14 and VPS15 [28]. Phagophore expansion and maturation are completed by ATG8, which is cleaved by cysteine proteinase ATG4 to expose the C-terminal glycine residue [29]. Subsequently, the exposed glycine of ATG8 is conjugated to the membrane lipid phosphatidylethanolamine (PE) via a ubiquitin-like conjugation reaction catalyzed by ATG7 (E1-like enzyme), ATG3 (E2-like enzyme) and the ATG12-ATG5 complex (E3-like enzyme) [30,31,32]. The ATG8-PE adduct can be deconjugated from the membrane by ATG4 proteinase; hence, ATG8 is recycled to participate in new conjugation events [29,33].

ATG18 is an autophagy-related molecule that regulates the vacuolar shape and is conserved from yeast to higher organisms, including the human proteins WIPI1–WIPI4 [34]. While yeast has only one ATG18 gene and two other genes with WD40 repeats, the plant ATG18 family diversifies from two genes in algae to multiple genes in higher plants. The Atg18 protein is characterized by the presence of several WD-40 domains and has been predicted to form a *β*-propeller structure that binds to phosphatidylinositol 3-phosphate (PtdIns(3)P) and phosphatidylinositol 3,5-bisphosphate (PtdIns(3,5)P_2_) [35,36,37]. The binding of PtdIns(3)P and Atg18 is needed for the efficient recruitment of Atg8 and Atg16 during phagophore formation at the phagophore assembly site (PAS) [38]. A previous study showed that phagophore formation could also be affected in the absence of the Atg2-Atg18 complex, although other Atg proteins accumulate at the PAS [39]. The Atg2-Atg18 complex has also been shown to localize to a few specific spots on the opening edge of the isolation membrane that lie close to sites for COPII vesicle formation in the endoplasmic reticulum (ER) or ER exit sites [40,41].

Among plants, *Arabidopsis* contains eight *ATG18* homologs, which are classified as *AtATG18a–h*, and multiple splice variants [42,43], and rice has six *ATG18* homologs. *AtATG18a* is involved in oxidative, drought and salt stress [42,43,44,45]. Recent studies have also suggested the regulation of autophagy by the reversible persulfidation of *AtATG18a* under ER stress [46]. Similarly, *ATG18* is reportedly involved in autophagy regulation under abiotic stress conditions in sweet orange (*Citrus sinensis*) [47], tomato (*Solanum lycopersicum*) [48] and apple (*Malus domestica*) [49,50]. To date, *AtATG18a* is the only member of the *ATG18* family that has been established as an essential component of autophagy in *A. thaliana*.

Recent studies on *ATG* genes conducted by Norizuki and colleagues (2019) [51] have shown the diversification of *ATG*s from early plant lineages to higher plants. However, legumes are a large and economically important family of flowering plants, and few studies have investigated autophagy-related aspects. The aim of the present study was to expand the previous studies to higher clades, specifically to fabaceous plants, and thus understand the current diversity and complexity of *ATG*s. Furthermore, we focused on the *ATG18* family to understand its evolutionary relationships, diversification, expression patterns and *cis-*regulatory elements in many plants ranging from early plant lineages to fabaceous members. We also performed a comprehensive study of various functional and structural aspects of *ATG18b* in *P. vulgaris*.

## 2. Results

### 2.1. Identification of ATG families in P. vulgaris, M. truncatula and G. max

In *A. thaliana*, a total of 39 ATG sequences divided into 17 families have been reported. In the present study, we identified a total of 32 genes in *P. vulgaris* (2n), 39 genes in *M. truncatula* (2n) and 61 genes in *G. max* (4x) (Table 1). A BLAST analysis of *Arabidopsis* sequences returned 19 (59.37%) homologs in *P. vulgaris*, 28 (77.77%) homologs in *M. truncatula* and 30 (48.38%) homologs in *G. max* with a query coverage of 93–94% and 66–77% identity (Supplementary Information SI1). For this reason, other ortholog analysis databases were used to identify any missing ATG members. The KEGG orthology table for the autophagy pathway was the second main tool because it contains a wide variety of species, and we used this table to obtain more than 70% of genes in *P. vulgaris* and *M. truncatula* and 58% in *G. max*. An analysis of legumes using Ensembl Plants provided more than 70% of ATGs in the legumes under study. Other studies were performed through a HMMER analysis using Ensembl databases and the InParanoid tools in Phytozome. The obtained sequences were verified using Pfam to acquire the positions of the families, domains and repeats, and the protein motifs were determined with MEME. Additional studies were performed using EggNOG, which provided a list of orthologs, particularly in *P. vulgaris* (Supplementary Figure S1). We also identified 21, 17 and 15 orthologs and 10, 17 and 21 paralogs in *P. vulgaris*, *M. truncatula* and *G. max,* respectively. The genes identified in *P. vulgaris, M. truncatula* and *G. max* are listed in Table S1.

### 2.2. Phylogenetic Relationships, Chromosome Localization, Synteny and Ka/Ks Ratio of ATG Families in Legumes

**Figure 1:**
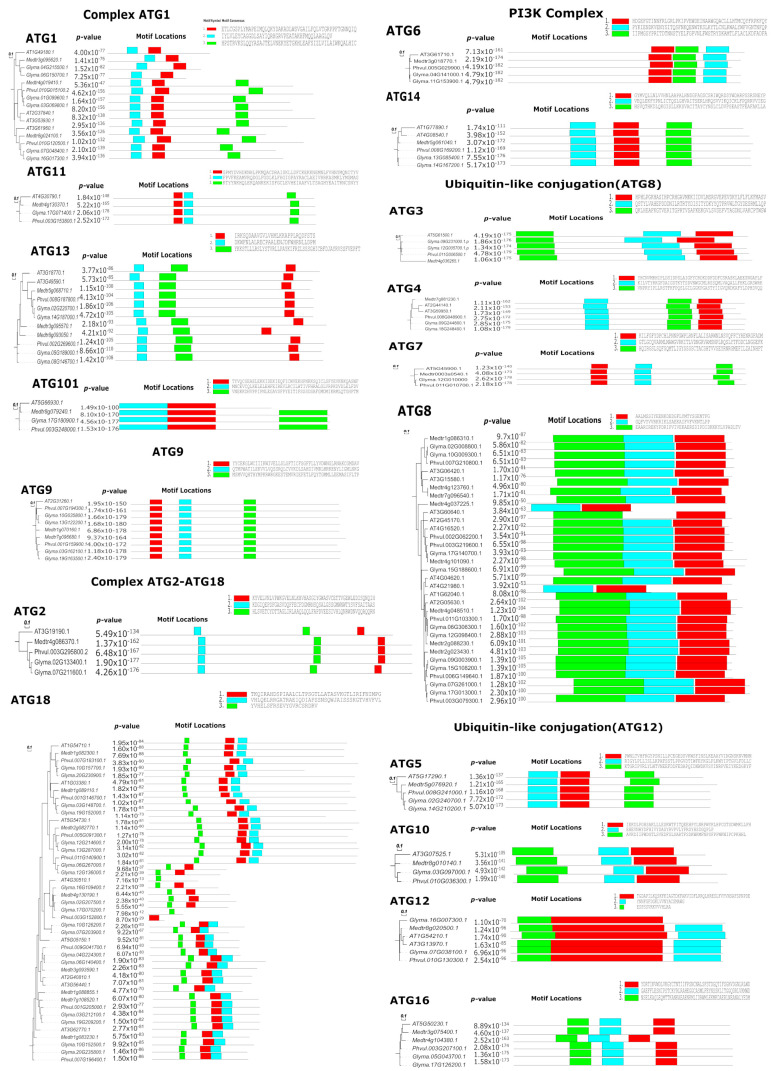
Phylogenetic analysis and protein motifs of 17 ATG families in *A. thaliana*, *P. vulgaris*, *M. truncatula* and *G. max*. The phylogenetic tree was constructed with the neighbor-joining method with 1000 repeated bootstrap tests, p-distance and pairwise deletion in MEGA X software and visualized using EvolView. MEME was used to identify motifs of the ATG homologs in *A. thaliana*, *P. vulgaris*, *M. truncatula* and *G. max*.

To understand the evolutionary relationships among ATGs, we generated 17 phylogenetic trees, one for each ATG family in *A. thaliana, P. vulgaris*, *M. truncatula* and *G. max* as per the classification in *A. thaliana*. The primary protein sequences of *A. thaliana*, *P. vulgaris*, *M. truncatula* and *G. max* were aligned using Clustal Omega with the default parameters, and phylogenetic trees were obtained with the neighbor-joining method. Each of the ATG sequences was also subjected to a motif analysis, which revealed that the sequences and motifs in all the studied legumes showed high identity to their homologs in *Arabidopsis*. The phylogenetic tree also revealed that the majority of the ATG family distributions was predominantly composed of Medicago sequences that were more closely related to those in Arabidopsis. Among all the phylogenetic trees of ATGs developed, 11 contained only one clade (ATG2, ATG3, ATG4, ATG5, ATG6, ATG7, ATG10, ATG11, ATG12, ATG14 and ATG101), even if there was more than one isoform, and most of the motif P-values were greater than 1e-100. ATG8 and ATG18 were the families with the highest number of members: ATG18, eight each in *Arabidopsis*, *Medicago* and *Phaseolus* and 19 in *G. max*; ATG8, nine in *Arabidopsis*, eight in *Medicago*, six in *P. vulgaris* and 10 in *G. max*. The phylogenetic analysis of ATG8 and ATG18 was divided into three clades with motif P-values between 1 × 10^−13^ and 1 × 10^−90^ (Figure 1). The close association of the homologs in all the species studied depicts the conservation of sequences and hence implies biological function conservation.

**Figure 2:**
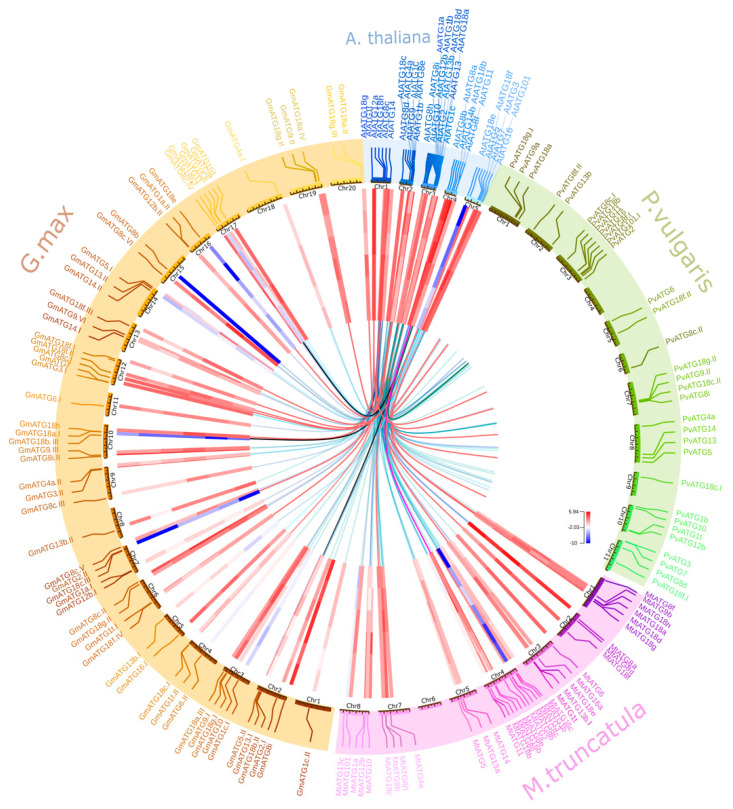
The chromosomal localization, synteny relationship and gene expression of autophagy genes were integrated into the Circos plot designed using OmicCircos. The outermost circle shows the *A. thaliana* (blue), *P. vulgaris* (green)*, M. truncatula* (pink) and *G. max* (brown) chromosomes. The inner circle is a heatmap that shows the log_2_ RPKM values of gene expression in leaves and roots under ammonia, nitrate and urea treatments. The innermost line is the synteny of autophagy genes, but the yellow, purple and red lines represent ATG18b subfamilies I, II and III, respectively.

The chromosome localization of ATGs in the *A. thaliana* and legume genomes was mapped using Circos (Figure 2). The distribution of ATG homologs among the chromosomes was uneven in all the species compared. Among all 17 families, the maximal number of homologs was located on chromosome 3 in *A. thaliana* (8) and *P. vulgaris* (6)*,* chromosome 4 in *M. truncatula* (6) and chromosomes 4 and 17 in *G. max* (6).

**Figure 3:**
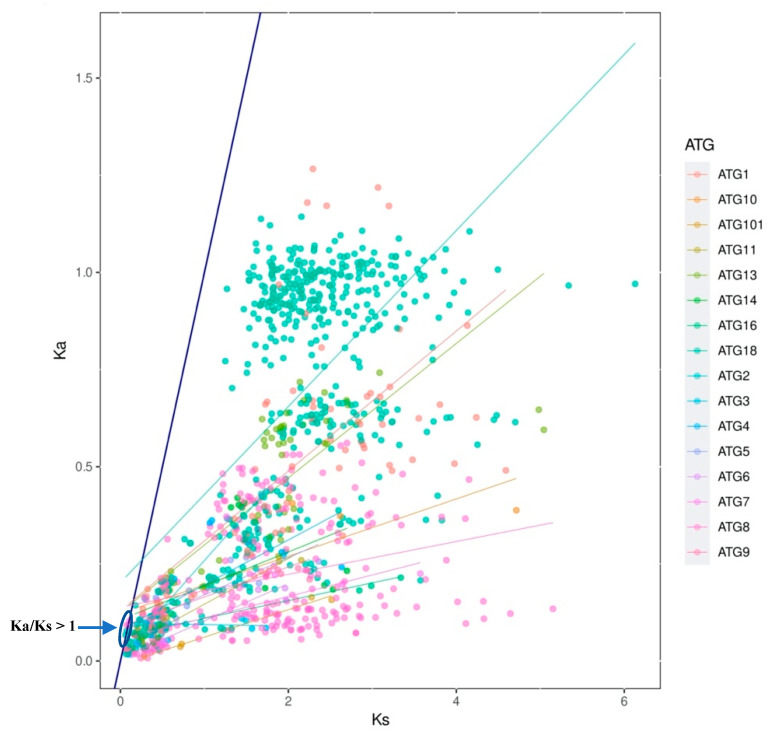
Ka/Ks ratios of 17 families of ATGs in *A. thaliana, P. vulagris, G. max* and *M. truncatula*. The distribution of Ka and Ks values are obtained using TBtools. The dark blue line divides the Ka/Ks ratios lower and higher than 1 (dots in the highlighted area Ka/Ks > 1).

The Ka/Ks ratio among most of the ATG sequences was lower than 1 (average 0.17), which indicates purifying selection; in contrast, the sequences of ATG8 (1.24) and two sequences (GmATG18e and GmATG18b. I) of ATG18 (1.09 and 1.04) in *G. max* had values higher than 1, which indicated accelerated evolution and positive selection (Figure 3). The Ka/Ks ratios suggest the conservation of ATG homologs in terms of both sequence and biological function.

### 2.3. Promoter Analysis and Expression Profiling of ATG Families

**Figure 4:**
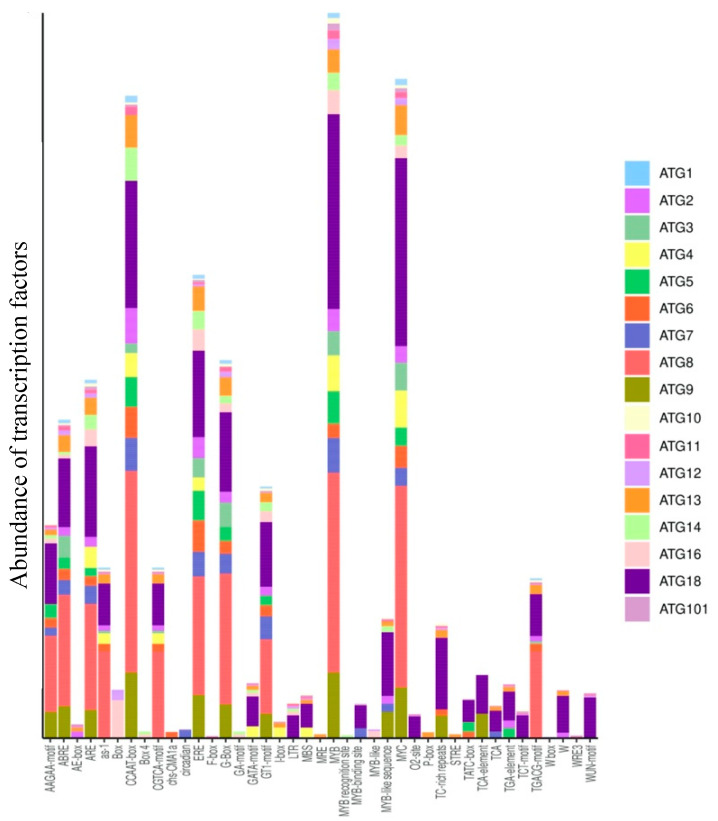
Transcription factor-binding sites in the promoter regions of ATGs (2000 bp) identified using PlantCARE.

Promoter analysis is an important method for understanding the regulatory mechanisms governing ATGs in response to growth and developmental issues and to environmental cues. The analysis of *cis*-acting elements in the promoters of all 17 ATG families resulted in 44 different transcription factors. The most abundant transcription factors identified were B-Proto-Oncogene-MYB involved in the ABA response and C-Proto-Oncogene-MYC related to jasmonate signaling, and the transcription factors with the motifs ethylene response elements (ERE), TATA box, CAATT-box and G-box were found for all ATGs in *A. thaliana*, *P. vulgaris*, *M. truncatula* and *G. max* (Supplementary Figure S5). Our results also showed that the ATG8 and ATG18 families contained the highest numbers of MYB, MYC, ERE and Box 4 (ATTAAT) transcription factor-binding sites. Most of the promoters contained MeJA-, SA-, GA- and ABA-responsive elements. Furthermore, light-responsive transcription factors such as BOX-4, G-box, GT1 motif, MRE and ACE were also detected abundantly in most of the families (Figure 4).

**Figure 5:**
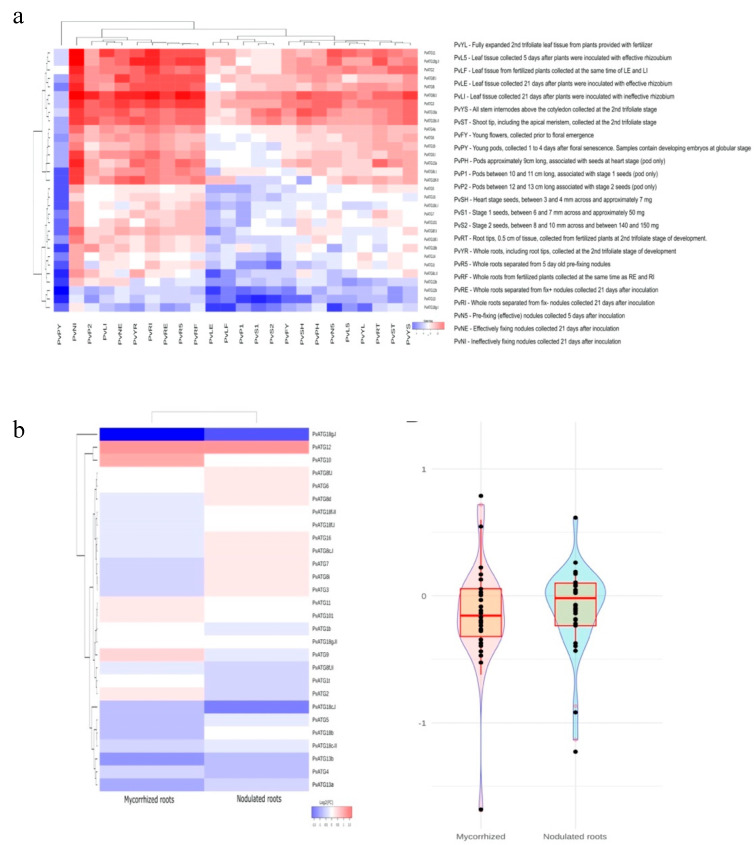
Expression profiles of ATGs in *P. vulgaris* tissues. (**a**) The transcription abundances of *P. vulgaris ATGs* in different tissues and organs during different stages of development and during rhizobial infections obtained from the PvGEA database. (**b**) Expression data from nodulated roots (*R. tropici*) and mycorrhized roots (*R. irregularis*) obtained from RNA-seq analysis. A violin plot shows total number of up/dowregulated ATGs under nodulated/mycorrhized conditions. The highlighted box represents higher number of downregulated genes in mycorrhized condition.

Interestingly, we elucidated the influence of nitrogen sources on ATG expression in the legume members *P. vulgaris, M. truncatula* and *G. max* due to their ability to establish symbiotic associations with nitrogen-fixing Rhizobia. Gene expression data from the Phytozome database were retrieved for leaf and root tissues under urea as the organic source and nitrate and ammonia as inorganic sources, as depicted in Figure 2. The highest expression of ATGs was recorded in roots treated with ammonia and leaves treated with urea. *ATG8i* and *ATG3* showed the highest abundance in all the treatments, and the lowest expression levels were recorded for *ATG18b, e, c* and *h, ATG2* and *ATG2.II* in *G. max* and *ATG3* and *ATG8c* in *M. truncatula*. The *ATG18* family homologs *ATG18a.II*, *ATG18g* and *ATG18h* showed induced expression in all tissues under all treatments (Figure 2 and Figure 5a).

Furthermore, the differential expression analysis of *ATG*s in *P. vulgaris* tissues showed very low expression in young pods collected 1 to 4 days post floral senescence, whereas the fix-(inefficient) nodules collected at 21 days showed the most abundant expression of all *ATG*s. Interestingly, inefficient fixation increased the expression levels compared with those found with efficient fixation. Among all *PvATG*s, the *ATG1*, *ATG10*, *ATG13b*, *ATG18c* and *ATG18g.I* genes showed the lowest expression in all the analyzed tissues, and a total of 16 ATGs were found to be expressed in most of the tissues (Figure 5a; Supplementary Information SI2). Following the interesting observation of *ATG* expression in nodules, we analyzed the expression of *ATG*s using our previously generated RNA-seq data of *Rhizobium*/mycorrhiza-inoculated *P. vulgaris* roots. The results were interesting: Six *ATG*s were upregulated and 16 *ATG*s were downregulated in mycorrhized roots, and nine *ATG*s were upregulated and 12 *ATG*s were downregulated in nodulated roots (Figure 5b; Supplementary Information SI2). The expression of *ATG10* was found to be specifically induced in mycorrhized roots, *ATG12* was highly induced and *ATG18g.l* was highly suppressed under both symbiotic conditions. The RNA-seq data was validated using RT-qPCR for *PvATG2*, *PvATG8i*, *PvATG9* and *PvATG10*.

### 2.4. Identification of ATG18 Families in Plants

Through an extensive study aiming to identify and analyze the *ATG18* family, we selected 27 plant species starting from the early plant lineage Chlorophyta, Charophyta, liverworts, mosses and higher plants such as monocots and dicots. As with other *ATG*s, the *ATG18* family is also well conserved in all the studied plant species; herein, a total of 280 genes and amino acid sequences were identified and retrieved from various databases. Initially, we identified the *ATG18* homologs through a BLAST search of NCBI, and we then used the Pfam database to ensure the presence of WD40 repeats in the characteristic *ATG18* members. The identified members were named using the aliases registered in the legume information system, NCBI, Phytozome, InParanoid, EGGNOG and Ensembl (Supplementary Information SI3). The genes with the same names were distinguished by adding a Roman numeral: The number I indicated the closest sequence to that in NCBI. For the primitive plants *Physcomitrella patens, Chara braunii, Chlamydomonas reinhardtii, Dunaliella salina, Volvox carteri, Klebsormidium* *nitens, Micromonas pusilla, Ostreococcus* *lucimarinus, Ostreococcus tauri* and *Coccomyxa* *subellipsoidea,* we retained the same names that were reported by Norizuki and colleagues [51].

Starting from the most primitive photosynthetic organisms of Chlorophyta, all the members studied had two *ATG18* homologs except *C. subellipsoidea*, which had three *ATG18* genes. Charophyta (*C. braunii*), liverworts (Marchantia polymorpha) and mosses (*P. patens*) had two, four and eight genes, respectively. Among monocots, we found that *Oryza sativa* had the lower number of genes (8), and *Z. mays* had the highest number of genes (31). *Arabidopsis* had a total of eight *ATG18* members, and the 12 legumes considered here together had a total of 180 genes belonging to the *ATG18* family. *P. sativum* had a minimum of six, and a maximum of 27 genes were found in *L. angustifolius*. The details of the *ATG18* homologs in every species are listed in Table 2 and Table 3.

### 2.5. Principal Component Analysis of the ATG18 Family

**Figure 6:**
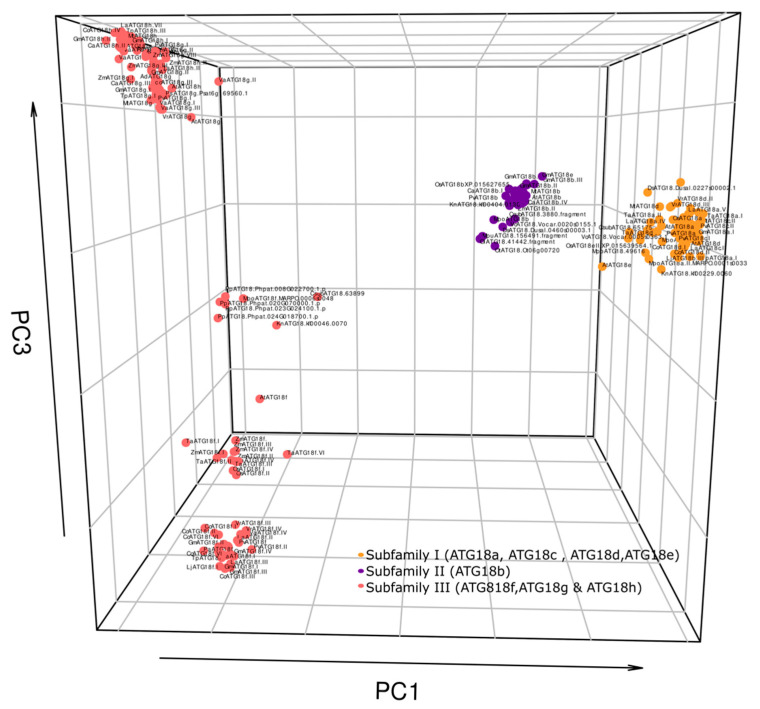
Three-dimensional representation of 280 ATG18 proteins from different plant species analyzed by multidimensional scaling using Bios2mds. The ATG18 subfamilies are color-coded as follows: Subfamily I (yellow), subfamily II (purple) and subfamily III (red). PC, principal component. The axes are the principal components (PC): *x*-axis (PC1), *y*-axis (PC2) and *z*-axis (PC3).

Multidimensional scaling analysis using Bios2mds demonstrates the similarity between 280 *ATG18* protein sequences from 27 different species. The plot clearly shows that orthologs (genes with closely related sequences and having the same function in different species) are more similar than paralogs (genes that have similar sequences but have different functions in the same species). The plots show that all *ATG18* sequences were grouped into three clusters (Figure 6 and Supplementary Figure S3A). The principal components (PCs) allowed us to construct graphs with PC1, PC2 and PC3, and we then applied the K-means method. Cluster I formed a subfamily with *ATG18a, c, d* and *e* members from all the higher plant species studied. Cluster II contained only *ATG18b* homologs, and cluster III contained *ATG18f, g* and *h* members. Cluster III consisted of 3 groups: Lower plants formed a distant group, the second group contained the monocot-derived proteins, and the third group harbored all dicots except *Arabidopsis*, which was more similar to monocots than dicots. Lower plant species were found to be distributed mostly in clusters I and II with the exception of *K. nitens, C. subellipsoidea, M. polymorpha* and *P. patens,* which were also grouped in cluster III but exhibited more similarities among themselves than with higher plants. These clusters were named subfamilies I, II and III for convenience.

### 2.6. Phylogenetic Relationships of the ATG18 Family in Plants

**Figure 7:**
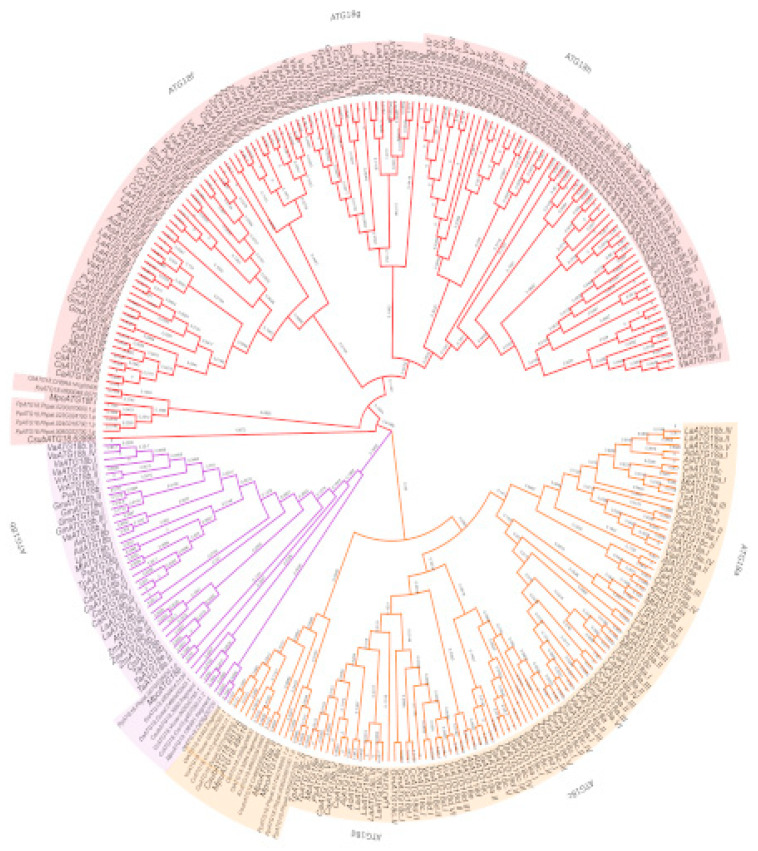
Phylogenetic tree of ATG18 proteins in plants. The protein sequences were aligned using Clustal Omega, and the phylogenetic tree was constructed using the ML method in MEGA X software with 1000 bootstrap replications. A total of 280 sequences of ATG18 are differentiated into subfamilies: Subfamily I (yellow), subfamily II (purple) and subfamily III (red). The plant species are differentiated by letters: *A. thaliana* (*At*), *M. polymorpha* (Mpo), *O. sativa* (Os), *Triticum* *aestivum* (Ta)*, Zea mays* (Zm), *Arachis* *duranensis* (Ad), *Arachis ipaensis* (Ai), *Cajanus cajan* (Cc), *Lotus japonicus* (Lj), *Cicer arietinum* (Ca), *Lupinus angustifolius* (La), *Pisum sativum* (Ps), *Vigna angularis* (Va), *Vigna radiata* (Vr) and *Trifolium pratense* (Tp), *P. patens, C. braunii* (Cb)*, C. reinhardtii* (Cr)*, D. salina* (Ds), *V. carteri* (Vc), *K. nitens* (Kn)*, M. pusilla* (Mpu)*, O. lucimarinus* (Ol)*, O. tauri* (Ot) and *C. subellipsoidea* (Cs). The branch lengths are labeled.

To understand the evolutionary relationship among primitive and advanced dicot plant species, a multiple sequence alignment of 280 ATG18 amino acid sequences was performed. The aligned sequences were used to generate phylogenetic trees based on the maximum likelihood and Bayes methods using MEGA and Phangorn software (Figure 7 and Supplementary Figure S3B). The largest clade was subfamily III followed by subfamily I, which was mainly composed of *ATG18 a, c, d* and *e*. Subfamily II harbored *ATG18b*. Subfamilies II and III consisted of the Bryopsida, Charophyceae, Klebsormidiophyceae, Mamiellophyceae and Trebouxiophyceae plants, which is important for understanding the divergence of *ATG18* homologs.

### 2.7. Analysis of the Primary Structure and the Secondary Structure Predictions of the ATG18 Family in Plants

**Figure 8:**
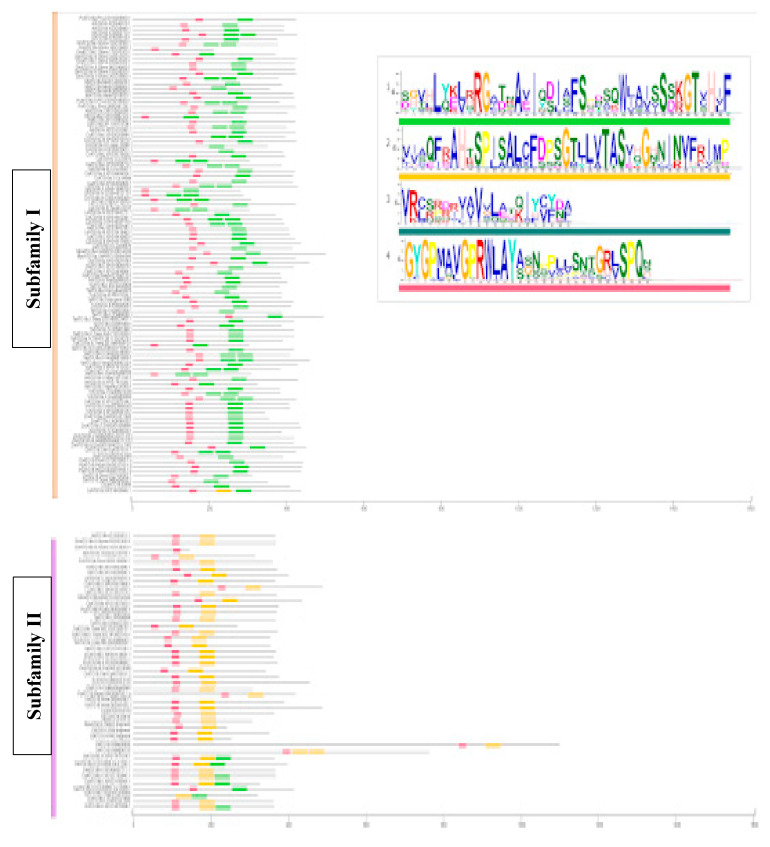

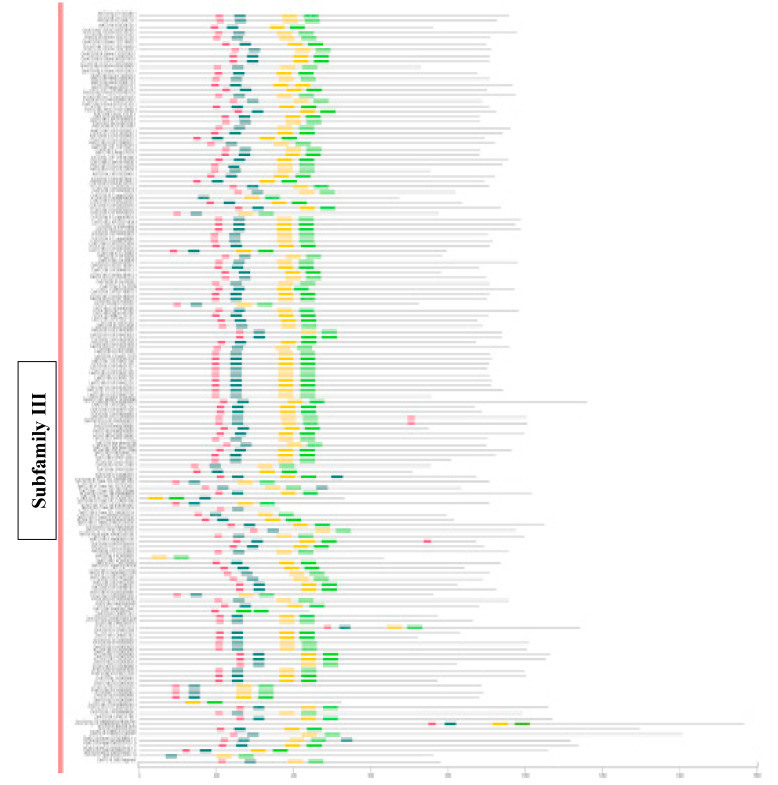
Protein motifs of the ATG18b family from different plant species. The conserved motifs were identified with MEME. The amino acid sequence of the ATG18 family is represented by lines, and the motifs identified using TBtools are shown with boxes: Motif 1 (green), motif 2 (yellow), motif 3 (dark green) and motif 4 (pink).

For the detection of motifs in 280 aa sequences, we identified four main motifs using MEME software. Motif 1 (SGVHLYKLRRGATNAVIQDIAFSHDSQWJAISSSKGTVHIF) contained 41 aa, and the motif sequence matched that of the WD40 family (PF00400) and β propeller clan 186 (CL0186) in the Pfam database. The InterProScan results also showed that motif 1 belongs to the superfamily WD40 (IPR036322), WD40 repeat-like (SSF50978) and breast carcinoma amplified sequence 3 (PTHR13268). Motif 2 (VIAQFRAHTSPISALCFDPSGTLLVTASVHGHNINVFRIMP) contained 41 aa and was similar to motif 1 but contained an additional domain (WD40/YVTN repeat-like domain, IPR015943). Moreover, motifs 3 (VRCSRDRVAVVLATQIYCYBA) and 4 (GYGPMAVGPRWLAYASNPPLLSNTGRLSPQN) did not belong to any protein family (Figure 8).

**Figure 9:**
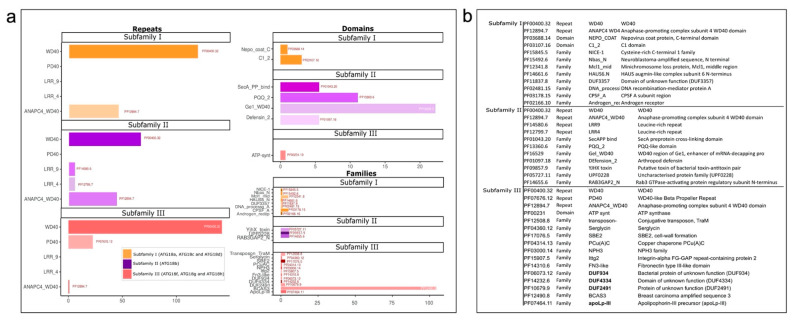
Repeats, domains and families of ATG18b sub-families. (**a**) The ATG18 protein functions were determined using Pfam, and the proteins were divided into subfamilies: Subfamily I (yellow), subfamily II (purple) and subfamily III (red). (**b**) Pfam identifiers and their annotations.

The motif sequences were further analyzed with PfamScan to identify the repeats, domains and families. Subfamily I was characterized by motifs 1 and 4, which consisted of WD40 and ANAPC4_WD40 repeats. These motifs also had two domains and eight families, although these Pfam family results are not representative of the subfamily. Subfamily II had motifs 1, 2 and 4, and we detected WD40 and ANAPC4_WD40 repeats in all the members. Only the green alga *O. tauri* contained leucine-rich repeats (LRR9 and LRR4). A total of four domains were identified: Gel_WD40, which was the largest, a defensin domain and PQQ and SecA preprotein crosslinking domains. Subfamily II also consisted of three families in six plants (Figure 9; Supplementary Information SI4).

Subfamily III had all four motifs, and we found PD40 repeats along with WD40 and ANAPC4_WD40 repeats. Among the 27 plant species analyzed, nine of them had 12 domains and ATP synthase was specific *Z. mays*. Breast carcinoma amplified sequence 3 (BCAS3) is a characteristic domain found in most members (Figure 9; Supplementary Information SI4).

The secondary structure of ATG18 was determined by protein alignment using JPred software. Here, we found that the sequence of ATG18h in *A. thaliana* was the largest sequence in the alignment with 927 aa. The protein contains seven blades with four beta blades commonly found in the WD40 family (Supplementary Figure S4). This sequence composition was 1% alpha-helix (H), 29% beta-sheet and 68% coil. ATG18 sequences have four antiparallel β-strands, which are named blades [52]. The beta-sheets in ATG18 proteins contain flexible loops that facilitate molecule binding.

**Figure 10:**
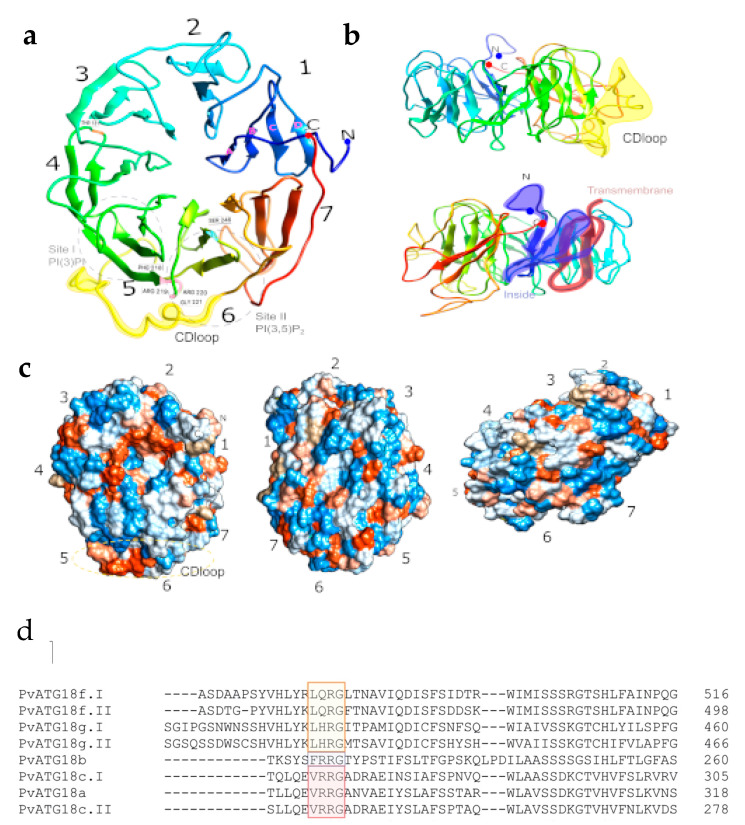
Three-dimensional structural model of PvATG18b determined by molecular dynamics simulation and alignment of ATG18 protein sequences of *P. vulgaris*. (**a**) The PvATG18b protein structure preserves seven blades of four β-strands. (**a**–**d**) In the colored rainbow, the N-terminus is shown in blue, the C-terminal is shown in red, the FRRG repeat (F218-G221) is colored pink, the conserved T131 residue is shown in orange and S246 Ser is presented in blue. The region consisting of site I PI(3)P and site II PI(3,5)P_2_ are shown in the gray circle. (**b**) PvATG18b protein structure rotated 180° and showing the CD loop (S269-T288) in yellow. (**c**) PvATG18b protein structure surfaces (positive and negative charges are shown in blue and red, respectively) showing a nonspecific electrostatic charge. (**d**) The FRRG repeat position is highlighted with the following colors: Subfamily I (yellow), subfamily II (purple) and subfamily III (red).

AtATG18h has an LHRG sequence in the same place where the alignments have the FRRG sequence, and we found the BCAS3 domain with Phe17 (Figure 10). The sequence alignment performed to identify the FRRG motif revealed that FRRGs appeared in subfamily II, which consists of ATG18b. In addition, subfamily I contained the LRRG or VRRG sequences, whereas subfamily III contained LQRG, LHRG or LYRG sequences. The sequences that appear in ATG18 contain the same pattern of two polar and neutral amino acids in the center of the sequence between two neutral and nonpolar amino acids. ATG18b in subfamily II has the conserved sequence for PtdInsP binding, and other subfamilies likely also show PtdInsP binding (Figure 10, Supplementary Figure S4).

### 2.8. Microsynteny of ATG18 in P. vulgaris

To explore the origins and evolutionary processes of the *P. vulgaris ATG18* family genes, a comparative synteny map between the eight *PvATG18* homologs and 15 other genomes was constructed. The species compared in this study were based on their availability in the GCV database. The classification of the *ATG18* family was based on the subfamilies obtained by multidimensional scaling (Figure 6).

#### 2.8.1. Subfamily I

*ATG18a* was highly conserved in all species with the exception of *A. ipaensis. SPATA 20* (legfed_v1_0.L_1H5ZXB) is tandemly duplicated in *P. vulgaris.* In contrast, the *lyase dihydroneopterin aldolase* (legfed_v1_0.L_2MWVJ4) was only found in *P. vulgaris* in the syntenic block. Other genes conserved in the syntenic block were related to cell cycle regulation, transcriptional regulation, transcription factors, zinc finger proteins and other structural motifs involved in peroxisomal and mitochondrial import (Supplementary Figure S5A).

*ATG18c* was not located in the syntenic block in *L. albus, M. truncatula, P. sativum or V. angularis*. Genes related to *ABC* transport, vacuolar iron transport, proteins with WD40 repeats involved in protein–protein interactions, cytochrome *P450*, oxidoreductases and zinc-binding dehydrogenase were highly conserved in the syntenic block. *T. pratense* and *P. lunatus* show duplication of oxidoreductases and zinc-binding dehydrogenase family proteins (Supplementary Figure S5B).

*ATG18c II* was not located in the syntenic block in *L. japonicus.* Transcriptional regulator *SUPERMAN-like* (legfed_v1_0.L_Tx802x and legfed_v1_0.L_NLQvfk) were specific to *P. vulgaris*. Furthermore, an uncharacterized protein (legfed_v1_0.L_2ffJFT) was found to have undergone duplications in *G. max*, indicating a putative functional role. Pre-mRNA-splicing factor (legfed_v1_0.L_1Bt8v9) was specifically found in milletioid members of legumes, such as *P. vulgaris*, *G. max*, *G. soja* and *V. unguiculata* (Supplementary Figure S5C).

#### 2.8.2. Subfamily II

*ATG18b* was not located in *L. japonicus* or *V. angularis. L. japonicus* exhibited inversions in the syntenic block involving the synthesis of pectic cell wall components, ATPases and DUF788 proteins, which have been proven to be involved in autophagy regulation. *ATG11* was also found in the same syntenic block (Supplementary Figure S5D; Supplementary Information SI5).

#### 2.8.3. Subfamily III

*ATG18f.I* was identified in most of the species compared, and most of the flanking genes were conserved. An important observation from this syntenic block is the tandem duplication of *Histone H2A* (legfed_v1_0.L_0mwghf) in all species except *Arachis* and *Lotus*. *Fe(II)-*dependent *dioxygenase-*like (legfed_v1_0.L_81S90D) was missing in *L. albus* and *L. angustifolia* (Supplementary Figure S6A; Supplementary Information SI5).

*ATG18g.I* was only found in *P. vulgaris*, *C. cajan*, *G. max*, *L. japonicus* and *V. angularis*, and in the other species, the circadian clock-regulated growth regulator Zinc knuckle family protein (legfed_v1_0.L_001qtq) was found in the same syntenic block. The most significant feature of this block was the repeated duplication of disease resistance-responsive dirigent-like protein family protein (legfed_v1_0.L_08frmp) in all the species except *V. angularis*. In *Arachis* species, the clustering of vacuolar protein-sorting protein (legfed_v1_0.L_0c0sd2) and breast carcinoma amplified sequence 3 protein (legfed_v1_0.L_cdgcy6) with other genes was an important observation (Supplementary Figure S6B; Supplementary Information SI5).

*ATG18g.II* was missing in *L. albus* and was well conserved in other species. In *Arachis*, gene clusters involving *FANTASTIC FOUR 3-like* (legfed_v1_0.L_xmq5fm) protein were found associated with shoot meristem growth (Supplementary Figure S6C; Supplementary Information SI5).

### 2.9. ATG18 Protein Characterization

As mentioned previously, *ATG18* homologs in *P. vulgaris* were also divided into three subfamilies with the characteristic motifs FRRG in PvATG18b, VRRG in PvATG18a and PvATG18c, LQRG in PvATG18f and LHRG in PvATG18g. Characterization of the PvATG18 homologs revealed that PvATG18b had the lowest molecular weight, was stable with an isoelectric point of 8.86 and had a high aliphatic index. High-molecular-weight proteins were specifically found in subfamily III (Supplementary Table S2).

Prediction of the subcellular localization of ATG18 homologs showed that ATG18a, c.I, c.II, g.I and g.II were localized in the cytoplasm, and ATG18f.I and f.II were located in the ER membrane and plasma membrane. Only ATG18c homologs were localized to the lumen of lysosomes. ATG18b was unique because it was found in the mitochondrial inner membrane, inner membrane space and ER membrane (Supplementary Table S2). Furthermore, only three of the PvATG18 proteins had a transmembrane helix spanning the aa 44–67 in PvATG18b and located between the aa 12 and 34 in PvATG18f.I and the aa 7 and 26 in PvATG18f.II (Supplementary Figure S7). Furthermore, we predicted the putative phosphorylation sites in PvATG18 homologs and found that these were located on the amino acids threonine and serine in all sequence alignments (Supplementary Figure S8).

### 2.10. Protein Structure Prediction and Molecular Dynamics Simulation of ATG18b in P. vulgaris

**Figure 11:**
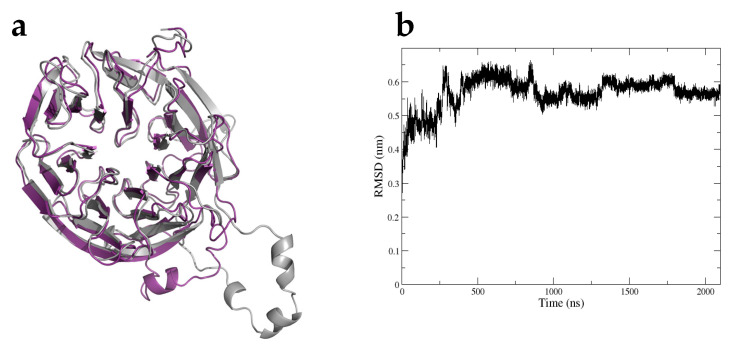
ATG18 structure. (**a**) Three-dimensional structural model of Atg18b before (gray) and after (purple) running the molecular dynamics simulation. (**b**) RMSD of the modeled ATG18b protein over a time period of 2.1 µs.

The above-described analysis implies that PvATG18b is the functional ortholog of AtATG18b; hence, we attempted to understand the structure of this protein using the Robetta Server. This model was submitted to 2.1-µs-long unbiased MD to evaluate the predicted protein model (Figure 11a). In the simulation, we monitored the root mean square deviation (RMSD) of the model protein. The graph clearly indicates a change in the RMSD during the first 1.8 µs of simulation, but the RMSD then reached a plateau. This finding indicates that after 1.8 µs of simulation, the 3D structural model of PvATG18b represents a stable folding conformation (Figure 11b). The model shows the seven-bladed β-propeller architecture conserved among the ATG18 family of proteins [52]. The PvATG18 protein structure consists of seven blades formed by antiparallel β-stands connected by short loop regions. The blades are listed with the numbers 1 to 7 beginning at the C-terminus, whereas the β-stands are named with letters from an inner to outer location as A to D. These structures were similar to those observed with the biophysical characterization of PROPPIN ATG18 in *Pichia angusta* [52]. We also found a CD loop (S269 to T288) located between the two phosphoinositide-binding sites and the FRRG motif at positions F218, R219, R220 and G221 between blades 5 and 6 (Figure 10d). PROPPINs are WD-40 family propeller proteins that act as scaffolds for protein–protein interactions. The binding of PvATG18b to PtdIns(3,5)P_2_ and PtdIns3P might be mediated by additional protein–protein interactions, as observed in *Kluyveromyces lactis* [37]. Earlier models of PROPPINS predicted the insertion of two loops into the membrane in a perpendicular orientation in the phagophore membrane through nonspecific electrostatic interactions [53,54]. Our results for PvATG18 reveal the previously reported nonspecific electrostatic interaction in the protein structure and the presence of one transmembrane motif (Figure 10b, c.)

## 3. Discussion

Autophagy is recognized as a highly selective cellular clearance pathway that helps maintain homeostasis in eukaryotic cells. The genes involved in autophagy are highly conserved from yeast to humans, and the process is the result of the interaction of these *ATG*s and other associated genes. The number of identified *ATG*s shows a marked variation among different species. In yeast, a total of 41 genes have been identified to date, and several studies on plant *ATG*s have also identified a varied number of genes. In the present investigation, we attempted to perform a comprehensive study for identifying *ATG* families in three important legume species, namely, *P. vulgaris, M. truncatula* and *G. max*. Furthermore, we focused on the *ATG18* gene family, the largest of all the families, to identify and phylogenetically compare 27 plant species starting from early plant lineages, chlorophytes to higher plants including legumes.

### 3.1. Autophagy Genes in Legumes Are Highly Conserved

Using *Arabidopsis ATG*s as a reference, we retrieved *ATG* homologs in all the species listed in various databases, including Phytozome, and the sequences were confirmed to be affiliated with ATG-like homologs by analyzing their Pfam matches in the Pfam database. We identified a total of 32, 28 and 61 *ATG* homologs in *P. vulgaris*, *M. truncatula* and *G. max*, respectively. The identified homologs could be classified into 17 families based on their phylogenetic relationships and motifs. The phylogenetic analysis revealed that homologs in *Medicago* were located closer to *Arabidopsis* than those in other species. Unlike in yeast, which contains a single copy of each family, many of the gene families have multiple copies. *ATG1* has 4, 3, 2 and 6 homologs in *Arabidopsis*, *Medicago*, *Phaseolus* and *Glycine*, respectively, *ATG13* has 2 homologs in *Arabidopsis*, *Medicago* and *Phaseolus* (2 in each) and 4 homologs in *G. max*, *ATG9* has 2 or 4 homologs in *Medicago*, *Phaseolus* and *G. max* and *ATG14* and *ATG4* have 2 homologs in *Arabidopsis* and 2 homologs in *G. max*. The analysis of larger families revealed that *ATG8* has 9, 6, 7 and 10 homologs in *Arabidopsis*, *Medicago*, *Phaseolus* and *G. max*, respectively, and that *ATG18* has 8 homologs in *Arabidopsis*, *Medicago* and *Phaseolus* (8 in each) and a maximum of 19 homologs in *G. max*. Similar results were also obtained with *O. sativa* [55], *Nicotiana tabacum* [56], *Vitis vinifera* [57], *Musa acuminate* [58] and *Setaria italic* [59]. However, in most of the families, the homologs were placed in one clade, which clearly showed sequence similarity and the derivation of statistically reliable pairs of possible orthologous proteins sharing similar functions from a common ancestor, consistent with the results from a previous study conducted by Kellogg (2001) [60]. Furthermore, the *ATG* families identified constituted a relatively complete autophagic machinery in forming the complexes, namely, the ATG1 kinase complex, class III PI3K complex, ATG9 recycling complex, Atg8-lipidation system and Atg12-conjugation system.

ATG17 is an important accessory protein along with ATG31-ATG29, which acts as a scaffold/modulator in linking the ATG1-ATG13 complex to the phagophore assembly site in yeast. Homologs of the ATG17-ATG31-ATG29 subcomplex were not detected in *Arabidopsis*. However, single orthologs of ATG11 and ATG101 were identified, and ATG11 reportedly contains a short cryptic ATG17-like domain with weak identity to yeast ATG17 [61]. The identification of ATG homologs in the present study revealed one homolog of ATG11 and one homolog of ATG101 in all the legumes analyzed.

For further exploration of the origin and evolutionary process of ATGs, a comparative synteny map that depicted the presence of 160 genes in *Arabidopsis* and three legumes compared was constructed. The results suggested that the majority of ATGs had a common ancestor. The Ka/Ks ratio is an important genetic parameter for determining whether positive Darwinian selection is related to gene differentiation [62]. Positive Darwinian selection will retain the advantages of nonsynonymous mutations, and purification selection will gradually remove deleterious nonsynonymous mutations. Herein, the Ka/Ks ratio among most of the ATG sequences was lower than 1 (average of 0.17), indicating purifying selection; in contrast, the sequences of *ATG8* (1.24) and two *ATG18s* (1.09 and 1.04) in *G. max* had higher values, indicating accelerated evolution and positive selection.

Plant macroautophagy is a process in which macromolecules and cellular components are recycled in lytic vacuoles to be reused. Recycling is crucial for the maintenance of cellular homeostasis by acting as a quality control mechanism under nonstressful conditions and is stimulated under stress conditions [63]. Stress-induced autophagy is well documented in some plant species. Our study of the transcription factors binding to the ATGs revealed that several light-responsive transcription factors, such as BOX-4, G-box, GT1-motif, MRE and ACE, were abundant in most of the ATGs. Furthermore, *cis*-acting elements related to circadian control were also identified. Phytohormones play key roles in different plant processes, including stress responses. The ATGs analyzed exhibited TF-binding sites for EREs, ABA-responsive ABREs, MeJA-responsive CGTCA motifs, auxin-responsive TGA elements and gibberellin-responsive GARE motifs. Ethylene is considered a key regulator of autophagy in petal senescence in petunia, and ERF5 is also shown to induce autophagy by binding to ATG8 and ATG18h under drought stress in tomato. Upregulation of autophagy by low concentrations of salicylic acid is found to delay methyl jasmonate-induced leaf senescence in *Arabidopsis* [64,65,66]. In addition, several wound-responsive, pathogen-responsive, flavonoid biosynthetic gene regulation-related and meristem-specific elements were also detected. Based on all the results, the involvement of autophagy in the regulation of plant responses to biotic and abiotic stresses is undeniable.

### 3.2. Autophagy Genes Are Responsive to Nitrate

To assess the differential expression pattern and responsive nature of *ATG*s to the presence of different nitrate sources, we developed heatmaps using the data retrieved from databases and from a previous RNA-seq analysis performed by our research group. The differential expression pattern in Phaseolus tissues showed that most of the ATGs were expressed in all tested tissues. Nitrogen is an essential component of life that is needed for building proteins and DNA, and despite its abundance in the atmosphere, only limited reserves of soil inorganic nitrogen are accessible to plants, and this nitrogen is primarily in the forms of nitrate and ammonium. Legumes have a unique ability to establish a symbiotic association with nitrogen-fixing rhizobia. Due to our understanding of the evolution of *ATG*s in legumes, we opted to understand the response of both arial and root tissues of these legumes to different nitrate sources. The expression patterns showed that the highest expression was found in roots treated with ammonia and leaves treated with urea. *ATG18* homologs a, g and h were specifically induced in all tissues and by all treatments, indicating the nitrate-responsive nature of these genes.

Furthermore, an analysis of the differential expression patterns of *ATG*s in *Phaseolus* tissues revealed that the highest expression level was noted in 21-day fix (-) nodules, which could be due to the involvement of the autophagic process in providing the necessary amino acids for the synthesis of nitrogen in the absence of the symbiont. In yeast and other eukaryotes, it has been proven that nitrogen deficiency induces autophagy. A recent study using yeast cells also suggested that autophagy sustains glutamate and aspartate synthesis during nitrogen starvation [67]. RNA-seq data from early symbiosis with rhizobia and mycorrhizae showed differential ATG expression, and more *ATG*s were upregulated in rhizobia-inoculated roots than in mycorrhizae-inoculated roots. This analysis provided candidate genes that could play pivotal roles in symbiosis. The involvement of *ATG6/beclin* has previously been reported in *P. vulgaris* during rhizobial infection progression and arbuscule maturation [68].

### 3.3. The ATG18 Family Is Highly Conserved and Has a Broader Sequence-Based Classification

*Atg18* is one of the autophagy-related molecules responsible for autophagic processes and is conserved from yeast to higher organisms [34]. ATG18 proteins belong to the PROPPINs (β-propellers that bind polyphosphoinositides) family and work as PI3P effectors. Earlier studies that focused on the identification of ATG genes in primitive and higher plants showed that each family is represented by only one gene for each component of the core autophagy machinery. *ATG8* and *ATG18* are exceptions and have multiple homologs with lower redundancy in *Arabidopsis* and *P. patens* [51].

ATG18 was the family with the highest number of homologs; hence, we chose this family for a comprehensive analysis of the family from the early plant lineage to legumes. The multiple sequence alignment and phylogeny of ATG18 homologs resulted in separation of the homologs into three clades. Each of the clades had subfamily members, as determined by the multidimensional scaling projection of 280 ATG18 homologs in 27 photosynthetic organisms. Unlike previous studies by Norizuki and colleagues [51], the classification of the ATG18 family was not based on the BCAS3 domain alone. Knockout of the BCAS3 gene in *Dictyostelium* resulted in a reduction in early autophagosomes compared with that found in wild-type cells [69]. In the present study, due to the multidimensional scaling projection of the retrieved sequences, we classified the ATG18 sequences into three subfamilies. Subfamily I contained ATG18a, ATG18c, ATG18d and ATG18e homologs, subfamily II had only ATG18b and subfamily III had ATG18f, ATG18g and ATG18h members. All homologs with BCAS3 were found to be clustered within subfamily III.

Subfamily II, which contained only ATG18b homologs, had few members but was detected in all the plant species investigated in this study, which suggested the sequence and functional conservation of these proteins. Among the early photosynthetic organisms, we identified at least one homolog in subfamilies I and II, but significant divergence was detected, particularly within subfamily III. Among monocots, *O. sativa* had 8 homologs, whereas 32 and 21 homologs were found in *Z. mays* and *T. aestivum*, respectively. The analysis of dicots revealed 8 homologs in each of *Arabidopsis, L. japonicus, M. truncatula* and *P. vulgaris*, whereas *Arachis* sp. had 9 and 10. The maximum number of homologs was recorded in *C. cajan* (18), *G. max* (18), *C. arietinum* (20), *Vigna* sp. and *L. angustifolius* (27).

The legume family includes one of the most agroeconomically important plant crops after Poaceae [70]. Of the three subfamilies within Fabaceae, Papilionoideae is the largest, the most recently evolved and monophyletic. Because Papilionoideae includes the most important cultivated legumes, we sought to determine the members of this subfamily in different clades. In the present study, the maximum number of homologs (27) was identified in *L. angustifolius*, which belongs to the genistoid clade and exhibited an early divergence at approximately 56.4 ± 2 mya. Furthermore, in *Arachis* species, we found less than half of the ATG18 homologs, indicating possible deletions. Among the members of the next recent (45 mya) clade, which consisted of milletoids, an increase in the number of homologs (18) was detected, which might be due to whole-genome duplication in *G. max*. However, *P. vulgaris* had only eight members of ATG18, indicating possible divergence prior to whole-genome duplications, whereas *Vigna* sp. was found to have high numbers of homologs. Furthermore, more recent robinioid (48.3 ± 1.0 mya) and IRLC (39.0 ± 2.4 mya) clade members had fewer members with the exception of the tribe Vicieae, whose gene numbers were due to genome expansion and related genomic events. In contrast, syntenic relations were not disrupted due to differences in genome sizes [71,72]. A phylogenetic analysis revealed that the ATG18 homologs of Chlorophyta, Charophyta, Marchantiophyta and Bryophyta were always grouped together, and similar results were obtained for monocots and dicots. However, in a comparison of a broad class of species, it is often not simple to precisely define orthologous genes or genomic loci in a straightforward manner, and this analysis is complicated due to gene duplication, recurring polyploidy and extensive genome rearrangement [73].

### 3.4. The ATG18 Protein Structure Predicts Possible Functional Diversification

In addition, the prediction of the primary and secondary structures of the proteins strengthens the classification of ATG18 proteins into subfamilies. The protein size, motif structure and changes in FRRG motifs among the ATG18 homologs were identified as the fundamental features that contribute to the classification. The changes in the FRRG motifs found in members of subfamily II comprising ATG18b to LRRG, VRRG in subfamily I, LQRG, LHRG or LYRG in subfamily III indicate functional diversification. The WD40 domain is among the top ten most abundant domains in eukaryotic genomes and is also ranked as the top interacting domain in *S. cerevisae* [74] (Stirnimann et al., 2010). Based on the SMART database, the human genome contains approximately 349 WD40 domain-containing proteins [75]. The presence of the WD40 domain in ATG18 homologs could indicate their involvement in cellular functions. Proteins containing WD40 domains are known to be involved in signal transduction, vesicular trafficking, cytoskeletal assembly, cell cycle control, apoptosis, chromatin dynamics and transcription regulation due to their ability to bind and thus function as interchangeable substrate receptors to target different substrates and recruit different substrates in distinct modes [76]. In *C. elegans*, ATG18 and WIPI 1/2 (WD-repeat protein interacting with phosphoinositides) in mammals have FRRGs and EPG-6 and WIPI 3/4 have LRRGs. The substitution of the FRRG motif by FTTG and FKKG does not allow PtdInsP binding; however, the changes in LKKG and LTTG still allow PtdInsP binding [77], implying a possible functional diversification of ATG18 homologs. The studies conducted thus far also demonstrate the involvement of ATG18 homologs in abiotic stress responses in plants [42,43,44,45,46,47,48,49,50].

### 3.5. ATG18 Family in P. vulgaris

In *P. vulgaris*, a total of eight ATG18 homologs were identified in the current study and were also classified into three subfamilies. While the functional roles of these subfamilies were not determined in this study, the involvement of these proteins in diversified cellular functions cannot be ruled out. All the subfamilies showed conserved phosphorylation sites but different subcellular localizations.

The conserved nature of serine/threonine sites could indicate the functional roles corresponding to several cellular responses in *P. vulgaris*. In yeast, *Pichia pastoris*, Atg18 phosphorylation in the loops in the propeller structure of blades 6 and 7 decreases its binding affinity to phosphatidylinositol 3,5-bisphosphate. The association of ATG18 with the vacuolar membrane is inhibited until dephosphorylation [78]. A recent study in *Arabidopsis* showed that the phosphorylation of ATG18a by brassinosteroid insensitive 1-associated receptor kinase 1 (BAK1) suppresses autophagy and attenuates plant resistance against necrotrophic pathogens [79].

The microsynteny of *P. vulgaris* ATG18 homologs showed that subfamily I members were highly conserved across the compared species and were flanked by genes involved in cell cycle regulation, transcriptional regulation, cellular transport and metal ion binding. Furthermore, subfamily II was flanked by the ATPase and DUF788 proteins, which have been proven to be involved in autophagy regulation. ATG11, which is a part of the ATG13-ATG1 complex in autophagy initiation, was also found in the same syntenic block. The subfamily III syntenic block contained conserved genes related to histones, circadian clock, growth and vacuolar transport.

### 3.6. PvATG18b Could Be the Homolog of AtATG18b

In accordance with a well-established fact, the most important feature of ATG18 proteins is the presence of the FRRG motif and its ability to bind to phosphoinositide. Among *P. vulgaris* ATG18 homologs, the FRRG motif was found only in ATG18b belonging to subfamily II. Hence, we propose PvATG18b as the functional homolog of *A. thaliana* ATG18b. We also hypothesize that other ATG18 homologs might be involved in other molecular recognition events through binding to surface molecules that play a distinctive role in autophagy, and similar findings have been observed with human ATG18 homologs, e.g., WIPI 1/WIPI 2 with FRRG repeats and WIPI 3/WIPI 4 with LRRG repeats bind to various PtdIns and thus play distinct roles in autophagy [76,80].

We then performed a molecular dynamic simulation of PvATG18b that is unique to ATG models in legumes. Our model shows the stable folding conformation of the seven-bladed β-propeller architecture. PvATG18b is composed of 359 amino acids, and we found the CD loop (S269 to T288) in blade 6. While this loop sequence differs among species, it forms an amphipathic alpha-helix and might insert into a membrane to allow two lipid-binding sites (PtdIns3P and PtdIns(3,5)P_2_) [81]. Additionally, PvATG18b contains the FRRG repeat and helps form the site for binding to lipids. The FRRG repeat is in F218 to G221 and is conserved in ATG18b to form the PROPPIN family. The FRRG motif (Phe-Arg-Arg-Gly) in ATG18 proteins has been studied in mammals, yeast and *C. elegance* [79,82]. In *Kluyveromyces lactis*, the mutation of the blade 6 β3-β4 loop affects the loss of liposome binding, and the flexible loop coordinates two distinct lipid-binding sites [83]. Previous studies with *S. cerevisiae* have demonstrated that loops A and B of blade 7 are the locations where ATG2 interacts with ATG18. Further research should be performed to understand the interaction of ATG18 with ATG2 and thus ensure the binding site and vacuole scission function of PvATG18b.

## 4. Materials and Methods

### 4.1. Identification of ATG Families in Legumes

*Arabidopsis* (taxid: 3702) ATG family gene sequences were retrieved from the Araport (https://www.araport.org; accessed on 13 May 2020) and TAIR (https://www.arabidopsis.org; accessed on 15 May 2020) databases through Phytozome v.13. Using these sequences, a BLAST [84] (http://www.ncbi.nlm.nih.gov; Stephen et al., 1997; accessed on 19 May 2020) search was conducted to identify the homologs of *ATG* genes in *Phaseolus vulgaris* v 2.1 (taxid: 3885), *Medicago truncatula* Mt4.0v1 (taxid: 3880) and *Glycine max* Wm82.a2.v1 (taxid: 3847). The stringency of the search was maintained by keeping the mean BLAST results within a query coverage of 93.85% and 67.78% identity.

The detection of homologs was further optimized using other programs, such as KEGG (www.genome.jp/kegg/; accessed on 2 June 2020) [85], Ensembl Plants (https://plants.ensembl.org; accessed on 4 June 2020) [86], HMMER suite server (http://hmmer.org; accessed on 4 June 2020) [87] and InParanoid 4.1 [88]. Additionally, we examined the ontology IDs for all ATG families using KOG (EuKaryotic Orthologous subfamilies) in the EggNOG v5.0 database [89] (http://eggnog.embl.de; accessed on 7 June 2020) and Protein ANalysis THrough Evolutionary Relationships (PANTHER v14.0, http://www.pantherdb.org; accessed on 10 June 2020) and Pfam IDs were identified in Portal v33.1 (http://pfam.xfam.org/about accessed on 30 October 2020).

The *ATG18* protein family was studied in 27 photosynthetic organisms, 13 dicots (legumes), 3 monocots and 10 plants through the evolution of land plants from an algal ancestor. We obtained the ATG18 protein sequences of monocotyledonous crops such as *Zea mays* (taxid: 4577), *Triticum aestivum* (taxid: 4565) and *Oryza sativa* (rice, taxid: 4530) and legumes such as *Arachis duranensis* (peanut, taxid: 130453), *Arachis ipaensis* (taxid: 130454), *Cajanus cajan* (taxid: 3821), *Lotus japonicus* (taxid: 34305), *Cicer arietinum* (taxid: 3827), *Lupinus angustifolius* (taxid: 3871), *Pisum sativum* (pea, taxid: 3888), *Vigna angularis* (taxid: 3914), *Vigna radiata* (taxid: 157791) and *Trifolium pratense* (red clover, taxid: 57577) through a BLAST analysis of the NCBI, Phytozome, LegumeInfo (https://legumeinfo.org; accessed on 18 June 2020), KEGG, InParanoid, Ensembl, EggNOG and Pfam databases. Additionally, we used the Norizuki report of early-divergent plant lineages to extract the ATG18 protein sequences of Bryopsida (*Physcomitrella patens*, taxid: 3218), Charophyceae (*Chara braunii*, taxid: 69332), Chlorophyceae (*Chlamydomonas reinhardtii*, taxid: 3055, *Dunaliella salina*, taxid: 3046), (*Volvox carteri*, taxid: 3067), Klebsormidiophyceae (*Klebsormidium nitens*, taxid: 105231), Mamiellophyceae (*Micromonas pusilla*, taxid: 38833; *Ostreococcus lucimarinus*, taxid: 242159; *Ostreococcus tauri*, taxid: 70448) and Trebouxiophyceae (*Coccomyxa subellipsoidea*, taxid: 248742) [51].

### 4.2. Alignment and Phylogenetic Tree Analyses

The protein sequences of ATG families were aligned using Clustal Omega (1.2.4) [90] (www.clustal.org and www.ebi.ac.uk; accessed on 5 July 2020) with the default parameters. The phylogenetic tree was a neighbor-joining tree without distance corrections, and we extracted the outputs from the tree and generated circular phylogram and cladogram tree images using EvolView. The different phylogenetic trees were combined with the MEME results for all sequences, and the final details were obtained using Inkscape software [91] (https://www.evolgenius.info/evolview/; accessed on 6 July 2020).

Multiple sequence alignment of 280 intraspecies protein sequences of ATG18 family members was performed using Clustal Omega. The phylogenetic analysis was performed using MEGA X with the maximum likelihood method and Bayes analyses with 1000 bootstrap replicates and the default parameters [92]. Phangorn and APE packages in R were used to build the phylogenetic trees [93,94]. In Phangorn, we used the Akaike information criterion and the Whelan and Goldman matrix (WAG) as the substitution model.

### 4.3. Chromosome Localization, Synteny and Ka/Ks Calculation

The chromosomal localization of ATG family homologs in *A. thaliana, P. vulgaris, M. truncatula* and *G. max* was verified using NCBI. Furthermore, Ensembl Plants was used to compare and explore the gene alignments and generate a segment to link the genomes. The synteny relation of ATG genes was drawn using OmicCircos in R36 [95]. The macro- and microsynteny of the ATG18 family was developed using the Genome Context Viewer (GCV) in the Legume information system [96] (https://legumeinfo.org/lis_context_viewer/instructions; accessed on 16 July 2020).

The CDSs and protein sequences were obtained from Phytozome and used to calculate the synonymous substitutions (Ks) and nonsynonymous substitutions (Ka) with TBtools software (https://github.com/CJ-Chen/TBtools; accessed on 26 July 2020). Using the data table, we developed a graph of the Ka and Ks values for all ATG families in *P. vulgaris, M. truncatula* and *G. max* using the ggplot2 R packages (https://ggplot2.tidyverse.org/; accessed on 28 July 2020).

### 4.4. Promoter Analysis, Expression Profiling and Transcriptome of ATG Families

The 2000-bp upstream sequences of *ATG* genes were retrieved from Phytozome, and these sequences were used as query sequences in PlantCARE software (http://bioinformatics.psb.ugent.be/webtools/plantcare/html/; accessed on 2 August 2020). The results were analyzed, and the most abundant transcription factors were identified using ggplot2 in R.

*ATG* gene expression data for *A. thaliana, M. truncatula* and *G. max* were extracted from Phytozome to determine the differential expression of the genes under different nitrogen treatments [97]. Data on the differential expression of genes in *P. vulgaris* under nitrogen treatments and after fixation and inoculation with *Rhizobium tropici* (CIAT899) were obtained from the PvGEA website (https://plantgrn.noble.org/PvGEA/; accessed on 2 July 2020).

We calculated the log2 values of the RPKM values for the comparison. To show the data for *A. thaliana, M. truncatula* and *G. max*, we used the OmicCircos package and constructed subfamilies using the synteny graph. However, for *P. vulgaris*, we constructed an independent heatmap of ggplot2 because the amounts of treatments and tissues were higher. The expression data for ATGs under rhizobial and mycorrhizal symbiotic conditions were obtained from our previous global transcriptomic analysis [98]. A heatmap of the fold change values was constructed using the ggplot2 package.

### 4.5. Quantitative Real-Time PCR Analysis

Four genes were selected for RT-qPCR analysis, which was performed to validate the RNA-seq data. High-quality total RNA was isolated from frozen root tissues using TRIzol reagent (Sigma) according to the manufacturer’s instructions. RNA integrity was verified by gel electrophoresis and RNA concentration was assessed using a NanoDrop spectrophotometer (Thermo Scientific). RNA was treated with DNase to eliminate DNA contamination (1 U/μL; Roche, USA) according to the manufacturer’s instructions. Reverse-transcription quantitative PCR (RT-qPCR) analysis was performed using a DNA-free RNA and iScript^TM^ One-Step RT-PCR Kit with SYBR^®^ Green (Bio-Rad) according to the manufacturer’s instructions. To confirm the absence of DNA contamination, a sample lacking reverse transcriptase was included. Relative expression values were calculated using the 2^-ΔCt^ method, where the quantification cycle (Cq) value equals the Cq value of the gene of interest minus the Cq value of the reference gene [99]. Gene-specific primers were used for RT-qPCR analysis (Table S3). *Pv*EF1α and *Pv*IDE were used as reference as described previously by Arthikala et al. [100]. The relative expression values were normalized with respect to two reference genes EF1α and IDE as described previously by Vandesompele et al. [101]. The values presented are averages of three biological replicates, and each data set was recorded using triplicate samples.

### 4.6. Principal Components Analysis of the ATG18 Family

Based on multiple alignments of ATG18 protein sequences, we converted the information into a distance matrix calculated using the bios2mds packages (https://CRAN.R-project.org/package=bios2mds; accessed on 3 July 2020) in R. The matrix used was BLOSUM62 (BLOcks of Amino Acid SUbstitution Matrix), and sequences with 62% identity were obtained. Using the same packages, we obtain the K-means and principal components to generate the multidimensional scaling projection and thus define the subfamilies within the protein family.

### 4.7. Detection of Motifs, Domains, Repeats, Families and Secondary Protein Structure of the ATG18 Family

ATG sequences were analyzed for a repeated sequence motif pattern using Multiple Expectation Maximization for Motif Elicitation [102] (http://meme-suite.org/tools/meme; accessed on 18 July 2020) in the classical motif discovery mode and using a limit of three motifs. The secondary structures of the proteins were developed after alignment with Clustal Omega using the online tool JPred in FASTA format. To obtain the repeats, domains and families, a Pfam scan of EMBL-EBI was performed (https://www.ebi.ac.uk/Tools/pfa/pfamscan/; accessed on 26 August 2020).

### 4.8. Microsynteny and Protein Sequence Parameters of ATG18 in P. vulgaris

The computed parameters for PvATG18, including the molecular weight, theoretical pI, amino acid composition, atomic composition, extinction coefficient, estimated half-life, instability index, aliphatic index, grand average of hydropathicity (GRAVY), phosphorylation sites, predicted transmembrane helixes and subcellular localization, were obtained using ProtParam, PSORT, THMHMM and NetPhos 51 (https://web.expasy.org; accessed on 5 July 2020). The ATG positions were extracted from Phytozome, and microsynteny calculations were generated using GCV v1.2.0 [103] (https://legumeinfo.org/lis_context_viewer/; accessed on 6 August 2020).

### 4.9. ATG18b Protein in P. vulgaris

The 3D structure of the PvATG18b protein was determined using the Robetta server [102]. Comparative models were built from structures detected and aligned using HHSEARCH, SPARKS and Raptor [104,105,106,107]. The loop regions were assembled from fragments and optimized to fit the aligned template structures. The final structure prediction was selected using the lowest-energy model as determined by a low-resolution Rosetta energy function. The final 3D image was colored with Quimera [108].

## 5. Conclusions

The present study was carried out to understand the diversification of ATG genes during plant evolution with special emphasis on legumes and *P. vulgaris*. In the present study, we identified 32, 39 and 61 core ATG genes in *P. vulgaris*, *M. truncatula* and *G. max*, respectively. The ATG genes were conserved across the species, but the higher plants revealed great redundancy. Most of the ATGs in *Phaseolus* were found to be nitrate responsive and were differentially expressed under rhizobial and mycorrhizal symbiosis, implying their possible role during symbiosis. Further, analysis ATG18 of the family in 27 photosynthetic organisms showed their classification into three subfamilies based on the sequence. In *Phaseolus*, ATG18 members belonging to all the three subfamilies were conserved. Comparison of *Phaseolus* ATG18b structure to the crystal structure in *Arabidopsis* showed conserved FRRG sequence.

## Figures and Tables

**Table 1:** List of 17 autophagy gene families in *A. thaliana, P. vulgaris, M. truncatula* and *G. max*.

			*Arabidopsis thaliana*		*Phaseolus vulgaris*		*Medicago truncatula*		*Glycine max*	
	Complex	Family	Name	ID	Name	ID	Name	ID	Name	ID
Initiation of autophagy	ATG1 complex	ATG1	AtATG1a	At3g61960			MtATG1a	Medtr8g024100	GmATG1a.I	Glyma.07g048400
									GmATG1a.II	Glyma.16g017300
			AtATG1b	At3g53930	PvATG1b	Phvul.010g015100	MtATG1b	Medtr4g019410	GmATG1b.I	Glyma.03g069800
			AtATG1c	At2g37840					GmATG1b.II	Glyma.01g099600
			AtATG1t	At1g49180	PvATG1t	Phvul.010g120500	MtATG1t	Medtr3g095620	GmATG1t.I	Glyma.06g150700
									GmATG1t.II	Glyma.04g215500
		ATG11	AtATG11	At4g30790	PvATG11	Phvul.003g153800	MtATG11	Medtr4g130370	GmATG11	Glyma.17g071400
		ATG13	AtATG13	At3g49590	PvATG13a	Phvul.008g187800	MtATG13a	Medtr5g068710	GmATG13a.I	Glyma.02g220700
									GmATG13a.II	Glyma.14g187000
			AtATG13b	At3g18770	PvATG13b	Phvul.002g269600	MtATG13b	Medtr3g095570	GmATG13b.I	Glyma.05g189000
							MtATG13c	Medtr8g093050	GmATG13b.II	Glyma.08g146700
		ATG101	AtATG101	At5g66930	PvATG101	Phvul.003g248000	MtATG101	Medtr8g079240	GmATG101	Glyma.17g180900
Membrane recruitment to autophagosomez	Complex ATG2-ATG18	ATG9	AtATG9	At2g31260	PvATG9a	Phvul.001g159900	MtATG9a	Medtr7g096680	GmATG9a.I	Glyma.03g162100
									GmATG9a.II	Glyma.19g163500
					PvATG9b	Phvul.007g194300	MtATG9b	Medtr1g070160	GmATG9b.III	Glyma.10g035800
									GmATG9b.vI	Glyma.13g122200
		ATG2	AtATG2	At3g19190	PvATG2	Phvul.003g295800	MtATG2	Medtr4g086370	GmATG2.I	Glyma.02g133400
									GmATG2.II	Glyma.07g211600
		ATG18	AtATG18a	At3g62770	PvATG18a	Phvul.001g205000	MtATG18a	Medtr1g083230	GmATG18a.I	Glyma.10g152500
									GmATG18a.II	Glyma.20g235800
									GmATG18a.III	Glyma.03g212100
									GmATG18a.Iv	Glyma.19g209200
			AtATG18b	At4g30510	PvATG18b	Phvul.003g152800	MtATG18b	Medtr4g130190	GmATG18b.I	Glyma.17g070200
									GmATG18b.II	Glyma.02g207500
									GmATG18b.III	Glyma.10g126200
			AtATG18c	At2g40810	PvATG18c.I	Phvul.009g041700	MtATG18c	Medtr7g108520	GmATG18c.I	Glyma.04g224300
					PvATG18c.II	Phvul.007g196400			GmATG18c.II	Glyma.06g140400
			AtATG18d	At3g56440			MtATG18d	Medtr1g088855		
			AtATG18e	At5g05150			MtATG18e	Medtr3g093590	GmATG18e	Glyma.16g109400
			AtATG18f	At5g54730	PvATG18f.I	Phvul.011g140900	MtATG18f	Medtr2g082770	GmATG18f.I	Glyma.12g214600
					PvATG18f.II	Phvul.005g091300			GmATG18f.II	Glyma.12g136000
									GmATG18f.III	Glyma.13g287000
									GmATG18f.IV	Glyma.06g267000
			AtATG18g	At1g03380	PvATG18g.I	Phvul.001g146700	MtATG18g	Medtr1g089110	GmATG18g.I	Glyma.03g148700
								
					PvATG18g.II	Phvul.007g183100			GmATG18g.II	Glyma.19g152000
									GmATG18g.III	Glyma.20g230900
			AtATG18h	At1g54710			MtATG18h	Medtr1g082300	GmATG18h	Glyma.10g157700
Autophagosome formation		ATG6	AtATG6	At3g61710	PvATG6	Phvul.005g029900	MtATG6	Medtr3g018770	GmATG6.I	Glyma.11g153900
									GmATG6.II	Glyma.04g141000
	PI3K complex	ATG14	AtATG14a	At1g77890	PvATG14	Phvul.008g169200	MtATG14	Medtr5g061040	GmATG14.I	Glyma.13g085400
									GmATG14.II	Glyma.14g167200
			AtATG14b	At4g08540						
Ubiquitin-like protein conjugation systems	Ubiquitin-like conjugation (ATG8)	ATG3	AtATG3	At5g61500	PvATG3	Phvul.011g006500	MtATG3	Medtr4g036265	GmATG3.I	Glyma.12g005700
									GmATG3.II	Glyma.09g231000
			AtATG4a	At2g44140	PvATG4a	Phvul.008g048900	MtATG4a	Medtr7g081230	GmATG4a.I	Glyma.18g248400
		ATG4							GmATG4a.II	Glyma.09g244800
			AtATG4b	At3g59950						
		ATG7	AtATG7	At5g45900	PvATG7	Phvul.011g010700	MtATG7	Medtr0003s0540	GmATG7	Glyma.12g010000
		ATG8	AtATG8a	At4g21980			MtATG8a	Medtr2g023430		
			AtATG8b	At4g04620			MtATG8b	Medtr4g037225	GmATG8b	Glyma.15g188600
			AtATG8c	At1g62040	PvATG8c.I	Phvul.003g079300	MtATG8c	Medtr4g048510	GmATG8c.I	Glyma.12g098400
					PvATG8c.II	Phvul.006g149640			GmATG8c.II	Glyma.06g306300
									GmATG8c.III	Glyma.09g003900
									GmATG8c.IV	Glyma.17g013000
									GmATG8c.V	Glyma.07g261000
									GmATG8c.VI	Glyma.15g108200
			AtATG8d	At2g05630	PvATG8d	Phvul.011g103300	MtATG8d	Medtr2g088230		
			AtATG8e	At2g45170			MtATG8e	Medtr4g101090		
			AtATG8f	At4g16520	PvATG8f.I	Phvul.003g219600	MtATG8f	Medtr1g086310	GmATG8f	Glyma.17g140700
					PvATG8f.II	Phvul.002g062200				
			AtATG8g	At3g60640			MtATG8g	Medtr4g123760		
			AtATG8h	At3g06420			MtATG8h	Medtr7g096540		
			AtATG8i	At3g15580	PvATG8i	Phvul.007g210800			GmATG8i	Glyma.02g008800
		ATG5	AtATG5	At5g17290	PvATG5	Phvul.008g241000	MtATG5	Medtr5g076920	GmATG5.I	Glyma.14g210200
									GmATG5.II	Glyma.02g240700
	Ubiquitin-like conjugation (ATG12)	ATG10	AtATG10	At3g07525	PvATG10	Phvul.010g036300	MtATG10	Medtr8g010140	GmATG10	Glyma.03g097000
		ATG12	AtATG12a	At1g54210						
			AtATG12b	At3g13970	PvATG12b	Phvul.010g130300	MtATG12b	Medtr8g020500	GmATG12b.I	Glyma.07g038100
									GmATG12b.II	Glyma.16g007300
		ATG16	AtATG16	At5g50230	PvATG16	Phvul.003g207100	MtATG16a	Medtr3g075400	GmATG16.I	Glyma.05g043700
							MtATG16b	Medtr4g104380	GmATG16.II	Glyma.17g126200
							MtATG16c	Medtr4g007500		

**Table 2:** List of ATG18 homologs in early plant lineages.

		*Chlorophyta*							*Charophyta*		*Liverworts*	*Bryophyta*		*Monocots*		*Arabidopsis*
		*Dunaliella salina*	*Volvox carteri*	*Ostreococcus tauri*	*OOstreococcus lucimarinus*	*Micromonas pusilla*	*Coccomyxa subellipsoidea*	*Chlamydomonas reinhardtii*	*Chara braunii*	*Klebsormidium nitens*	*Marchantia polymorpha*	*Physcomitrella patens*	*Oryza sativa*	*Zea mays*	*Triticum aestivum*	*Arabidopsis thaliana*
Subfamily I	A	DsATG18 (Dusal.0227s00002.1)	VcATG18 (Vocar.0005s0363)	OtATG18 (Ot06g00830)	OlATG18 (OlATG18.3284. fragment)	MpuATG18 (MpuATG1849616)	CsubATG18 (CsATG18.65175)	CrATG18 (Cre10.g425750.t1)	CbATG18 (CHBRA95g00960)	KnATG18 (kfl00229.0060)	MpoATG18a.I (MARPO.0005s0065)	PpATG18 (Phpat.005G022700)	OsATG18a (XP.015621196)	ZmATG18a (Zm00001d011920)	TaATG18a.I (CDM86058)	AtATG18a (AT3G62770)
											MpoATG18a.II (MARPO.0001s0033)	PpATG18 (Phpat.006G095100)			TaATG18a.II (AGW81806)	
												PpATG18 (Phpat.017G015900)			TaATG18a.III (Traes.3B.19AF6BFF0)	
															TaATG18a.IV (TRAES.3B.113DC4275)	
														ZmATG18b.IV (Zm00001d042215.T002)		
														ZmATG18b.V (GRMZM2G143211)		
	C													ZmATG18c.I (AQK90439)	TaATG18c.I (Traes.3DS.985ED34D7)	AtATG18c (AT2G40810)
														ZmATG18c.II (Zm00001d008691)	TaATG18c.II (Traes.3AS.71D103050)	
														ZmATG18c.III (GRMZM2G069177)	TaATG18c.III (TraesCS3B02G110900)	
														ZmATG18c.IV (AQK90440)	TaATG18c.IV (CDM81498)	
	D												OsATG18d.I (XP.015620970)		TaATG18d (AGW81809)	AtATG18d (AT3G56440)
	E												OsATG18eII (XP.015639564)			AtATG18e (AT5G05150)
Subfamily II	B	DsATG18 (Dusal.0460s00003)	VcATG18 (Vocar.0020s0155)	OtATG18 (Ot06g00720)	OlATG18 (OlATG18.41442.fragment)	MpuATg18 (MpuATG18.156491. fragment)	CsubATG18 (CsATG18.3880.fragment)	CrATG18 (Cre10.g457550)		KnATG18 (kfl00404.0130)	MpoATG18b (MARPO.0027s0044)	PpATG18 (Phpat.007G038400)	OsATG18b (XP.015627655)	ZmATG18b.I (NP_00114563.1)	TaATG18b (Traes.6AL.DDF2EBF31)	AtATG18b (AT4G30510)
														ZmATG18b.II (XP.020408852)	TaATG18e.I (Traes_6BL_B2A8BBB52)	
														ZmATG18b.III (Zm00001d018355)	TaATG18e.II (Traes.6DL.9F29527A0)	
Subfamily III	F						CsubATG18 (CsATG18.63899)		CbATG18 (CHBRA141g00400)	KnATG18 (kfl00046.0070)	MpoATG18f (MARPO.0006s0048)	PpATG18 (Phpat.008G022700)	OsATG18f.I (XP.015621123)	ZmATG18f.I (ONM37261)	TaATG18f.I (Traes.3B.F4F2FC6FA)	AtATG18f (AT5G54730)
												PpATG18 (Phpat.020G070000)	OsATG18f.II (XP.025877429)	ZmATG18f.II (ONM37262)	TaATG18f.II (Traes.3DL.E400E521A)	
												PpATG18 (Phpat.023G024100)	OsATG18f.III (LOC.Os05g33610)	ZmATG18f.III (Zm00001d043239)	TaATG18f.III (TraesCS3D02G318200)	
												PpATG18 (Phpat.024G018700)		ZmATG18f.IV (ONM37265)	TaATG18f.IV (CDM84501)	
														ZmATG18f.V (PWZ31673)	TaATG18f.V (Traes.3B.7A23DFB41)	
															TaATG18f.VI (Traes.3AL.B27F0D4FF)	
	G													ZmATG18g.I (AQK85845)		AtATG18g (AT1G03380)
														ZmATG18g.II (AQK85860)		
														ZmATG18g.III (AQK93836)		
														ZmATG18g.IV (AQK93828)		
														ZmATG18g.V (AQK93834)		
														ZmATG18g.VI (AQK85849)		
														ZmATG18g.VII (GRMZM2G078468)		
														ZmATG18g.VIII (PWZ17532)		
														ZmATG18g.IX (AQK93830)		
														ZmATG18g.X (AQK93829)		
														ZmATG18g.XI (AQK93835)		
														ZmATG18g.XII (AQK85856)		
														ZmATG18g.XIII (AQK93827)		
	H													ZmATG18h.I (XP.008649626)	TaATG18h.I (Traes.1BL.45E2558BB.1)	AtATG18h (AT1G54710)
													OsATG18h (XP.015639663)	ZmATG18h.II (PWZ11786)	TaATG18h.II (TraesCS1A02G254200.1)	
														ZmATG18h.III (XP.008656294)	TaATG18h.III (Traes.1DL.DB75BFD8A.1)	
															TaATG18h.IV (Traes.1AL.C4A651390.1)	

**Table 3:** List of *ATG18* homologs in legumes.

		*Genestoids*	*Dalbergioids*	*Milletioids*					*Robinioids*	*IRLC*				
		*Lupinus angustifolius*	*Arachis duranensis*	*Arachis ipaensis*	*Glycine max*	*Vigna angularis*	*Vigna radiata*	*Phaseolus vulgaris*	*Lotus Japonica*	*Cicer arietinum*	*Cajanus* *cajan*	*Medicago truncatula*	*Pisum sativum*	*Trifolium pratense*
Subfamily I	A	LaATG18a.I (XP.019421581.1)	AdATG18a.I (XP.015939789.1)	AiATG18a (XP.016174738.1)	GmATG18a.I (Glyma.10G152500.1)	VaATG18a.I (VIGAN03G286700)	VrATG18a.I (VRADI08G12430)	PvATG18a (Phvul.001G205000.1)		CaATG18a.I (XP.004495714.1)	CcATG18a.I (XP.020209984.1)	MtATG18a (Medtr1G083230.1)	PsATG18a (PSAT0S3233G0120.1)	TpATG18a.I (TRIPR.GENE96259)
		LaATG18a.II (XP.019452261.1)	AdATG18a.II (XP.015967701.1)		GmATG18a.II (Glyma.20G235800.1)	VaATG18a.II (XP.017412432.1)	VrATG18a.II (VRADI03G05850)			CaATG18a.II (XP.004494924.1)	CcATG18a.II (C.CAJAN.10296.1)			TpATG18a.II (TRIPR.GENE33973)
		LaATG18a.III (XP.019419463.1)			GmATG18a.III (Glyma.03G212100.1)	VaATG18a.III (VANG04G16030.1)				CaATG18a.III (C.CA.05407.1)	CcATG18a.III (XP.020212010.1)			TpATG18a.III (PNX79795.1)
		LaATG18a.IV (XP.019441771.1)			GmATG18a.IV (Glyma.19G209200.1)	VaATG18a.IV (VANG06G12920.1)								
		LaATG18a.V (XP.019441170.1)												
		LaATG18b.III (TanjilG.02747)							LjATG18b.II (Lj5g3v1496760.1)	CaATG18b.V (Ca.04089)				
									LjATG18b.III (Lj0g3v0083309.1)	CaATG18b.VI (CC4958C.Ca14068.1)				
									LjATG18b.IV (Lj1g3v4912170.1)					
	C	LaATG18c.I (XP.019430950.1)	AdATG18c (XP.015945005.1)	AiATG18c (XP.016181861.1)	GmATG18c.I (Glyma.04G224300.1)			PvATG18c.I (Phvul.009G041700.1)		CaATG18c (C.CA.03673)			PsATG18c (PSAT5G069920.1)	TpATG18c.I (TRIPR.GENE13965)
		LaATG18c.II (XP.019417508.1)			GmATG18c.II (Glyma.06G140400.1)			PvATG18c.II (Phvul.007G196400.1)				MtATG18c (Medtr7G108520.1)		TpATG18c.II (PNX92525.1)
		LaATG18c.III (LUP000470)							LjATG18c (Lj1G3V1112870.1)					
	D	LaATG18d (XP.019430946.1)				VaATG18d.I (VIGAN04G120000)	VrATG18d.I (VRADI0239S00050)			CaATG18d (XP.004502800.1)	CcATG18d.I (XP.029129536.1)	MtATG18d (Medtr1G088855.1)		
						VaATG18d.II (VANG0200S00330.1)	VrATG18d.II (XP.022632145.1)				CcATG18d.II (XP.020229011.1)			
	E						VrATG18d.VI (XP.022632144.1)					MtATG18e (Medtr3G093590.1)		
Subfamily II		LaATG18b.I (XP.019441874.1)	AdATG18b (XP.015933286.1)	AiATG18b (XP.016200540.1)	GmATG18b.I (Glyma.17G070200.1)	VaATG18b.I (VIGAN01G240600)	VrATG18b.I (VRADI07G21660)	PvATG18b (Phvul.003G152800.1)	LjATG18b.I (Lj4G3V2018270.1)	CaATG18b.I (XP.027192941.1)		MtATG18b (Medtr4G130190.1)	PsATG18b (PSAT0S2826G0080.1)	TpATG18b.I (PNX94509)
	B*	LaATG18b.II (XP.019441865.1			GmATG18b.II (Glyma.02G207500.2)	VaATG18b.II (XP.017411081.1)	VrATG18b.II (XP.014510099.1)			CaATG18b.II (XP.004507771.1)				TpATG18b.II (PNY02700.1)
					GmATG18b.III (Glyma.10G126200.1)	VaATG18b.III (XP.017411091.1)				CaATG18b.III (XP.027192940.1)				
						VaATG18b.IV (VANG11G12160.2)				CaATG18b.IV (ICC4958.CA.21790.1)				
						VaATG18b.V (XP.017411074.1)								
					GmATG18e (Glyma.16G109400.1)									
	*						VrATG18d.III (XP.014522590.1)							
		LaATG18f.I (XP.019437124.1)	AdATG18f.I (ARADU.XJ3JE.1)	AiATG18f.I (XP.016170472.1)	GmATG18f.I (Glyma.12G214600.1)	VaATG18f.I (VIGAN05G145500)	VrATG18f.I (XP.014522059.1)	PvATG18f.I (Phvul.011G140900.1)	LjATG18f (Lj3G3V1544540.1)	CaATG18f.I (XP.004487613.1)	CcATG18f.I (XP.020229318.1)	MtATG18f (Medtr2G082770.1)	PsATG18f (PSAT5G249880.1)	TpATG18f (TRIPR.GENE36798)
Subfamily III	F	LaATG18f.II (XP.019453655.1)	AdATG18f.II (XP.015936500.1)	AiATG18f.II (ARAIP.FRI7H.1)	GmATG18f.II (Glyma.12G136000.1)	VaATG18f.II (XP.017425518.1)	VrATG18f.II (XP.014494161.1)	PvATG18f.II (Phvul.005G091300.1)		CaATG18f.II (XP.027187641.1)	CcATG18f.II (XP.020229320.1)			
		LaATG18f.III (OIW15456.1)			GmATG18f.III (Glyma.13G287000.1)	VaATG18f.III (VIGAN08G077000)	VrATG18f.III (XP.022634400.1)			CaATG18f.III (XP.004487612.1)	CcATG18f.III (C.CAJAN32508.1)			
		LaATG18f.IV (XP.019453653.1)			GmATG18f.IV. (Glyma.06G267000.1)	VaATG18f.IV (VANG1095S00020.1)	VrATG18f.IV (VRADI02G09460.1)			CaATG18f.IV (CA.00864.1)	CcATG18f.IV (XP.020235274.1)			
											CcATG18f.V (XP.020229319.1)			
											CcATG18f.VI (XP.020229316.1)			
		LaATG18g.I (XP.019441802.1)	AdATG18g (XP.015951046.1)	AiATG18g (XP.016184366.1)	GmATG18g.I (Glyma.03G148700.1)	VaATG18g.I (XP.017419622.1)	VrATG18g (VRADI03G00450)	PvATG18g.I (Phvul.001G146700.1)	LjATG18g (Lj1G3V4404380.1)	CaATG18g.I (CA.09934.1)	CcATG18g.I (XP.020211839.1)	MtATG18g.I (Medtr1G089110.1)	PsATG18g (PSAT6G169560.1)	TpATG18g.I (TRIPR.GENE16922)
	G	LaATG18g.II (XP.019441803.1)			GmATG18g.II (Glyma.19G152000.1)	VaATG18g.II (KOM38883.1)		PvATG18g.II (Phvul.007G183100.1)		CaATG18g.III (CA.08309)	CcATG18g.II (C.CAJAN09614.1)			TpATG18g.II (TRIPR.GENE2713)
					GmATG18g.III (Glyma.20G230900.1)	VaATG18g.III (VIGAN.VANG07G05180.1)					CcATG18g.III (KYP70659.1)			
		LaATG18h.I (XP.019421306.1)	AdATG18h.I (XP.015939933.1)	AiATG18h.I (XP.016205481.1)	GmATG18h (Glyma.10G157700.1)	VaATG18h.I (KOM55039.1)	VrATG18h (VRADI08G12840.1)		LjATG18h (Lj5G3V1451080.1)	CaATG18h.I (XP.027189075.1)	CcATG18h.I (XP.020233978.1)	MtATG18h (Medtr1G082300.1)	PsATG18h (PSAT6G148560.1)	TpATG18h.I (PNY09258.1)
	H	LaATG18h.II (XP.019421307.1)	AdATG18h.II (XP.015939934.1)	AiATG18h.II (XP.016176031.1)		VaATG18h.II (VANG06G10190.1)				CaATG18h.II (XP.027189076.1)	CcATG18h.II (C.CAJAN06885.1)			TpATG18h.II (PNY17060.1)
		LaATG18h.III (XP.019421305.1)	AdATG18h.III (XP.015968551.1)	AiATG18h.III (XP.016176030.1)						CaATG18h.III (CA.09238.1)	CcATG18h.III (XP.020233954.1)			TpATG18h.III (PNY12850.1)
		LaATG18h.IV (XP.019452235.1)		AiATG18h.IV (XP.016176032.1)							CcATG18h.IV (XP.029125824.1)			
		LaATG18h.V (TANJILG.10103)												
		LaATG18h.VI (OIW07130.1)												
		LaATG18h.VII (XP.019452236.1)												
		LaATG18h.VIII (XP.019452234.1) LaATG18h.IX (OIW12695.1)												

* Sequence ID with assigned the letter but belongs to other ATG18 Subfamily.

## Data Availability

The data reported in this study are available in the supplementary information provided in the supplementary data.

## Supplementary Materials

The following are available online at https://www.mdpi.com/article/10.3390/plants10122619/s1, Figure S1: Percentage of legume ATG homologs in different software programs. Figure S2: Validation of expression patterns of ATGs of symbiont-colonized P. vulgaris roots by RT-qPCR analysis. Figure S3A: Representation of 280 ATG18 proteins from different plant species analyzed by multidimensional scaling using Bios2mds. Figure S3B: Phylogenetic tree of ATG18 in plants. Figure S4: Secondary structure prediction using JPred. Figure S5: Microsynteny analysis of ATG18 (Subfamily I & II) in *P. vulgaris.* Figure S6: Microsynteny analysis of ATG18 (Subfamily III) in *P. vulgaris.* Figure S7: Phosphorylation sites of ATG18 in *P. vulgaris* identified using NetPhos. Figure S8: Prediction of transmembrane helices in PvATG18 proteins using TMHMM. Table S1: List of identifiers of the genes, transcripts, and proteins of each ATG in *P. vulgaris*, Table S2: ATG18 protein characterization in *P. vulgaris*. Table S3: List of Oligos for RT-Qpcr. Supplementary information: Supplementary Information SI1: Analysis of ATG genes homologs in *P. vulgaris, M. truncatula, G. max* in different databases; Supplementary Information SI2: Expression profiles of ATGs in *P. vulgaris*; Supplementary Information SI3: Analysis of ATG18 homologs; Supplementary Information SI4: Family, repeats, motifs and domain positions in legumes; Supplementary Information SI5: Alignment and synteny of ATG genes between *A. thaliana* and legumes using the comparative genomics in Ensembl.
