# Supplementary material for: Exploration of Autophagy Families in Legumes and Dissection of the ATG18 Family with a Special Focus on Phaseolus vulgaris
Source: Plants (Basel). 2021 Nov 29;10(12):2619. doi: 10.3390/plants10122619 (PMC8703869; doi:10.3390/plants10122619)
Supplement: Supplementary file 1 [file plants-10-02619-s001.zip › Supplementary figures revised.pptx]

## Slide 1
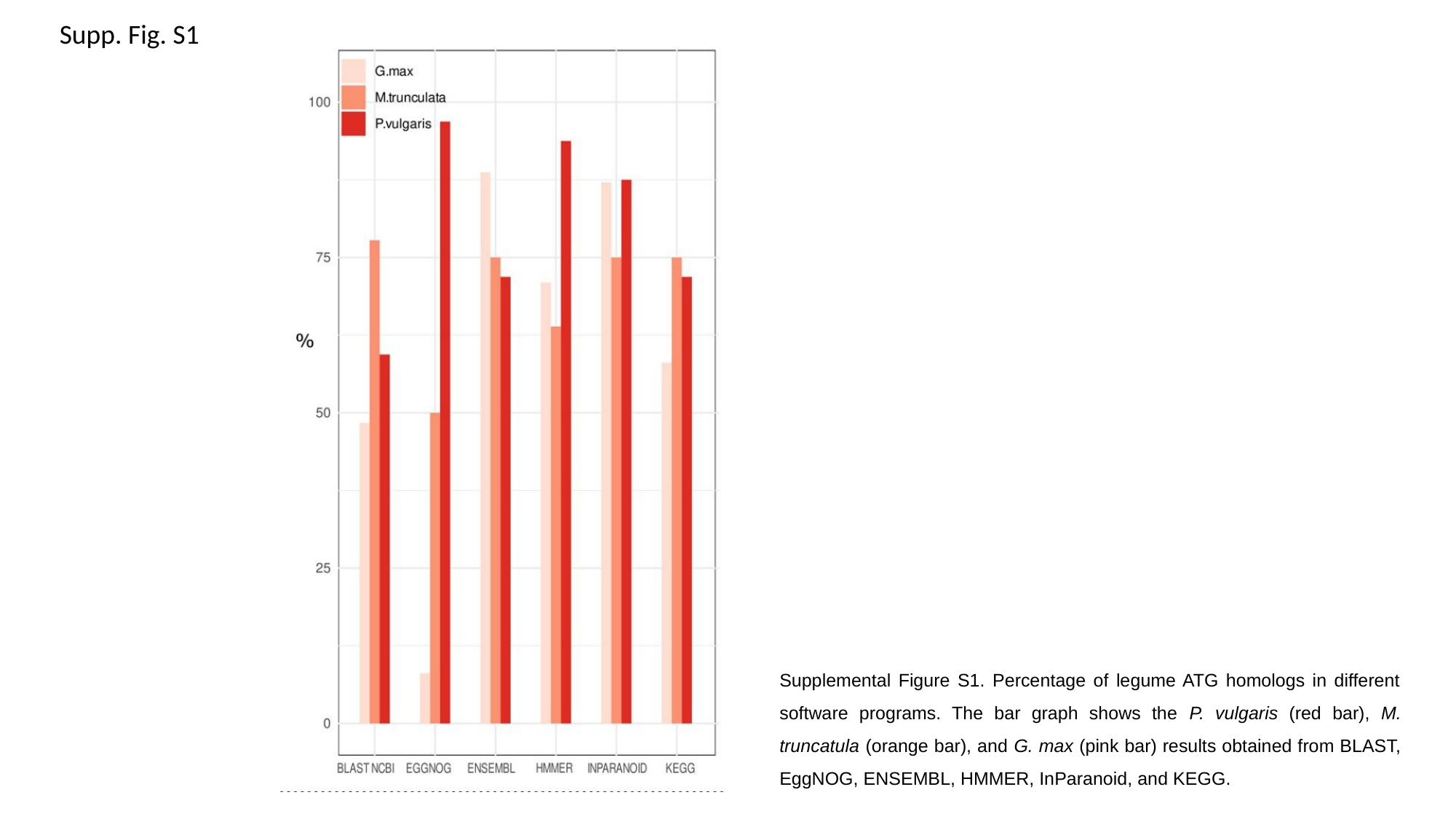

Supp. Fig. S1
Supplemental Figure S1. Percentage of legume ATG homologs in different software programs. The bar graph shows the P. vulgaris (red bar), M. truncatula (orange bar), and G. max (pink bar) results obtained from BLAST, EggNOG, ENSEMBL, HMMER, InParanoid, and KEGG.

## Slide 2
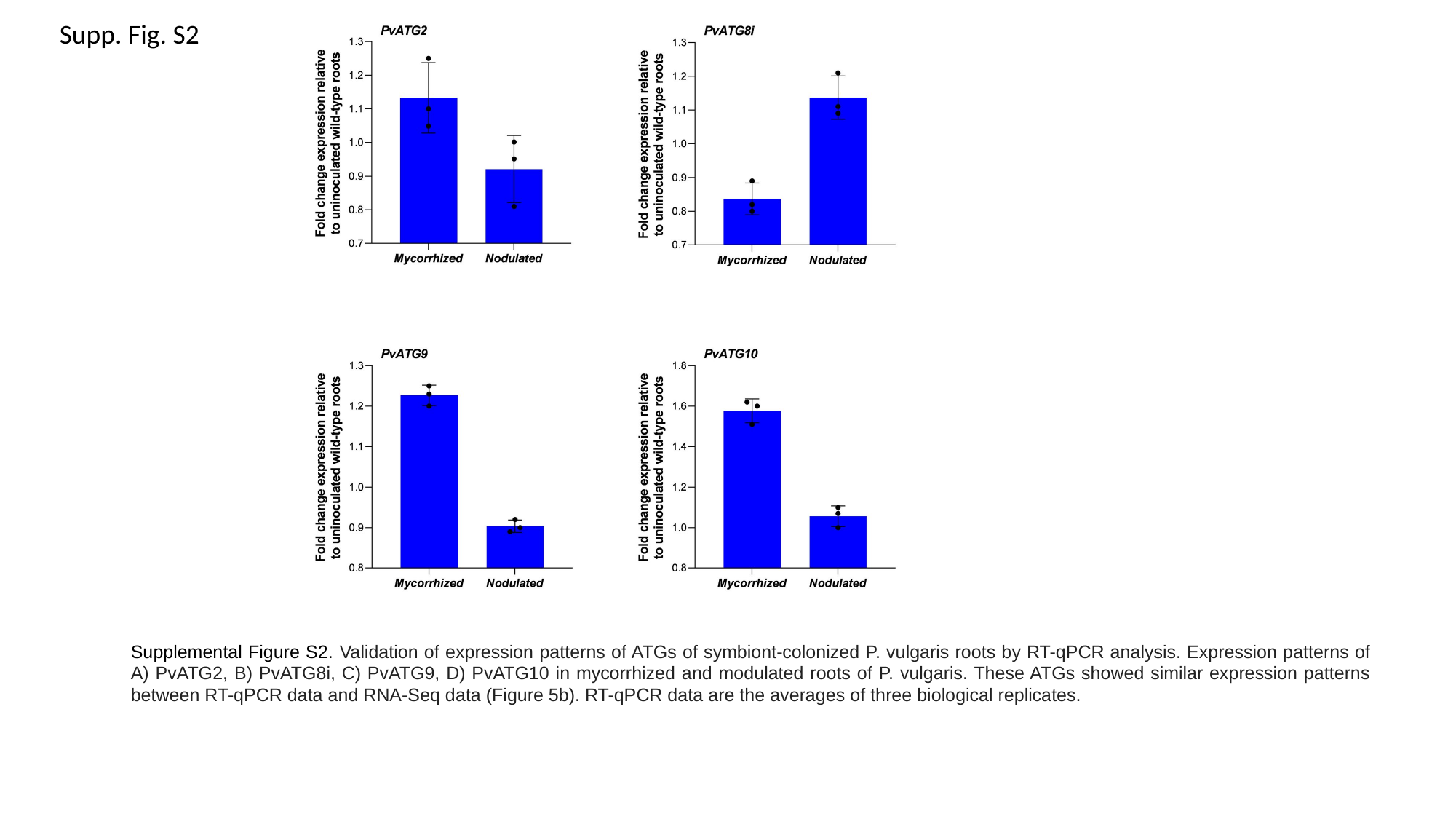

Supp. Fig. S2
Supplemental Figure S2. Validation of expression patterns of ATGs of symbiont-colonized P. vulgaris roots by RT-qPCR analysis. Expression patterns of A) PvATG2, B) PvATG8i, C) PvATG9, D) PvATG10 in mycorrhized and modulated roots of P. vulgaris. These ATGs showed similar expression patterns between RT-qPCR data and RNA-Seq data (Figure 5b). RT-qPCR data are the averages of three biological replicates.

## Slide 3
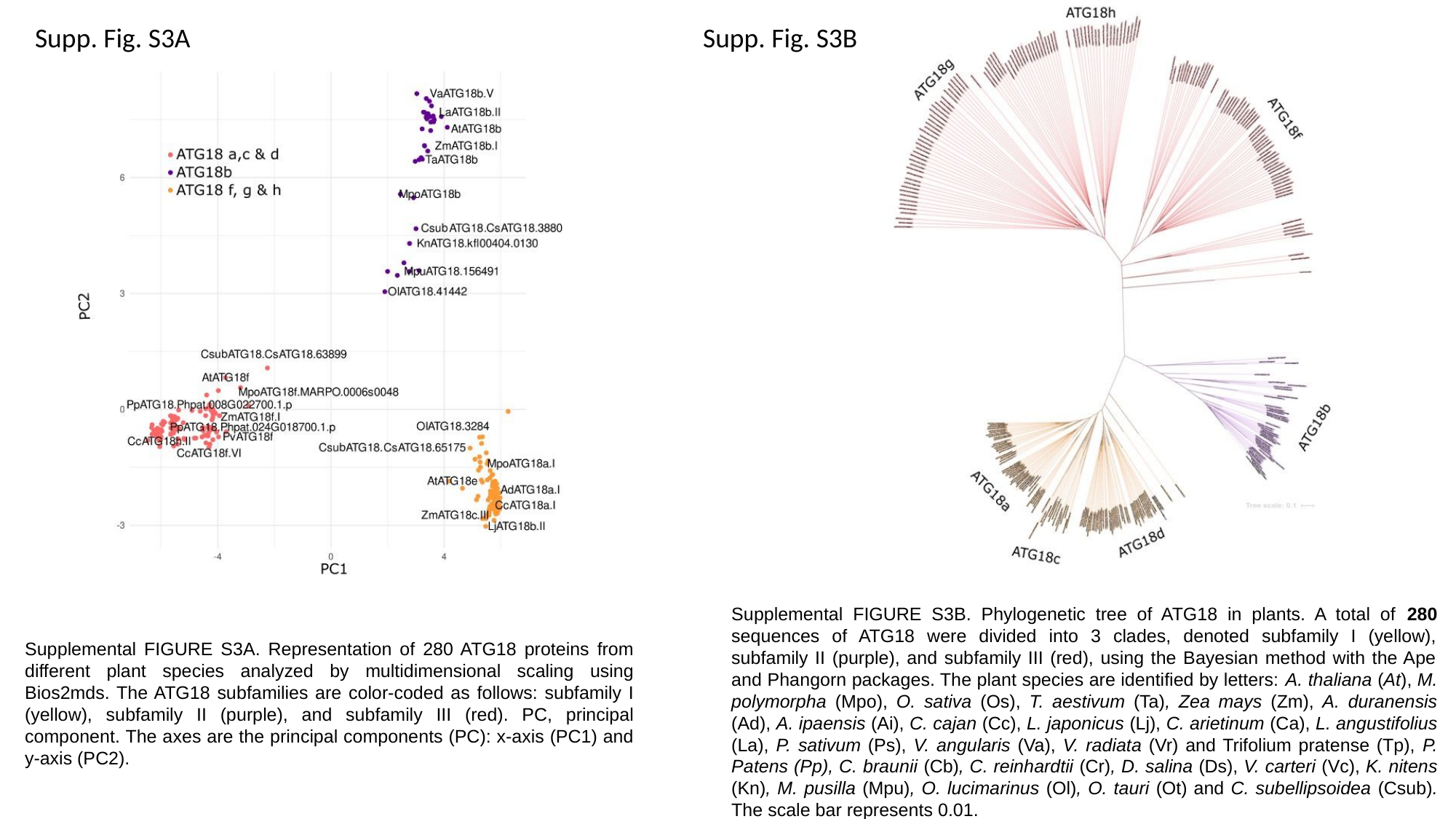

Supp. Fig. S3A
Supp. Fig. S3B
Supplemental FIGURE S3B. Phylogenetic tree of ATG18 in plants. A total of 280 sequences of ATG18 were divided into 3 clades, denoted subfamily I (yellow), subfamily II (purple), and subfamily III (red), using the Bayesian method with the Ape and Phangorn packages. The plant species are identified by letters: A. thaliana (At), M. polymorpha (Mpo), O. sativa (Os), T. aestivum (Ta), Zea mays (Zm), A. duranensis (Ad), A. ipaensis (Ai), C. cajan (Cc), L. japonicus (Lj), C. arietinum (Ca), L. angustifolius (La), P. sativum (Ps), V. angularis (Va), V. radiata (Vr) and Trifolium pratense (Tp), P. Patens (Pp), C. braunii (Cb), C. reinhardtii (Cr), D. salina (Ds), V. carteri (Vc), K. nitens (Kn), M. pusilla (Mpu), O. lucimarinus (Ol), O. tauri (Ot) and C. subellipsoidea (Csub). The scale bar represents 0.01.
Supplemental FIGURE S3A. Representation of 280 ATG18 proteins from different plant species analyzed by multidimensional scaling using Bios2mds. The ATG18 subfamilies are color-coded as follows: subfamily I (yellow), subfamily II (purple), and subfamily III (red). PC, principal component. The axes are the principal components (PC): x-axis (PC1) and y-axis (PC2).

## Slide 4
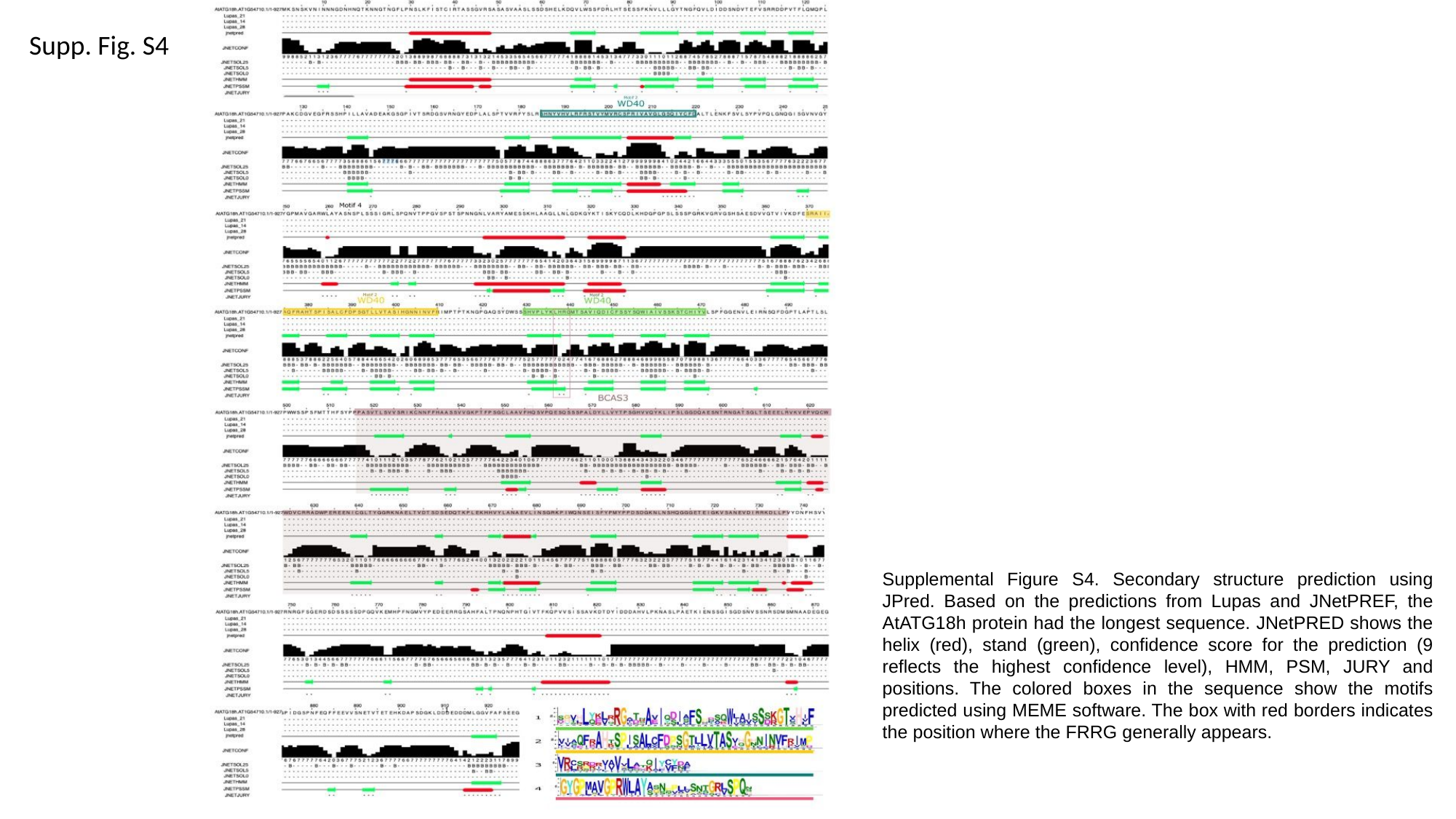

Supp. Fig. S4
Supplemental Figure S4. Secondary structure prediction using JPred. Based on the predictions from Lupas and JNetPREF, the AtATG18h protein had the longest sequence. JNetPRED shows the helix (red), stand (green), confidence score for the prediction (9 reflects the highest confidence level), HMM, PSM, JURY and positions. The colored boxes in the sequence show the motifs predicted using MEME software. The box with red borders indicates the position where the FRRG generally appears.

## Slide 5
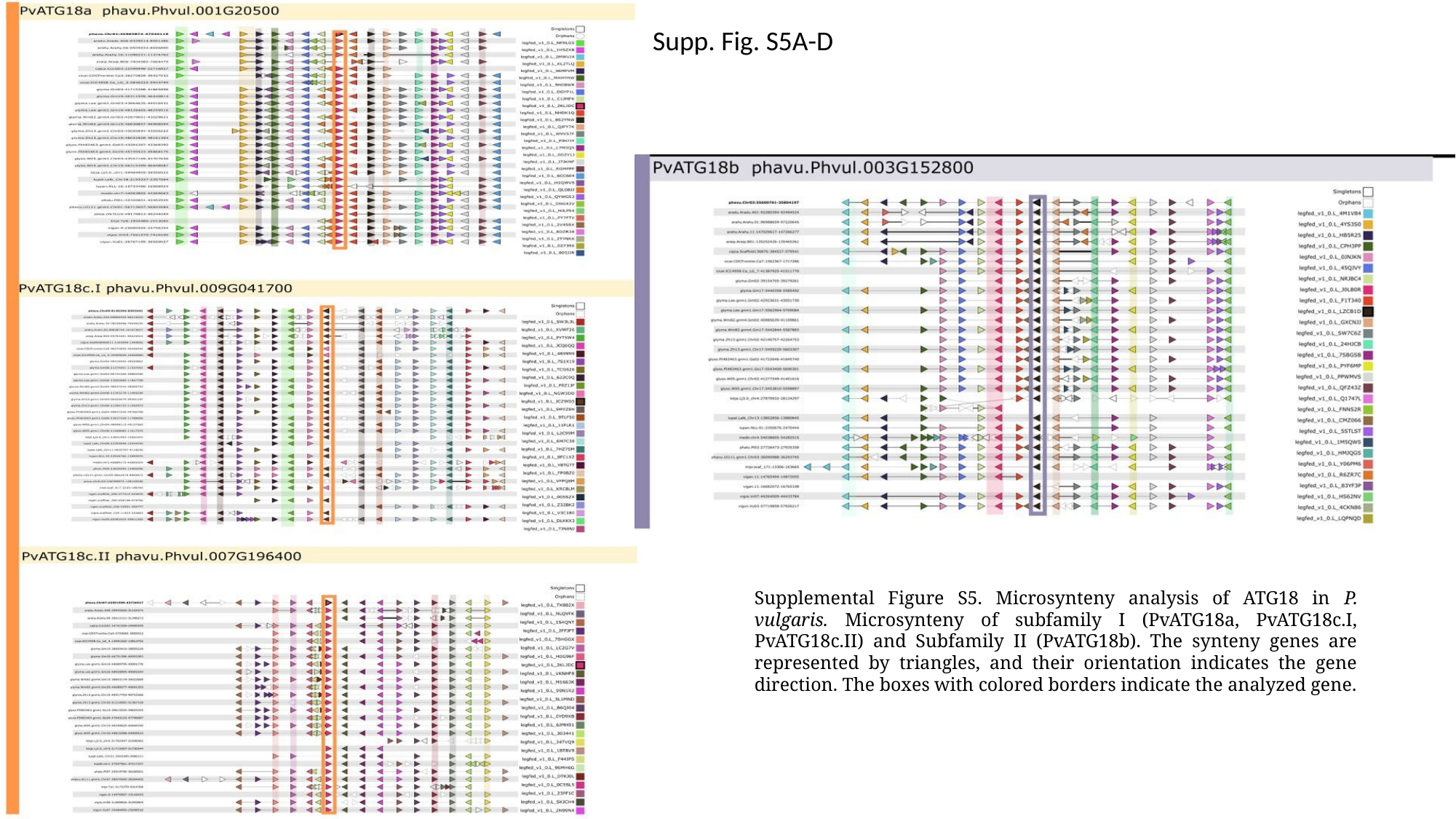

Supp. Fig. S5A-D
Supplemental Figure S5. Microsynteny analysis of ATG18 in P. vulgaris. Microsynteny of subfamily I (PvATG18a, PvATG18c.I, PvATG18c.II) and Subfamily II (PvATG18b). The synteny genes are represented by triangles, and their orientation indicates the gene direction. The boxes with colored borders indicate the analyzed gene.

## Slide 6
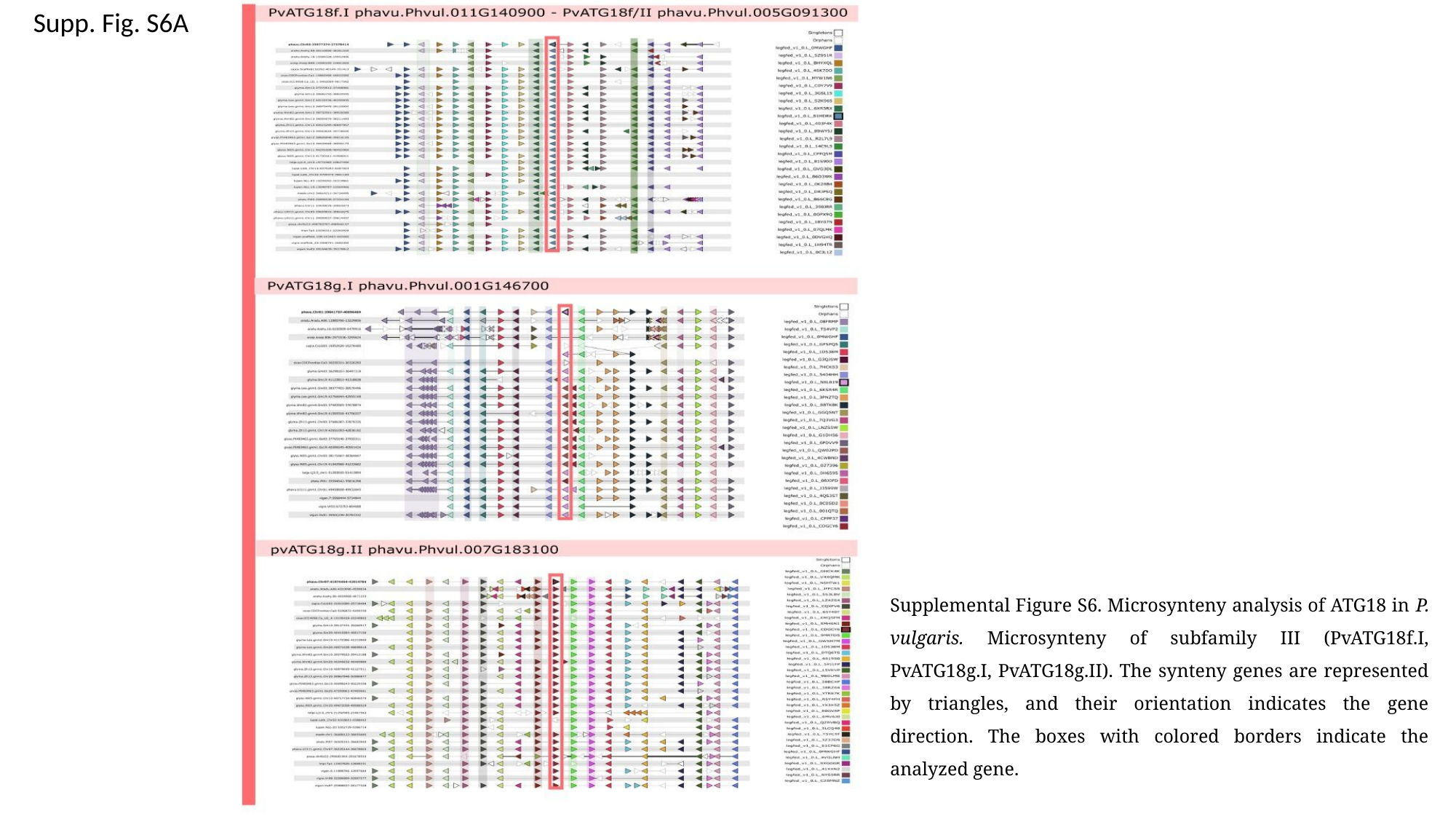

Supp. Fig. S6A
Supplemental Figure S6. Microsynteny analysis of ATG18 in P. vulgaris. Microsynteny of subfamily III (PvATG18f.I, PvATG18g.I, PvATG18g.II). The synteny genes are represented by triangles, and their orientation indicates the gene direction. The boxes with colored borders indicate the analyzed gene.

## Slide 7
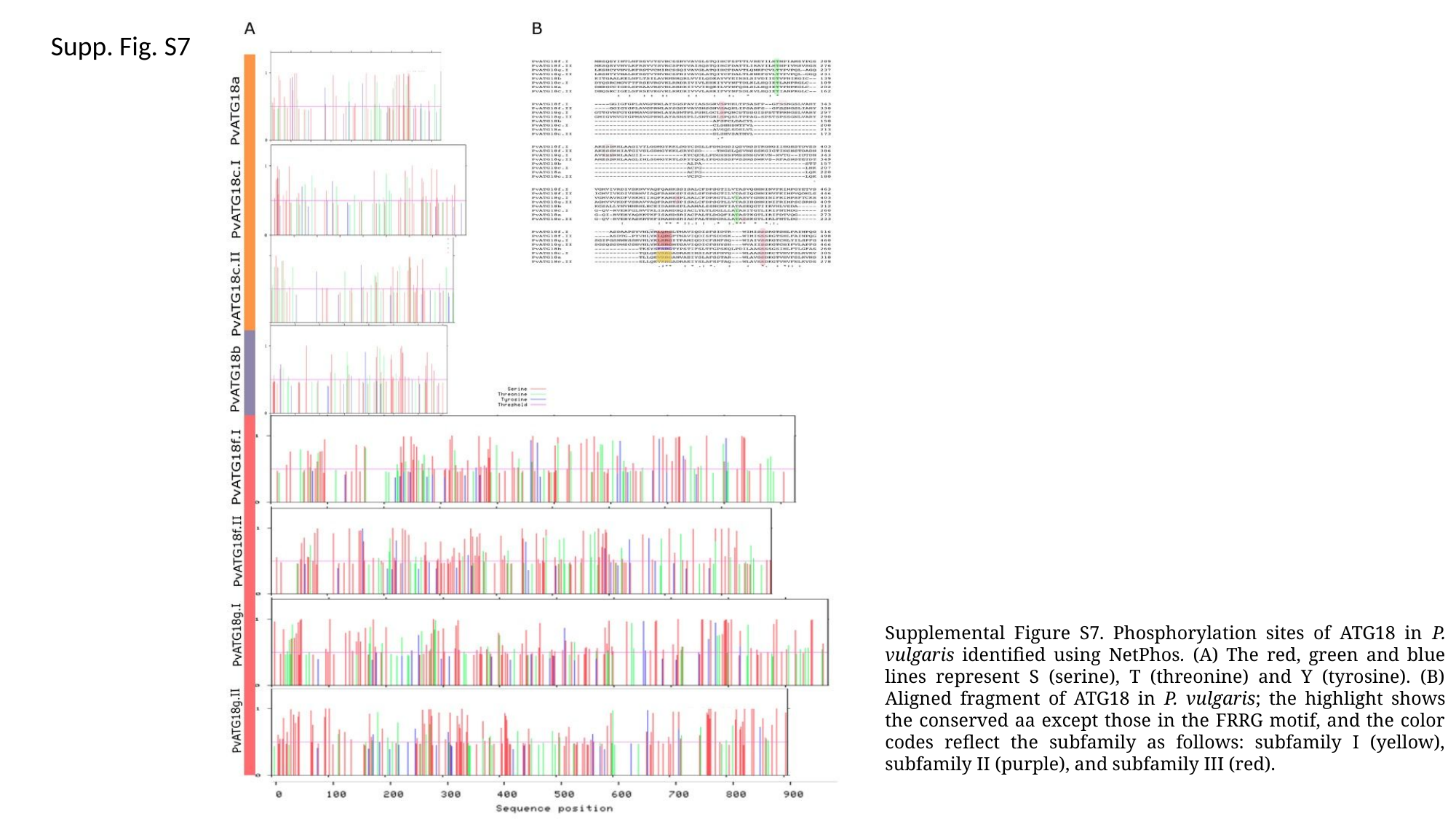

Supp. Fig. S7
Supplemental Figure S7. Phosphorylation sites of ATG18 in P. vulgaris identified using NetPhos. (A) The red, green and blue lines represent S (serine), T (threonine) and Y (tyrosine). (B) Aligned fragment of ATG18 in P. vulgaris; the highlight shows the conserved aa except those in the FRRG motif, and the color codes reflect the subfamily as follows: subfamily I (yellow), subfamily II (purple), and subfamily III (red).

## Slide 8
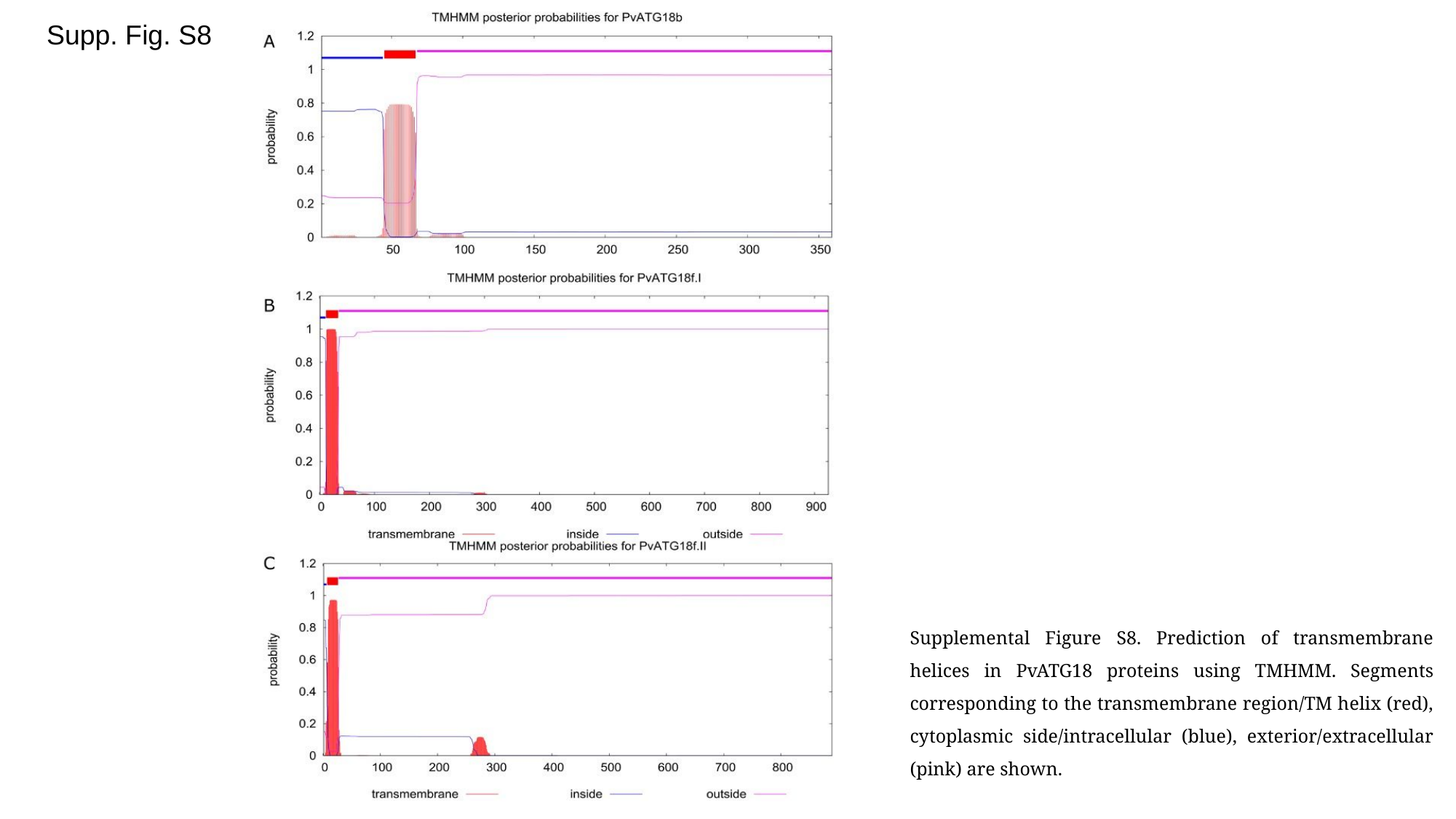

Supp. Fig. S8
Supplemental Figure S8. Prediction of transmembrane helices in PvATG18 proteins using TMHMM. Segments corresponding to the transmembrane region/TM helix (red), cytoplasmic side/intracellular (blue), exterior/extracellular (pink) are shown.
