# Supplementary material for: Exploration of Autophagy Families in Legumes and Dissection of the ATG18 Family with a Special Focus on Phaseolus vulgaris
Source: Plants (Basel). 2021 Nov 29;10(12):2619. doi: 10.3390/plants10122619 (PMC8703869; doi:10.3390/plants10122619)
Supplement: Supplementary file 1 [file plants-10-02619-s001.zip › Supplementary information/Supp. Info. SI1.pdf]

Supp. Info.SI1. Analysis of ATG genes homologs in *P. vulgaris, M. truncatula, G. max* in different databases. (A) Orthologous and paralogous, (B) Basic Local Alignment Search Tool -BLAST (C) KEGG (D)ENSEMBL (E)HMMER (F) INPARANOID (G) EGGNOG (H)PANTHER.

A. Orthologous and paralogous

Orthologs		Paralogs		Orthologs		Paralogs		Orthologs		Paralogs	
PvATG1t	Phvul.010g120500	**PvATG1b**	Phvul.010g015100	**MtrATG1b**	Medtr8g024100	**MtrATG1t**	Medtr3g095620	**GmATG1c.II**	Glyma.01g099600	**GmATG1a.I**	Glyma.07g048400
PvATG2	Phvul.003g295800	**PvATG5**	Phvul.008g241000	**MtrATG1c**	Medtr4g019410	**MtrATG6**	Medtr3g018770	**GmATG7**	Glyma.12g010000	**GmATG1a.II**	Glyma.16g017300
PvATG3	Phvul.011g006500	**PvATG8c.I**	Phvul.003g079300	**MtrATG2**	Medtr4g086370	**MtrATG8c**	Medtr4g048510	**GmATG8b**	glyma.15g188600	**GmATG1b.I**	Glyma.03g069800
PvATG4a	Phvul.008g048900	**PvATG9a**	Phvul.001g159900	**MtrATG3**	Medtr4g036265	**MtrATG8f**	Medtr1g086310	**GmATG8c.III**	Ggyma.09g003900	**GmATG1b.II**	Glyma.01g099600
PvATG6	Phvul.005g029900	**PvATG9b**	Phvul.007g194300	**MtrATG4a**	Medtr7g081230	**MtrATG8g**	Medtr4g123760	**GmATG8c.V**	glyma.07g261000	**GmATG1t.I**	Glyma.06g150700
PvATG7	Phvul.011g010700	**PvATG18a**	Phvul.001g205000	**MtrATG5**	Medtr5g076920	**MtrATG9a**	Medtr7g096680	**GmATG8c.VI**	glyma.15g108200	**GmATG1t.II**	Glyma.04g215500
PvATG8d	Phvul.011g103300	**PvATG18c.II**	Phvul.007g196400	**MtrATG7**	Medtr0003s0540	**MtrATG9b**	Medtr1g070160	**GmATG8f**	glyma.17g140700	**GmATG2.I**	Glyma.02g133400
PvATG8f.I	Phvul.003g219600	**PvATG18f.I**	Phvul.011g140900	**MtrATG8e**	Medtr4g101090	**MtrATG11**	Medtr4g130370	**GmATG12b.I**	glyma.07g038100	**GmATG2.II**	Glyma.07g211600
PvATG8f.II	Phvul.002g062200	**PvATG18f.II**	Phvul.005g091300	**MtrATG10**	Medtr8g010140	**MtrATG13b.II**	Medtr3g095570	**GmATG12b.II**	glyma.16g007300	**GmATG3.I**	Glyma.12g005700
PvATG8i	Phvul.007g210800	**PvATG18g.II**	Phvul.007g183100	**MtrATG101**	Medtr8g079240	**MtrATG13c**	Medtr8g093050	**GmATG18a.II**	glyma.10g152500	**GmATG3.II**	Glyma.09g231000
PvATG10	Phvul.010g036300			**MtrATG12b**	Medtr8g020500	**MtrATG18a**	Medtr1g083230	**GmATG18a.II**	glyma.20g23500	**GmATG4a.I**	Glyma.18g248400
PvATG11	Phvul.003g153800			**MtrATG13a**	Medtr5g068710	**MtrATG18b**	Medtr4g0130190	**GmATG18c.I**	glyma.04g224300	**GmATG4a.II**	Glyma.09g244800
PvATG12b	Phvul.010g130300			**MtrATG14a**	Medtr5g061040	**MtrATG18c**	Medtr7g108520	**GmATG18c.III**	glyma.07g203900	**GmATG5.I**	Glyma.14g210200
PvATG13	Phvul.008g187800			**MtrATG16.II**	Medtr3g075400	**MtrATG18c.I**	Medtr1g088855	**GmATG18f.I**	glyma.12g214600	**GmATG5.II**	Glyma.02g240700
PvATG13b	Phvul.002g269600			**MtrATG16b**	Medtr4g104380	**MtrATG18e**	Medtr3g093590	**GmATG101**	glyma.17g180900	**GmATG6.I**	Glyma.11g153900
PvATG14	Phvul.008g169200			**MtrATG18f**	Medtr2g082770	**MtrATG18e**	Medtr3g093590			**GmATG6.II**	Glyma.04g141000
PvATG16	Phvul.003g207100			**MtrATG18g**	Medtr1g089110	**MtrATG18g**	Medtr1g082300			**GmATG8c.I**	Glyma.12g098400
PvATG18b	Phvul.003g152800									**GmATG8c.II**	Glyma.06g306300
PvATG18c.I	Phvul.009g041700									**GmATG8c.II**	Glyma.06g306300
PvATG18g.I	Phvul.001g146700									**GmATG8c.III**	Glyma.09g003900
PvATG101	Phvul.003g248000									**GmATG8c.III**	Glyma.09g003900

B. Basic Local Alignment Search Tool (BLAST) of *P. vulgaris, M. truncatula, G.max* based in *A. thaliana.*

Phaseolus vulgaris

Arabidopsis Protein accession numbers	***Phaseolus vulgaris Protein accession numbers***	**Query Cover**	**Per. Ident**
At1g49180.1	Phvul.010G120500	74	39.22
At1g49180.2	Phvul.010G120500	81	38.96
At2g37840.1	Phvul.010G015100	86	57.32

At2g37840.2	Phvul.010G015100	93	53.38
At2g37840.3	Phvul.010G015100	95	48.13
At3g53930.1	Phvul.010G015100	86	55.3
At3g53930.2	Phvul.010G015100	86	55.45
At3g53930.3	Phvul.010G015100	98	50
At3g53930.4	Phvul.010G015100	98	50.18
At3g53930.5	Phvul.010G015100	98	50
At3g61960.1	Phvul.010G120500	99	48.16
At3g61960.2	Phvul.010G120500	99	45.55
At3g19190.1	Phvul.003G295800	99	44.68
At3g19190.2	Phvul.003G295800	99	46.62
At3g19190.3	Phvul.003G295800	99	46.62
At5g61500.1	Phvul.011G006500	99	85.67
At5g61500.2	Phvul.011G006500	81	77.82
At2g44140.1	Phvul.008G048900	99	58.61
At2g44140.2	Phvul.008G048900	95	61.43
At2g44140.3	Phvul.008G048900	99	58.61
At2g44140.4	Phvul.008G048900	99	62.44
At2g44140.5	Phvul.008G048900	99	59.14
At3g59950.1	Phvul.008G048900	100	55.98
At3g59950.2	Phvul.008G048900	91	54.85
At3g59950.3	Phvul.008G048900	91	65.45
At3g59950.4	Phvul.008G048900	99	62.41
At3g59950.5	Phvul.008G048900	99	57.61
At5g17290.1	Phvul.008G241000	97	62.46
At3g61710.1	Phvul.005G029900	94	74.9
At3g61710.2	Phvul.005G029900	94	71.7
At3g61710.3	Phvul.005G029900	92	73.99
At3g61710.4	Phvul.005G029900	93	73.23
At5g45900.1	Phvul.011G010700	96	70.18
At4g21980.1	Phvul.011G103300	95	84.72
At4g21980.2	Phvul.011G103300	84	84.82
At4g04620.1	Phvul.003G079300	95	80.51
At4g04620.2	Phvul.003G079300	95	80.51
At4g04620.3	Phvul.003G079300	95	80.51
At1g62040.1	Phvul.011G103300	99	90.76
At1g62040.2	Phvul.011G103300	88	90.75
At2g05630.1	Phvul.011G103300	99	91.67
At2g05630.2	Phvul.011G103300	66	91.74
At2g45170.1	Phvul.003G219600	95	81.36
At2g45170.2	Phvul.003G219600	95	81.36

At4g16520.1	Phvul.003G219600	96	91.38
At4g16520.1	Phvul.002G062200	96	91.38
At4g16520.2	Phvul.003G219600	95	91.38
At4g16520.3	Phvul.003G219600	95	91.38
At3g60640.1	Phvul.003G219600	91	86.61
At3g06420.1	Phvul.007G210800	94	68.14
At3g15580.1	Phvul.007G210800	98	71.68
At2g31260.1	Phvul.001G159900	99	65.2
At2g31260.1	Phvul.007G194300	97	59.77
At3g07525.1	Phvul010G036300	96	52.73
At3g07525.2	Phvul.010G036300	96	52.49
At4g30790.1	Phvul.003G153800	99	60.79
At1g54210.1	Phvul.010G130300	94	89.13
At1g54210.2	Phvul.010G130300	90	47.83
At1g54210.3	Phvul.010G130300	94	89.13
At3g13970.1	Phvul.010G130300	100	82.98
At3g13970.2	Phvul.010G130300	83	71.43
At3g13970.3	Phvul.010G130300	83	71.43
At3g13970.4	Phvul.010G130300	85	78.67
At3g49590.1	Phvul.008G187800	98	49.11
At3g49590.2	Phvul.008G187800	98	49.11
At3g49590.3	Phvul.008G187800	99	47.69
At3g18770.1	Phvul.002G269600	96	54.19
AT1G77890.1	Phvul.008G169200	96	51.21
AT1G77890.2	Phvul.008G169200	96	49.67
AT1G77890.3	Phvul.008G169200	96	51.21
AT4G08540.1	Phvul.008G169200	99	71.49
At5g50230.1	Phvul.003G207100	99	72.98
At3g62770.1	Phvul.007G196400	88	74.41
At3g62770.1	Phvul.001G205000	98	66.36
At3g62770.3	Phvul.007G196400	86	74.79
At4g30510.1	Phvul.003G152800	97	68.95
At4g30510.2	Phvul.003G152800	99	72.06
At2g40810.1	Phvul.009G041700	98	69.82
At2g40810.2	Phvul.009G041700	98	69.82
At2g40810.3	Phvul.009G041700	98	67.24
At3g56440.1	Phvul.009G041700	97	68.11
At3g56440.2	Phvul.009G041700	98	68.12
At3g56440.3	Phvul.009G041700	95	69.72
At5g05150.1	Phvul.009G041700	97	48.05
At5g54730.1	Phvul.005G091300	88	42.8

At5g54730.2	Phvul.011G140900	89	40.66
At1g03380.1	Phvul.001G146700	86	57.35
At1g54710.1	Phvul.007G183100	98	55.02
At1g54710.2	Phvul.007G183100	100	53.1
At5g66930.1	Phvul.003G248000	87	80.58
At5g66930.2	Phvul.003G248000	100	75.8
At5g66930.3	Phvul.003G248000	81	75

Medicago truncatula

Arabidopsis Protein accession numbers	*Medicago truncatula* Protein accession numbers	Query Cover	Per. Ident
At1g49180.1	MTR_3g095620	62	57.65
At1g49180.2	MTR_3g095620	67	57.65
At2g37840.1	MTR_4g019410	97	63.5
At2g37840.2	MTR_4g019410	93	58.51
At2g37840.3	MTR_4g019410	95	53.47
At3g53930.1	MTR_4g019410	98	59.89
At3g53930.2	MTR_4g019410	98	59.94
At3g53930.3	MTR_4g019410	98	53.79
At3g53930.4	MTR_4g019410	98	53.87
At3g53930.5	MTR_4g019410	98	53.79
At3g61960.1	MTR_8g024100	98	49.71
At3g61960.2	MTR_8g024100	98	46.76
At3g19190.1	MTR_4g086370	99	43.74
At3g19190.2	MTR_4g086370	98	46.76
At3g19190.3	MTR_4g086370	99	45.9
At5g61500.1	MTR_4g036265	99	84.98
At5g61500.2	MTR_4g036265	80	76.65
At2g44140.1	MTR_7g081230	99	58.85
At2g44140.2	MTR_7g081230	96	60.38
At2g44140.3	MTR_7g081230	99	58.85
At2g44140.4	MTR_7g081230	99	62
At2g44140.5	MTR_7g081230	99	59.54
At3g59950.1	MTR_7g081230	99	56.26
At3g59950.2	MTR_7g081230	90	55.05
At3g59950.3	MTR_7g081230	91	63.01
At3g59950.4	MTR_7g081230	99	61.75
At3g59950.5	MTR_7g081230	99	57.36
At5g17290.1	MTR_5g076920	99	59.44
At3g61710.1	MTR_3g018770	99	74.27

At3g61710.2	MTR_3g018770	94	73.9
At3g61710.3	MTR_3g018770	92	77.36
At3g61710.4	MTR_3g018770	93	74.37
At5g45900.1	MTR_0003s0540	97	68.98
At4g21980.1	MTR_2g023430	95	84.75
At4g21980.2	MTR_2g023430	85	84.75
At4g04620.1	MTR_2g023430	96	82.35
At4g04620.2	MTR_2g023430	96	82.35
At4g04620.3	MTR_2g023430	96	82.35
At1g62040.1	MTR_4g048510	96	82.35
At1g62040.1	MTR_4g037225	95	60.87
At1g62040.2	MTR_2g023430	96	82.35
At2g05630.1	MTR_4g048510	98	90.76
At2g05630.2	MTR_4g048510	65	90.74
At2g05630.1	MTR_2g088230	98	72.88
At2g45170.1	MTR_4g101090	92	83.33
At2g45170.2	MTR_4g101090	92	83.33
At4g16520.1	MTR_4g101090	96	92.31
At4g16520.2	MTR_4g101090	96	92.31
At4g16520.3	MTR_4g101090	76	86.96
At4g16520.1	MTR_1g086310	96	53.85
At3g60640.1	MTR_4g101090	99	80.99
At3g06420.1	MTR_4g123760	96	73.04
At3g15580.1	MTR_4g123760	99	71.3
At3g15580.1	MTR_7g096540	98	79.03
At2g31260.1	MTR_7g096680	99	65.31
At2g31260.1	MTR_1g070160	98	65.22
At3g07525.1	MTR_8g010140	96	55.25
At3g07525.2	MTR_8g010140	96	54.09
AT4G30790.1	MTR_4g130370	99	60.69
At1g54210.1	MTR_8g020500	94	91.3
At1g54210.2	MTR_8g020500	90	47.83
At1g54210.3	MTR_8g020500	94	91.3
At3g13970.1	MTR_8g020500	97	87.1
At3g13970.2	MTR_8g020500	80	76
At3g13970.3	MTR_8g020500	80	76.36
At3g13970.4	MTR_8g020500	80	76.36
At3g49590.1	MTR_5g068710	98	48.78
At3g49590.2	MTR_5g068710	98	48.78
At3g49590.3	MTR_5g068710	98	47.45
At3g18770.1	MTR_3g095570	95	50.16

AT1G77890.1	MTR_5g061040	96	55.88
AT1G77890.2	MTR_5g061040	96	49.34
AT1G77890.3	MTR_5g061040	96	50.88
AT4G08540.1	MTR_5g061040	99	71.42
At5g50230.1	MTR_4g104380	99	67.19
At5g50230.1	MTR_4g007500	88	56.28
At3g62770.1	MTR_1g083230	88	73.49
At3g62770.3	MTR_1g083230	87	73.5
At4g30510.1	MTR_4g130190	99	68.71
At4g30510.2	MTR_4g130190	99	72.87
At2g40810.1	MTR_7g108520	88	62.00
At2g40810.1	MTR_3g093590	98	71.92
At2g40810.2	MTR_3g093590	98	71.92
At2g40810.3	MTR_3g093590	98	73.53
At3g56440.1	MTR_3g093590	96	74.23
At3g56440.2	MTR_3g093590	96	74.59
At3g56440.3	MTR_3g093590	97	74.66
At3g56440.1	MTR_1g088855	90	53.80
At5g05150.1	MTR_3g093590	96	48.16
At5g54730.1	MTR_3g093590	88	44.62
At5g54730.2	MTR_3g093590	89	43.11
At3g56440.1	MTR_2g082770	21	29.90
At1g03380.1	MTR_1g089110	86	58
At1g54710.1	MTR_1g082300	99	53.68
At1g54710.2	MTR_1g082300	100	52.44
AT5G66930.1	MTR_8g079240	75	83.33
AT5G66930.2	MTR_8g079240	100	75.34
AT5G66930.3	MTR_8g079240	81	74.52

Glycine max

**Arabidopsis Protein accession numbers**	***Glycine max* *Protein accession numbers***	**Query Cover**	**Per. Ident**
At1g49180.1	GLYMA_04G215500	61	61.81
At1g49180.2	GLYMA_04G215500	61	61.81
At2g37840.1	GLYMA_03G069800	97	64.49
At2g37840.2	GLYMA_03G069800	93	58.89
At2g37840.3	GLYMA_03G069800	95	53.94
At2g37840.1	GLYMA_01G099600	97	63.99
At2g37840.1	GLYMA_06G150700	34	42.86

At2g37840.1	GLYMA_02G220700	98	49.84
At3g53930.1	GLYMA_03G069800	98	61.41
At3g53930.2	GLYMA_03G069800	98	61.83
At3g53930.3	GLYMA_03G069800	98	55.15
At3g53930.4	GLYMA_03G069800	98	55.72
At3g53930.5	GLYMA_03G069800	98	55.15
At3g61960.1	GLYMA_07G048400	98	52.71
At3g61960.2	GLYMA_07G048400	98	49.77
At3g61960.1	GLYMA_16G017300	98	50.82
At3g19190.1	GLYMA_02G133400	99	45.07
At3g19190.2	GLYMA_02G133400	99	47.13
At3g19190.3	GLYMA_02G133400	99	47.13
At5g61500.1	GLYMA_12G005700	99	87.22
At5g61500.2	GLYMA_12G005700	80	79.38
At5g61500.1	GLYMA_09G231000	99	78.21
At2g44140.1	GLYMA_09G244800	99	59.02
At2g44140.2	GLYMA_09G244800	96	61.12
At2g44140.3	GLYMA_09G244800	99	59.02
At2g44140.4	GLYMA_18G248400	99	62.84
At2g44140.5	GLYMA_09G244800	99	59.9
At3g59950.1	GLYMA_09G244800	99	56.22
At3g59950.2	GLYMA_09G244800	91	55.59
At3g59950.3	GLYMA_09G244800	91	65.04
At3g59950.4	GLYMA_09G244800	99	61.8
At3g59950.5	GLYMA_09G244800	99	58.23
At5g17290.1	GLYMA_14G210200	98	62.57
At5g17290.1	GLYMA_02G240700	98	62.68
At3g61710.1	GLYMA_11G153900	99	74.07
At3g61710.2	GLYMA_11G153900	94	72.8
At3g61710.3	GLYMA_11G153900	92	75.34
At3g61710.4	GLYMA_11G153900	93	74.14
At3g61710.1	GLYMA_04g141000	99	73.68
At5g45900.1	GLYMA_12G010000	98	70.52
At4g21980.1	GLYMA_15G108200	95	86.44
At4g21980.2	GLYMA_17G013000	95	79.55
At4g04620.1	GLYMA_15G108200	95	82.2
At4g04620.2	GLYMA_15G108200	95	82.2
At4g04620.3	GLYMA_15G108200	95	82.2
At4g04620.1	GLYMA_15G188600	56	74.29
At1g62040.1	GLYMA_12G098400	99	91.6
At1g62040.2	GLYMA_12G098400	88	91.6

At1g62040.1	GLYMA_06G306300	99	90.76
At1g62040.1	GLYMA_09G003900	97	88.89
At1g62040.1	GLYMA_07G261000	99	88.03
At2g05630.1	GLYMA_12G098400	99	90.83
At2g05630.2	GLYMA_12G098400	66	90.83
At2g45170.1	GLYMA_17G140700	92	84.21
At2g45170.2	GLYMA_17G140700	92	84.21
At4g16520.1	GLYMA_17G140700	95	93.16
At4g16520.2	GLYMA_17G140700	95	93.16
At4g16520.3	GLYMA_17G140700	75	88.04
At3g60640.1	GLYMA_17G140700	95	83.76
At3g06420.1	GLYMA_02G008800	94	68.14
At3g15580.1	GLYMA_02G008800	97	90.83
At2g31260.1	GLYMA_13G122200	99	84.21
At2g31260.1	GLYMA_03G162100	99	64.88
At2g31260.1	GLYMA_19G163500	99	64.88
At3g07525.1	GLYMA_03G097000	96	84.21
At3g07525.2	GLYMA_03G097000	96	93.16
At4g30790.1	GLYMA_17G071400	99	93.16
At1g54210.1	GLYMA_07G038100	94	88.04
At1g54210.2	GLYMA_16G007300	90	83.76
At1g54210.3	GLYMA_07G038100	94	68.14
At3g13970.1	GLYMA_07G038100	98	90.83
At3g13970.2	GLYMA_16G007300	79	71.68
At3g13970.3	GLYMA_16G007300	82	65.87
At3g13970.4	GLYMA_07G038100	85	65.89
At3g49590.1	GLYMA_14G187000	98	54.55
At3g49590.2	GLYMA_14G187000	98	53.64
At3g49590.3	GLYMA_14G187000	98	62.23
At3g18770.1	GLYMA_05G189000	96	90.22
AT1G77890.1	GLYMA_13G085400	96	50.22
AT1G77890.2	GLYMA_13G085400	96	49.12
AT1G77890.3	GLYMA_13G085400	96	50.22
AT4G08540.1	GLYMA_14G167200	99	70.53
At5g50230.1	GLYMA_05G043700	99	61.97
At5g50230.1	GLYMA_17G126200	99	73.43
At3g62770.1	GLYMA_20G235800	88	90.22
At3g62770.3	GLYMA_20G235800	87	82.98
At3g62770.1	GLYMA_10G152500	89	72.94
At3g62770.1	GLYMA_03G212100	99	68.94
At3g62770.1	GLYMA_19G209200	79	73.76

At4g30510.1	GLYMA_17G070200	99	74.07
At4g30510.2	GLYMA_17G070200	99	71.43
At2g40810.1	GLYMA_06G140400	99	78.67
At2g40810.2	GLYMA_06G140400	99	50.08
At2g40810.3	GLYMA_06G140400	99	50.08
At2g40810.1	GLYMA_10g126200	89	68.94
At2g40810.1	GLYMA_04g224300	96	73.76
At2g40810.1	GLYMA_07g203900	18	65.22
At3g56440.1	GLYMA_06G140400	97	48.42
At3g56440.2	GLYMA_06G140400	96	56.26
At3g56440.3	GLYMA_06G140400	93	73.57
At5g05150.1	GLYMA_06G140400	97	73.82
At5g05150.1	GLYMA_16g109400	60	25.43
At5g54730.1	GLYMA_13G287000	88	74.43
At5g54730.2	GLYMA_13G287000	89	70.65
At5g54730.1	GLYMA_12g214600	91	44.23
At5g54730.1	GLYMA_12g136000	88	43.33
At5g54730.1	GLYMA_06g267000	88	42.50
At1g03380.1	GLYMA_03G148700	86	75.00
At1g03380.1	GLYMA_19g152000	86	58.47
At1g03380.1	GLYMA_20g230900	75	56.93
At1g54710.1	GLYMA_10G157700	98	71.71
At1g54710.2	GLYMA_10G157700	99	71.71
AT5G66930.1	GLYMA_17G180900	87	82.01
AT5G66930.2	GLYMA_17G180900	100	76.71
AT5G66930.3	GLYMA_17G180900	81	75.96

B.KEGG Orthology analysis of *A. thaliana, P. vulgaris, M. truncatula,and G.max*.

**KEGG ID**	**Arabidopsis thaliana**	**Phaseolus vulgaris**	**Medicago truncatula**	**Glycine max**
GrpGenusOrganismK07204	**ath**	**pvu**	**mtr**	**gmx ***
(RAPTOR) [590] K07203	AT3G08850<br>AT5G01770	PHAVU_008G08780<br>PHAVU_008G08810	MTR_7g072330	100777178<br>100779159
(MTOR)[616]K08266	AT1G50030	PHAVU_002G04990	MTR_5g005380	100816558<br>100816446
(MLST8)[544]K08269	AT2G22040<br>AT3G18140	PHAVU_006G17370	MTR_2g016690	100781599<br>100812024
(ULK2)[656]K08331	AT2G37840<br>AT3G53930	PHAVU_010G01510	MTR_4g019410	100791596
(ATG13)[641]K19730	AT3G18770<br>AT3G49590	PHAVU_002G26960<br>PHAVU_008G18780	MTR_8g093050<br>MTR_5g068710<br>MTR_3g095570	100778254<br>100798585<br>100818104<br>100816476
(ATG101)[514]K08330	AT5G66930	PHAVU_003G24800	MTR_8g079240	100811916

(ATG11)[272]K17606	AT4G30790	PHAVU_003G15380	MTR_4g130370	100794018<br>100792447
(IGBP1)[592]K04382	AT5G53000	PHAVU_009G05190	MTR_3g091640<br>MTR_1g050518	100780847<br>100807894
(PPP2C)[1332]K17907	AT1G10430<br>AT1G59830<br>AT1G69960<br>AT2G42500<br>AT3G58500	PHAVU_007G26220<br>PHAVU_003G07260<br>PHAVU_002G07880<br>PHAVU_002G23540<br>PHAVU_001G20030	MTR_8g085610<br>MTR_5g037200<br>MTR_8g062430<br>MTR_7g107310	100783950<br>100784312<br>100794029<br>100801108<br>100807329<br>100798408<br>100817449<br>100782279<br>100775812<br>100812326
(ATG9)[712]K17906	AT2G31260	PHAVU_007G19430<br>PHAVU_001G15990	MTR_1g070160<br>MTR_7g096680	100777405<br>100779344<br>100809347<br>732654
(ATG2)[646]K17908	AT3G19190	PHAVU_003G29580	MTR_4g086370	100802641<br>100805552
(WIPI1_2)[751]K08334	AT4G30510	PHAVU_003G15280	MTR_4g130190	100794908<br>100803048<br>100820465<br>100816367
(BECN)[598]K00914	AT3G61710	PHAVU_005G02990	MTR_3g018770	100812721<br>732646
(PIK3C3)[590]K08333	AT1G60490	PHAVU_002G07010	MTR_5g034120	547983<br>100778348
(PIK3R4)[527]K08336	AT4G29380	PHAVU_004G17510	MTR_6g088835	100796837<br>100818467
(ATG12)[519]K08339	AT3G13970<br>AT1G54210	PHAVU_010G13030	MTR_8g020500	100527905<br>100527733
(ATG5)[511]K17890	AT5G17290	PHAVU_008G24100	MTR_5g076920	732567<br>100789045
(ATG16L1)[426]K08337	AT5G50230	PHAVU_003G20710	MTR_3g075400<br>MTR_4g104380<br>MTR_4g007500	100806054<br>100778336
(ATG7)[535]K17888	AT5G45900	PHAVU_011G01070	MTR_0003s0540	100781725
(ATG10L)[432]K08343	AT3G07525	PHAVU_010G03630	MTR_8g010140	100786914<br>100808589
(ATG3)[588]K08341	AT5G61500	PHAVU_011G00650	MTR_4g036265	100787410<br>100814257
(GABARAP)[1655]K08342	AT2G05630<br>AT4G16520<br>AT4G04620<br>AT4G21980<br>AT1G62040<br>AT2G45170<br>AT3G60640<br>AT3G06420<br>AT3G15580	PHAVU_011G15160<br>PHAVU_007G21080<br>PHAVU_003G21960<br>PHAVU_011G10330<br>PHAVU_002G06220<br>PHAVU_003G07930	MTR_4g101090<br>MTR_4g048510<br>MTR_7g096540<br>MTR_2g023430<br>MTR_1g086310<br>MTR_4g123760<br>MTR_4g037225<br>MTR_2g088230	100301895<br>100499835<br>100527884<br>100781703<br>100818620<br>100818315<br>100500165<br>100305892<br>100500023<br>100527345<br>100301894<br>100784677
(ATG4)[1152]	AT2G44140	PHAVU_008G04890	MTR_7g081230	100809664<br>100810317

*

Glycine max gmx	Glycine max Ids gmx
100777178	GLYMA_09G278500
100779159	GLYMA_18G210300
100816558	GLYMA_01G241300
100816446	GLYMA_11G002600
100781599	GLYMA_15G085200
100812024	GLYMA_13G227200
100791596	GLYMA_03G069800
100778254	GLYMA_02G220700
100798585	GLYMA_08G146700
100818104	GLYMA_14G187000
100816476	GLYMA_05G189000
100811916	GLYMA_17G180900
100794018	GLYMA_02G206500
100792447	GLYMA_17G071400
100780847	GLYMA_06G129600
100807894	GLYMA_04G234900
100783950	GLYMA_08G293400
100784312	GLYMA_01G038800
100794029	GLYMA_02G025900
100801108	GLYMA_02G169200
100807329	GLYMA_03G206900

C.ENSEMBL Orthology analysis of *A. thaliana, P. vulgaris, M. truncatula,*and *G.max*.

ID Arabidopsis	Species	Type	Orthologue	dN/dS	Target %id	Query %id	GOC Score	WGA Coverage	High Confidence
At1g49180	Species without orthologues								
At2g37840	Phaseolus vulgaris	1-to-manyView Gene Tree	PHAVU_010G015100g<br>Compare Regions (10:2,437,128-2,443,461:-1)<br>View Sequence Alignments	n/a	53.40 %	47.20 %	n/a	**93.18**	Yes
At3g53930	Phaseolus vulgaris	1-to-manyView Gene Tree	PHAVU_010G015100g<br>Compare Regions (10:2,437,128-2,443,461:-1)<br>View Sequence Alignments	n/a	51.85 %	47.19 %	n/a	**91.04**	Yes
At3g61960	Phaseolus vulgaris	1-to-1View Gene Tree	PHAVU_010G120500g<br>Compare Regions (10:38,972,887-38,977,458:-1)<br>View Sequence Alignments	n/a	46.41 %	46.49 %	n/a	**85.79**	Yes
At3g19190	Phaseolus vulgaris	1-to-1View Gene Tree	PHAVU_003G295800g<br>Compare Regions (3:52,098,694-52,109,253:1)<br>View Sequence Alignments	n/a	44.76 %	46.78 %	n/a	**95.15**	No
At5g61500	Phaseolus vulgaris	1-to-1View Gene Tree	PHAVU_011G006500g<br>Compare Regions (11:475,609-480,221:1)<br>View Sequence Alignments	0.04384	85.67 %	85.94 %	n/a	**100**	No
At2g44140	Phaseolus vulgaris	1-to-manyView Gene Tree	PHAVU_008G048900g<br>Compare Regions (8:4,303,200-4,308,640:1)<br>View Sequence Alignments	n/a	57.87 %	60.60 %	n/a	**93.4**	No
At3g59950	Phaseolus vulgaris	1-to-manyView Gene Tree	PHAVU_008G048900g<br>Compare Regions (8:4,303,200-4,308,640:1)<br>View Sequence Alignments	n/a	55.83 %	57.23 %	n/a	**93.29**	No
At5g17290	haseolus vulgaris	1-to-1View Gene Tree	PHAVU_008G241000g<br>Compare Regions (8:55,537,709-55,545,667:1)<br>View Sequence Alignments	n/a	59.60 %	61.72 %	n/a	**98.51**	No
At3g61710	Phaseolus vulgaris	1-to-1View Gene Tree	PHAVU_005G029900g<br>Compare Regions (5:2,774,177-2,781,388:1)<br>View Sequence Alignments	0.07597	73.82 %	69.83 %	n/a	**99.97**	Yes
At5g45900	Phaseolus vulgaris	1-to-1View Gene Tree	PHAVU_011G010700g<br>Compare Regions (11:816,956-822,085:-1)<br>View Sequence Alignments	n/a	68.57 %	68.87 %	n/a	**98.91**	No
At4g21980	Species without orthologues								
At4g04620	Species without orthologues								
At1g62040	Species without orthologues								
At2g05630	Phaseolus vulgaris	1-to-1View Gene Tree	PHAVU_011G103300g<br>Compare Regions (11:11,625,632-11,627,983:-1)<br>View Sequence Alignments	0.02628	89.17 %	65.24 %	n/a	**80.07**	Yes
At2g45170	Species without orthologues								
At4g16520	Species without orthologues								
At3g60640	Species without orthologues								
At3g06420	Phaseolus vulgaris	1-to-1View Gene Tree	PHAVU_007G210800g<br>Compare Regions (7:44,931,240-44,932,490:1)<br>View Sequence Alignments	n/a	63.11 %	64.71 %	n/a	**95.95**	No
At3g15580	Species without orthologues								
At2g31260	Phaseolus vulgaris	1-to-manyView Gene Tree	PHAVU_001G159900g<br>Compare Regions (1:41,950,751-41,958,196:1)<br>View Sequence Alignments	n/a	65.34 %	64.67 %	n/a	**98.58**	No
	Phaseolus vulgaris	1-to-manyView Gene Tree	PHAVU_007G194300g<br>Compare Regions (7:43,257,363-43,263,137:-1)<br>View Sequence Alignments	n/a	58.19 %	58.66 %	n/a	**79.44**	No
At3g07525	Species without orthologues								
AT4G30790	Phaseolus vulgaris	1-to-1View Gene Tree	PHAVU_003G153800g<br>Compare Regions (3:35,797,412-35,804,197:-1)<br>View Sequence Alignments	n/a	60.62 %	60.89 %	n/a	**100**	No
At1g54210	Phaseolus vulgaris	1-to-manyView Gene Tree	PHAVU_010G130300g<br>Compare Regions (10:40,034,779-40,037,243:-1)<br>View Sequence Alignments	n/a	87.23 %	85.42 %	n/a	**89.65**	Yes
	Phaseolus vulgaris	1-to-manyView Gene Tree	PHAVU_010G130300g<br>Compare Regions (10:40,034,779-40,037,243:-1)<br>View Sequence Alignments	n/a	82.98 %	82.98 %	n/a	**90.45**	Yes
At3g13970	Species without orthologues								
At3g49590	Species without orthologues								
At3g18770	Phaseolus vulgaris	1-to-1View Gene Tree	PHAVU_002G269600g<br>Compare Regions (2:43,452,066-43,457,109:1)<br>View Sequence Alignments	n/a	52.00 %	52.00 %	n/a	**92.96**	No
AT1G77890	Species without orthologues								
AT4G08540	Phaseolus vulgaris	1-to-manyView Gene Tree	03g22220	n/a	67.65 %	68.08 %	n/a	**97.88**	No

At5g50230	Phaseolus vulgaris	1-to-1View Gene Tree	PHAVU_003G207100g<br>Compare Regions (3:42,141,096-42,144,835:1)<br>View Sequence Alignments	n/a	71.79 %	72.50 %	n/a	**99.94**	No
At3g62770	Phaseolus vulgaris	1-to-1View Gene Tree	PHAVU_001G205000g<br>Compare Regions (1:46,989,620-46,992,624:1)<br>View Sequence Alignments	n/a	62.65 %	62.35 %	n/a	**98.54**	No
At4g30510	Phaseolus vulgaris	1-to-1View Gene Tree	PHAVU_003G152800g<br>Compare Regions (3:35,700,014-35,706,391:-1)<br>View Sequence Alignments	0.11637	68.52 %	67.21 %	n/a	**94.29**	No
At2g40810	Phaseolus vulgaris	1-to-manyView Gene Tree	PHAVU_009G041700g<br>Compare Regions (9:8,255,010-8,259,849:-1)<br>View Sequence Alignments	0.13407	63.51 %	68.19 %	n/a	**91.13**	No
	Phaseolus vulgaris	1-to-manyView Gene Tree	PHAVU_009G041700g<br>Compare Regions (9:8,255,010-8,259,849:-1)<br>View Sequence Alignments	0.1555	61.14 %	65.98 %	n/a	**90.44**	No
At3g56440	Phaseolus vulgaris	1-to-manyView Gene Tree	PHAVU_009G041700g<br>Compare Regions (9:8,255,010-8,259,849:-1)<br>View Sequence Alignments	0.1555	61.14 %	65.98 %	n/a	**90.44**	No
At5g05150	Species without orthologues								
At5g54730	Phaseolus vulgaris	1-to-manyView Gene Tree	PHAVU_005G091300g<br>Compare Regions (5:26,142,022-26,148,175:-1)<br>View Sequence Alignments	n/a	33.86 %	39.45 %	n/a	**84.88**	No
	Phaseolus vulgaris	1-to-manyView Gene Tree	PHAVU_011G140900g<br>Compare Regions (11:33,681,120-33,687,304:1)<br>View Sequence Alignments	n/a	30.81 %	37.35 %	n/a	**82.63**	No
At1g03380	Phaseolus vulgaris	1-to-1View Gene Tree	PHAVU_001G146700g<br>Compare Regions (1:39,969,412-39,975,976:-1)<br>View Sequence Alignments	n/a	49.03 %	49.84 %	n/a	**82.26**	Yes
At1g54710	Phaseolus vulgaris	1-to-1View Gene Tree	PHAVU_007G183100g<br>Compare Regions (7:41,925,427-41,932,753:1)<br>View Sequence Alignments	n/a	54.69 %	53.51 %	n/a	**92.14**	No
AT5G66930	Phaseolus vulgaris	1-to-1View Gene Tree	PHAVU_003G248000g<br>Compare Regions (3:47,394,870-47,402,376:1)<br>View Sequence Alignments	n/a	72.02 %	62.55 %	n/a	**84.41**	No

ID Arabidopsis	***Species***	***Type***	***Orthologue***	***dN/dS***	***Target %id***	***Query %id***	***GOC Score***	***WGA Coverage***	***High Confidence***
At1g49180	Medicago truncatula	1-to-1View Gene Tree	MTR_3g095620<br>Compare Regions (3:43,689,826-43,692,334:1)<br>View Sequence Alignments	n/a	50.69 %	36.03 %	n/a	**79.09**	No
At2g37840	Medicago truncatula	1-to-manyView Gene Tree	MTR_4g019410<br>Compare Regions (4:6,057,862-6,065,974:1)<br>View Sequence Alignments	0.17581	59.70 %	60.03 %	n/a	**97.36**	Yes
At3g53930	Medicago truncatula	1-to-manyView Gene Tree	MTR_4g019410<br>Compare Regions (4:6,057,862-6,065,974:1)<br>View Sequence Alignments	0.15035	57.67 %	59.69 %	n/a	**99.38**	Yes
At3g61960	Medicago truncatula	1-to-1View Gene Tree	MTR_8g024100<br>Compare Regions (8:8,817,813-8,824,200:1)<br>View Sequence Alignments	n/a	43.82 %	48.72 %	n/a	**86.73**	Yes
At3g19190	Medicago truncatula	1-to-1View Gene Tree	MTR_4g086370<br>Compare Regions (4:33,827,078-33,844,760:-1)<br>View Sequence Alignments	n/a	44.86 %	46.83 %	n/a	**95.7**	No
At5g61500	Medicago truncatula	1-to-1View Gene Tree	MTR_4g036265<br>Compare Regions (4:13,052,245-13,057,301:1)<br>View Sequence Alignments	0.04108	85.48 %	84.66 %	n/a	**100**	No
At2g44140	Medicago truncatula	1-to-manyView Gene Tree	ATG4 (MTR_7g081230)<br>Compare Regions (7:30,993,699-30,998,401:-1)<br>View Sequence Alignments	0.14879	58.32 %	60.81 %	n/a	**97.84**	No
At3g59950	Medicago truncatula	1-to-manyView Gene Tree	ATG4 (MTR_7g081230)<br>Compare Regions (7:30,993,699-30,998,401:-1)<br>View Sequence Alignments	0.17612	55.44 %	56.60 %	n/a	**93.4**	No
At5g17290	Medicago truncatula	1-to-1View Gene Tree	MTR_5g076920<br>Compare Regions (5:32,806,624-32,813,118:-1)<br>View Sequence Alignments	n/a	56.79 %	60.83 %	n/a	**98.72**	No
At3g61710	Medicago truncatula	1-to-1View Gene Tree	MTR_3g018770<br>Compare Regions (3:5,165,817-5,174,556:1)<br>View Sequence Alignments	0.07716	73.08 %	71.95 %	n/a	**98.36**	Yes
At5g45900	Medicago truncatula	1-to-1View Gene Tree	MTR_0003s0540<br>Compare Regions (scaffold0003:305,855-310,747:1)<br>View Sequence Alignments	n/a	67.19 %	67.29 %	n/a	**99.29**	No
At4g21980	Species without orthologues								
At4g04620	Species without orthologues								
At1g62040	Species without orthologues								
At2g05630	Medicago truncatula	1-to-manyView Gene Tree	MTR_2g088230<br>Compare Regions (2:37,163,050-37,165,680:-1)<br>View Sequence Alignments	n/a	87.04 %	57.32 %	n/a	**78.12**	Yes
	Medicago truncatula	1-to-manyView Gene Tree	MTR_4g048510<br>Compare Regions (4:17,207,135-17,210,565:-1)<br>View Sequence Alignments	0.02767	88.33 %	64.63 %	n/a	**86.12**	Yes
At2g45170	Species without orthologues								
At4g16520	Species without orthologues								

At3g60640	Species without orthologues								
At3g06420	Medicago truncatula	1-to-manyView Gene Tree	MTR_4g123760<br>Compare Regions (4:51,007,802-51,010,377:-1)<br>View Sequence Alignments	n/a	71.19 %	70.59 %	n/a	**89.67**	Yes
	Medicago truncatula	1-to-manyView Gene Tree	MTR_7g096540<br>Compare Regions (7:38,739,985-38,740,615:1)<br>View Sequence Alignments	n/a	74.19 %	38.66 %	n/a	n/a	Yes
At3g15580	Species without orthologues								
At2g31260	Medicago truncatula	1-to-manyView Gene Tree	MTR_1g070160<br>Compare Regions (1:30,830,518-30,837,261:-1)<br>View Sequence Alignments	n/a	63.39 %	63.39 %	n/a	**81.43**	No
	Medicago truncatula	1-to-manyView Gene Tree	MTR_7g096680<br>Compare Regions (7:38,799,346-38,805,558:1)<br>View Sequence Alignments	n/a	62.49 %	64.43 %	n/a	**99.56**	No
At3g07525	Species without orthologues								
AT4G30790	Medicago truncatula	1-to-1View Gene Tree	MTR_4g130370<br>Compare Regions (4:54,307,709-54,314,660:-1)<br>View Sequence Alignments	n/a	60.57 %	60.89 %	n/a	**100**	No
At1g54210	Medicago truncatula	1-to-manyView Gene Tree	MTR_8g020500<br>Compare Regions (8:7,198,686-7,202,464:1)<br>View Sequence Alignments	n/a	67.74 %	87.50 %	n/a	**80.1**	Yes
At1g54210	Medicago truncatula	1-to-manyView Gene Tree	MTR_8g020500<br>Compare Regions (8:7,198,686-7,202,464:1)<br>View Sequence Alignments	n/a	65.32 %	86.17 %	n/a	**82.59**	Yes
At3g13970	Species without orthologues								
At3g49590	Species without orthologues								
At3g18770	Medicago truncatula	1-to-manyView Gene Tree	MTR_3g095570<br>Compare Regions (3:43,671,041-43,677,624:-1)<br>View Sequence Alignments	n/a	46.45 %	47.04 %	n/a	**97.3**	No
	Medicago truncatula	1-to-manyView Gene Tree	MTR_8g093050<br>Compare Regions (8:38,885,014-38,889,871:1)<br>View Sequence Alignments	n/a	44.43 %	41.44 %	n/a	**87.12**	No
AT1G77890	Species without orthologues								
AT4G08540	Medicago truncatula	1-to-manyView Gene Tree	MTR_5g061040<br>Compare Regions (5:25,385,477-25,394,644:-1)<br>View Sequence Alignments	n/a	67.01 %	67.86 %	n/a	**99.1**	No
At5g50230	Medicago truncatula	1-to-manyView Gene Tree	MTR_3g075400<br>Compare Regions (3:34,315,394-34,318,708:-1)<br>View Sequence Alignments	n/a	67.19 %	67.19 %	n/a	**99.94**	No
	Medicago truncatula	1-to-manyView Gene Tree	MTR_4g007500<br>Compare Regions (4:1,115,999-1,117,649:1)<br>View Sequence Alignments	n/a	60.99 %	43.61 %	n/a	**95.24**	No
	Medicago truncatula	1-to-manyView Gene Tree	MTR_4g104380<br>Compare Regions (4:43,185,561-43,189,052:-1)<br>View Sequence Alignments	n/a	69.65 %	70.33 %	n/a	**99.22**	No
At3g62770	Medicago truncatula	1-to-manyView Gene Tree	MTR_1g088855<br>Compare Regions (1:39,776,324-39,778,721:-1)<br>View Sequence Alignments	n/a	61.30 %	51.06 %	n/a	**94.59**	No
	Medicago truncatula	1-to-manyView Gene Tree	MTR_7g108520<br>Compare Regions (7:44,206,217-44,209,925:1)<br>View Sequence Alignments	n/a	64.11 %	63.06 %	n/a	**99.43**	No
At4g30510	Medicago truncatula	1-to-1View Gene Tree	MTR_4g130190<br>Compare Regions (4:54,209,571-54,215,694:-1)<br>View Sequence Alignments	n/a	66.13 %	67.21 %	n/a	**90.93**	No
At2g40810	Medicago truncatula	1-to-manyView Gene Tree	MTR_3g093590<br>Compare Regions (3:42,763,022-42,768,303:1)<br>View Sequence Alignments	n/a	68.67 %	72.52 %	n/a	**97.9**	No
At3g56440	Medicago truncatula	1-to-manyView Gene Tree	MTR_3g093590<br>Compare Regions (3:42,763,022-42,768,303:1)<br>View Sequence Alignments	0.11384	67.23 %	71.36 %	n/a	**91.19**	No
At5g05150	Species without orthologues								
At5g54730	Medicago truncatula	1-to-1View Gene Tree	MTR_2g082770<br>Compare Regions (2:34,727,900-34,734,357:-1)<br>View Sequence Alignments	n/a	34.41 %	40.63 %	n/a	**85.36**	No
At1g03380	Medicago truncatula	1-to-1View Gene Tree	MTR_1g089110<br>Compare Regions (1:40,103,141-40,108,943:-1)<br>View Sequence Alignments	0.19286	50.41 %	50.89 %	n/a	**83.24**	Yes
At1g54710	Medicago truncatula	1-to-1View Gene Tree	MTR_1g082300<br>Compare Regions (1:36,587,909-36,596,198:-1)<br>View Sequence Alignments	n/a	52.68 %	51.89 %	n/a	**90.75**	No
AT5G66930	Medicago truncatula	1-to-1View Gene Tree	MTR_8g079240<br>Compare Regions (8:33,765,931-33,771,318:-1)<br>View Sequence Alignments	n/a	70.64 %	61.35 %	n/a	**92.37**	No

ID Arabidopsis	Species	Type	Orthologue	dN/dS	Target %id	Query %id	GOC Score	WGA Coverage	High Confidence
At1g49180	Glycine max	1-to-manyView Gene Tree	GLYMA_04G215500 Compare Regions (4:48,694,717-48,697,763:-1) View Sequence Alignments	n/a	43.09 %	38.97 %	n/a	**71.35**	No
	Glycine max	1-to-manyView Gene Tree	GLYMA_06G150700 Compare Regions (6:12,289,992-12,292,323:1) View Sequence Alignments	0.1922	44.66 %	33.82 %	n/a	**73.54**	No
At2g37840	Glycine max	Many-to-manyView Gene Tree	GLYMA_01G099600 Compare Regions (1:33,115,843-33,125,726:-1) View Sequence Alignments	0.18238	60.00 %	59.35 %	n/a	**97.91**	Yes
	Glycine max	Many-to-manyView Gene Tree	GLYMA_03G069800 Compare Regions (3:15,703,649-15,719,178:-1) View Sequence Alignments	0.16133	60.27 %	60.44 %	n/a	**97.92**	Yes
At3g53930	Glycine max	Many-to-manyView Gene Tree	GLYMA_01G099600 Compare Regions (1:33,115,843-33,125,726:-1) View Sequence Alignments	0.17792	58.62 %	59.69 %	n/a	**96.24**	Yes
	Glycine max	Many-to-manyView Gene Tree	GLYMA_03G069800 Compare Regions (3:15,703,649-15,719,178:-1) View Sequence Alignments	0.16097	58.23 %	60.11 %	n/a	n/a	Yes
At3g61960	Glycine max	1-to-manyView Gene Tree	GLYMA_07G048400 Compare Regions (7:4,096,301-4,102,195:1) View Sequence Alignments	n/a	48.04 %	50.96 %	n/a	**87.4**	Yes
	Glycine max	1-to-manyView Gene Tree	GLYMA_16G017300 Compare Regions (16:1,519,674-1,525,290:1) View Sequence Alignments	n/a	45.04 %	50.00 %	n/a	**89.35**	Yes
At3g19190	Glycine max	1-to-manyView Gene Tree	GLYMA_02G133400 Compare Regions (2:13,780,268-13,793,313:-1) View Sequence Alignments	n/a	45.42 %	47.46 %	n/a	**95.15**	No
	Glycine max	1-to-manyView Gene Tree	GLYMA_07G211600 Compare Regions (7:38,313,562-38,326,234:1) View Sequence Alignments	n/a	45.35 %	47.41 %	n/a	**95.29**	No
At5g61500	Glycine max	1-to-manyView Gene Tree	GLYMA_09G231000 Compare Regions (9:45,443,954-45,447,812:-1) View Sequence Alignments	0.05542	85.07 %	78.27 %	n/a	**90.02**	No
	Glycine max	1-to-manyView Gene Tree	GLYMA_12G005700 Compare Regions (12:433,113-437,871:1) View Sequence Alignments	0.04154	87.22 %	87.22 %	n/a	**100**	No
At2g44140	Glycine max	Many-to-manyView Gene Tree	GLYMA_09G244800 Compare Regions (9:46,708,330-46,712,967:1) View Sequence Alignments	n/a	58.64 %	61.03 %	n/a	**92.57**	No
	Glycine max	Many-to-manyView Gene Tree	GLYMA_18G248400 Compare Regions (18:53,539,192-53,544,229:-1) View Sequence Alignments	n/a	59.18 %	61.46 %	n/a	**1.87**	No
At3g59950	Glycine max	Many-to-manyView Gene Tree	GLYMA_09G244800 Compare Regions (9:46,708,330-46,712,967:1) View Sequence Alignments	0.17607	56.17 %	57.23 %	n/a	**93.33**	No
	Glycine max	Many-to-manyView Gene Tree	GLYMA_18G248400 Compare Regions (18:53,539,192-53,544,229:-1) View Sequence Alignments	0.18877	55.67 %	56.60 %	n/a	**93.57**	No
At5g17290	Glycine max	1-to-manyView Gene Tree	GLYMA_02G240700 Compare Regions (2:42,910,921-42,918,001:-1) View Sequence Alignments	n/a	59.71 %	62.02 %	n/a	**99.42**	No
	Glycine max	1-to-manyView Gene Tree	GLYMA_14G210200 Compare Regions (14:47,535,839-47,545,840:-1) View Sequence Alignments	n/a	60.17 %	62.31 %	n/a	**99.01**	No
At3g61710	Glycine max	1-to-manyView Gene Tree	GLYMA_04G141000 Compare Regions (4:23,665,626-23,682,229:-1) View Sequence Alignments	n/a	72.69 %	71.57 %	n/a	**98.19**	Yes
	Glycine max	1-to-manyView Gene Tree	GLYMA_11G153900 Compare Regions (11:12,389,260-12,396,651:-1) View Sequence Alignments	0.0854	72.89 %	71.76 %	n/a	**98.29**	Yes
At5g45900	Glycine max	1-to-1View Gene Tree	GLYMA_12G010000 Compare Regions (12:712,288-717,264:1) View Sequence Alignments	n/a	68.95 %	67.86 %	n/a	**98.09**	No
At4g21980	Glycine max	1-to-manyView Gene Tree	GLYMA_15G188600 Compare Regions (15:19,737,953-19,738,255:-1) View Sequence Alignments	n/a	73.97 %	39.42 %	n/a	n/a	Yes
At4g04620	Glycine max	1-to-manyView Gene Tree	GLYMA_15G188600 Compare Regions (15:19,737,953-19,738,255:-1) View Sequence Alignments	n/a	71.23 %	42.62 %	n/a	n/a	Yes
At1g62040	Species without orthologues								
At2g05630	Glycine max	1-to-manyView Gene Tree	GLYMA_06G306300 Compare Regions (6:49,529,272-49,531,512:1) View Sequence Alignments	0.04223	87.50 %	64.02 %	n/a	**88.13**	Yes
	Glycine max	1-to-manyView Gene Tree	GLYMA_12G098400 Compare Regions (12:8,463,842-8,466,127:-1) View Sequence Alignments	0.02436	88.33 %	64.63 %	n/a	**88.97**	Yes
At2g45170	Species without orthologues								
At4g16520	Species without orthologues								
At3g60640	Species without orthologues								
At3g06420	Glycine max	1-to-manyView Gene Tree	GLYMA_02G008800 Compare Regions (2:882,915-884,424:1) View Sequence Alignments	n/a	63.11 %	64.71 %	n/a	n/a	No
	Glycine max	1-to-manyView Gene Tree	GLYMA_10G009300 Compare Regions (10:896,609-897,903:1) View Sequence Alignments	n/a	63.11 %	64.71 %	n/a	**73.91**	No

At3g15580	Species without orthologues								
At2g31260	Glycine max	1-to-manyView Gene Tree	GLYMA_03G162100<br>Compare Regions (3:37,703,629-37,711,157:1)<br>View Sequence Alignments	n/a	64.45 %	64.90 %	n/a	**81.59**	No
	Glycine max	1-to-manyView Gene Tree	GLYMA_10G035800<br>Compare Regions (10:3,130,966-3,138,037:1)<br>View Sequence Alignments	n/a	65.82 %	65.59 %	n/a	**99.15**	No
	Glycine max	1-to-manyView Gene Tree	GLYMA_13G122200<br>Compare Regions (13:23,509,326-23,515,360:1)<br>View Sequence Alignments	n/a	65.09 %	65.24 %	n/a	**96.58**	No
	Glycine max	1-to-manyView Gene Tree	GLYMA_19G163500<br>Compare Regions (19:42,435,811-42,443,348:1)<br>View Sequence Alignments	n/a	63.52 %	63.74 %	n/a	**82.35**	No
At3g07525	Species without orthologues								
AT4G30790	Glycine max	1-to-manyView Gene Tree	GLYMA_02G206500<br>Compare Regions (2:39,154,706-39,161,370:1)<br>View Sequence Alignments	n/a	61.35 %	61.67 %	n/a	**99.74**	No
	Glycine max	1-to-manyView Gene Tree	GLYMA_17G071400<br>Compare Regions (17:5,578,787-5,585,492:-1)<br>View Sequence Alignments	n/a	61.94 %	62.37 %	n/a	**97.89**	No
At1g54210	Glycine max	Many-to-manyView Gene Tree	GLYMA_07G038100<br>Compare Regions (7:3,134,753-3,137,886:1)<br>View Sequence Alignments	n/a	88.30 %	86.46 %	n/a	**88.85**	Yes
	Glycine max	Many-to-manyView Gene Tree	GLYMA_16G007300<br>Compare Regions (16:599,220-602,627:1)<br>View Sequence Alignments	n/a	67.37 %	66.67 %	n/a	**85.16**	Yes
At3g13970	Glycine max	Many-to-manyView Gene Tree	GLYMA_07G038100<br>Compare Regions (7:3,134,753-3,137,886:1)<br>View Sequence Alignments	n/a	82.98 %	82.98 %	n/a	**89.56**	Yes
At3g49590	Glycine max	Many-to-manyView Gene Tree	GLYMA_16G007300<br>Compare Regions (16:599,220-602,627:1)<br>View Sequence Alignments	n/a	63.16 %	63.83 %	n/a	n/a	Yes
At3g49590	Glycine max	1-to-manyView Gene Tree	GLYMA_02G220700<br>Compare Regions (2:40,856,235-40,861,423:-1)<br>View Sequence Alignments	n/a	46.80 %	44.98 %	n/a	**96.63**	Yes
	Glycine max	1-to-manyView Gene Tree	GLYMA_14G187000<br>Compare Regions (14:45,159,106-45,164,166:1)<br>View Sequence Alignments	n/a	47.80 %	45.79 %	n/a	**97.4**	Yes
At3g18770	Glycine max	1-to-manyView Gene Tree	GLYMA_05G189000<br>Compare Regions (5:37,485,611-37,491,652:1)<br>View Sequence Alignments	n/a	54.05 %	53.44 %	n/a	**96.26**	No
	Glycine max	1-to-manyView Gene Tree	GLYMA_08G146700<br>Compare Regions (8:11,166,509-11,172,366:1)<br>View Sequence Alignments	n/a	53.61 %	53.44 %	n/a	**98.06**	No
AT1G77890	Species without orthologues								
AT4G08540	Glycine max	Many-to-manyView Gene Tree	GLYMA_13G085400<br>Compare Regions (13:19,705,658-19,715,073:-1)<br>View Sequence Alignments	n/a	67.51 %	67.65 %	n/a	**97.58**	No
	Glycine max	Many-to-manyView Gene Tree	GLYMA_14G167200<br>Compare Regions (14:41,352,672-41,365,398:-1)<br>View Sequence Alignments	n/a	66.74 %	67.02 %	n/a	**98.11**	No
At5g50230	Glycine max	1-to-manyView Gene Tree	GLYMA_05G043700<br>Compare Regions (5:3,901,985-3,905,389:1)<br>View Sequence Alignments	n/a	72.37 %	73.08 %	n/a	**100**	No
	Glycine max	1-to-manyView Gene Tree	GLYMA_17G126200<br>Compare Regions (17:10,054,028-10,058,177:1)<br>View Sequence Alignments	n/a	72.18 %	72.89 %	n/a	**99.54**	No
At3g62770	Glycine max	1-to-manyView Gene Tree	GLYMA_03G212100<br>Compare Regions (3:41,817,313-41,820,500:1)<br>View Sequence Alignments	n/a	66.19 %	65.88 %	n/a	**98.57**	No
	Glycine max	1-to-manyView Gene Tree	GLYMA_19G209200<br>Compare Regions (19:46,393,051-46,396,379:1)<br>View Sequence Alignments	n/a	64.30 %	64.00 %	n/a	**90.99**	No
At4g30510	Glycine max	1-to-manyView Gene Tree	GLYMA_02G207500<br>Compare Regions (2:39,248,283-39,253,677:1)<br>View Sequence Alignments	n/a	67.93 %	68.31 %	n/a	**93.81**	No
	Glycine max	1-to-manyView Gene Tree	GLYMA_10G126200<br>Compare Regions (10:33,430,366-33,432,117:-1)<br>View Sequence Alignments	n/a	60.96 %	24.32 %	n/a	**69.62**	No
	Glycine max	1-to-manyView Gene Tree	GLYMA_16G109400<br>Compare Regions (16:23,481,725-23,483,856:-1)<br>View Sequence Alignments	0.114<br>85	64.11 %	36.61 %	n/a	**83.4**	No
	Glycine max	1-to-manyView Gene Tree	GLYMA_17G070200<br>Compare Regions (17:5,495,613-5,501,594:-1)<br>View Sequence Alignments	n/a	68.56 %	69.13 %	n/a	**83.23**	No
	Glycine max	Many-to-manyView Gene Tree	GLYMA_04G224300<br>Compare Regions (4:49,475,519-49,486,851:1)<br>View Sequence Alignments	0.105<br>99	68.50 %	73.03 %	n/a	**96.88**	No
	Glycine max	Many-to-manyView Gene Tree	GLYMA_06G140400<br>Compare Regions (6:11,446,439-11,452,684:-1)<br>View Sequence Alignments	0.099<br>11	68.10 %	72.77 %	n/a	**98.94**	No
At2g40810	Glycine max	Many-to-manyView Gene Tree	GLYMA_04G224300<br>Compare Regions (4:49,475,519-49,486,851:1)<br>View Sequence Alignments	0.105<br>99	68.50 %	73.03 %	n/a	**96.88**	No
	Glycine max	Many-to-manyView Gene Tree	GLYMA_06G140400<br>Compare Regions (6:11,446,439-11,452,684:-1)<br>View Sequence Alignments	0.099<br>11	68.10 %	72.77 %	n/a	**98.94**	No
At3g56440	Glycine max	Many-to-manyView Gene Tree	GLYMA_04G224300<br>Compare Regions (4:49,475,519-49,486,851:1)<br>View Sequence Alignments	0.127<br>83	65.87 %	70.59 %	n/a	**91.99**	No

	Glycine max	Many-to-manyView Gene Tree	GLYMA_06G140400 Compare Regions (6:11,446,439-11,452,684:-1) View Sequence Alignments	0.1263	65.71 %	70.59 %	n/a	**4.52**	No
At5g05150	Species without orthologues								
At5g54730	Glycine max	1-to-manyView Gene Tree	GLYMA_06G267000 Compare Regions (6:45,497,961-45,504,163:-1) View Sequence Alignments	n/a	32.15 %	38.14 %	n/a	**84.63**	No
	Glycine max	1-to-manyView Gene Tree	GLYMA_12G136000 Compare Regions (12:15,957,977-15,965,220:1) View Sequence Alignments	n/a	33.00 %	38.93 %	n/a	**83.56**	No
	Glycine max	1-to-manyView Gene Tree	GLYMA_12G214600 Compare Regions (12:37,403,821-37,410,688:1) View Sequence Alignments	n/a	34.61 %	41.42 %	n/a	**82.09**	No
	Glycine max	1-to-manyView Gene Tree	GLYMA_13G287000 Compare Regions (13:38,736,311-38,743,172:-1) View Sequence Alignments	n/a	34.91 %	41.55 %	n/a	**85.17**	No
At1g03380	Glycine max	1-to-manyView Gene Tree	GLYMA_03G148700 Compare Regions (3:36,433,817-36,440,498:-1) View Sequence Alignments	0.21122	51.38 %	52.45 %	n/a	**84.8**	Yes
	Glycine max	1-to-manyView Gene Tree	GLYMA_19G152000 Compare Regions (19:41,245,379-41,252,443:-1) View Sequence Alignments	0.21538	54.62 %	49.95 %	n/a	**86.95**	Yes
At1g54710	Glycine max	1-to-manyView Gene Tree	GLYMA_10G157700 Compare Regions (10:39,177,037-39,184,196:1) View Sequence Alignments	n/a	53.85 %	52.86 %	n/a	**92.88**	No
	Glycine max	1-to-manyView Gene Tree	GLYMA_20G230900 Compare Regions (20:46,480,639-46,484,770:-1) View Sequence Alignments	n/a	60.22 %	47.36 %	n/a	**91.76**	No
AT5G66930	Glycine max	1-to-1View Gene Tree	GLYMA_17G180900 Compare Regions (17:20,745,714-20,749,941:1) View Sequence Alignments	n/a	72.94 %	63.35 %	n/a	**93.46**	No

D.HMMER analysis in *A. thaliana, P. vulgaris, M. truncatula,and G.max*.

ID Arabidopsis	***Target***	***Species***	***E-value***
AT1G49180.1	PHAVU_010G120500g	Phaseolus vulgaris	2.10E-71
AT1G49180.2	PHAVU_010G120500g	Phaseolus vulgaris	1.60E-71
AT2G37840.1	PHAVU_010G015100g	Phaseolus vulgaris	5.70E-211
AT2G37840.2	PHAVU_010G015100g	Phaseolus vulgaris	2.10E-162
AT2G37840.3	PHAVU_010G015100g	Phaseolus vulgaris	2.30E-104
AT3G53930.1	PHAVU_010G015100g	Phaseolus vulgaris	4.50E-194
AT3G53930.2	PHAVU_010G015100g	Phaseolus vulgaris	6.20E-196
AT3G53930.3	PHAVU_010G015100g	Phaseolus vulgaris	1.10E-132
AT3G53930.4	PHAVU_010G015100g	Phaseolus vulgaris	1.60E-134
AT3G53930.5	PHAVU_010G015100g	Phaseolus vulgaris	1.10E-132
AT3G61960.1	PHAVU_010G120500g	Phaseolus vulgaris	3.90E-180
AT3G61960.2	PHAVU_010G120500g	Phaseolus vulgaris	5.40E-163
AT3G19190.1	PHAVU_003G295800g	Phaseolus vulgaris	0.00E+00
AT3G19190.2	PHAVU_003G295800g	Phaseolus vulgaris	0.00E+00
AT3G19190.3	PHAVU_003G295800g	Phaseolus vulgaris	0.00E+00
AT5G61500.1	PHAVU_011G006500g	Phaseolus vulgaris	2.90E-184
AT5G61500.2	PHAVU_011G006500g	Phaseolus vulgaris	4.20E-132
AT2G44140.1	PHAVU_008G048900g	Phaseolus vulgaris	1.40E-184
AT2G44140.2	PHAVU_008G048900g	Phaseolus vulgaris	4.30E-171
AT2G44140.3	PHAVU_008G048900g	Phaseolus vulgaris	1.40E-184
AT2G44140.4	PHAVU_008G048900g	Phaseolus vulgaris	6.50E-166

AT2G44140.5	PHAVU_008G048900g	Phaseolus vulgaris	1.40E-151
AT3G59950.1	PHAVU_008G048900g	Phaseolus vulgaris	2.30E-177
AT3G59950.2	PHAVU_008G048900g	Phaseolus vulgaris	1.40E-114
AT3G59950.3	PHAVU_008G048900g	Phaseolus vulgaris	1.80E-105
AT3G59950.4	PHAVU_008G048900g	Phaseolus vulgaris	7.10E-167
AT3G59950.5	PHAVU_008G048900g	Phaseolus vulgaris	1.60E-147
AT5G17290.1	PHAVU_008G241000g	Phaseolus vulgaris	5.70E-144
AT3G61710.1	PHAVU_005G029900g	Phaseolus vulgaris	1.20E-250
AT3G61710.2	PHAVU_005G029900g	Phaseolus vulgaris	1.10E-171
AT3G61710.3	PHAVU_005G029900g	Phaseolus vulgaris	3.80E-139
AT3G61710.4	PHAVU_005G029900g	Phaseolus vulgaris	4.10E-216
AT5G45900.1	PHAVU_011G010700g	Phaseolus vulgaris	0.00E+00
AT4G21980.1	PHAVU_011G103300g	Phaseolus vulgaris	1.80E-60
AT4G21980.2	PHAVU_011G103300g	Phaseolus vulgaris	3.70E-60
AT4G04620.1	PHAVU_003G079300g	Phaseolus vulgaris	7.90E-59
AT4G04620.2	PHAVU_003G079300g	Phaseolus vulgaris	7.90E-59
AT4G04620.3	PHAVU_003G079300g	Phaseolus vulgaris	7.90E-59
AT1G62040.1	PHAVU_011G103300g	Phaseolus vulgaris	2.80E-66
AT1G62040.2	PHAVU_011G103300g	Phaseolus vulgaris	5.40E-66
AT2G05630.1	PHAVU_011G103300g	Phaseolus vulgaris	4.00E-67
AT2G05630.2	PHAVU_011G103300g	Phaseolus vulgaris	3.60E-60
AT2G45170.1	PHAVU_003G219600g	Phaseolus vulgaris	4.10E-60
AT2G45170.2	PHAVU_003G219600g	Phaseolus vulgaris	4.10E-60
AT4G16520.1	PHAVU_003G219600g	Phaseolus vulgaris	1.90E-65
AT4G16520.2	PHAVU_003G219600g	Phaseolus vulgaris	1.90E-65
AT4G16520.3	PHAVU_003G219600g	Phaseolus vulgaris	1.90E-65
AT3G60640.1	PHAVU_003G219600g	Phaseolus vulgaris	1.90E-60
AT3G06420.1	PHAVU_007G210800g	Phaseolus vulgaris	1.10E-30
AT3G15580.1	PHAVU_011G151600g	Phaseolus vulgaris	2.20E-39
AT2G31260.1	PHAVU_001G159900g	Phaseolus vulgaris	0.00E+00
AT3G07525.1	PHAVU_010G036300g	Phaseolus vulgaris	4.80E-68
AT3G07525.2	PHAVU_010G036300g	Phaseolus vulgaris	6.20E-67
AT4G30790.1	PHAVU_003G153800g	Phaseolus vulgaris	0.00E+00
AT1G54210.1	PHAVU_010G130300g	Phaseolus vulgaris	4.40E-50
AT1G54210.2	PHAVU_010G130300g	Phaseolus vulgaris	4.40E-50
AT1G54210.3	PHAVU_010G130300g	Phaseolus vulgaris	4.40E-50
AT3G13970.1	PHAVU_010G130300g	Phaseolus vulgaris	9.90E-49
AT3G13970.2	PHAVU_010G130300g	Phaseolus vulgaris	1.40E-20
AT3G13970.3	PHAVU_010G130300g	Phaseolus vulgaris	1.40E-20
AT3G13970.4	PHAVU_010G130300g	Phaseolus vulgaris	7.00E-34
AT3G49590.1	PHAVU_008G187800g	Phaseolus vulgaris	7.30E-157

AT3G49590.2	PHAVU_008G187800g	Phaseolus vulgaris	7.30E-157
AT3G49590.3	PHAVU_008G187800g	Phaseolus vulgaris	7.30E-157
AT3G18770.1	PHAVU_002G269600g	Phaseolus vulgaris	3.90E-173
AT1G77890.1	PHAVU_008G169200g	Phaseolus vulgaris	1.10E-125
AT1G77890.2	PHAVU_008G169200g	Phaseolus vulgaris	2.80E-116
AT1G77890.3	PHAVU_008G169200g	Phaseolus vulgaris	8.20E-124
AT4G08540.1	PHAVU_008G169200g	Phaseolus vulgaris	2.80E-222
AT5G50230.1	PHAVU_003G207100g	Phaseolus vulgaris	6.50E-257
AT4G30510.1	PHAVU_003G152800g	Phaseolus vulgaris	2.10E-132
AT4G30510.2	PHAVU_003G152800g	Phaseolus vulgaris	5.90E-112
AT3G62770.1	PHAVU_007G196400g	Phaseolus vulgaris	6.80E-186
AT3G62770.3	PHAVU_007G196400g	Phaseolus vulgaris	6.80E-186
AT2G40810.1	PHAVU_009G041700g	Phaseolus vulgaris	1.20E-183
AT2G40810.2	PHAVU_009G041700g	Phaseolus vulgaris	1.20E-183
AT2G40810.3	PHAVU_009G041700g	Phaseolus vulgaris	5.00E-104
AT3G56440.1	PHAVU_009G041700g	Phaseolus vulgaris	2.00E-179
AT3G56440.2	PHAVU_009G041700g	Phaseolus vulgaris	6.90E-166
AT3G56440.3	PHAVU_009G041700g	Phaseolus vulgaris	1.80E-148
AT5G05150.1	PHAVU_009G041700g	Phaseolus vulgaris	3.00E-110
AT5G54730.1	PHAVU_005G091300g	Phaseolus vulgaris	2.60E-160
AT5G54730.2	PHAVU_005G091300g	Phaseolus vulgaris	2.60E-160
AT1G03380.1	PHAVU_001G146700g	Phaseolus vulgaris	1.00E-297
AT1G54710.1	PHAVU_007G183100g	Phaseolus vulgaris	1.00E-302
AT1G54710.2	PHAVU_007G183100g	Phaseolus vulgaris	5.80E-252
AT5G66930.1	PHAVU_003G248000g	Phaseolus vulgaris	2.30E-70
AT5G66930.2	PHAVU_003G248000g	Phaseolus vulgaris	1.70E-106
AT5G66930.3	PHAVU_003G248000g	Phaseolus vulgaris	1.90E-98

ID Arabidopsis	***Target***	***Species***	***E-value***
AT1G49180.1	MTR_3g095620	Medicago truncatula	1.70E-94
AT1G49180.2	MTR_3g095620	Medicago truncatula	1.70E-94
AT2G37840.1	MTR_4g019410	Medicago truncatula	2.20E-284
AT2G37840.2	MTR_4g019410	Medicago truncatula	9.60E-195
AT2G37840.3	MTR_4g019410	Medicago truncatula	2.90E-126
AT3G53930.1	MTR_4g019410	Medicago truncatula	1.20E-260
AT3G53930.2	MTR_4g019410	Medicago truncatula	1.20E-262
AT3G53930.3	MTR_4g019410	Medicago truncatula	2.50E-158
AT3G53930.4	MTR_4g019410	Medicago truncatula	2.20E-160
AT3G53930.5	MTR_4g019410	Medicago truncatula	2.50E-158
AT3G61960.1	MTR_8g024100	Medicago truncatula	1.40E-184
AT3G61960.2	MTR_8g024100	Medicago truncatula	2.10E-170

AT3G19190.1	MTR_4g086370	Medicago truncatula	0.00E+00
AT3G19190.2	MTR_4g086370	Medicago truncatula	0.00E+00
AT3G19190.3	MTR_4g086370	Medicago truncatula	0.00E+00
AT5G61500.1	MTR_4g036265	Medicago truncatula	5.00E-181
AT5G61500.2	MTR_4g036265	Medicago truncatula	4.60E-129
AT2G44140.1	MTR_7g081230	Medicago truncatula	6.90E-186
AT2G44140.2	MTR_7g081230	Medicago truncatula	9.70E-172
AT2G44140.3	MTR_7g081230	Medicago truncatula	6.90E-186
AT2G44140.4	MTR_7g081230	Medicago truncatula	1.50E-166
AT2G44140.5	MTR_7g081230	Medicago truncatula	1.50E-152
AT3G59950.1	MTR_7g081230	Medicago truncatula	3.30E-176
AT3G59950.2	MTR_7g081230	Medicago truncatula	4.80E-113
AT3G59950.3	MTR_7g081230	Medicago truncatula	4.60E-104
AT3G59950.4	MTR_7g081230	Medicago truncatula	8.30E-166
AT3G59950.5	MTR_7g081230	Medicago truncatula	2.40E-146
AT5G17290.1	MTR_5g076920	Medicago truncatula	7.40E-138
AT3G61710.1	MTR_3g018770	Medicago truncatula	1.00E-258
AT3G61710.2	MTR_3g018770	Medicago truncatula	1.50E-175
AT3G61710.3	MTR_3g018770	Medicago truncatula	3.80E-144
AT3G61710.4	MTR_3g018770	Medicago truncatula	3.20E-218
AT5G45900.1	MTR_0003s0540	Medicago truncatula	0.00E+00
AT4G21980.1	MTR_2g023430	Medicago truncatula	2.90E-62
AT4G21980.2	MTR_2g023430	Medicago truncatula	5.90E-62
AT4G04620.1	MTR_2g023430	Medicago truncatula	2.30E-60
AT4G04620.2	MTR_2g023430	Medicago truncatula	2.30E-60
AT4G04620.3	MTR_2g023430	Medicago truncatula	2.30E-60
AT1G62040.1	MTR_4g048510	Medicago truncatula	6.10E-65
AT1G62040.2	MTR_4g048510	Medicago truncatula	1.10E-64
AT2G05630.1	MTR_4g048510	Medicago truncatula	2.70E-66
AT2G05630.2	MTR_4g048510	Medicago truncatula	2.00E-59
AT2G45170.1	MTR_4g101090	Medicago truncatula	4.70E-60
AT2G45170.2	MTR_4g101090	Medicago truncatula	4.70E-60
AT4G16520.1	MTR_4g101090	Medicago truncatula	2.60E-66
AT4G16520.2	MTR_4g101090	Medicago truncatula	2.60E-66
AT4G16520.3	MTR_4g101090	Medicago truncatula	2.60E-66
AT3G60640.1	MTR_4g101090	Medicago truncatula	9.30E-61
AT3G06420.1	MTR_4g123760	Medicago truncatula	5.90E-31
AT3G15580.1	MTR_4g123760	Medicago truncatula	3.40E-52
AT2G31260.1	MTR_1g070160	Medicago truncatula	0.00E+00
AT3G07525.1	MTR_8g010140	Medicago truncatula	3.50E-69
AT3G07525.2	MTR_8g010140	Medicago truncatula	2.20E-68

AT4G30790.1	MTR_4g130370	Medicago truncatula	0.00E+00
AT1G54210.1	MTR_8g020500	Medicago truncatula	7.40E-51
AT1G54210.2	MTR_8g020500	Medicago truncatula	7.40E-51
AT1G54210.3	MTR_8g020500	Medicago truncatula	7.40E-51
AT3G13970.1	MTR_8g020500	Medicago truncatula	7.40E-51
AT3G13970.2	MTR_8g020500	Medicago truncatula	6.50E-21
AT3G13970.3	MTR_8g020500	Medicago truncatula	6.50E-21
AT3G13970.4	MTR_8g020500	Medicago truncatula	8.10E-35
AT3G49590.1	MTR_5g068710	Medicago truncatula	1.30E-148
AT3G49590.2	MTR_5g068710	Medicago truncatula	1.30E-148
AT3G49590.3	MTR_5g068710	Medicago truncatula	1.30E-148
AT3G18770.1	MTR_3g095570	Medicago truncatula	4.50E-154
AT1G77890.1	MTR_5g061040	Medicago truncatula	4.80E-127
AT1G77890.2	MTR_5g061040	Medicago truncatula	1.30E-117
AT1G77890.3	MTR_5g061040	Medicago truncatula	3.80E-125
AT4G08540.1	MTR_5g061040	Medicago truncatula	3.00E-222
AT5G50230.1	MTR_4g104380	Medicago truncatula	4.40E-247
AT4G30510.1	MTR_4g130190	Medicago truncatula	2.40E-136
AT4G30510.2	MTR_4g130190	Medicago truncatula	1.80E-116
AT3G62770.1	MTR_1g083230	Medicago truncatula	6.00E-189
AT3G62770.3	MTR_1g083230	Medicago truncatula	6.00E-189
AT2G40810.1	MTR_3g093590	Medicago truncatula	4.10E-191
AT2G40810.2	MTR_3g093590	Medicago truncatula	4.10E-191
AT2G40810.3	MTR_3g093590	Medicago truncatula	7.70E-109
AT3G56440.1	MTR_3g093590	Medicago truncatula	9.10E-193
AT3G56440.2	MTR_3g093590	Medicago truncatula	5.20E-179
AT3G56440.3	MTR_3g093590	Medicago truncatula	4.30E-158
AT5G05150.1	MTR_3g093590	Medicago truncatula	1.40E-112
AT5G54730.1	MTR_2g082770	Medicago truncatula	4.30E-174
AT5G54730.2	MTR_2g082770	Medicago truncatula	4.30E-174
AT1G03380.1	MTR_1g089110	Medicago truncatula	6.40E-295
AT1G54710.1	MTR_1g082300	Medicago truncatula	7.90E-295
AT1G54710.2	MTR_1g082300	Medicago truncatula	6.50E-246
AT5G66930.1	MTR_8g079240	Medicago truncatula	3.80E-71
AT5G66930.2	MTR_8g079240	Medicago truncatula	3.80E-106
AT5G66930.3	MTR_8g079240	Medicago truncatula	1.00E-98

ID Arabidopsis	Target	Species	E-value
AT1G49180.1	GLYMA_04G215500	Glycine max	1.10E-95
AT1G49180.2	GLYMA_04G215500	Glycine max	6.20E-102
AT2G37840.1	GLYMA_03G069800	Glycine max	1.20E-288
AT2G37840.2	GLYMA_03G069800	Glycine max	3.90E-202
AT2G37840.3	GLYMA_03G069800	Glycine max	1.30E-130
AT3G53930.1	GLYMA_03G069800	Glycine max	2.10E-269
AT3G53930.2	GLYMA_03G069800	Glycine max	2.40E-271
AT3G53930.3	GLYMA_03G069800	Glycine max	1.20E-169
AT3G53930.4	GLYMA_03G069800	Glycine max	3.90E-171
AT3G53930.5	GLYMA_03G069800	Glycine max	1.20E-169
AT3G61960.1	GLYMA_07G048400	Glycine max	4.00E-195
AT3G61960.2	GLYMA_07G048400	Glycine max	1.30E-178
AT3G19190.1	GLYMA_02G133400	Glycine max	0.00E+00
AT3G19190.2	GLYMA_02G133400	Glycine max	0.00E+00
AT3G19190.3	GLYMA_02G133400	Glycine max	0.00E+00
AT5G61500.1	GLYMA_12G005700	Glycine max	1.20E-185
AT5G61500.2	GLYMA_12G005700	Glycine max	1.70E-133
AT2G44140.1	GLYMA_09G244800	Glycine max	1.70E-186
AT2G44140.2	GLYMA_09G244800	Glycine max	2.80E-171
AT2G44140.3	GLYMA_09G244800	Glycine max	1.70E-186
AT2G44140.4	GLYMA_09G244800	Glycine max	2.10E-166
AT2G44140.5	GLYMA_09G244800	Glycine max	7.60E-153
AT3G59950.1	GLYMA_09G244800	Glycine max	2.10E-176
AT3G59950.2	GLYMA_18G248400	Glycine max	2.60E-113
AT3G59950.3	GLYMA_18G248400	Glycine max	3.70E-105
AT3G59950.4	GLYMA_09G244800	Glycine max	2.60E-166
AT3G59950.5	GLYMA_18G248400	Glycine max	1.30E-146
AT5G17290.1	GLYMA_14G210200	Glycine max	5.70E-146
AT3G61710.1	GLYMA_11G153900	Glycine max	4.00E-258
AT3G61710.2	GLYMA_11G153900	Glycine max	2.20E-173
AT3G61710.3	GLYMA_11G153900	Glycine max	1.70E-141
AT3G61710.4	GLYMA_11G153900	Glycine max	8.90E-218
AT5G45900.1	GLYMA_12G010000	Glycine max	0.00E+00
AT4G21980.1	GLYMA_09G003900	Glycine max	1.40E-62
AT4G21980.2	GLYMA_07G261000	Glycine max	8.60E-64
AT4G04620.1	GLYMA_09G003900	Glycine max	4.50E-60
AT4G04620.2	GLYMA_09G003900	Glycine max	4.50E-60
AT4G04620.3	GLYMA_09G003900	Glycine max	4.50E-60
AT1G62040.1	GLYMA_12G098400	Glycine max	1.10E-66
AT1G62040.2	GLYMA_12G098400	Glycine max	2.10E-66

AT2G05630.1	GLYMA_12G098400	Glycine max	1.10E-66
AT2G05630.2	GLYMA_12G098400	Glycine max	9.10E-60
AT2G45170.1	GLYMA_17G140700	Glycine max	1.50E-60
AT2G45170.2	GLYMA_17G140700	Glycine max	1.50E-60
AT4G16520.1	GLYMA_17G140700	Glycine max	8.10E-67
AT4G16520.2	GLYMA_17G140700	Glycine max	8.10E-67
AT4G16520.3	GLYMA_17G140700	Glycine max	8.10E-67
AT3G60640.1	GLYMA_17G140700	Glycine max	7.60E-61
AT3G06420.1	GLYMA_02G008800	Glycine max	6.40E-31
AT3G15580.1	GLYMA_02G008800	Glycine max	6.20E-51
AT2G31260.1	GLYMA_10G035800	Glycine max	0.00E+00
AT3G07525.1	GLYMA_07G124300	Glycine max	5.20E-68
AT3G07525.2	GLYMA_03G097000	Glycine max	4.60E-67
AT4G30790.1	GLYMA_17G071400	Glycine max	0.00E+00
AT1G54210.1	GLYMA_07G038100	Glycine max	9.70E-50
AT1G54210.2	GLYMA_07G038100	Glycine max	9.70E-50
AT1G54210.3	GLYMA_07G038100	Glycine max	9.70E-50
AT3G13970.1	GLYMA_07G038100	Glycine max	3.70E-48
AT3G13970.2	GLYMA_16G007300	Glycine max	3.70E-20
AT3G13970.3	GLYMA_16G007300	Glycine max	3.70E-20
AT3G13970.4	GLYMA_16G007300	Glycine max	2.10E-33
AT3G49590.1	GLYMA_14G187000	Glycine max	2.90E-160
AT3G49590.2	GLYMA_14G187000	Glycine max	2.90E-160
AT3G49590.3	GLYMA_14G187000	Glycine max	2.90E-160
AT3G18770.1	GLYMA_05G189000	Glycine max	8.40E-182
AT1G77890.1	GLYMA_14G167200	Glycine max	6.30E-127
AT1G77890.2	GLYMA_13G085400	Glycine max	1.20E-116
AT1G77890.3	GLYMA_14G167200	Glycine max	4.90E-125
AT4G08540.1	GLYMA_14G167200	Glycine max	8.70E-183
AT5G50230.1	GLYMA_05G043700	Glycine max	2.00E-256
AT4G30510.1	GLYMA_17G070200	Glycine max	2.40E-140
AT4G30510.2	GLYMA_17G070200	Glycine max	2.40E-140
AT3G62770.1	GLYMA_03G212100	Glycine max	7.00E-186
AT3G62770.3	GLYMA_03G212100	Glycine max	7.00E-186
AT2G40810.1	GLYMA_06G140400	Glycine max	8.60E-194
AT2G40810.2	GLYMA_06G140400	Glycine max	8.60E-194
AT2G40810.3	GLYMA_06G140400	Glycine max	2.70E-113
AT3G56440.1	GLYMA_06G140400	Glycine max	1.60E-19
AT3G56440.2	GLYMA_06G140400	Glycine max	9.40E-179
AT3G56440.3	GLYMA_06G140400	Glycine max	1.00E-157
AT5G05150.1	GLYMA_06G140400	Glycine max	3.20E-114

AT5G54730.1	GLYMA_13G287000	Glycine max	7.60E-172
AT5G54730.2	GLYMA_13G287000	Glycine max	7.60E-172
AT1G03380.1	GLYMA_03G148700	Glycine max	8.10E-306
AT1G54710.1	GLYMA_10G157700	Glycine max	1.60E-296
AT1G54710.2	GLYMA_10G157700	Glycine max	1.60E-296
AT5G66930.1	GLYMA_17G180900	Glycine max	7.70E-72
AT5G66930.2	GLYMA_17G180900	Glycine max	7.20E-108
AT5G66930.3	GLYMA_17G180900	Glycine max	8.80E-100

E. Inparanoid

ID Arabidopsis	***Homolog Gene . Primary Identifier***	***Gene Symbol***	***Gene Organism Name***	***Homolog Ortholog _ Gene . Primary Identifier***	***Ortholog _ Gene Organism Name***	***Homolog Relationship***	***Homolog Group Name***
At1g49180	Species without orthologues						
At2g37840	AT2G37840		A. thaliana	Phvul.010G015100	P. vulgaris	many-to-one	442_167_2102
	AT2G37840		A. thaliana early-release	Phvul.010G015100	P. vulgaris	many-to-one	442_447_2103
At3g53930	AT3G53930		A. thaliana	Phvul.010G015100	P. vulgaris	many-to-one	442_167_2102
	AT3G53930		A. thaliana early-release	Phvul.010G015100	P. vulgaris	many-to-one	442_447_2103
At3g61960	AT3G61960		A. thaliana	Phvul.010G120500	P. vulgaris	one-to-one	442_167_4678
	AT3G61960		A. thaliana early-release	Phvul.010G120500	P. vulgaris	one-to-one	442_447_4677
At3g19190	AT3G19190	ATG2	A. thaliana	Phvul.003G295800	P. vulgaris	one-to-one	442_167_397
	AT3G19190	ATG2	A. thaliana early-release	Phvul.003G295800	P. vulgaris	one-to-one	442_447_310
At5g61500	AT5G61500	ATG3	A. thaliana	Phvul.011G006500	P. vulgaris	one-to-one	442_167_4852
	AT5G61500	ATG3	A. thaliana early-release	Phvul.011G006500	P. vulgaris	one-to-one	442_447_4852
At2g44140	AT2G44140		A. thaliana	Phvul.008G048900	P. vulgaris	many-to-one	442_167_4694
	AT2G44140		A. thaliana early-release	Phvul.008G048900	P. vulgaris	many-to-one	442_447_4694
At3g59950	AT3G59950		A. thaliana	Phvul.008G048900	P. vulgaris	many-to-one	442_167_4694
	AT3G59950		A. thaliana early-release	Phvul.008G048900	P. vulgaris	many-to-one	442_447_4694
At5g17290	AT5G17290	APG5	A. thaliana	Phvul.008G241000	P. vulgaris	one-to-one	442_167_6496
	AT5G17290	APG5	A. thaliana early-release	Phvul.008G241000	P. vulgaris	one-to-one	442_447_6506
At3g61710		ATG6	A. thaliana	Phvul.005G029900	P. vulgaris	one-to-one	442_167_2769
	AT3G61710	ATG6	A. thaliana early-release	Phvul.005G029900	P. vulgaris	one-to-one	442_447_2778
At5g45900	AT5G45900	APG7	A. thaliana	Phvul.011G010700	P. vulgaris	one-to-one	442_167_1340
	AT5G45900	APG7	A. thaliana early-release	Phvul.011G010700	P. vulgaris	one-to-one	442_447_1344
At4g21980	Species without orthologues						
At4g04620	Species without orthologues						
At1g62040	AT1G62040	ATG8C	A. thaliana	Phvul.011G103300	P. vulgaris	one-to-one	442_167_10280
	AT1G62040	ATG8C	A. thaliana early-release	Phvul.011G103300	P. vulgaris	one-to-one	442_447_10288
At2g05630	Species without orthologues						
At2g45170	AT2G45170	ATG8E	A. thaliana early-release	Phvul.002G062200	P. vulgaris	many-to-many	442_447_10594
	AT2G45170	ATG8E	A. thaliana early-release	Phvul.003G219600	P. vulgaris	many-to-many	442_447_10594
At4g16520	AT4G16520	ATG8F	A. thaliana	Phvul.002G062200	P. vulgaris	one-to-many	442_167_10328

	AT4G16520	ATG8F	A. thaliana	Phvul.003G219600	P. vulgaris	one-to-many	442_167_10328
At3g60640	AT3G60640	ATG8G	A. thaliana early-release	Phvul.002G062200	P. vulgaris	many-to-many	442_447_10594
	AT3G60640	ATG8G	A. thaliana early-release	Phvul.003G219600	P. vulgaris	many-to-many	442_447_10594
At3g06420	Species without orthologues						
At3g15580	AT3G15580	APG8H	A. thaliana	Phvul.007G210800	P. vulgaris	one-to-one	442_167_11037
	AT3G15580	APG8H	A. thaliana early-release	Phvul.007G210800	P. vulgaris	one-to-one	442_447_11046
At2g31260	AT2G31260	APG9	A. thaliana	Phvul.001G159900	P. vulgaris	one-to-one	442_167_1012
	AT2G31260	APG9	A. thaliana early-release	Phvul.001G159900	P. vulgaris	one-to-one	442_447_1023
At3g07525	AT3G07525	ATG10	A. thaliana	Phvul.010G036300	P. vulgaris	one-to-one	442_167_10197
	AT3G07525	ATG10	A. thaliana early-release	Phvul.010G036300	P. vulgaris	one-to-one	442_447_10203
AT4G30790	AT4G30790		A. thaliana	Phvul.003G153800	P. vulgaris	one-to-one	442_167_523
	AT4G30790		A. thaliana early-release	Phvul.003G153800	P. vulgaris	one-to-one	442_447_528
At1g54210	AT1G54210	ATG12A	A. thaliana	Phvul.010G130300	P. vulgaris	many-to-one	442_167_11107
	AT1G54210	ATG12A	A. thaliana early-release	Phvul.010G130300	P. vulgaris	many-to-one	442_447_11118
At3g13970	AT3G13970	APG12B	A. thaliana	Phvul.010G130300	P. vulgaris	many-to-one	442_167_11107
	AT3G13970	APG12B	A. thaliana early-release	Phvul.010G130300	P. vulgaris	many-to-one	442_447_11118
At3g49590	AT3G49590	ATG13	A. thaliana early-release	Phvul.008G187800	P. vulgaris	one-to-one	442_447_5435
	AT3G49590		A. thaliana	Phvul.008G187800	P. vulgaris	one-to-one	442_167_5430
At3g18770	AT3G18770		A. thaliana	Phvul.002G269600	P. vulgaris	one-to-one	442_167_4855
	AT3G18770		A. thaliana early-release	Phvul.002G269600	P. vulgaris	one-to-one	442_447_4855
AT4g08540	AT4G08540		A. thaliana	Phvul.008G169200	P. vulgaris	one-to-one	442_167_3451
At5g50230	AT5G50230		A. thaliana	Phvul.003G207100	P. vulgaris	one-to-one	442_167_2508
	AT5G50230		A. thaliana early-release	Phvul.003G207100	P. vulgaris	one-to-one	442_447_2513
At3g62770	Species without orthologues						
At4g30510	AT4G30510	ATG18B	A. thaliana	Phvul.003G152800	P. vulgaris	one-to-one	442_167_7006
	AT4G30510	ATG18B	A. thaliana early-release	Phvul.003G152800	P. vulgaris	one-to-one	442_447_6033
At2g40810	AT2G40810	ATG18C	A. thaliana	Phvul.009G041700	P. vulgaris	many-to-one	442_167_4857
	AT2G40810	ATG18C	A. thaliana early-release	Phvul.009G041700	P. vulgaris	many-to-one	442_447_4857
At3g56440	AT3G56440	ATG18D	A. thaliana	Phvul.009G041700	P. vulgaris	many-to-one	442_167_4857
	AT3G56440	ATG18D	A. thaliana early-release	Phvul.009G041700	P. vulgaris	many-to-one	442_447_4857
At5g05150	Species without orthologues						
At5g54730	AT5G54730	G18F	A. thaliana	Phvul.005G091300	P. vulgaris	one-to-many	442_167_5446
	AT5G54730	G18F	A. thaliana	Phvul.011G140900	P. vulgaris	one-to-many	442_167_5446
	AT5G54730	G18F	A. thaliana early-release	Phvul.005G091300	P. vulgaris	one-to-many	442_447_5452
	AT5G54730	G18F	A. thaliana early-release	Phvul.011G140900	P. vulgaris	one-to-many	442_447_5452
At1g03380	AT1G03380	ATG18G	A. thaliana	Phvul.001G146700	P. vulgaris	one-to-one	442_167_1655
	AT1G03380	ATG18G	A. thaliana early-release	Phvul.001G146700	P. vulgaris	one-to-one	442_447_1658
At1g54710	AT1G54710	ATG18H	A. thaliana	Phvul.007G183100	P. vulgaris	one-to-one	442_167_1517
	AT1G54710	ATG18H	A. thaliana early-release	Phvul.007G183100	P. vulgaris	one-to-one	442_447_1524
AT5G66930	AT5G66930		A. thaliana	Phvul.003G248000	P. vulgaris	one-to-one	442_167_8650
	AT5G66930		A. thaliana early-release	Phvul.003G248000	P. vulgaris	one-to-one	442_447_8655

ID Arabidopsis	Homolog Gene . Primary Identifier	Gene Symbol	Gene Organism Name	Homolog Ortholog _ Gene . Primary Identifier	Ortholog _ Gene Organism Name	Homolog Relationship	Homolog Group Name
At1g49180	AT1G49180		A. thaliana	Medtr3g095620	M. truncatula	one-to-one	167_285_8624
At2g37840	AT2G37840		A. thaliana	Medtr4g019410	M. truncatula	many-to-one	167_285_1975
	AT2G37840		A. thaliana early-release	Medtr4g019410	M. truncatula	many-to-one	285_447_1979
At3g53930	AT3G53930		A. thaliana	Medtr4g019410	M. truncatula	many-to-one	167_285_1975
	AT3G53930		A. thaliana early-release	Medtr4g019410	M. truncatula	many-to-one	285_447_1979
At3g61960	AT3G61960		A. thaliana	Medtr8g024100	M. truncatula	one-to-one	167_285_4535
	AT3G61960		A. thaliana early-release	Medtr8g024100	M. truncatula	one-to-one	285_447_4536
At3g19190	AT3G19190	ATG2	A. thaliana	Medtr4g086370	M. truncatula	one-to-one	167_285_411
	AT3G19190	ATG2	A. thaliana early-release	Medtr4g086370	M. truncatula	one-to-one	285_447_309
At5g61500	AT5G61500	ATG3	A. thaliana	Medtr4g036265	M. truncatula	one-to-one	167_285_4887
	AT5G61500	ATG3	A. thaliana early-release	Medtr4g036265	M. truncatula	one-to-one	285_447_4890
At2g44140	AT2G44140		A. thaliana	Medtr7g081230	M. truncatula	many-to-one	167_285_4639
	AT2G44140		A. thaliana early-release	Medtr7g081230	M. truncatula	many-to-one	285_447_4647
At3g59950	AT3G59950		A. thaliana	Medtr7g081230	M. truncatula	many-to-one	167_285_4639
	AT3G59950		A. thaliana early-release	Medtr7g081230	M. truncatula	many-to-one	285_447_4647
At5g17290	AT5G17290	APG5	A. thaliana	Medtr5g076920	M. truncatula	one-to-one	167_285_6531
	AT5G17290	APG5	A. thaliana early-release	Medtr5g076920	M. truncatula	one-to-one	285_447_6546
At3g61710	AT3G61710	ATG6	A. thaliana	Medtr3g018770	M. truncatula	one-to-one	167_285_2512
	AT3G61710	ATG6	A. thaliana early-release	Medtr3g018770	M. truncatula	one-to-one	285_447_2523
At5g45900	AT5G45900	APG7	A. thaliana	Medtr0003s0540	M. truncatula	one-to-one	167_285_1357
	AT5G45900	APG7	A. thaliana early-release	Medtr0003s0540	M. truncatula	one-to-one	285_447_1362
At4g21980	AT4G21980	APG8A	A. thaliana	Medtr2g023430	M. truncatula	many-to-one	167_285_10350
	AT4G21980	APG8A	A. thaliana early-release	Medtr2g023430	M. truncatula	many-to-one	285_447_10362
At4g04620	AT4G04620	ATG8B	A. thaliana	Medtr2g023430	M. truncatula	many-to-one	167_285_10350
	AT4G04620	ATG8B	A. thaliana early-release	Medtr2g023430	M. truncatula	many-to-one	285_447_10362
At1g62040	AT1G62040	ATG8C	A. thaliana	Medtr4g048510	M. truncatula	one-to-one	167_285_10219
	AT1G62040	ATG8C	A. thaliana early-release	Medtr4g048510	M. truncatula	one-to-one	285_447_10225
At2g05630	Species without orthologues						
At2g45170	Species without orthologues						
At4g16520	AT4G16520	ATG8F	A. thaliana	Medtr4g101090	M. truncatula	one-to-one	167_285_10131
At3g60640	AT3G60640	ATG8G	A. thaliana early-release	Medtr4g101090	M. truncatula	one-to-one	285_447_10455
At3g06420	AT3G06420	ATG8H	A. thaliana	Medtr4g123760	M. truncatula	one-to-one	167_285_10842
	AT3G06420	ATG8H	A. thaliana early-release	Medtr4g123760	M. truncatula	one-to-one	285_447_10851
At3g15580	Species without orthologues						
At2g31260	AT2G31260	APG9	A. thaliana	Medtr1g070160	M. truncatula	one-to-many	167_285_963
	AT2G31260	APG9	A. thaliana	Medtr7g096680	M. truncatula	one-to-many	167_285_963
	AT2G31260	APG9	A. thaliana early-release	Medtr1g070160	M. truncatula	one-to-many	285_447_971
	AT2G31260	APG9	A. thaliana early-release	Medtr7g096680	M. truncatula	one-to-many	285_447_971

	AT2G31260	APG9	A. thaliana	Medtr1g070160	M. truncatula	one-to-many	167_285_963
	AT2G31260	APG9	A. thaliana	Medtr7g096680	M. truncatula	one-to-many	167_285_963
	AT2G31260	APG9	A. thaliana early-release	Medtr1g070160	M. truncatula	one-to-many	285_447_971
	AT2G31260	APG9	A. thaliana early-release	Medtr7g096680	M. truncatula	one-to-many	285_447_971
At3g07525	AT3G07525	ATG10	A. thaliana	Medtr8g010140	M. truncatula	one-to-one	167_285_9971
	AT3G07525	ATG10	A. thaliana early-release	Medtr8g010140	M. truncatula	one-to-one	285_447_9982
AT4G30790	AT4G30790		A. thaliana	Medtr4g130370	M. truncatula	one-to-one	167_285_526
	AT4G30790		A. thaliana early-release	Medtr4g130370	M. truncatula	one-to-one	285_447_528
At1g54210	AT1G54210	ATG12A	A. thaliana	Medtr8g020500	M. truncatula	many-to-one	167_285_11190
	AT1G54210	ATG12A	A. thaliana early-release	Medtr8g020500			
At3g13970	AT3G13970	APG12B	A. thaliana	Medtr8g020500	M. truncatula	many-to-one	167_285_11190
	AT3G13970	APG12B	A. thaliana early-release	Medtr8g020500	M. truncatula	many-to-one	285_447_11201
At3g49590	AT3G49590	ATG13	A. thaliana early-release	Medtr5g068710	M. truncatula	one-to-one	285_447_5668
	AT3G49590		A. thaliana	Medtr5g068710	M. truncatula	one-to-one	167_285_5658
At3g18770	AT3G18770		A. thaliana	Medtr3g095570	M. truncatula	one-to-one	167_285_5417
	AT3G18770		A. thaliana early-release	Medtr3g095570	M. truncatula	one-to-one	285_447_5424
AT4g08540	AT4G08540		A. thaliana	Medtr5g061040	M. truncatula	one-to-many	167_275_3592
At5g50230	AT5G50230		A. thaliana	Medtr3g075400	M. truncatula	one-to-many	167_285_2690
	AT5G50230		A. thaliana	Medtr4g104380	M. truncatula	one-to-many	167_285_2690
	AT5G50230		A. thaliana early-release	Medtr3g075400	M. truncatula	one-to-many	285_447_2698
	AT5G50230		A. thaliana early-release	Medtr4g104380	M. truncatula	one-to-many	285_447_2698
At3g62770	AT3G62770	AtATG18a	A. thaliana	Medtr1g083230	M. truncatula	one-to-one	167_285_4678
	AT3G62770	AtATG18a	A. thaliana early-release	Medtr1g083230	M. truncatula	one-to-one	285_447_4685
At4g30510	AT4G30510	ATG18B	A. thaliana	Medtr4g130190	M. truncatula	one-to-one	167_285_6727
	AT4G30510	ATG18B	A. thaliana early-release	Medtr4g130190	M. truncatula	one-to-one	285_447_5760
At2g40810	AT2G40810	ATG18C	A. thaliana	Medtr3g093590	M. truncatula	many-to-one	167_285_4491
	AT2G40810	ATG18C	A. thaliana early-release	Medtr3g093590	M. truncatula	many-to-one	285_447_4493
At3g56440	AT3G56440	ATG18D	A. thaliana	Medtr3g093590	M. truncatula	many-to-one	167_285_4491
	AT3G56440	ATG18D	A. thaliana early-release	Medtr3g093590	M. truncatula	many-to-one	285_447_4493
At5g05150	Species without orthologues						
At5g54730	AT5G54730	G18F	A. thaliana	Medtr2g082770	M. truncatula	one-to-one	167_285_4885
	AT5G54730	G18F	A. thaliana early-release	Medtr2g082770	M. truncatula	one-to-one	285_447_4888
At1g03380	AT1G03380	ATG18G	A. thaliana	Medtr1g089110	M. truncatula	one-to-one	167_285_1615
	AT1G03380	ATG18G	A. thaliana early-release	Medtr1g089110	M. truncatula	one-to-one	285_447_1623
At1g54710	AT1G54710	ATG18H	A. thaliana	Medtr1g082300	M. truncatula	one-to-one	167_285_1581
	AT1G54710	ATG18H	A. thaliana early-release	Medtr1g082300	M. truncatula	one-to-one	285_447_1588
AT5G66930	AT5G66930		A. thaliana	Medtr8g079240	M. truncatula	one-to-one	167_285_8486
	AT5G66930		A. thaliana early-release	Medtr8g079240	M. truncatula	one-to-one	285_447_8493

ID Arabidopsis	*Homolog Gene . Primary Identifier*	*Gene Symbol*	*Gene Organism Name*	*Homolog Ortholog _ Gene . Primary Identifier*	*Ortholog _ Gene Organism Name*	*Homolog Relationship*	*Homolog Group Name*
AT1G49180	AT1G49180		A. thaliana	Glyma.04G215500	G. max	one-to-many	167_275_8523
	AT1G49180		A. thaliana	Glyma.06G150700	G. max	one-to-many	167_275_8523
At2g37840	AT2G37840		A. thaliana	Glyma.01G099600	G. max	many-to-many	167_275_2008
	AT2G37840		A. thaliana	Glyma.03G069800	G. max	many-to-many	167_275_2008
	AT2G37840		A. thaliana early-release	Glyma.01G099600	G. max	many-to-many	275_447_2016
	AT2G37840		A. thaliana early-release	Glyma.03G069800	G. max	many-to-many	275_447_2016
At3g53930	AT3G53930		A. thaliana	Glyma.01G099600	G. max	many-to-many	167_275_2008
	AT3G53930		A. thaliana	Glyma.03G069800	G. max	many-to-many	167_275_2008
	AT3G53930		A. thaliana early-release	Glyma.01G099600	G. max	many-to-many	275_447_2016
	AT3G53930		A. thaliana early-release	Glyma.03G069800	G. max	many-to-many	275_447_2016
At3g61960	AT3G61960		A. thaliana	Glyma.07G048400	G. max	one-to-many	167_275_4368
	AT3G61960		A. thaliana	Glyma.16G017300	G. max	one-to-many	167_275_4368
	AT3G61960		A. thaliana early-release	Glyma.07G048400	G. max	one-to-many	275_447_4383
	AT3G61960		A. thaliana early-release	Glyma.16G017300	G. max	one-to-many	275_447_4383
At3g19190	AT3G19190	ATG2	A. thaliana	Glyma.02G133400	G. max	one-to-many	167_275_380
	AT3G19190	ATG2	A. thaliana	Glyma.07G211600	G. max	one-to-many	167_275_380
	AT3G19190	ATG2	A. thaliana early-release	Glyma.02G133400	G. max	one-to-many	275_447_300
	AT3G19190	ATG2	A. thaliana early-release	Glyma.07G211600	G. max	one-to-many	275_447_300
At5g61500	AT5G61500	ATG3	A. thaliana	Glyma.12G005700	G. max	one-to-one	167_275_4956
	AT5G61500	ATG3	A. thaliana early-release	Glyma.12G005700	G. max	one-to-one	275_447_4964
At2g44140	AT2G44140		A. thaliana	Glyma.09G244800	G. max	many-to-many	167_275_4802
	AT2G44140		A. thaliana	Glyma.18G248400	G. max	many-to-many	167_275_4802
	AT2G44140		A. thaliana early-release	Glyma.09G244800	G. max	many-to-many	275_447_4808
	AT2G44140		A. thaliana early-release	Glyma.18G248400	G. max	many-to-many	275_447_4808
	AT3G59950		A. thaliana	Glyma.09G244800	G. max	many-to-many	167_275_4802
	AT3G59950		A. thaliana	Glyma.18G248400	G. max	many-to-many	167_275_4802
	AT3G59950		A. thaliana early-release	Glyma.09G244800	G. max	many-to-many	275_447_4808
	AT3G59950		A. thaliana early-release	Glyma.18G248400	G. max	many-to-many	275_447_4808
At3g59950	AT3G59950		A. thaliana	Glyma.09G244800	G. max	many-to-many	167_275_4802
	AT3G59950		A. thaliana	Glyma.18G248400	G. max	many-to-many	167_275_4802
	AT3G59950		A. thaliana early-release	Glyma.09G244800	G. max	many-to-many	275_447_4808
	AT3G59950		A. thaliana early-release	Glyma.18G248400	G. max	many-to-many	275_447_4808
At5g17290	AT5G17290	APG5	A. thaliana	Glyma.02G240700	G. max	one-to-many	167_275_6533
	AT5G17290	APG5	A. thaliana	Glyma.14G210200	G. max	one-to-many	167_275_6533
	AT5G17290	APG5	A. thaliana early-release	Glyma.02G240700	G. max	one-to-many	275_447_6550
	AT5G17290	APG5	A. thaliana early-release	Glyma.14G210200	G. max	one-to-many	275_447_6550
At3g61710	AT3G61710	ATG6	A. thaliana	Glyma.04G141000	G. max	one-to-many	167_275_2659
	AT3G61710	ATG6	A. thaliana	Glyma.11G153900	G. max	one-to-many	167_275_2659
	AT3G61710	ATG6	A. thaliana early-release	Glyma.04G141000	G. max	one-to-many	275_447_2674

	AT3G61710	ATG6	A. thaliana early-release	Glyma.11G153900	G. max	one-to-many	275_447_2674
At5g45900	AT5G45900	APG7	A. thaliana	Glyma.12G010000	G. max	one-to-one	167_275_1374
	AT5G45900	APG7	A. thaliana early-release	Glyma.12G010000	G. max	one-to-one	275_447_1383
At4g21980	AT4G21980	APG8A	A. thaliana	Glyma.07G261000	G. max	many-to-many	167_275_10571
	AT4G21980	APG8A	A. thaliana	Glyma.09G003900	G. max	many-to-many	167_275_10571
	AT4G21980	APG8A	A. thaliana	Glyma.15G108200	G. max	many-to-many	167_275_10571
	AT4G21980	APG8A	A. thaliana	Glyma.17G013000	G. max	many-to-many	167_275_10571
	AT4G21980	APG8A	A. thaliana early-release	Glyma.07G261000	G. max	many-to-many	275_447_10593
	AT4G21980	APG8A	A. thaliana early-release	Glyma.09G003900	G. max	many-to-many	275_447_10593
	AT4G21980	APG8A	A. thaliana early-release	Glyma.15G108200	G. max	many-to-many	275_447_10593
	AT4G21980	APG8A	A. thaliana early-release	Glyma.17G013000	G. max	many-to-many	275_447_10593
At4g04620	AT4G04620	ATG8B	A. thaliana	Glyma.09G003900	G. max	many-to-many	167_275_10571
	AT4G04620	ATG8B	A. thaliana	Glyma.15G108200	G. max	many-to-many	167_275_10571
	AT4G04620	ATG8B	A. thaliana	Glyma.17G013000	G. max	many-to-many	167_275_10571
	AT4G04620	ATG8B	A. thaliana early-release	Glyma.07G261000	G. max	many-to-many	275_447_10593
	AT4G04620	ATG8B	A. thaliana early-release	Glyma.09G003900	G. max	many-to-many	275_447_10593
	AT4G04620	ATG8B	A. thaliana early-release	Glyma.15G108200	G. max	many-to-many	275_447_10593
At1g62040	AT1G62040	ATG8C	A. thaliana	Glyma.06G306300	G. max	one-to-many	167_275_10499
	AT1G62040	ATG8C	A. thaliana	Glyma.12G098400	G. max	one-to-many	167_275_10499
	AT1G62040	ATG8C	A. thaliana early-release	Glyma.06G306300	G. max	one-to-many	275_447_10515
	AT1G62040	ATG8C	A. thaliana early-release	Glyma.12G098400	G. max	one-to-many	275_447_10515
At2g05630	Species without orthologues						
At2g45170	AT2G45170	ATG8E	A. thaliana early-release	Glyma.01G210200	G. max	many-to-many	275_447_10793
	AT2G45170	ATG8E	A. thaliana early-release	Glyma.05G058300	G. max	many-to-many	275_447_10793
	AT2G45170	ATG8E	A. thaliana early-release	Glyma.11G031800	G. max	many-to-many	275_447_10793
	AT2G45170	ATG8E	A. thaliana early-release	Glyma.17G140700	G. max	many-to-many	275_447_10793
At4g16520	AT4G16520	ATG8F	A. thaliana	Glyma.01G210200	G. max	one-to-many	167_275_10466
	AT4G16520	ATG8F	A. thaliana	Glyma.05G058300	G. max	one-to-many	167_275_10466
	AT4G16520	ATG8F	A. thaliana	Glyma.17G140700	G. max	one-to-many	167_275_10466
At3g60640	AT3G60640	ATG8G	A. thaliana early-release	Glyma.01G210200	G. max	many-to-many	275_447_10793
	AT3G60640	ATG8G	A. thaliana early-release	Glyma.05G058300	G. max	many-to-many	275_447_10793
	AT3G60640	ATG8G	A. thaliana early-release	Glyma.11G031800	G. max	many-to-many	275_447_10793
	AT3G60640	ATG8G	A. thaliana early-release	Glyma.17G140700	G. max	many-to-many	275_447_10793
At3g06420	Species without orthologues						
At3g15580	AT3G15580	APG8H	A. thaliana	Glyma.02G008800	G. max	one-to-many	167_275_11253
	AT3G15580	APG8H	A. thaliana	Glyma.10G009300	G. max	one-to-many	167_275_11253
	AT3G15580	APG8H	A. thaliana early-release	Glyma.02G008800	G. max	one-to-many	275_447_11278
	AT3G15580	APG8H	A. thaliana early-release	Glyma.10G009300	G. max	one-to-many	275_447_11278
At2g31260	AT2G31260	APG9	A. thaliana	Glyma.03G162100	G. max	one-to-many	167_275_968
	AT2G31260	APG9	A. thaliana	Glyma.10G035800	G. max	one-to-many	167_275_968
	AT2G31260	APG9	A. thaliana	Glyma.13G122200	G. max	one-to-many	167_275_968

	AT2G31260	APG9	A. thaliana	Glyma.19G163500	G. max	one-to-many	167_275_968
	AT2G31260	APG9	A. thaliana early-release	Glyma.03G162100	G. max	one-to-many	275_447_977
	AT2G31260	APG9	A. thaliana early-release	Glyma.10G035800	G. max	one-to-many	275_447_977
	AT2G31260	APG9	A. thaliana early-release	Glyma.13G122200	G. max	one-to-many	275_447_977
	AT2G31260	APG9	A. thaliana early-release	Glyma.19G163500	G. max	one-to-many	275_447_977
At3g07525	AT3G07525	ATG10	A. thaliana	Glyma.03G097000	G. max	one-to-many	167_275_10381
	AT3G07525	ATG10	A. thaliana	Glyma.07G124300	G. max	one-to-many	167_275_10381
	AT3G07525	ATG10	A. thaliana early-release	Glyma.03G097000	G. max	one-to-many	275_447_10402
	AT3G07525	ATG10	A. thaliana early-release	Glyma.07G124300	G. max	one-to-many	275_447_10402
AT4G30790	AT4G30790		A. thaliana	Glyma.02G206500	G. max	one-to-many	167_275_515
	AT4G30790		A. thaliana	Glyma.17G071400	G. max	one-to-many	167_275_515
	AT4G30790		A. thaliana early-release	Glyma.02G206500	G. max	one-to-many	275_447_519
	AT4G30790		A. thaliana early-release	Glyma.17G071400	G. max	one-to-many	275_447_519
At1g54210	AT1G54210	ATG12A	A. thaliana	Glyma.07G038100	G. max	many-to-one	167_275_11360
	AT1G54210	ATG12A	A. thaliana early-release	Glyma.07G038100	G. max	many-to-one	275_447_11387
At3g13970	AT3G13970	APG12B	A. thaliana	Glyma.07G038100	G. max	many-to-one	167_275_11360
	AT3G13970	APG12B	A. thaliana early-release	Glyma.07G038100	G. max	many-to-one	275_447_11387
At3g49590	AT3G49590	ATG13	A. thaliana early-release	Glyma.02G220700	G. max	one-to-many	275_447_5383
	AT3G49590	ATG13	A. thaliana early-release	Glyma.14G187000	G. max	one-to-many	275_447_5383
	AT3G49590		A. thaliana	Glyma.02G220700	G. max	one-to-many	167_275_5368
	AT3G49590		A. thaliana	Glyma.14G187000	G. max	one-to-many	167_275_5368
At3g18770	AT3G18770		A. thaliana	Glyma.05G189000	G. max	one-to-many	167_275_4665
	AT3G18770		A. thaliana	Glyma.08G146700	G. max	one-to-many	167_275_4665
	AT3G18770		A. thaliana early-release	Glyma.05G189000	G. max	one-to-many	275_447_4677
	AT3G18770		A. thaliana early-release	Glyma.08G146700	G. max	one-to-many	275_447_4677
At4g08540	AT4G08540		A. thaliana	Glyma.13G085400	G. max	one-to-many	167_275_3592
	AT4G08540		A. thaliana	Glyma.14G167200	G. max	one-to-many	167_275_3592
At5g50230	AT5G50230		A. thaliana	Glyma.05G043700	G. max	one-to-many	167_275_2616
	AT5G50230		A. thaliana	Glyma.17G126200	G. max	one-to-many	167_275_2616
	AT5G50230		A. thaliana early-release	Glyma.05G043700	G. max	one-to-many	275_447_2629
	AT5G50230		A. thaliana early-release	Glyma.17G126200	G. max	one-to-many	275_447_2629
At3g62770	AT3G62770	AtATG18a	A. thaliana	Glyma.03G212100	G. max	one-to-many	167_275_4880
	AT3G62770	AtATG18a	A. thaliana	Glyma.19G209200	G. max	one-to-many	167_275_4880
	AT3G62770	AtATG18a	A. thaliana early-release	Glyma.03G212100	G. max	one-to-many	275_447_4884
	AT3G62770	AtATG18a	A. thaliana early-release	Glyma.19G209200	G. max	one-to-many	275_447_4884
At4g30510	AT4G30510	ATG18B	A. thaliana	Glyma.02G207500	G. max	one-to-many	167_275_6877
	AT4G30510	ATG18B	A. thaliana	Glyma.17G070200	G. max	one-to-many	167_275_6877
	AT4G30510	ATG18B	A. thaliana early-release	Glyma.02G207500	G. max	one-to-many	275_447_5899
	AT4G30510	ATG18B	A. thaliana early-release	Glyma.17G070200	G. max	one-to-many	275_447_5899
At2g40810	AT2G40810	ATG18C	A. thaliana	Glyma.04G224300	G. max	many-to-many	167_275_4638
	AT2G40810	ATG18C	A. thaliana	Glyma.06G140400	G. max	many-to-many	167_275_4638

	AT2G40810	ATG18C	A. thaliana early-release	Glyma.04G224300	G. max	many-to-many	275_447_4651
At3g56440	AT3G56440	ATG18D	A. thaliana	Glyma.04G224300	G. max	many-to-many	167_275_4638
	AT3G56440	ATG18D	A. thaliana	Glyma.06G140400	G. max	many-to-many	167_275_4638
	AT3G56440	ATG18D	A. thaliana early-release	Glyma.04G224300	G. max	many-to-many	275_447_4651
	AT3G56440	ATG18D	A. thaliana early-release	Glyma.06G140400	G. max	many-to-many	275_447_4651
At5g05150	Species without orthologues						
At5g54730	AT5G54730	G18F	A. thaliana	Glyma.06G267000	G. max	one-to-many	167_275_5202
	AT5G54730	G18F	A. thaliana	Glyma.12G136000	G. max	one-to-many	167_275_5202
	AT5G54730	G18F	A. thaliana	Glyma.12G214600	G. max	one-to-many	167_275_5202
	AT5G54730	G18F	A. thaliana	Glyma.13G287000	G. max	one-to-many	167_275_5202
	AT5G54730	G18F	A. thaliana early-release	Glyma.06G267000	G. max	one-to-many	275_447_5215
	AT5G54730	G18F	A. thaliana early-release	Glyma.12G136000	G. max	one-to-many	275_447_5215
	AT5G54730	G18F	A. thaliana early-release	Glyma.12G214600	G. max	one-to-many	275_447_5215
	AT5G54730	G18F	A. thaliana early-release	Glyma.13G287000	G. max	one-to-many	275_447_5215
At1g03380	AT1G03380	ATG18G	A. thaliana	Glyma.03G148700	G. max	one-to-many	167_275_1595
	AT1G03380	ATG18G	A. thaliana	Glyma.19G152000	G. max	one-to-many	167_275_1595
	AT1G03380	ATG18G	A. thaliana early-release	Glyma.03G148700	G. max	one-to-many	275_447_1602
	AT1G03380	ATG18G	A. thaliana early-release	Glyma.19G152000	G. max	one-to-many	275_447_1602
At1g54710	AT1G54710	ATG18H	A. thaliana	Glyma.10G157700	G. max	one-to-many	167_275_1666
	AT1G54710	ATG18H	A. thaliana	Glyma.20G230900	G. max	one-to-many	167_275_1666
	AT1G54710	ATG18H	A. thaliana early-release	Glyma.10G157700	G. max	one-to-many	275_447_1676
	AT1G54710	ATG18H	A. thaliana early-release	Glyma.20G230900	G. max	one-to-many	275_447_1676
AT5G66930	AT5G66930		A. thaliana	Glyma.17G180900	G. max	one-to-one	167_275_8781
	AT5G66930		A. thaliana early-release	Glyma.17G180900	G. max	one-to-one	275_447_8795

F. EGGNOG analysis of *A. thaliana, P. vulgaris, M. truncatula,and G.max*.

Gene accession numbers	***KOG***	***Organism***	***#Seq***	***Members***	***Close homologs in this group (preferred gene names)***
At2g37840<br>*At3g53930*<br>*At3g61960*	**KOG0595**	*Phaseolus vulgaris*	3 seqs	3885.XP_007134050.1, 3885.XP_007135333.1, 3885.XP_007134049.1	3885.XP_007134050.1, 3885.XP_007135333.1, 3885.XP_007134049.1
At3g19190	**KOG2993**	*Phaseolus vulgaris*		3885.XP_007156552.1	3885.XP_007156552.1
At5g61500	**KOG2981**	*Phaseolus vulgaris*		3885.XP_007131351.1	3885.XP_007131351.1
At2g44140<br>*At3g59950*	**KOG2674**	*Phaseolus vulgaris*		3885.XP_007139667.1	3885.XP_007139667.1
At5g17290	**KOG2976**	*Phaseolus vulgaris*		3885.XP_007141966.1	3885.XP_007141966.1
At3g61710	**KOG2751**	*Phaseolus vulgaris*		3885.XP_007148972.1	3885.XP_007148972.1
At5g45900	**COG0476**	*Phaseolus vulgaris*	12 seqs	3885.XP_007141506.1, 3885.XP_007142173.1, 3885.XP_007133804.1, 3885.XP_007160018.1, 3885.XP_007146816.1,	3885.XP_007141506.1, 3885.XP_007142173.1, 3885.XP_007133804.1, 3885.XP_007160018.1, 3885.XP_007146816.1, CNX5,

				3885.XP_007142325.1, 3885.XP_007160019.1, 3885.XP_007131401.1, 3885.XP_007133324.1, 3885.XP_007154113.1, 3885.XP_007155550.1, 3885.XP_007132545.1	3885.XP_007160019.1, 3885.XP_007131401.1, 3885.XP_007133324.1, 3885.XP_007154113.1, 3885.XP_007155550.1, 3885.XP_007132545.1
At4g21980<br>*At4g04620*<br>*At1g62040*<br>*At2g05630*<br>*At2g45170*<br>*At4g16520*<br>*At3g60640*<br>*At3g06420*<br>*At3g15580*	**KOG1654**	*Phaseolus vulgaris*	6 seqs	3885.XP_007155648.1, 3885.XP_007157342.1, 3885.XP_007133099.1, 3885.XP_007145111.1, 3885.XP_007153954.1, 3885.XP_007132541.1	3885.XP_007155648.1, 3885.XP_007157342.1, 3885.XP_007133099.1, 3885.XP_007145111.1, 3885.XP_007153954.1, 3885.XP_007132541.1
At2g31260	**KOG2173**	*Phaseolus vulgaris*	2 seqs	3885.XP_007162532.1, 3885.XP_007144916.1	3885.XP_007162532.1, 3885.XP_007144916.1
At3g07525	**KOG4741**	*Phaseolus vulgaris*		3885.XP_007134309.1	3885.XP_007134309.1
AT4G30790	**ENOG5028MXS**	*Phaseolus vulgaris*		3885.XP_007154860.1	3885.XP_007154860.1
At1g54210<br>*At3g13970*	**KOG3439**	*Phaseolus vulgaris*		3885.XP_007135446.1	3885.XP_007135446.1
At3g49590<br>*At3g18770*	**KOG4573**	*Phaseolus vulgaris*	2 seqs	3885.XP_007159813.1, 3885.XP_007141345.1	3885.XP_007159813.1, 3885.XP_007141345.1
AT1G77890<br>*AT4G08540*	**KOG2351**	*Phaseolus vulgaris*	2 seqs	3885.XP_007147572.1, 3885.XP_007134240.1	3885.XP_007147572.1, 3885.XP_007134240.1
At5g50230	**KOG0288**	*Phaseolus vulgaris*		3885.XP_007155503.1	3885.XP_007155503.1
At4g30510	**KOG2110**	*Phaseolus vulgaris*		3885.XP_007154849.1	3885.XP_007154849.1
At3g62770	**KOG2111**	*Phaseolus vulgaris*	3 seqs	3885.XP_007144942.1, 3885.XP_007163087.1, 3885.XP_007136398.1	3885.XP_007144942.1, 3885.XP_007163087.1, 3885.XP_007136398.1
At2g40810	**KOG2111**				
At3g56440	**KOG2111**				
At5g05150	**KOG2111**				
At5g54730<br>*At1g03380*<br>*At1g54710*	**KOG2109**	*Phaseolus vulgaris*	4 seqs	3885.XP_007162375.1, 3885.XP_007149695.1, 3885.XP_007144768.1, 3885.XP_007132977.1	3885.XP_007162375.1, 3885.XP_007149695.1, 3885.XP_007144768.1, 3885.XP_007132977.1
AT5G66930	**KOG4493**	*Phaseolus vulgaris*		3885.XP_007155972.1	3885.XP_007155972.1

Gene accession numbers	*KOG*	*Organism*	*#Seq*	*Members**	*Close homologs in this group (preferred gene names)**
At2g37840	**KOG0595**	*Medicago truncatula*	2 seqs	3880.AES72802, 3880.AES72806	11420712, 11418929
At3g53930					
At3g61960					
At3g19190	**KOG2993**	*Medicago truncatula*	2 seqs	3880.AES90192, 3880.AES90193	3880.AES90192, 11441425
At5g61500	**KOG2981**			x	
At2g44140	**KOG2674**	*Medicago truncatula*		3880.AES80500	ATG4
At3g59950	**KOG2674**				
At5g17290	**KOG2976**	*Medicago truncatula*		3880.AES99130	11433638
At3g61710	**KOG2751**	*Medicago truncatula*		3880.AES68897	11414676
At5g45900	**COG0476**	*Medicago truncatula*	9 seqs	3880.AES92586, 3880.AES98758, 3880.AES88130, 3880.AET00579, 3880.AES73061, 3880.AES65672, 3880.AES98760, 3880.AET02216, 3880.AET00193	11436326, 3880.AES98758, 11444042, CNX5, 11431238, 11418341, 11409554, 25501072, 11431855
At4g21980	**KOG1654**	*Medicago truncatula*	4 seqs	3880.AES61582, 3880.AES92053, 3880.AES67226, 3880.AES90925	11443804, 11424644, 11431581, 11410993
At4g04620					
At1g62040					
At2g05630					
At2g45170					
At4g16520					
At3g60640					
At3g06420					
At3g15580					
At2g31260	**KOG2173**			x	
At3g07525	**KOG4741**			x	
AT4G30790	**ENOG5028MXS**	*Medicago truncatula*		3880.AES92467	11428392
At1g54210	**KOG3439**	*Medicago truncatula*		3880.AET01741	ATG12
At3g13970					
At3g49590	**KOG4573**	*Medicago truncatula*	4 seqs	3880.AES72801, 3880.AET04691, 3880.AES98447,	11424978, 3880.AET04691, 11426224, 11432206
At3g18770					
AT1G77890	**KOG2351**	*Medicago truncatula*	2 seqs	3880.AET01383, 3880.AES64594	11418894, 11416659
AT4G08540					
At5g50230	**KOG0288**			x	
At4g30510	**KOG2110**	*Medicago truncatula*		3880.AES92450	11446800
At3g62770	**KOG2111**	*Medicago truncatula*	2 seqs	3880.AES61388, 3880.AES82117	11416663, 11431898
At2g40810					
At3g56440					

At5g05150<br>*At5g54730*<br>*At1g03380*<br>*At1g54710*	**KOG2109**	*Medicago truncatula*	4 seqs	3880.AES61860, 3880.AES66872, 3880.AES61823, 3880.AES61302	3880.AES61860, 11407930, 11413910, 11426407
AT5G66930	**KOG4493**	*Medicago truncatula*		3880.AET04015	11406119

**NCBI ID**	**ID**
3880.AES61302	Medtr1g082300.1
3880.AES61388,	Medtr1g083230.1
3880.AES61582,	Medtr1g086310.1
3880.AES61823,	Medtr1g089110.1
3880.AES62926,	Medtr1g113830.1
3880.AES65672,	Medtr2g045230.1
3880.AES66872,	Medtr2g082770.1
3880.AES67226,	Medtr2g088230.1
3880.AES68897	Medtr3g018770.1
3880.AES72801,	Medtr3g095570.1
3880.AES72806	Medtr3g095620.1
3880.AES72850,	Medtr3g096100.1
3880.AES72851,	Medtr3g096110.1
3880.AES73061,	Medtr3g099240.1
3880.AES74138,	Medtr3g116250.1
3880.AES88130,	Medtr4g048220.1
3880.AES89060,	Medtr4g068580.1
3880.AES90192,	Medtr4g086350
3880.AES90193	Medtr4g086370.1
3880.AES90925	Medtr4g101090.1
3880.AES92053,	Medtr4g123760.1
3880.AES92450	Medtr4g130190.1
3880.AES92467	Medtr4g130370.1
3880.AES92586,	Medtr4g131690.1
3880.AES93602,	Medtr5g005380.1
3880.AES95122,	Medtr5g022000.1
3880.AES96176,	Medtr5g034120.1
3880.AES96520,	Medtr5g038110.1
3880.AES98447,	Medtr5g068710.1
3880.AET04707	Medtr5g068710.1
3880.AES98760,	Medtr5g072480.1
3880.AES99130	Medtr5g076920.1
3880.AET00193	Medtr5g089330.1

3880.AET00579,	Medtr5g093580.1
3880.AES79768	Medtr7g072330.1
3880.AES80500	Medtr7g081230.1
3880.AES82117	Medtr7g108520.1
3880.AET01741	Medtr8g020500.1
3880.AET04015	Medtr8g079240.1
3880.AET04691,	Medtr8g093050.1
3880.AET04735	Medtr8g093570.1
3880.AES61860,	MTR_1g089540
3880.AES64594	MTR_2g027530
3880.AET01383	MTR_8g011890

Gene accession numbers	***KOG***	***Organism***	***#Seq***	***Members***	***Close homologs in this group (preferred gene names)***
At2g37840<br>*At3g53930*<br>*At3g61960*	**KOG0595**	*Glycine max*	6 seqs	3847.GLYMA01G24510.1, 3847.GLYMA16G01970.2, 3847.GLYMA07G05400.3, 3847.GLYMA03G11280.1, 3847.GLYMA04G39350.1, 3847.GLYMA06G15570.2	3847.GLYMA01G24510.1, 3847.GLYMA16G01970.2, 3847.GLYMA07G05400.3, 3847.GLYMA03G11280.1, 3847.GLYMA04G39350.1, 3847.GLYMA06G15570.2
At3g19190	**KOG2993**	*Glycine max*	2 seqs	3847.GLYMA07G33400.4, 3847.GLYMA02G15050.3	3847.GLYMA07G33400.4, 3847.GLYMA02G15050.3
At5g61500	**KOG2981**	*Glycine max*	2 seqs	3847.GLYMA09G36540.1, 3847.GLYMA12G00830.1	3847.GLYMA09G36540.1, GMATG3A
At2g44140<br>*At3g59950*	**KOG2674**	*Glycine max*	2 seqs	3847.GLYMA18G48380.1, 3847.GLYMA09G38000.1	3847.GLYMA18G48380.1, 3847.GLYMA09G38000.1
At5g17290	**KOG2976**	*Glycine max*	3 seqs	3847.GLYMA10G30170.1, 3847.GLYMA02G40770.1, 3847.GLYMA14G39095.1	3847.GLYMA10G30170.1, ATG5, 3847.GLYMA14G39095.1
At3g61710	**KOG2751**	*Glycine max*	2 seqs	3847.GLYMA04G26840.1, 3847.GLYMA11G21490.1	3847.GLYMA04G26840.1, ATG6
At5g45900	**COG0476**	*Glycine max*	21 seqs	3847.GLYMA05G03350.1, 3847.GLYMA12G01250.1, 3847.GLYMA12G10540.1, 3847.GLYMA12G36450.1, 3847.GLYMA06G46210.1, 3847.GLYMA14G37610.1, 3847.GLYMA13G27140.1, 3847.GLYMA18G06620.1, 3847.GLYMA02G46300.1, 3847.GLYMA05G38190.1, 3847.GLYMA07G38770.1, 3847.GLYMA17G01940.4, 3847.GLYMA14G02410.1, 3847.GLYMA07G12250.1, 3847.GLYMA14G02260.1, 3847.GLYMA17G13940.1, 3847.GLYMA11G29302.1, 3847.GLYMA14G04000.1, 3847.GLYMA02G39495.2,	3847.GLYMA05G03350.1, GMATG7, 3847.GLYMA12G10540.1, 3847.GLYMA12G36450.1, 3847.GLYMA06G46210.1, 3847.GLYMA14G37610.1, 3847.GLYMA13G27140.1, 3847.GLYMA18G06620.1, 3847.GLYMA02G46300.1, 3847.GLYMA05G38190.1, 3847.GLYMA07G38770.1, 3847.GLYMA17G01940.4, 3847.GLYMA14G02410.1, 3847.GLYMA07G12250.1, 3847.GLYMA14G02260.1, 3847.GLYMA17G13940.1, 3847.GLYMA11G29302.1, 3847.GLYMA14G04000.1, 3847.GLYMA02G39495.2,

				3847.GLYMA02G44730.1,<br>3847.GLYMA08G01440.1	3847.GLYMA02G44730.1,<br>3847.GLYMA08G01440.1
At4g21980<br>*At4g04620*<br>*At1g62040*<br>*At2g05630*<br>*At2g45170*<br>*At4g16520*<br>*At3g60640*<br>*At3g06420*<br>*At3g15580*	**KOG1654**	*Glycine max*	13 seqs	3847.GLYMA10G01220.1,<br>3847.GLYMA17G14970.1,<br>3847.GLYMA09G00630.3,<br>3847.GLYMA07G39090.1,<br>3847.GLYMA12G10510.1,<br>3847.GLYMA02G01180.1,<br>3847.GLYMA05G04540.1,<br>3847.GLYMA11G03460.1,<br>3847.GLYMA15G11510.1,<br>3847.GLYMA01G41910.2,<br>3847.GLYMA17G01650.1,<br>3847.GLYMA06G46270.3,<br>3847.GLYMA15G21427.1	3847.GLYMA10G01220.1,<br>3847.GLYMA17G14970.1,<br>3847.GLYMA09G00630.3,<br>3847.GLYMA07G39090.1,<br>ATG8d, ATG8i,<br>3847.GLYMA05G04540.1,<br>3847.GLYMA11G03460.1,<br>3847.GLYMA15G11510.1,<br>3847.GLYMA01G41910.2,<br>3847.GLYMA17G01650.1,<br>3847.GLYMA06G46270.3,<br>3847.GLYMA15G21427.1
At2g31260	**KOG2173**	*Glycine max*	4 seqs	3847.GLYMA13G18300.2,<br>3847.GLYMA19G34630.1,<br>3847.GLYMA10G04120.1,<br>3847.GLYMA03G31890.3	3847.GLYMA13G18300.2,<br>ATG9,<br>3847.GLYMA10G04120.1,<br>3847.GLYMA03G31890.3
At3g07525	**KOG4741**	*Glycine max*	2 seqs	3847.GLYMA07G12280.2,<br>3847.GLYMA03G23850.2	3847.GLYMA07G12280.2,<br>3847.GLYMA03G23850.2
AT4G30790	**ENOG5028MXS**	*Glycine max*	2 seqs	3847.GLYMA02G36800.2,<br>3847.GLYMA17G07920.1	3847.GLYMA02G36800.2,<br>3847.GLYMA17G07920.1
At1g54210<br>*At3g13970*	**KOG3439**	*Glycine max*	2 seqs	3847.GLYMA16G00940.2,<br>3847.GLYMA07G04250.1	3847.GLYMA16G00940.2,<br>3847.GLYMA07G04250.1
At3g49590<br>*At3g18770*	**KOG4573**	*Glycine max*	4 seqs	3847.GLYMA05G32250.1,<br>3847.GLYMA08G15500.2,<br>3847.GLYMA14G36553.2, 3847.<br>.1	3847.GLYMA05G32250.1,<br>3847.GLYMA08G15500.2,<br>3847.GLYMA14G36553.2,<br>3847.GLYMA02G38530.1
AT1G77890	**KOG2351**	*Glycine max*	**6 seqs**	3847.GLYMA08G15515.1,<br>3847.GLYMA09G01880.2,<br>3847.GLYMA15G12830.1,<br>3847.GLYMA16G09890.1,<br>3847.GLYMA03G22220.1,<br>3847.GLYMA05G32260.1	3847.GLYMA08G15515.1,<br>3847.GLYMA09G01880.2,<br>3847.GLYMA15G12830.1,<br>3847.GLYMA16G09890.1,<br>3847.GLYMA03G22220.1,<br>3847.GLYMA05G32260.1
AT4G08540					
At5g50230	**KOG0288**	*Glycine max*	2 seqs	3847.GLYMA17G13520.2,<br>3847.GLYMA05G02850.1	3847.GLYMA17G13520.2,<br>3847.GLYMA05G02850.1
At4g30510	**KOG2110**	*Glycine max*	4 seqs	3847.GLYMA02G36960.4,<br>3847.GLYMA10G25246.1,<br>3847.GLYMA17G07800.2,<br>3847.GLYMA16G20500.2	3847.GLYMA02G36960.4,<br>3847.GLYMA10G25246.1,<br>3847.GLYMA17G07800.2,<br>3847.GLYMA16G20500.2
At3g62770<br>*At2g40810*<br>*At3g56440*<br>*At5g05150*	**KOG2111**	*Glycine max*	6 seqs	3847.GLYMA10G29320.1,<br>3847.GLYMA19G39660.1,<br>3847.GLYMA06G14550.2,<br>3847.GLYMA03G37030.1,<br>3847.GLYMA04G40230.2,<br>3847.GLYMA20G37960.1	3847.GLYMA10G29320.1,<br>3847.GLYMA19G39660.1,<br>3847.GLYMA06G14550.2,<br>3847.GLYMA03G37030.1,<br>3847.GLYMA04G40230.2,<br>3847.GLYMA20G37960.1

G.PANTHER analysis of *A. thaliana, P. vulgaris, M. truncatula,and G.max*.

Gene accession numbers	*PANTHER*	*Description*
At2g37840	PTHR24348:SF26	PROTEIN KINASE FAMILY PROTEIN
At3g53930	PTHR24348:SF26	PROTEIN KINASE FAMILY PROTEIN
At3g61960	PTHR24348:SF26	PROTEIN KINASE FAMILY PROTEIN
At3g19190	PTHR13190	FAMILY NOT NAMED
At5g61500	PTHR12866:SF2	UBIQUITIN-LIKE-CONJUGATING ENZYME ATG3
At2g44140	PTHR22624	APG4 AUTOPHAGY 4-RELATED
At3g59950	PTHR22624	APG4 AUTOPHAGY 4-RELATED
At5g17290	PTHR13040	AUTOPHAGY PROTEIN 5
At3g61710	PTHR12768	BECLIN 1
At5g45900	PTHR10953:SF3	UBIQUITIN-LIKE MODIFIER-ACTIVATING ENZYME ATG7
At4g21980	PTHR10969:SF43	AUTOPHAGY-RELATED PROTEIN 8A-RELATED
At4g04620	PTHR10969:SF43	AUTOPHAGY-RELATED PROTEIN 8A-RELATED
At1g62040	PTHR10969:SF43	AUTOPHAGY-RELATED PROTEIN 8A-RELATED
At2g05630	PTHR10969:SF43	AUTOPHAGY-RELATED PROTEIN 8A-RELATED
At2g45170	PTHR10969:SF39	AUTOPHAGY-RELATED PROTEIN 8F
At4g16520	PTHR10969:SF39	AUTOPHAGY-RELATED PROTEIN 8F
At3g60640	PTHR10969:SF31	AUTOPHAGY-RELATED PROTEIN 8G
At3g06420	PTHR10969:SF36	AUTOPHAGY-RELATED PROTEIN 8H
At3g15580	PTHR10969:SF25	AUTOPHAGY-RELATED PROTEIN 8I
At2g31260	PTHR13038:SF10	AUTOPHAGY-SPECIFIC GENE 9
At3g07525	PTHR12866:SF5	UBIQUITIN-LIKE-CONJUGATING ENZYME ATG10
AT4G30790	PTHR13222:SF1	RB1-INDUCIBLE COILED-COIL PROTEIN 1
At1g54210	PTHR13385	AUTOPHAGY PROTEIN 12
At3g13970	PTHR13385	AUTOPHAGY PROTEIN 12
At3g49590	PTHR13430:SF3	AUTOPHAGY-RELATED PROTEIN 13
At3g18770	PTHR13430	UNCHARACTERIZED
At5g50230	PTHR19878	AUTOPHAGY PROTEIN 16-LIKE
At3g62770	PTHR11227:SF18	WD REPEAT DOMAIN PHOSPHOINOSITIDE-INTERACTING PROTEIN 3
At4g30510	PTHR11227:SF17	AUTOPHAGY-SPECIFIC GENE 18, ISOFORM E
At2g40810	PTHR11227:SF25	AUTOPHAGY-RELATED PROTEIN 18C-RELATED
At3g56440	PTHR11227:SF25	AUTOPHAGY-RELATED PROTEIN 18C-RELATED
At5g05150	PTHR11227:SF25	AUTOPHAGY-RELATED PROTEIN 18C-RELATED
At5g54730	PTHR13268:SF1	AUTOPHAGY-RELATED PROTEIN 18F
At1g03380	PTHR13268:SF2	AUTOPHAGY-RELATED PROTEIN 18G
At1g54710	PTHR13268:SF0	BREAST CARCINOMA-AMPLIFIED SEQUENCE 3

AT5G66930	PTHR13292:SF0	AUTOPHAGY-RELATED PROTEIN 101
AT1G50030	PTHR11139:SF9	SERINE/THREONINE-PROTEIN KINASE MTOR
AT3G08850	PTHR12848:SF16	REGULATORY-ASSOCIATED PROTEIN OF MTOR
AT5G01770	PTHR12848:SF16	REGULATORY-ASSOCIATED PROTEIN OF MTOR
AT2G22040	PTHR19842	G BETA-LIKE PROTEIN GBL
AT3G18140	PTHR19842	G BETA-LIKE PROTEIN GBL
Gene accession numbers	**PANTHER**	**Description**
Phvul.010G120500	PTHR24348:SF26	PROTEIN KINASE FAMILY PROTEIN
Phvul.010G015100	PTHR24348	PROTEIN KINASE FAMILY PROTEIN
Phvul.003G295800	PTHR13190	FAMILY NOT NAMED
Phvul.011G006500	PTHR12866:SF2	UBIQUITIN-LIKE-CONJUGATING ENZYME ATG3
Phvul.008G048900	PTHR22624:SF34	AUTOPHAGY-SPECIFIC GENE 4, ISOFORM A
Phvul.008G241000	PTHR13040:SF2	AUTOPHAGY PROTEIN 5
Phvul.005G029900	PTHR12768:SF4	BECLIN 1
Phvul.011G010700	PTHR10953:SF3	UBIQUITIN-LIKE MODIFIER-ACTIVATING ENZYME ATG7
Phvul.003G079300	PTHR10969:SF43	AUTOPHAGY-RELATED PROTEIN 8A-RELATED
Phvul.011G103300	PTHR10969:SF43	AUTOPHAGY-RELATED PROTEIN 8A-RELATED
Phvul.006G149640	PTHR10969:SF43	AUTOPHAGY-RELATED PROTEIN 8A-RELATED
Phvul.011G151600	PTHR10969:SF43	AUTOPHAGY-RELATED PROTEIN 8A-RELATED
Phvul.003G219600	PTHR10969:SF39	AUTOPHAGY-RELATED PROTEIN 8F
Phvul.002G062200	PTHR10969:SF39	AUTOPHAGY-RELATED PROTEIN 8F
Phvul.007G210800	PTHR10969:SF25	AUTOPHAGY-RELATED PROTEIN 8I
Phvul.001G159900	PTHR13038:SF10	AUTOPHAGY-SPECIFIC GENE 9
Phvul.007G194300	PTHR13038:SF10	AUTOPHAGY-SPECIFIC GENE 9
Phvul.010G036300	PTHR12866:SF5	UBIQUITIN-LIKE-CONJUGATING ENZYME ATG10
Phvul.003G153800	PTHR13222:SF1	RB1-INDUCIBLE COILED-COIL PROTEIN 1
Phvul.010G130300	PTHR13385	AUTOPHAGY PROTEIN 12
Phvul.008G187800	PTHR13430:SF3	AUTOPHAGY-RELATED PROTEIN 13
Phvul.002G269600	PTHR13430	UNCHARACTERIZED
Phvul.003G207100	PTHR19878	AUTOPHAGY PROTEIN 16-LIKE
Phvul.002G020500	PTHR19878	AUTOPHAGY PROTEIN 16-LIKE
Phvul.003G245800	PTHR19878	AUTOPHAGY PROTEIN 16-LIKE
Phvul.007G196400	PTHR11227:SF25	AUTOPHAGY-RELATED PROTEIN 18C-RELATED
Phvul.001G205000	PTHR11227:SF18	WD REPEAT DOMAIN PHOSPHOINOSITIDE-INTERACTING PROTEIN 3
Phvul.003G152800	PTHR11227:SF17	AUTOPHAGY-SPECIFIC GENE 18, ISOFORM E
Phvul.009G041700	PTHR11227:SF25	AUTOPHAGY-RELATED PROTEIN 18C-RELATED
Phvul.005G091300	PTHR13268:SF1	AUTOPHAGY-RELATED PROTEIN 18F
Phvul.001G146700	PTHR13268:SF2	AUTOPHAGY-RELATED PROTEIN 18G
Phvul.011G140900	PTHR13268:SF1	AUTOPHAGY-RELATED PROTEIN 18F

Phvul.007G183100	PTHR13268:SF2	AUTOPHAGY-RELATED PROTEIN 18G
Phvul.003G24800	PTHR13292:SF0	AUTOPHAGY-RELATED PROTEIN 101
Phvul.002G04990	PTHR11139:SF9	SERINE/THREONINE-PROTEIN KINASE MTOR
Phvul.008G08780	PTHR12848:SF16	REGULATORY-ASSOCIATED PROTEIN OF MTOR
Phvul.008G8810	PTHR12848:SF16	REGULATORY-ASSOCIATED PROTEIN OF MTOR
Phvul.006G17370	PTHR19842	G BETA-LIKE PROTEIN GBL
Gene accession numbers	**PANTHER**	**Description**
Medtr3g095620	PTHR24348:SF24	PROTEIN KINASE FAMILY PROTEIN
Medtr4g019410	PTHR24348	SERINE/THREONINE-PROTEIN KINASE UNC-51-RELATED
Medtr8g024100	PTHR24348	SERINE/THREONINE-PROTEIN KINASE UNC-51-RELATED
Medtr4g086370	PTHR13190	FAMILY NOT NAMED
Medtr4g036265	PTHR12866:SF2	UBIQUITIN-LIKE-CONJUGATING ENZYME ATG3
Medtr7g081230	PTHR22624	APG4 AUTOPHAGY 4-RELATED
Medtr5g076920	PTHR13040	AUTOPHAGY PROTEIN 5
Medtr3g018770	PTHR12768	BECLIN 1
Medtr0003s0540	PTHR10953:SF3	UBIQUITIN-LIKE MODIFIER-ACTIVATING ENZYME ATG7
Medtr2g023430	PTHR10969:SF43	AUTOPHAGY-RELATED PROTEIN 8A-RELATED
Medtr4g048510	PTHR10969:SF43	AUTOPHAGY-RELATED PROTEIN 8A-RELATED
Medtr4g037225	PTHR10969:SF61	AUTOPHAGY-RELATED PROTEIN 8C
Medtr2g88230	PTHR10969:SF74	AUTOPHAGY-RELATED PROTEIN
Medtr4g101090	PTHR10969:SF39	AUTOPHAGY-RELATED PROTEIN 8F
Medtr4g123760	PTHR10969:SF36	AUTOPHAGY-RELATED PROTEIN 8H
Medtr7g096540	PTHR10969:SF36	AUTOPHAGY-RELATED PROTEIN 8H
Medtr1g086310	PTHR10969:SF25	AUTOPHAGY-RELATED PROTEIN 8I
Medtr7g096680	PTHR13038:SF10	AUTOPHAGY-SPECIFIC GENE 9
Medtr1g070160	PTHR13038:SF10	AUTOPHAGY-SPECIFIC GENE 9
Medtr8g010140	PTHR12866:SF5	UBIQUITIN-LIKE-CONJUGATING ENZYME ATG10
Medtr4g130370	PTHR13222:SF1	RB1-INDUCIBLE COILED-COIL PROTEIN 1
Medtr8g020500	PTHR13385	AUTOPHAGY PROTEIN 12
Medtr5g068710	PTHR13430:SF3	AUTOPHAGY-RELATED PROTEIN 13
MTR_8g011890	PTHR21297	DNA-DIRECTED RNA POLYMERASE II
MTR_2g027530	PTHR21297:SF2	DNA-DIRECTED RNA POLYMERASES IV AND V SUBUNIT 4
Medtr3g095570	PTHR13430	UNCHARACTERIZED
Medtr8g093050	PTHR13430	UNCHARACTERIZED
Medtr4g104380	PTHR19878	AUTOPHAGY PROTEIN 16-LIKE
Medtr3g075400	PTHR19878	AUTOPHAGY PROTEIN 16-LIKE
Medtr4g007500	PTHR19878	AUTOPHAGY PROTEIN 16-LIKE
MTR_6g27280	PTHR19878	AUTOPHAGY PROTEIN 16-LIKE

Medtr1g083230	PTHR11227:SF25	AUTOPHAGY-RELATED PROTEIN 18C-RELATED
Medtr3g093590	PTHR11227:SF25	AUTOPHAGY-RELATED PROTEIN 18C-RELATED
Medtr1g088855	PTHR11227:SF25	AUTOPHAGY-RELATED PROTEIN 18C-RELATED
Medtr7g108520.	PTHR11227:SF18	WD REPEAT DOMAIN PHOSPHOINOSITIDE-INTERACTING PROTEIN 3
Medtr4g130190	PTHR11227:SF17	AUTOPHAGY-SPECIFIC GENE 18, ISOFORM E
Medtr2g082770	PTHR13268:SF1	AUTOPHAGY-RELATED PROTEIN 18F
Medtr1g089110	PTHR13268:SF2	AUTOPHAGY-RELATED PROTEIN 18G
Medtr1g082300	PTHR13268:SF2	AUTOPHAGY-RELATED PROTEIN 18G
Medtr8g079240	PTHR13292:SF0	AUTOPHAGY-RELATED PROTEIN 101
Medtr5g005380	PTHR11139:SF9	SERINE/THREONINE-PROTEIN KINASE MTOR
Medtr7g072330	PTHR12848:SF16	REGULATORY-ASSOCIATED PROTEIN OF MTOR
Medtr2g016690	PTHR19842	G BETA-LIKE PROTEIN GBL

Gene accession numbers	***PANTHER***	***Description***
Glyma.04G215500	PTHR24348	SERINE/THREONINE-PROTEIN KINASE UNC-51-RELATED
Glyma.06G150700	PTHR24348	SERINE/THREONINE-PROTEIN KINASE UNC-51-RELATED
Glyma.03G069800	PTHR24348	SERINE/THREONINE-PROTEIN KINASE UNC-51-RELATED
Glyma.07G048400	PTHR24348	SERINE/THREONINE-PROTEIN KINASE UNC-51-RELATED
GLYMA_01G099600	PTHR24348:SF22	SERINE/THREONINE-PROTEIN KINASE ULK3
GLYMA_15G088500	PTHR24348:SF22	SERINE/THREONINE-PROTEIN KINASE ULK3
GLYMA_03G069800	PTHR24348:SF22	SERINE/THREONINE-PROTEIN KINASE ULK3
GLYMA_14G026700	PTHR24348:SF22	SERINE/THREONINE-PROTEIN KINASE ULK3
GLYMA_20G031300	PTHR24348:SF22	SERINE/THREONINE-PROTEIN KINASE ULK3
GLYMA_16G017300	PTHR24348:SF39	AUTOPHAGY-RELATED 1, ISOFORM B
Glyma.02G133400	PTHR13190	FAMILY NOT NAMED
Glyma.07G211600	PTHR13190	FAMILY NOT NAMED
Glyma.12G005700	PTHR12866:SF2	UBIQUITIN-LIKE-CONJUGATING ENZYME ATG3
Glyma.09G231000	PTHR12866:SF2	UBIQUITIN-LIKE-CONJUGATING ENZYME ATG3
Glyma.18G248400	PTHR22624	APG4 AUTOPHAGY 4-RELATED
Glyma.09G244800	PTHR22624	APG4 AUTOPHAGY 4-RELATED
Glyma.14G210200	PTHR13040	AUTOPHAGY PROTEIN 5
GLYMA.01G231500	PTHR13040:SF2	glyma_14G187000
Glyma.02G240700	PTHR13040:SF2	AUTOPHAGY PROTEIN 5
Glyma.11G153900	PTHR12768	BECLIN 1
Glyma.04G141000	PTHR12768	BECLIN 1
Glyma.12G010000	PTHR10953:SF3	UBIQUITIN-LIKE MODIFIER-ACTIVATING ENZYME ATG7
glyma.15G108200	PTHR10969:SF43	AUTOPHAGY-RELATED PROTEIN 8A-RELATED
glyma.17G013000	PTHR10969:SF43	AUTOPHAGY-RELATED PROTEIN 8A-RELATED
glyma.12G098400	PTHR10969:SF43	AUTOPHAGY-RELATED PROTEIN 8A-RELATED

Glyma.06G306300	PTHR10969:SF43	AUTOPHAGY-RELATED PROTEIN 8A-RELATED
Glyma.07G261000	PTHR10969:SF43	AUTOPHAGY-RELATED PROTEIN 8A-RELATED
Glyma.09G003900	PTHR10969:SF43	AUTOPHAGY-RELATED PROTEIN 8A-RELATED
Glyma.15G188600	PTHR10969:SF43	AUTOPHAGY-RELATED PROTEIN 8A-RELATED
glyma.17G140700	PTHR10969:SF39	AUTOPHAGY-RELATED PROTEIN 8F
GLYMA.05G058300		AUTOPHAGY-RELATED PROTEIN 8F (PTHR10969:SF58)
GLYMA.1G210200		AUTOPHAGY-RELATED PROTEIN 8F (PTHR10969:SF58)
GLYMA.11G031800		AUTOPHAGY-RELATED PROTEIN 8F (PTHR10969:SF58)
Glyma.10G009300	PTHR10969:SF25	AUTOPHAGY-RELATED PROTEIN 8I
glyma.02G008800	PTHR10969:SF25	AUTOPHAGY-RELATED PROTEIN 8I
glyma.13G122200	PTHR13038:SF10	AUTOPHAGY-SPECIFIC GENE 9
Glyma.03G162100.	PTHR13038:SF10	AUTOPHAGY-SPECIFIC GENE 9
Glyma.19G163500.	PTHR13038:SF10	AUTOPHAGY-SPECIFIC GENE 9
Glyma.10G035800.	PTHR13038:SF10	AUTOPHAGY-SPECIFIC GENE 9
glyma.03G097000	PTHR12866:SF5	UBIQUITIN-LIKE-CONJUGATING ENZYME ATG10
Glyma.07G124300	PTHR12866:SF5	UBIQUITIN-LIKE-CONJUGATING ENZYME ATG10
glyma.17G071400	PTHR13222:SF1	RB1-INDUCIBLE COILED-COIL PROTEIN 1
Glyma.02G206500	PTHR13222:SF1	RB1-INDUCIBLE COILED-COIL PROTEIN 1
glyma.16G007300	PTHR13385	AUTOPHAGY PROTEIN 12
glyma.07G038100	PTHR13385	AUTOPHAGY PROTEIN 12
glyma.14G187000	PTHR13430:SF3	AUTOPHAGY-RELATED PROTEIN 13
glyma.05G189000	PTHR13430	UNCHARACTERIZED
Glyma.08G146700	PTHR13430	UNCHARACTERIZED
Glyma.02G220700	PTHR13430:SF3	AUTOPHAGY-RELATED PROTEIN 13
GLYMA_15G121800	PTHR21297:SF2	DNA-DIRECTED RNA POLYMERASES IV AND V SUBUNIT 4
GLYMA_16G084800	PTHR21297	DNA-DIRECTED RNA POLYMERASE II
GLYMA_03G088500	PTHR21297	DNA-DIRECTED RNA POLYMERASE II
GLYMA_09G016100	PTHR21297:SF2	DNA-DIRECTED RNA POLYMERASES IV AND V SUBUNIT 4
GLYMA_08G146800	PTHR21297	DNA-DIRECTED RNA POLYMERASE II
GLYMA_05G189100	PTHR21297	DNA-DIRECTED RNA POLYMERASE II
glyma.05G043700	PTHR19878	AUTOPHAGY PROTEIN 16-LIKE
Glyma.05G090500	PTHR19878	AUTOPHAGY PROTEIN 16-LIKE
Glyma.17G176300	PTHR19878	AUTOPHAGY PROTEIN 16-LIKE
Glyma.17G126200	PTHR19878	AUTOPHAGY PROTEIN 16-LIKE
Glyma.11G057700	PTHR19878	AUTOPHAGY PROTEIN 16-LIKE
Glyma.01G184500	PTHR19878	AUTOPHAGY PROTEIN 16-LIKE
glyma.20G235800	PTHR11227:SF18	WD REPEAT DOMAIN PHOSPHOINOSITIDE-INTERACTING PROTEIN 3
Glyma.19G209200	PTHR11227:SF18	WD REPEAT DOMAIN PHOSPHOINOSITIDE-INTERACTING PROTEIN 3
Glyma.10G152500.	PTHR11227:SF18	WD REPEAT DOMAIN PHOSPHOINOSITIDE-INTERACTING PROTEIN 3

Glyma.03G212100	PTHR11227:SF18	WD REPEAT DOMAIN PHOSPHOINOSITIDE-INTERACTING PROTEIN 3
Glyma.17G070200	PTHR11227:SF17	AUTOPHAGY-SPECIFIC GENE 18, ISOFORM E
Glyma.02G207500	PTHR11227:SF17	AUTOPHAGY-SPECIFIC GENE 18, ISOFORM E
Glyma.16G109400	PTHR11227:SF17	AUTOPHAGY-SPECIFIC GENE 18, ISOFORM E
Glyma.10G126200	PTHR11227:SF17	AUTOPHAGY-SPECIFIC GENE 18, ISOFORM E
glyma.06G140400	PTHR11227:SF25	AUTOPHAGY-RELATED PROTEIN 18C-RELATED
Glyma.04G224300	PTHR11227:SF25	AUTOPHAGY-RELATED PROTEIN 18C-RELATED
Glyma.07G203900	PTHR11227:SF25	AUTOPHAGY-RELATED PROTEIN 18C-RELATED
glyma.13G287000	PTHR13268:SF1	AUTOPHAGY-RELATED PROTEIN 18F
Glyma.12G214600	PTHR13268:SF1	AUTOPHAGY-RELATED PROTEIN 18F
Glyma.12G136000	PTHR13268:SF1	AUTOPHAGY-RELATED PROTEIN 18F
Glyma.06G267000	PTHR13268:SF1	AUTOPHAGY-RELATED PROTEIN 18F
glyma.03G148700	PTHR13268:SF2	AUTOPHAGY-RELATED PROTEIN 18G
Glyma.20G230900	PTHR13268:SF2	AUTOPHAGY-RELATED PROTEIN 18G
Glyma.19G152000	PTHR13268:SF2	AUTOPHAGY-RELATED PROTEIN 18G
glyma.10G157700	PTHR13268:SF0	BREAST CARCINOMA-AMPLIFIED SEQUENCE 3
Glyma.17G180900	PTHR13292:SF0	AUTOPHAGY-RELATED PROTEIN 101
Glyma.01G241300	PTHR11139:SF9	SERINE/THREONINE-PROTEIN KINASE MTOR
Glyma.11G002600	PTHR11139:SF9	SERINE/THREONINE-PROTEIN KINASE MTOR
Glyma.09G278500	PTHR12848:SF16	REGULATORY-ASSOCIATED PROTEIN OF MTOR
Glyma.18G210300	PTHR12848:SF16	REGULATORY-ASSOCIATED PROTEIN OF MTOR
Glyma.U032100.1.p	PTHR12848:SF16	REGULATORY-ASSOCIATED PROTEIN OF MTOR
Glyma.15G085200	PTHR19842	G BETA-LIKE PROTEIN GBL
Glyma.13G227200	PTHR19842	G BETA-LIKE PROTEIN GBL
