# Supplementary material for: Exploration of Autophagy Families in Legumes and Dissection of the ATG18 Family with a Special Focus on Phaseolus vulgaris
Source: Plants (Basel). 2021 Nov 29;10(12):2619. doi: 10.3390/plants10122619 (PMC8703869; doi:10.3390/plants10122619)
Supplement: Supplementary file 1 [file plants-10-02619-s001.zip › Supplementary information/Supp. Info. SI5.pdf]

Supp. Info. SI5. Alignment and synteny of ATG genes between *A. thaliana* and legumes using the comparative genomics in Ensembl. (A) *ATG synteny (B)P. vulgaris Paralogous (C) M. truncatula Paralogous (D) G.max Paralogous.*

A. ATG synteny

ATG1

At3g61960

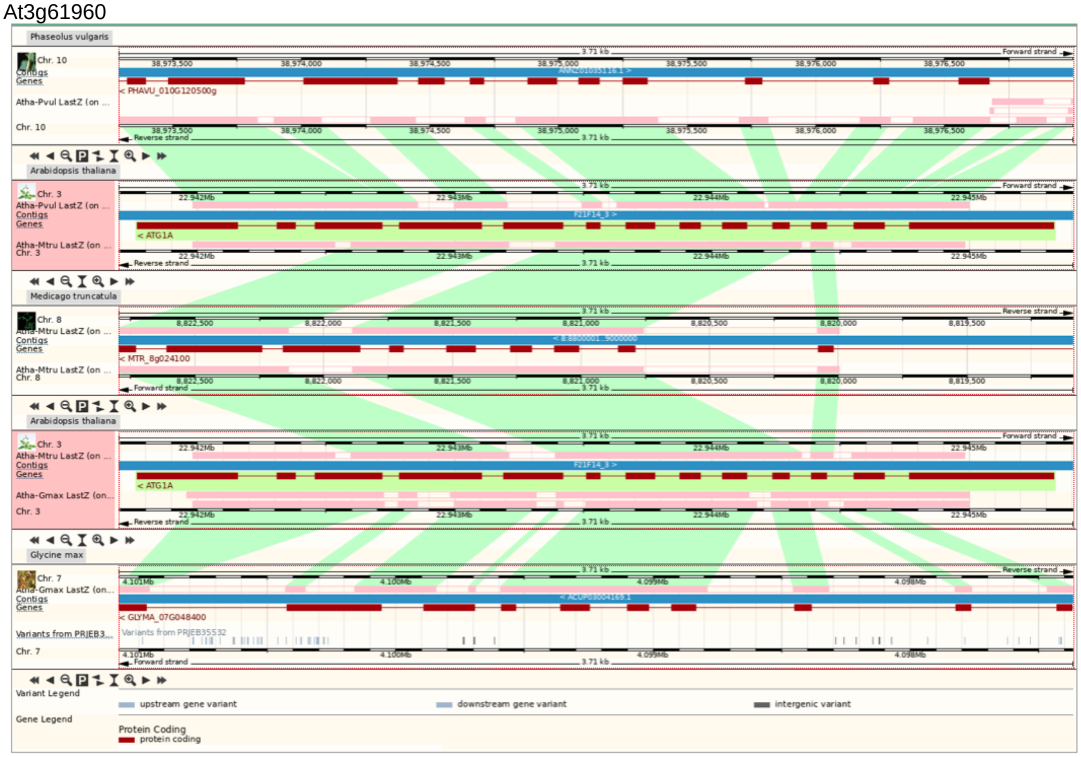

At3g53930

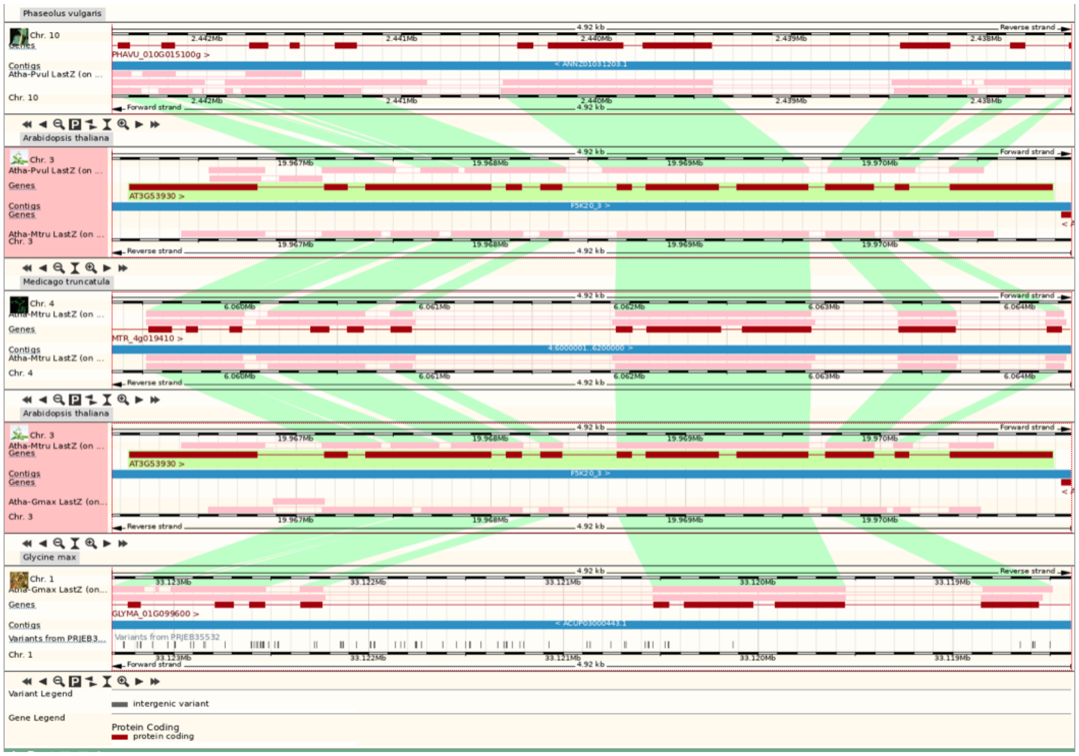

At2g37840

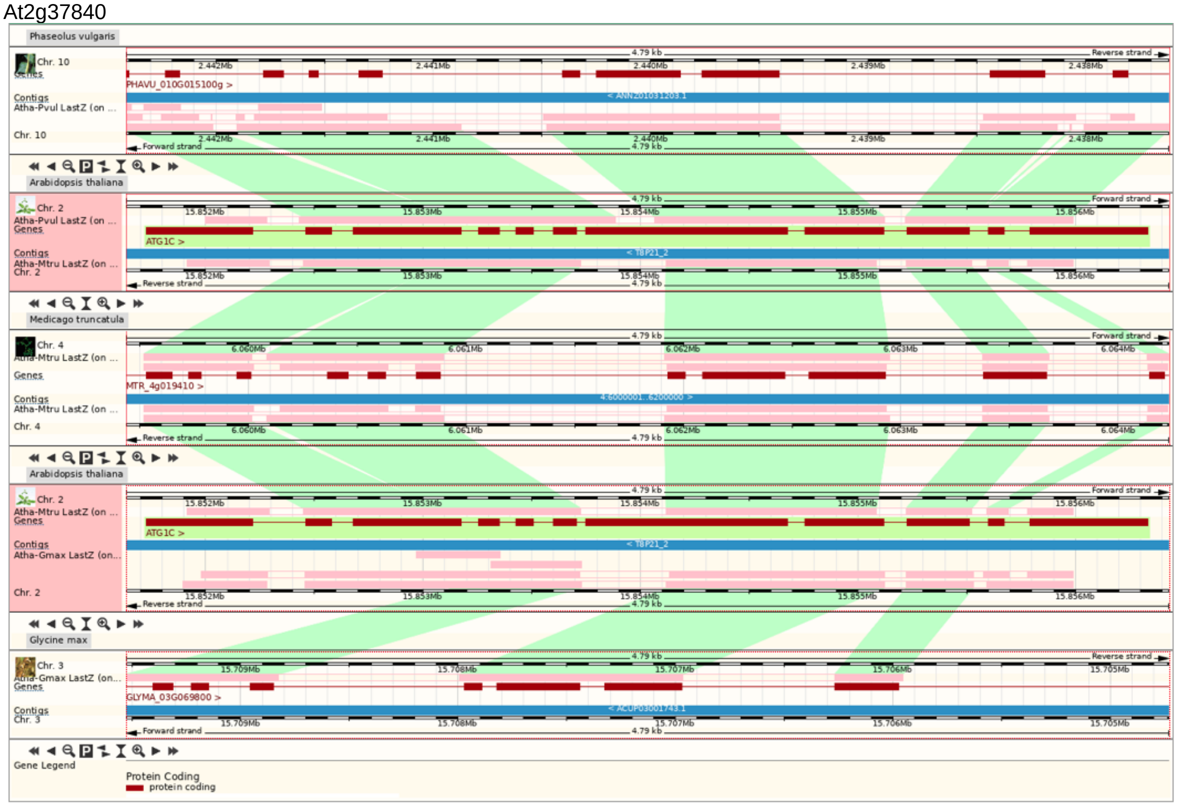

At1g49180

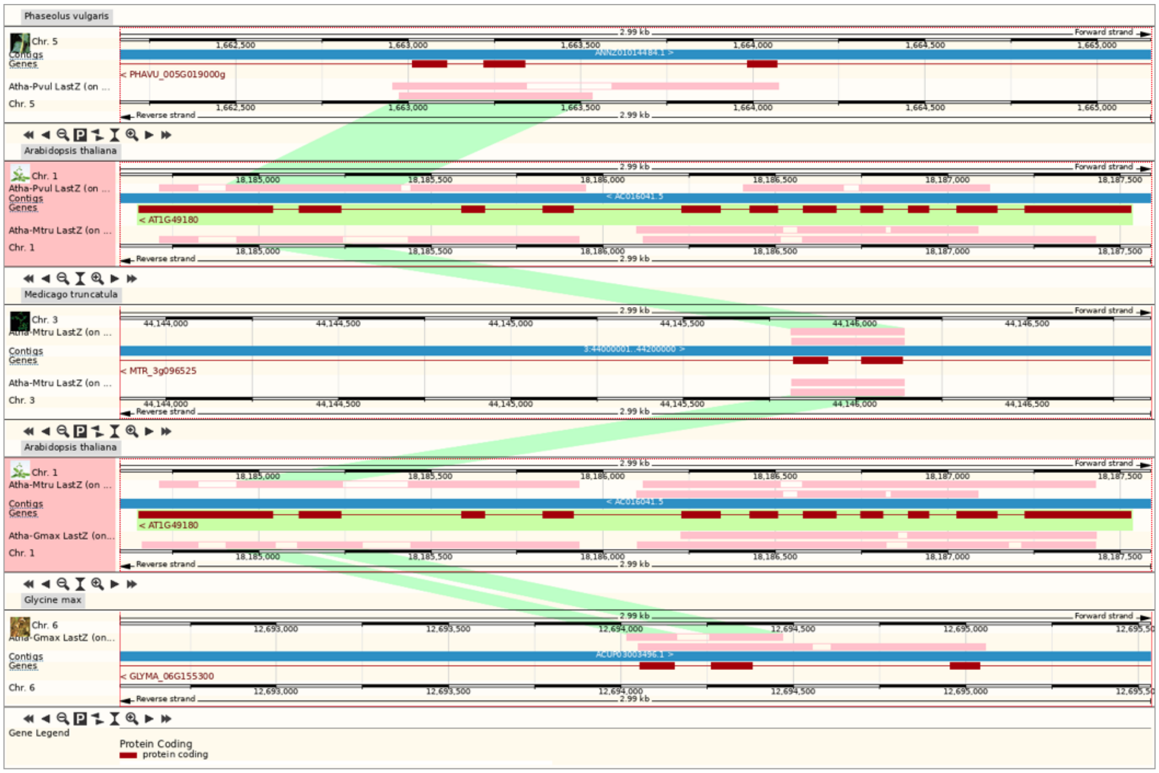

ATG2

At3g19190

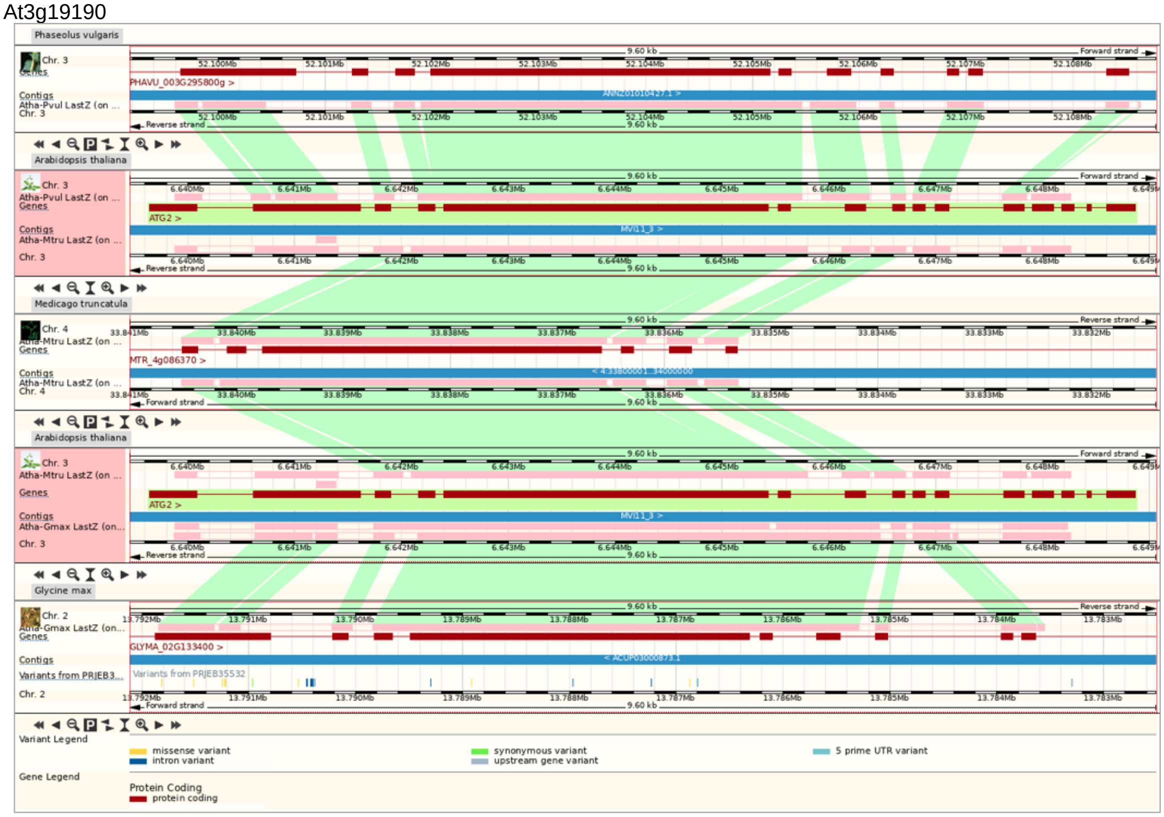

ATG3

At5g61500

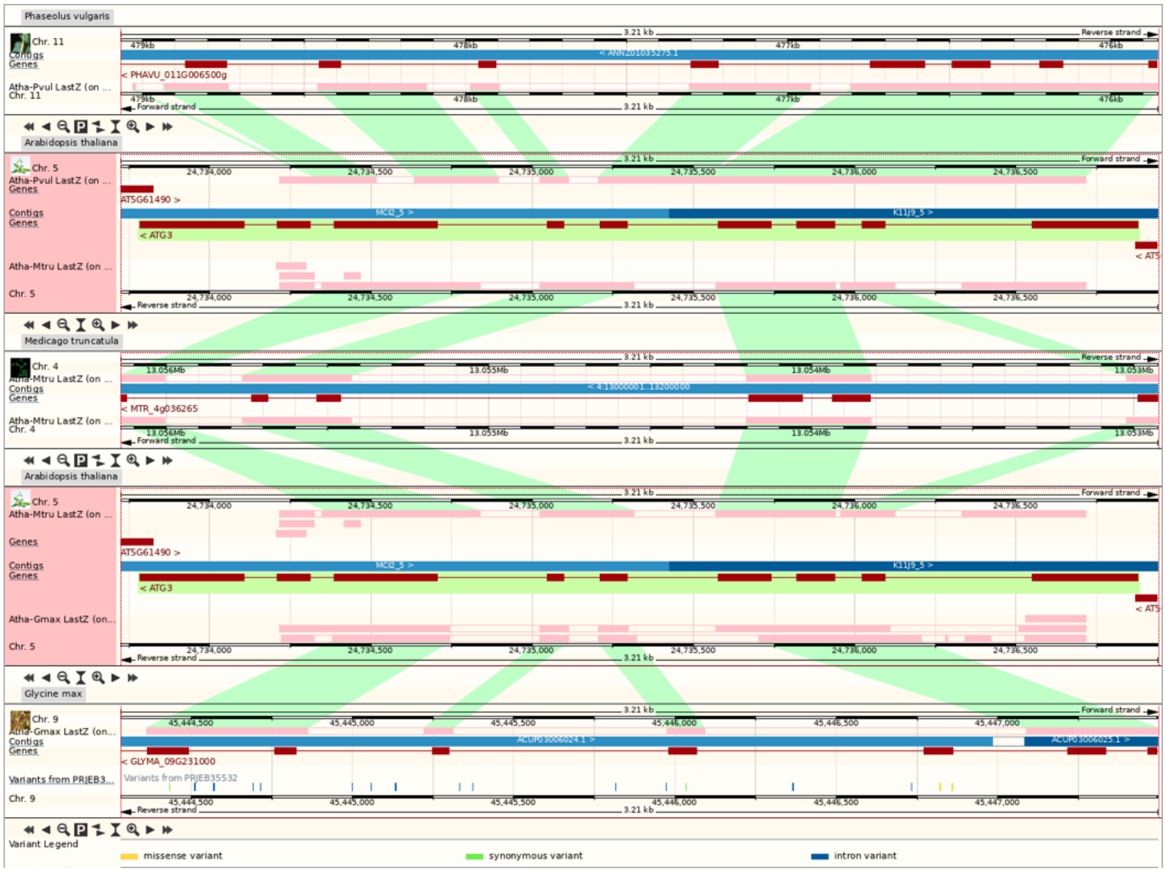

ATG4

At2g44140

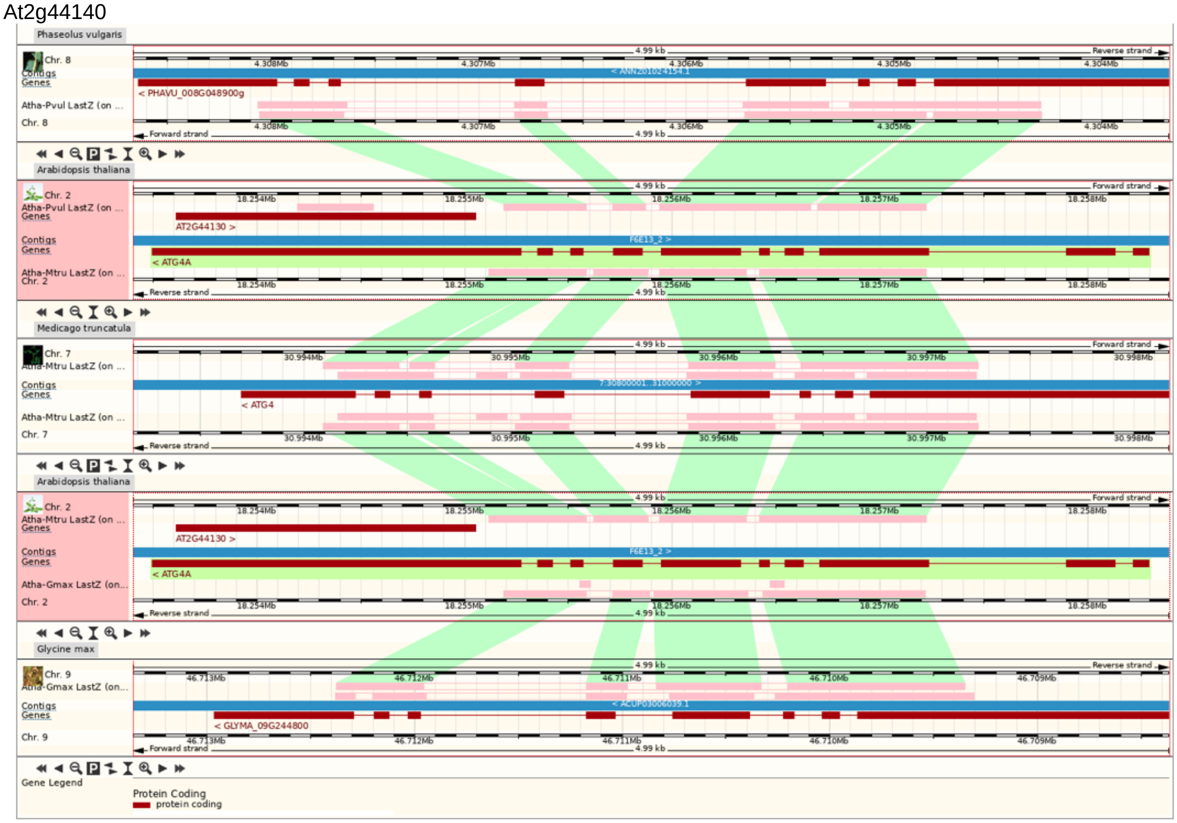

At3g59950

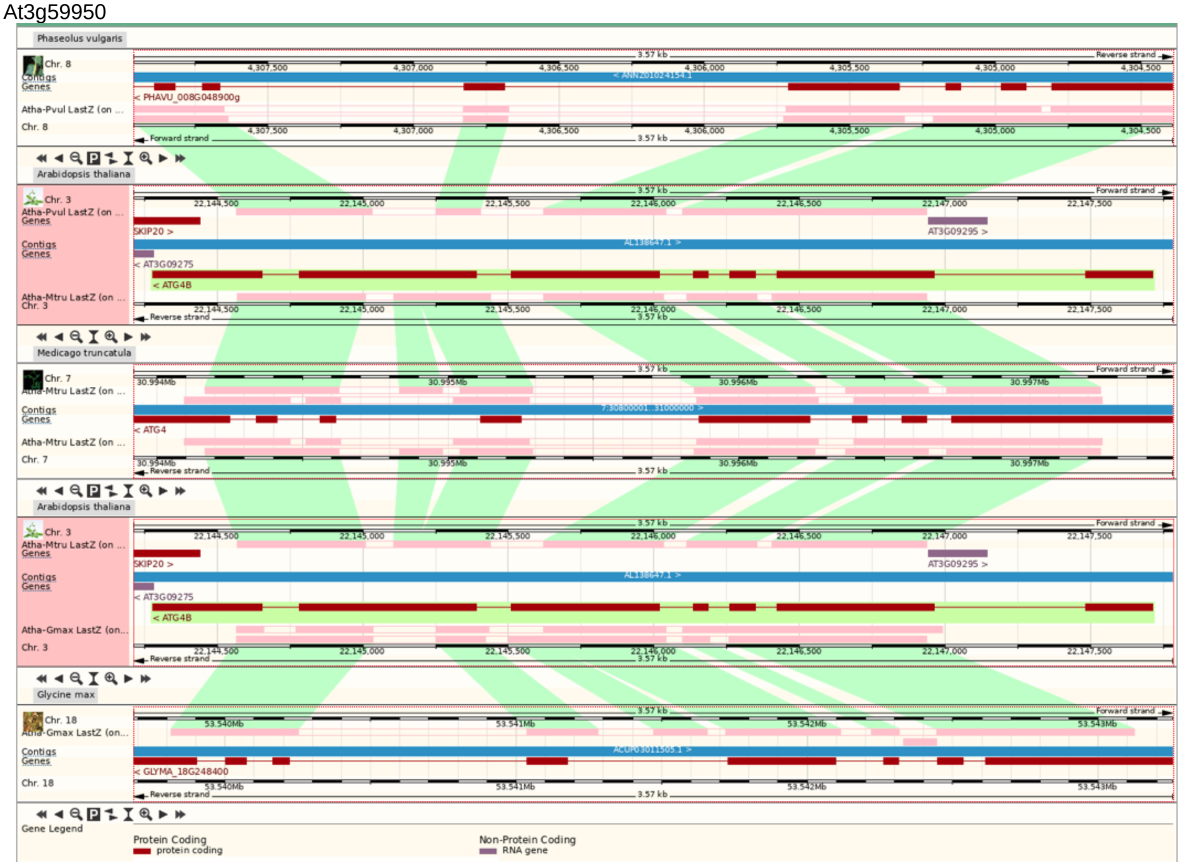

ATG5

At5g17290

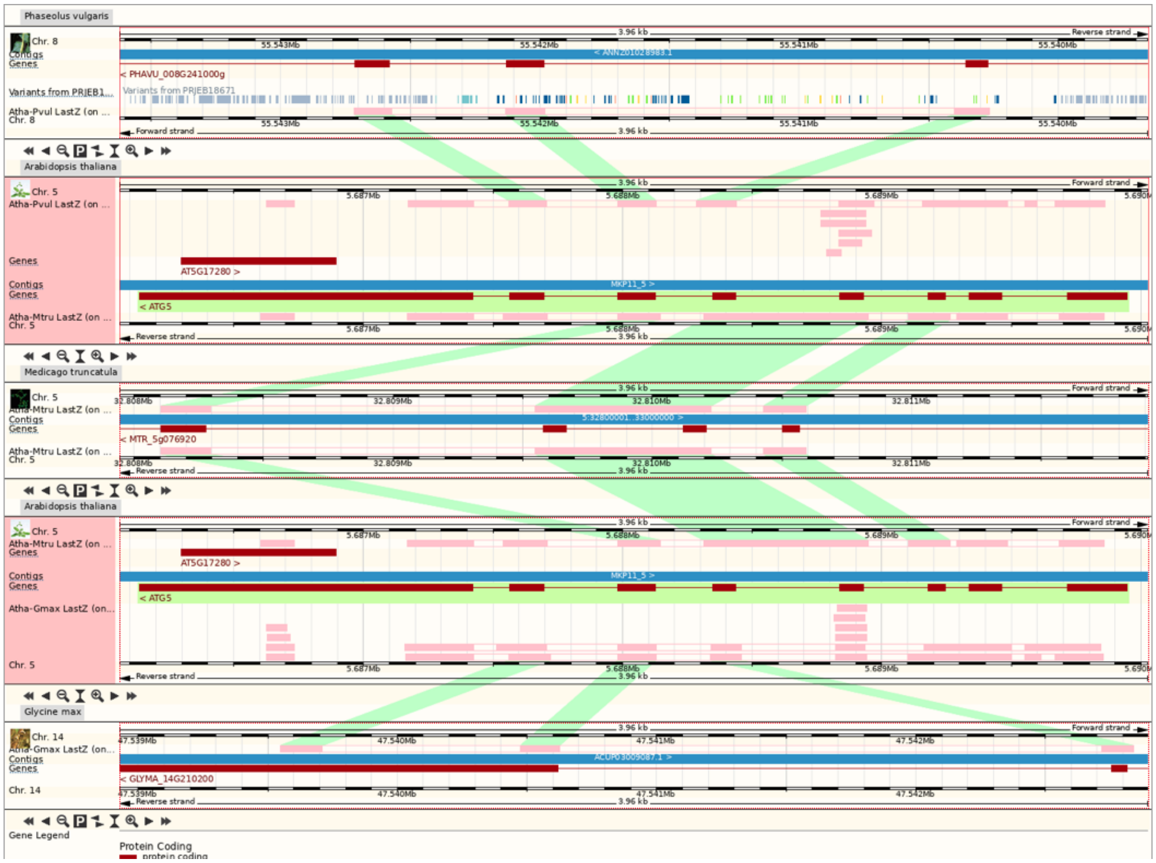

ATG6

At3g61710

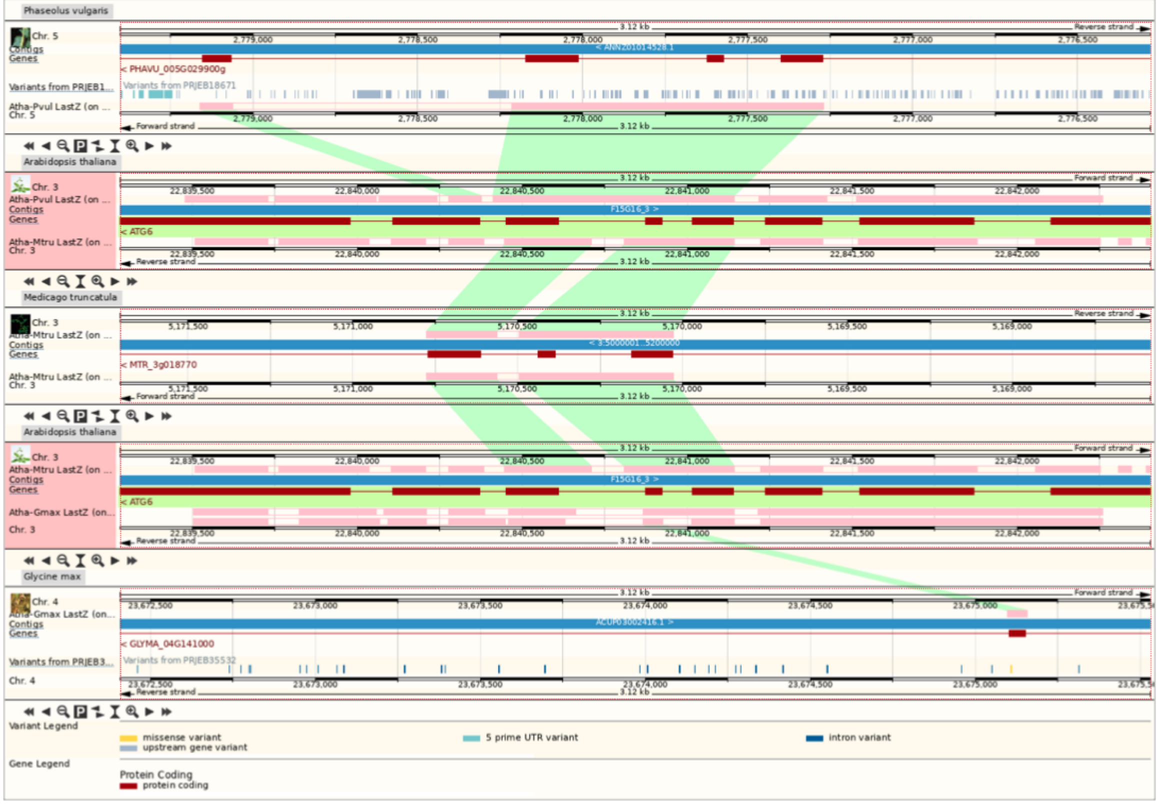

ATG7

At5g45900

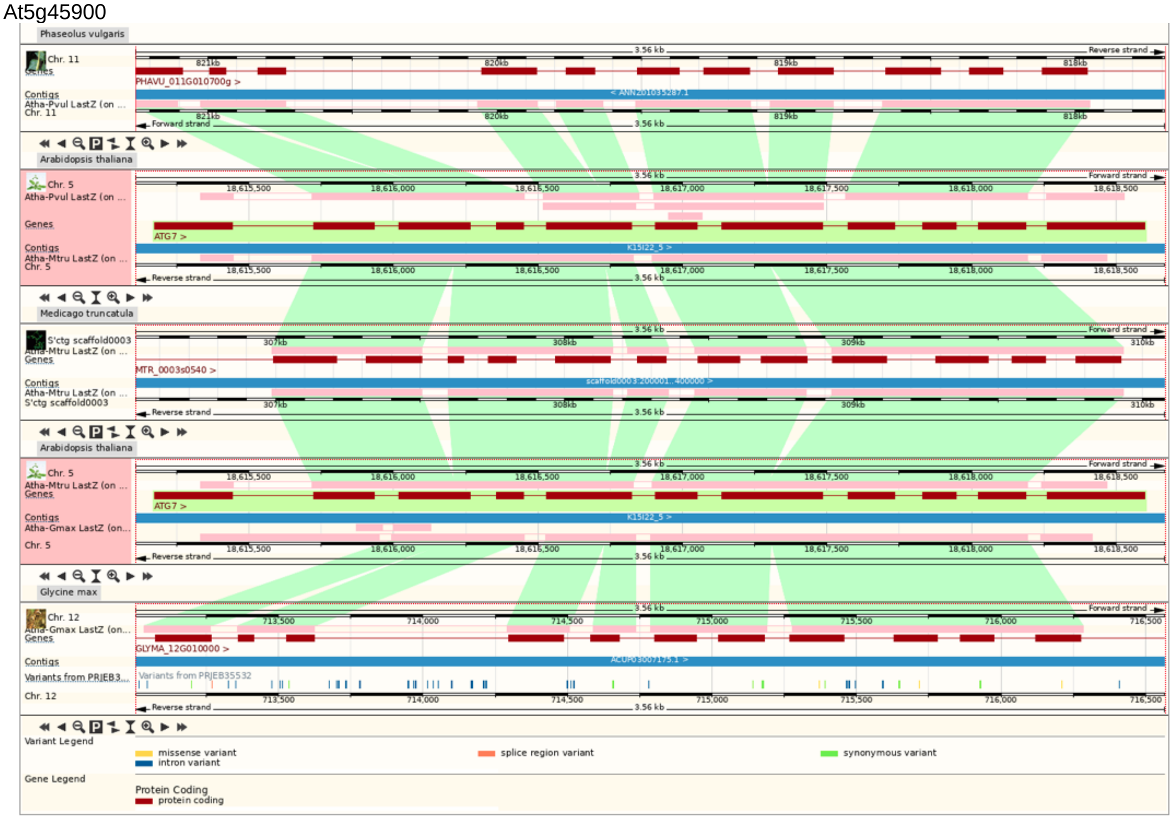

ATG8

At4g21980

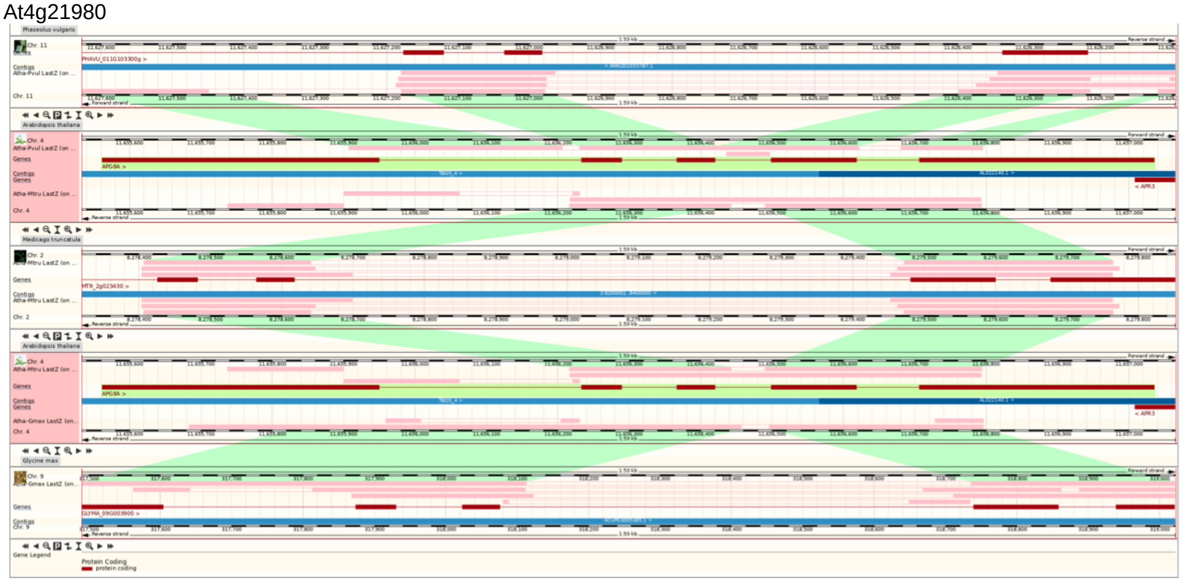

At4g04620

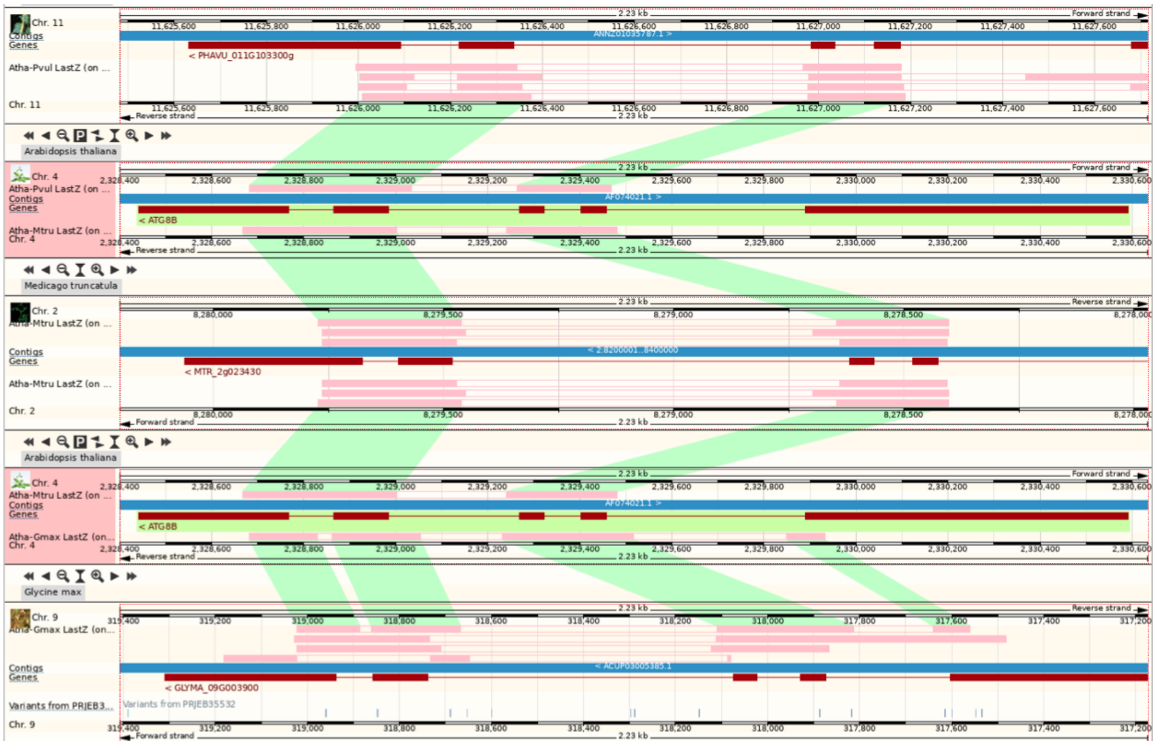

At1g62040

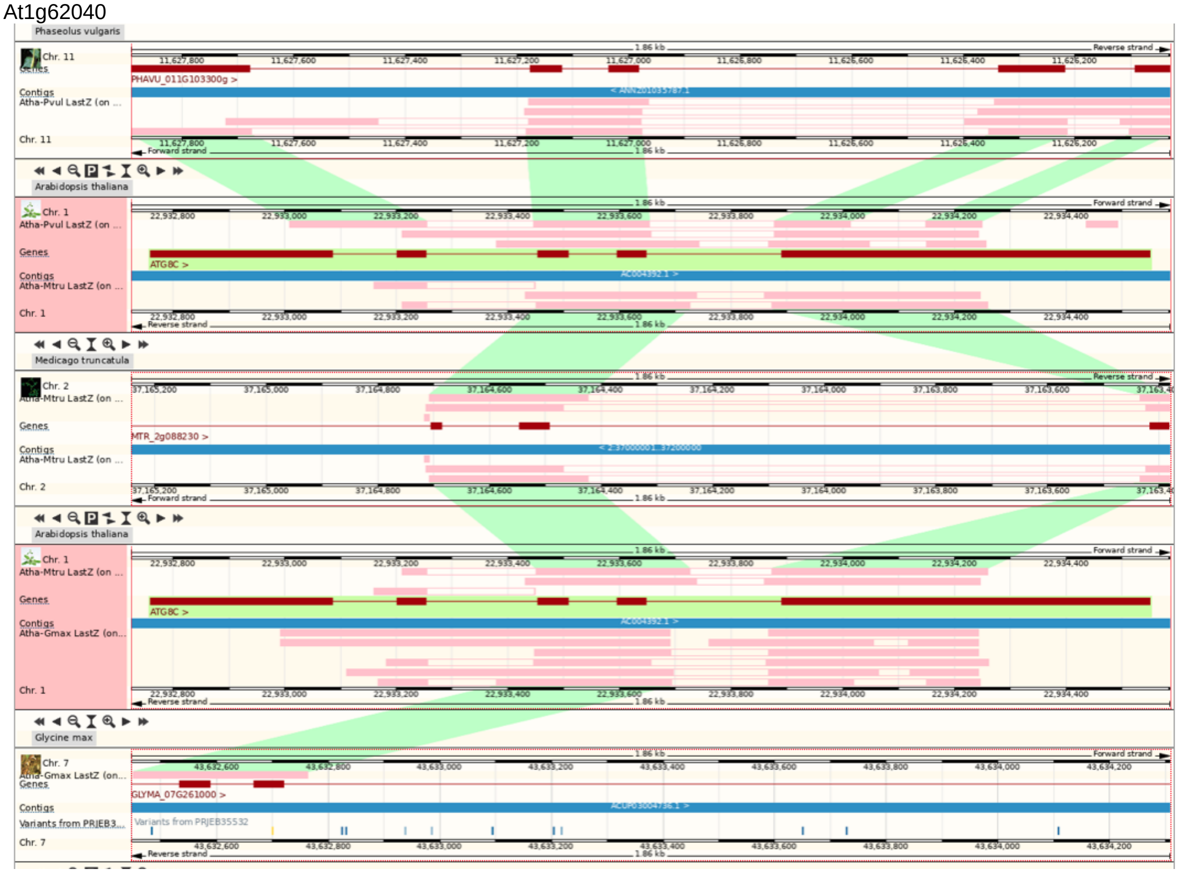

At2g05630

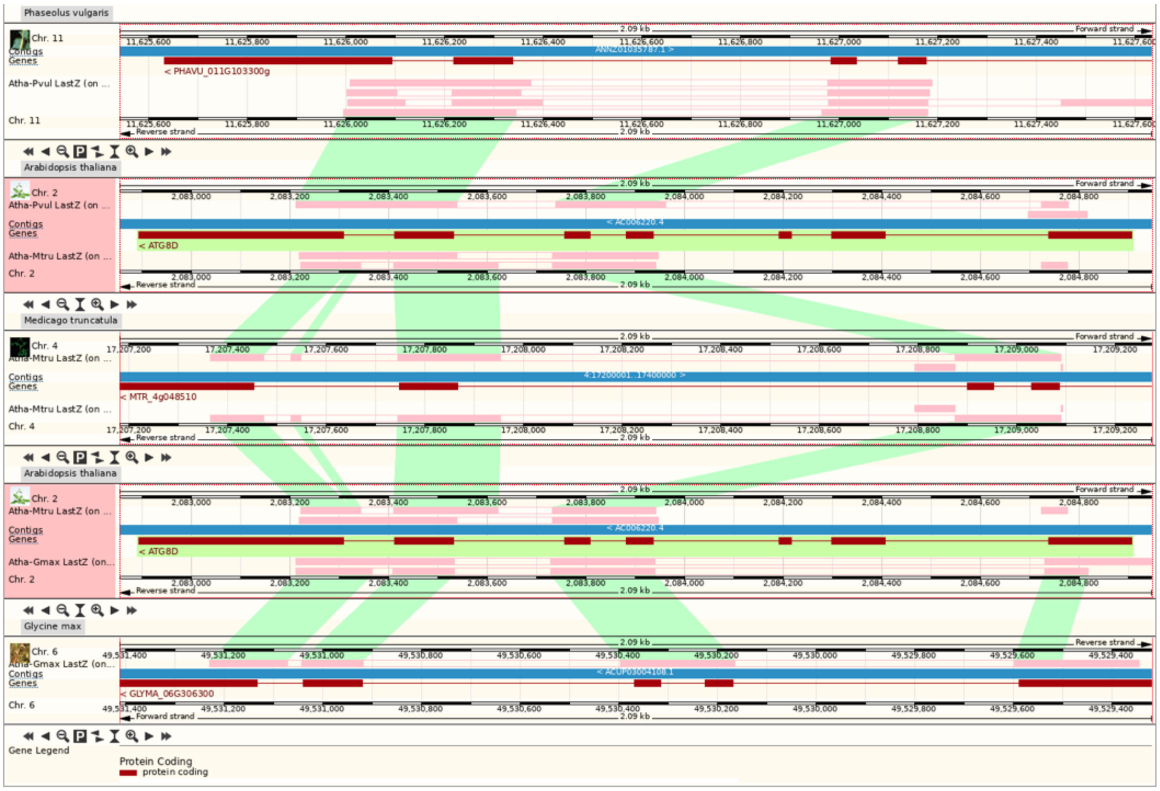

At2g45170

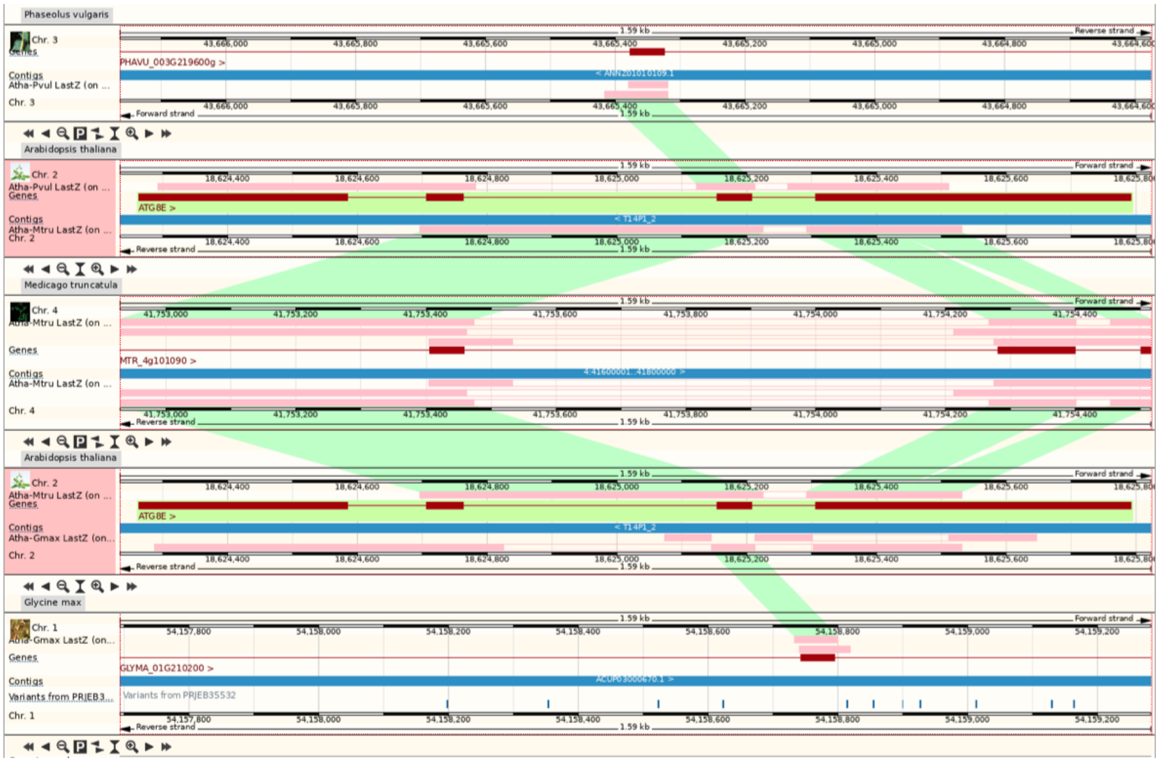

At4g16520

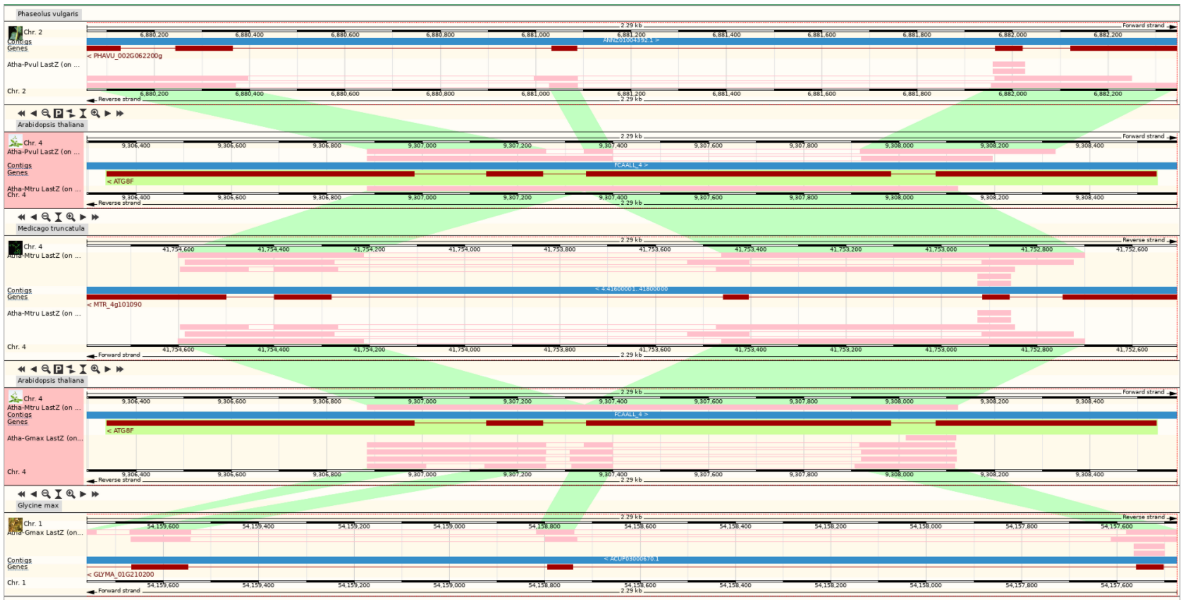

At3g60640

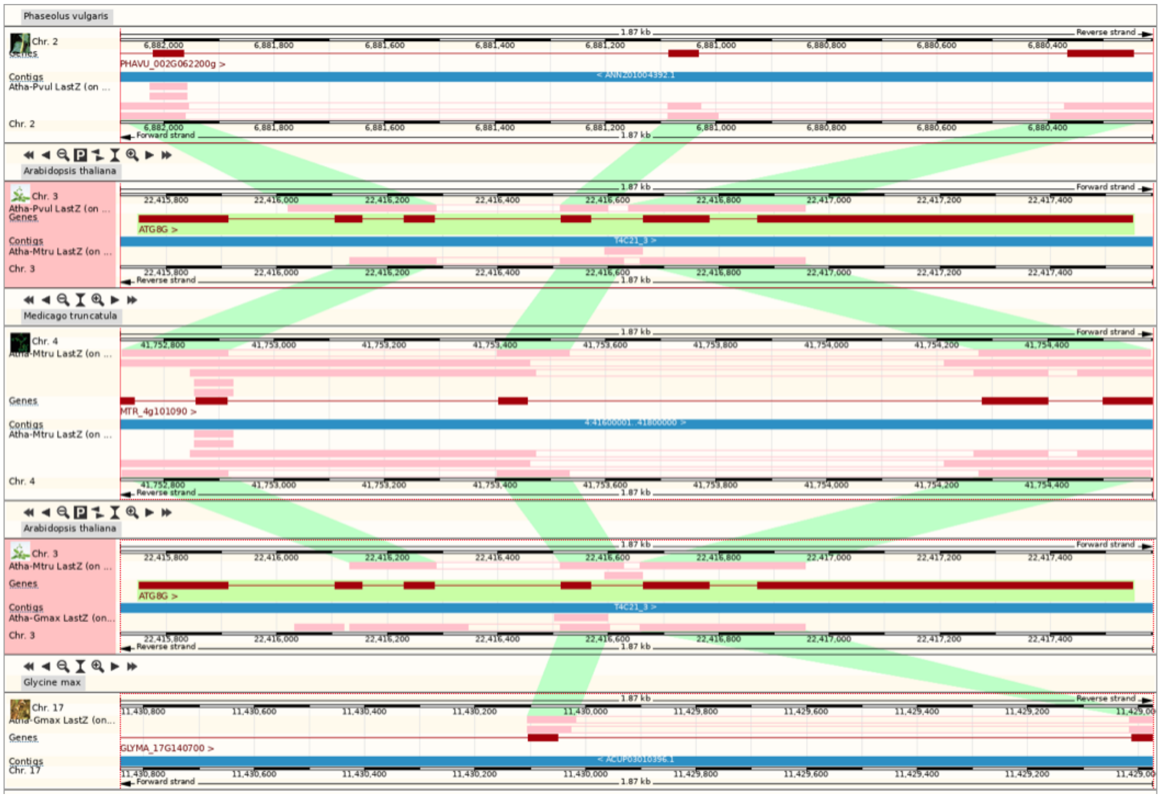

At3g06420

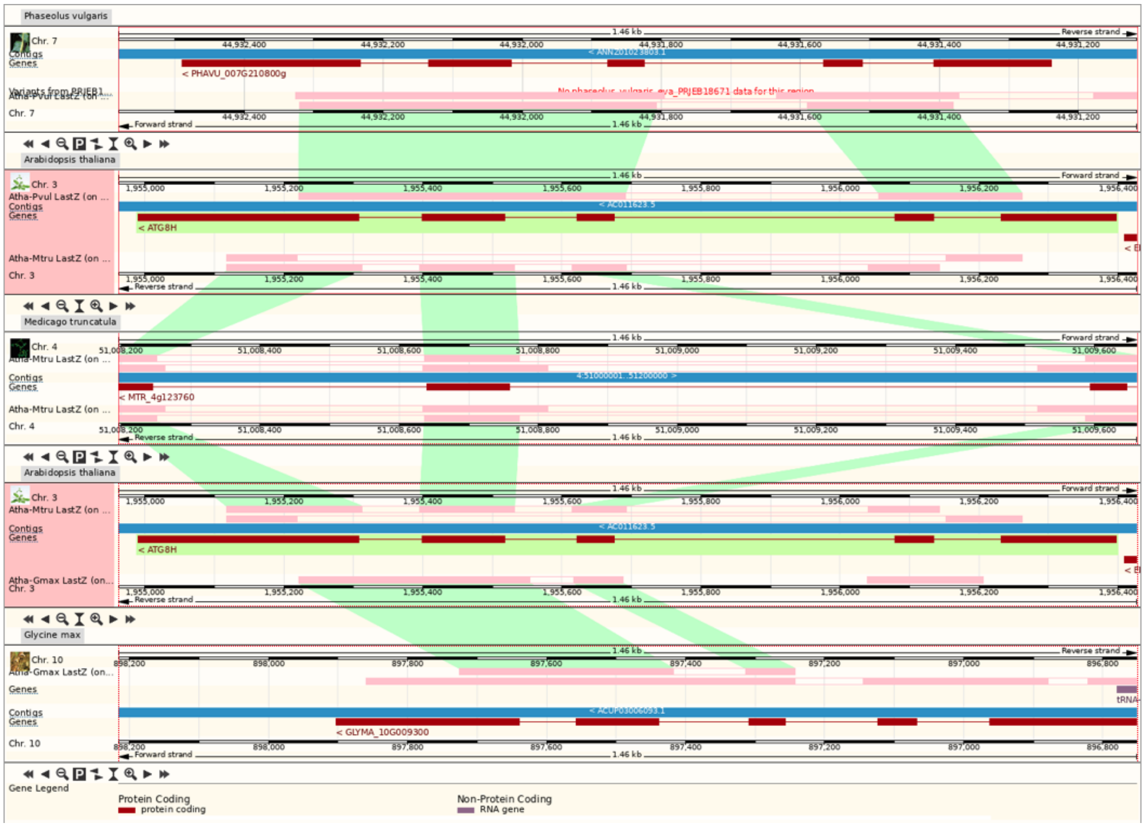

At3g15580

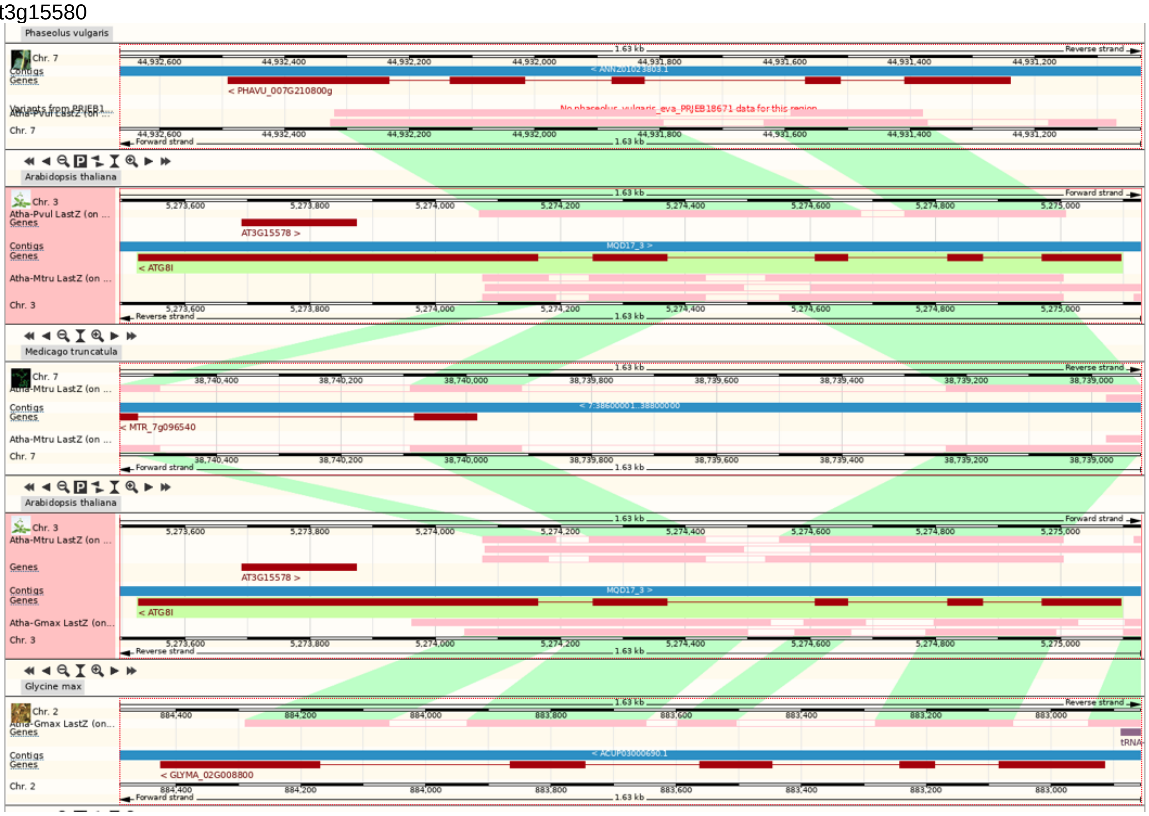

ATG9

At2g31260

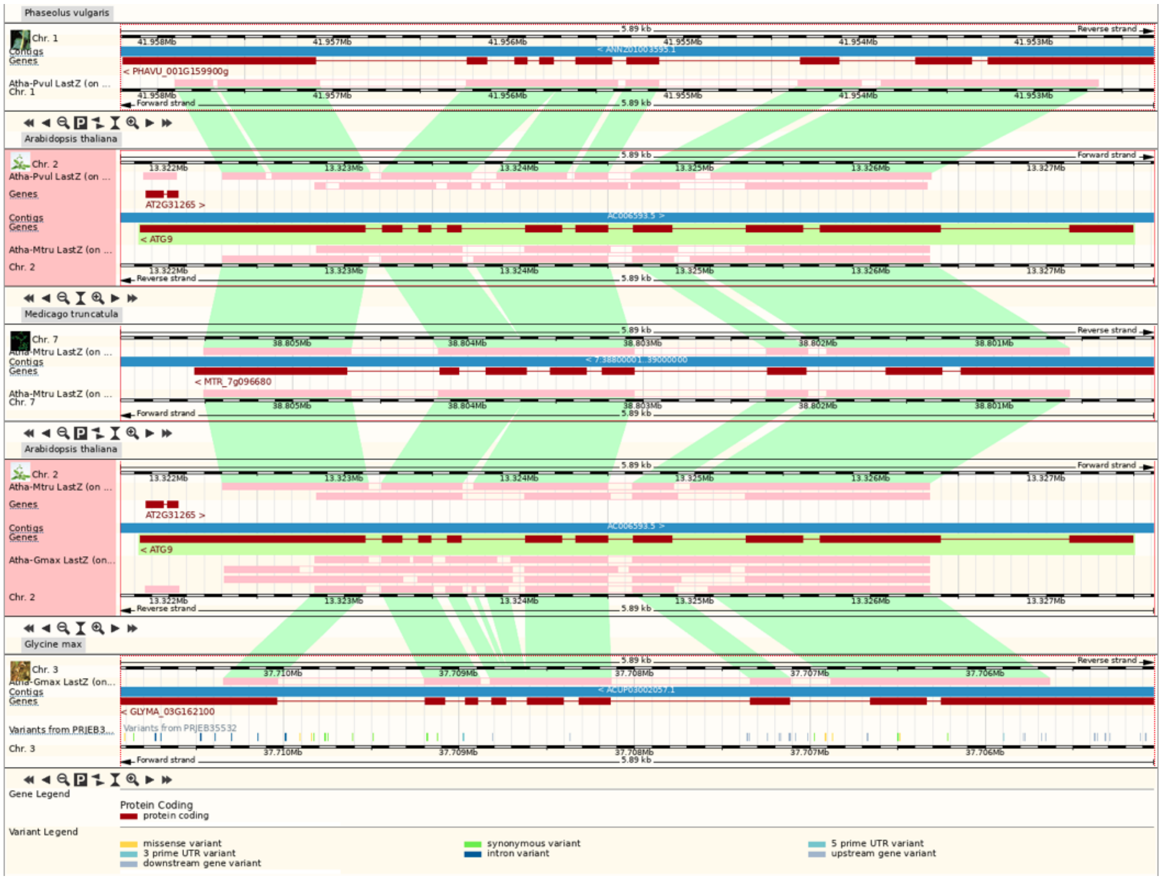

ATG10

At3g07525

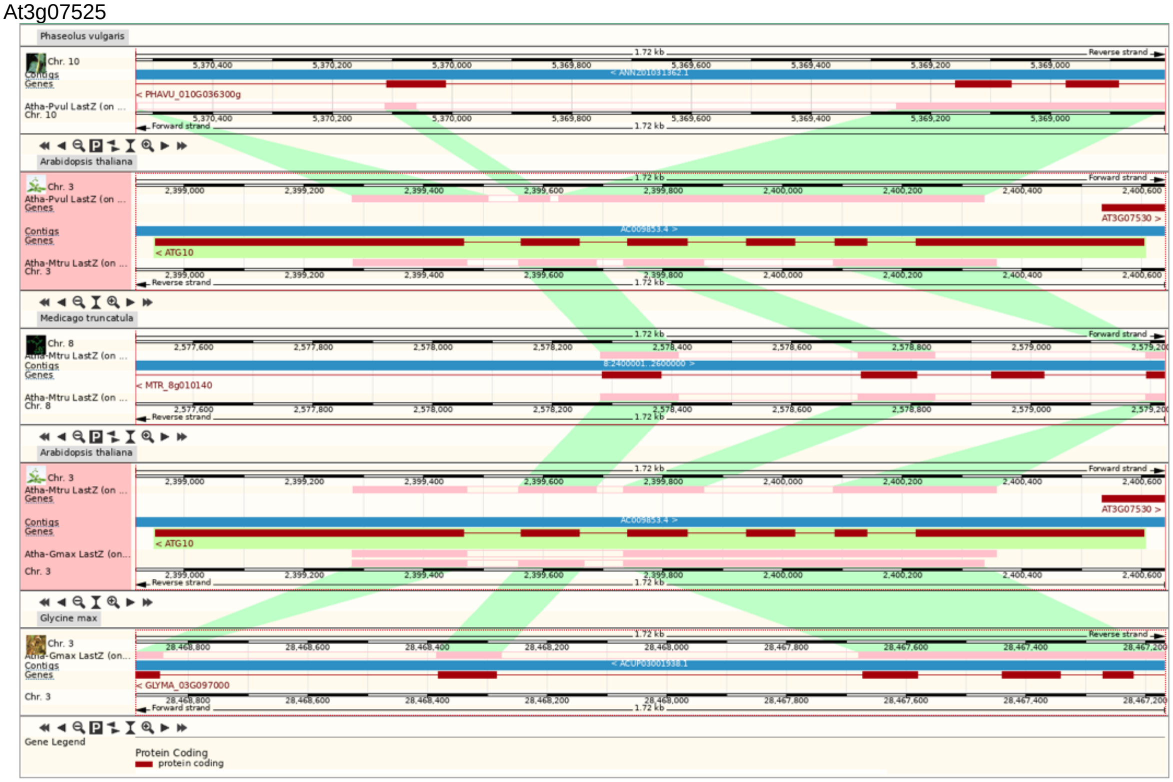

ATG11

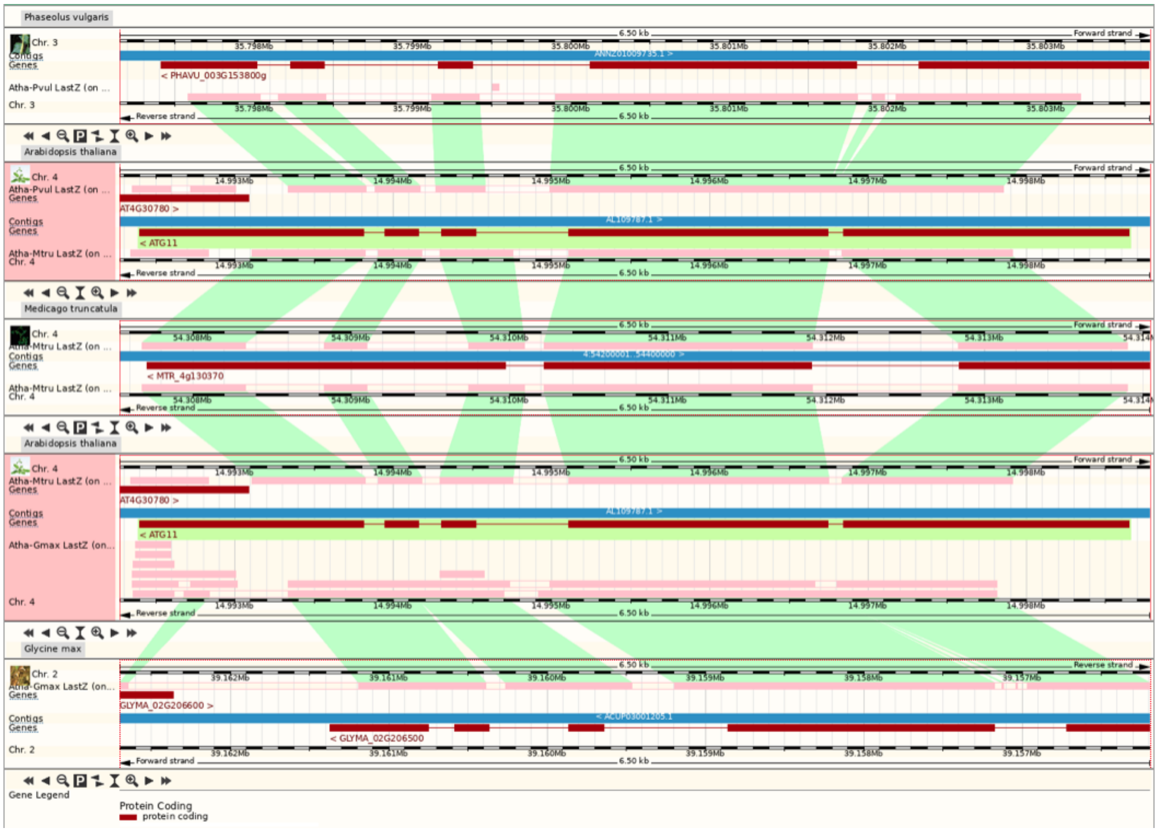

ATG12

At1g54210

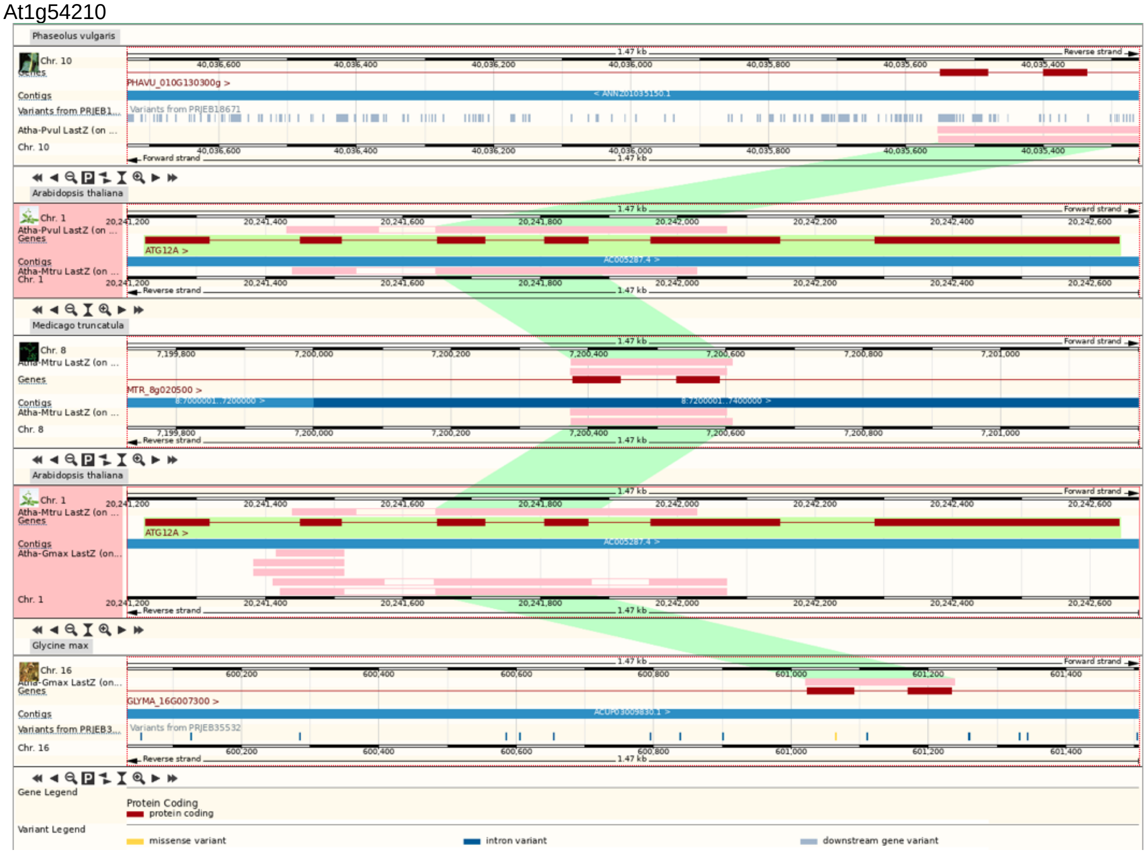

At3g13970

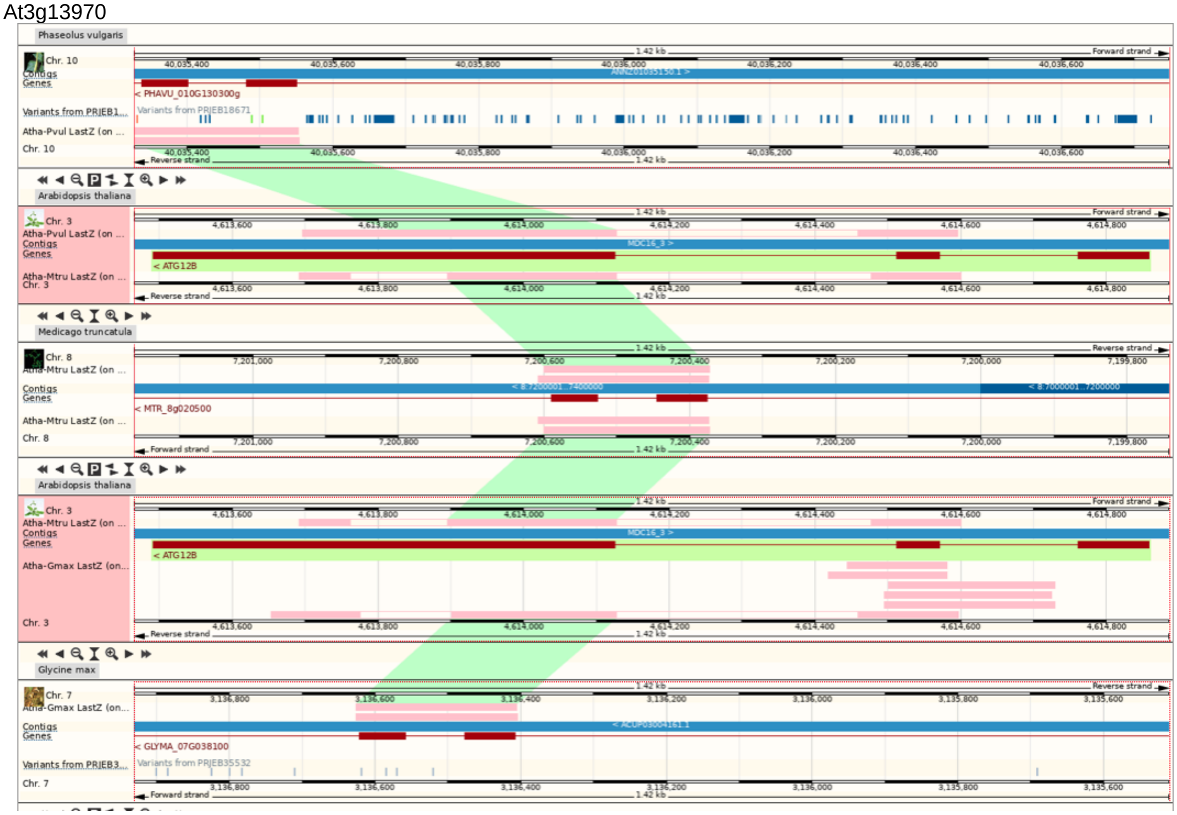

ATG13

At3g49590

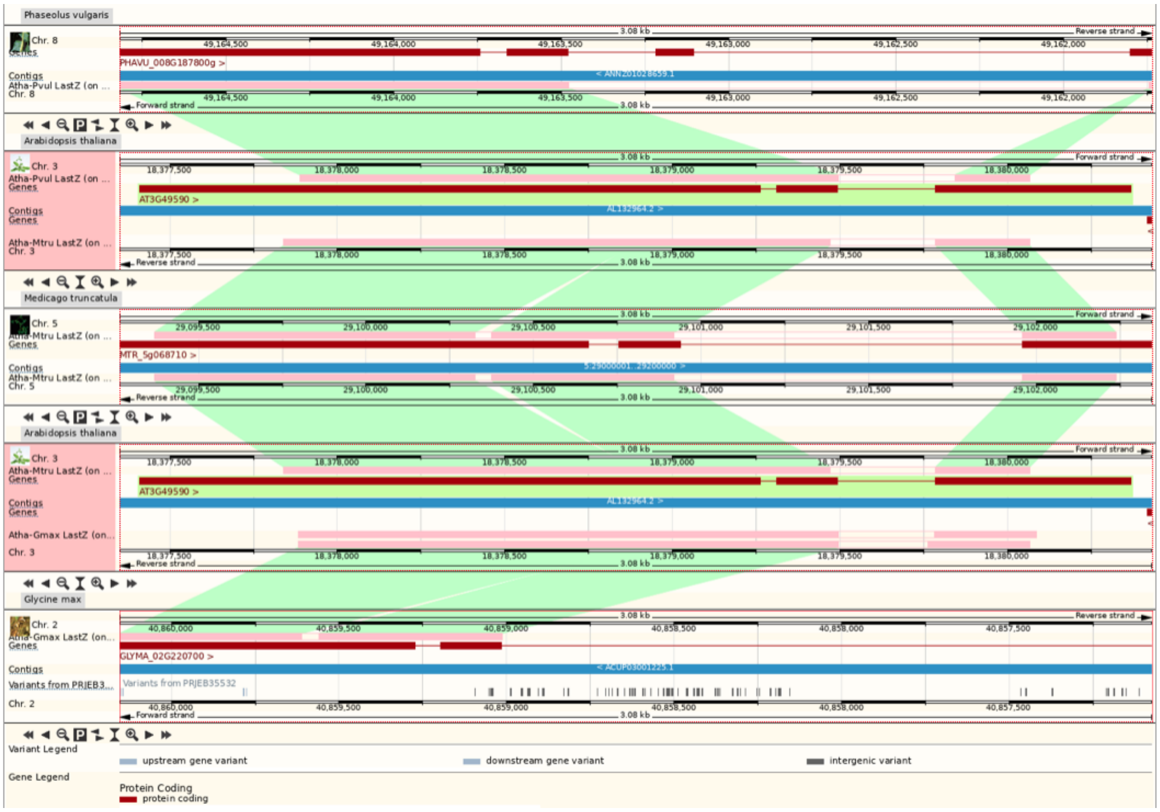

At3g18770

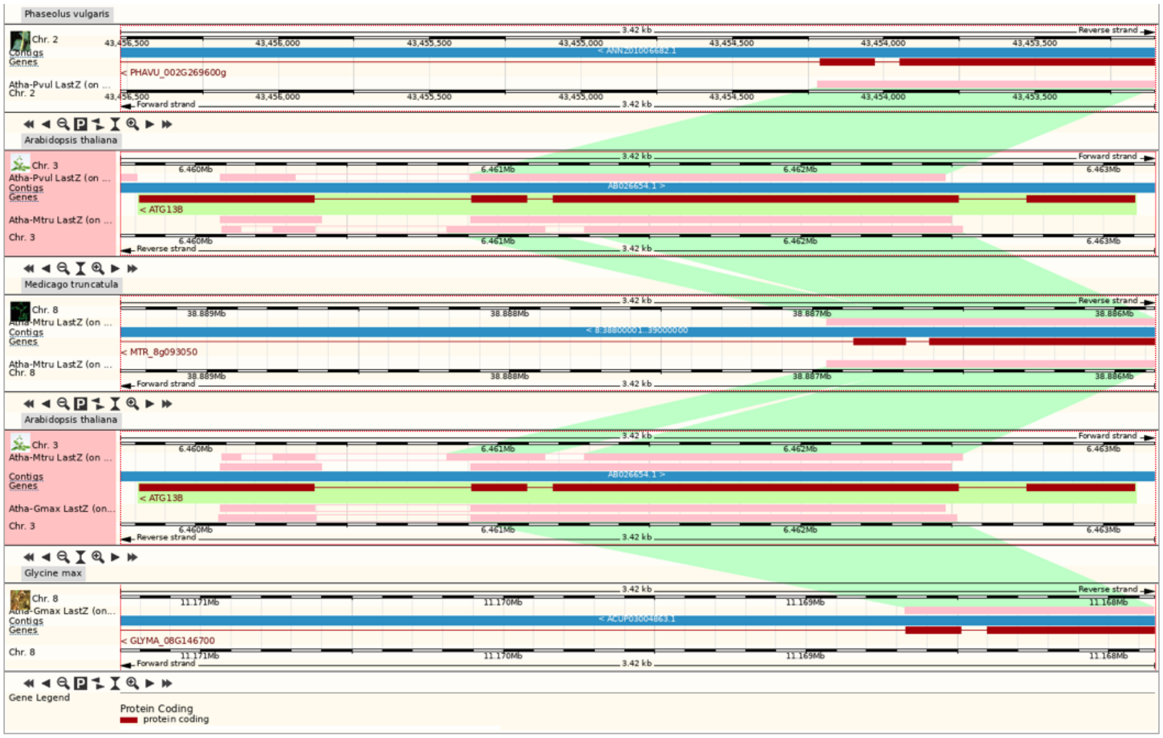

ATG14

At1g77890

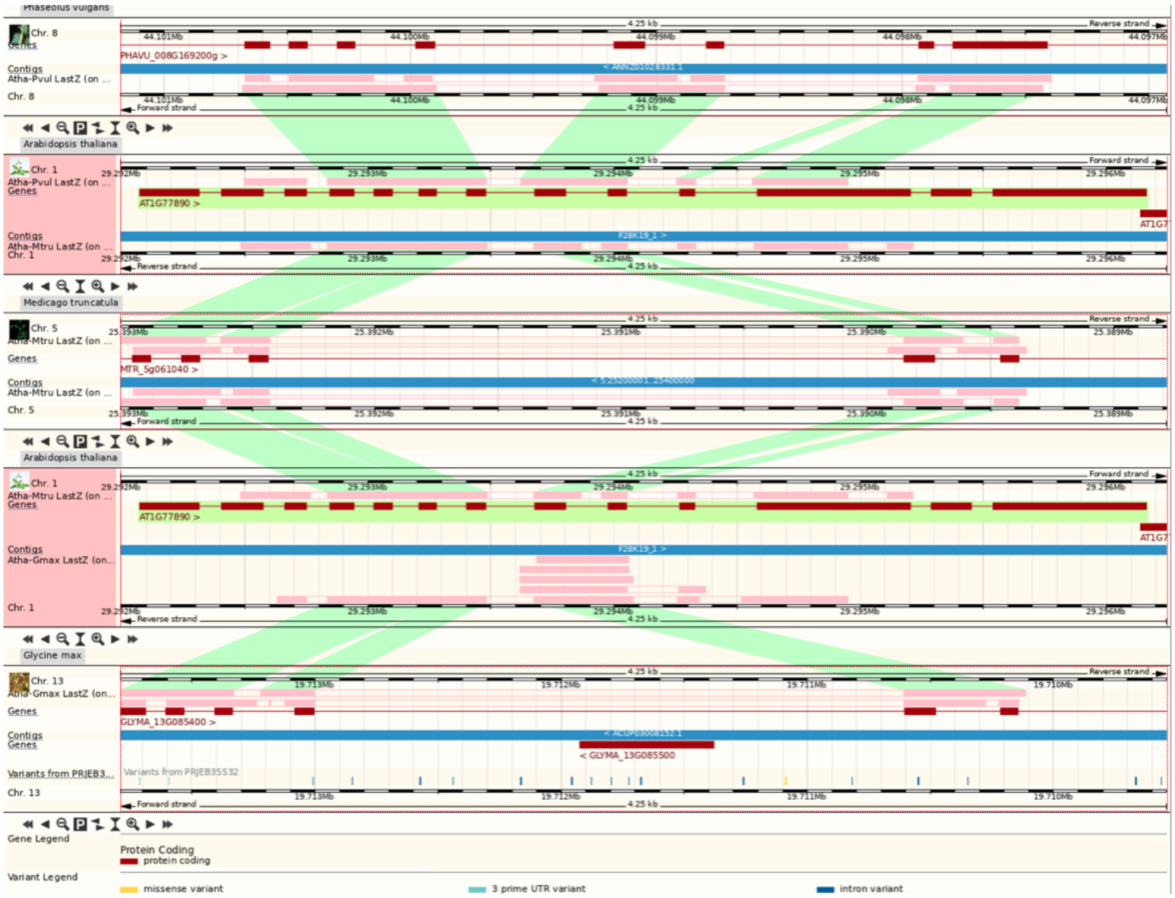

At4g08540

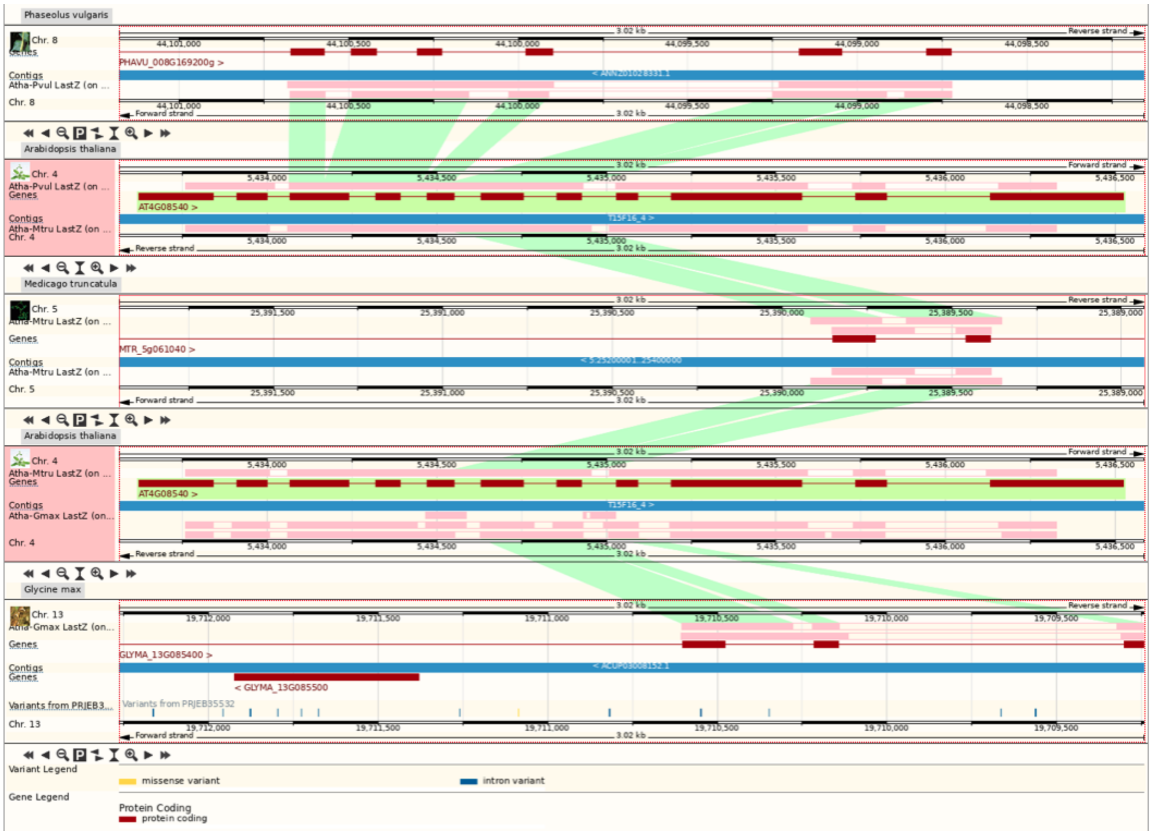

ATG16

At5g50230

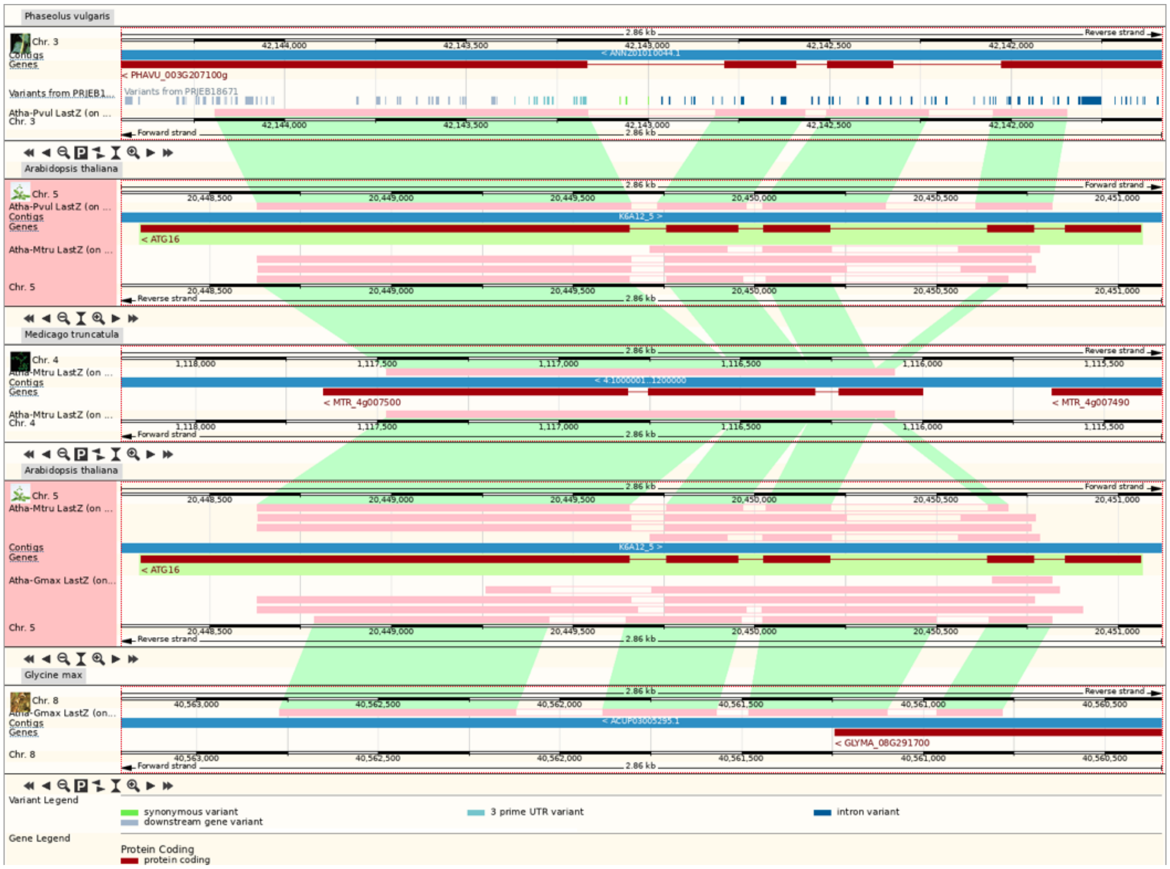

ATG18

At3g62770

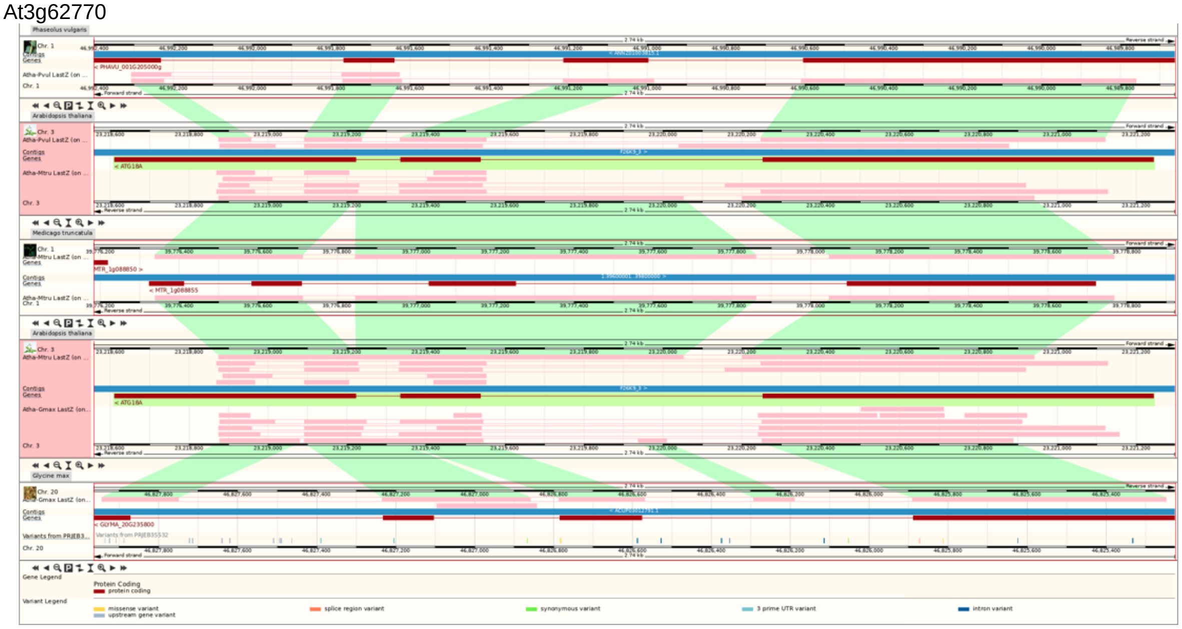

At4g30510

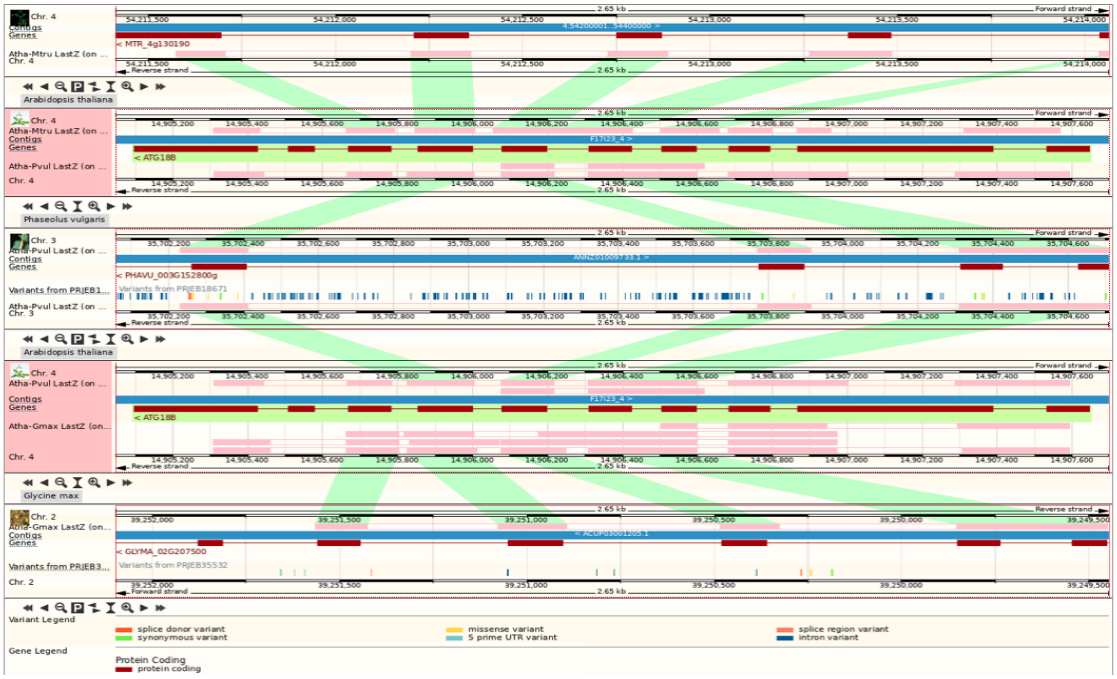

At4g30510

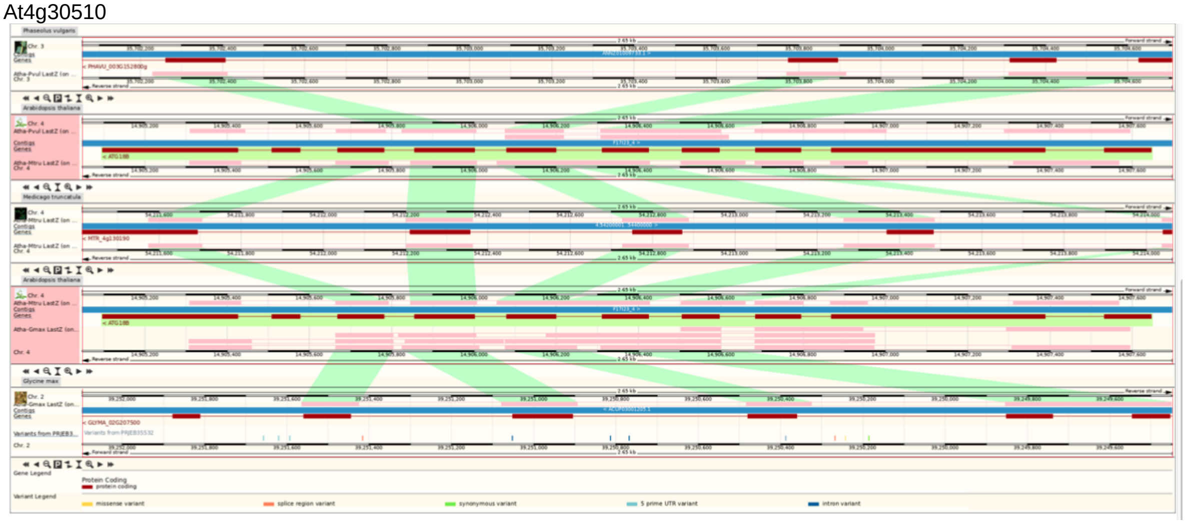
At2g40810

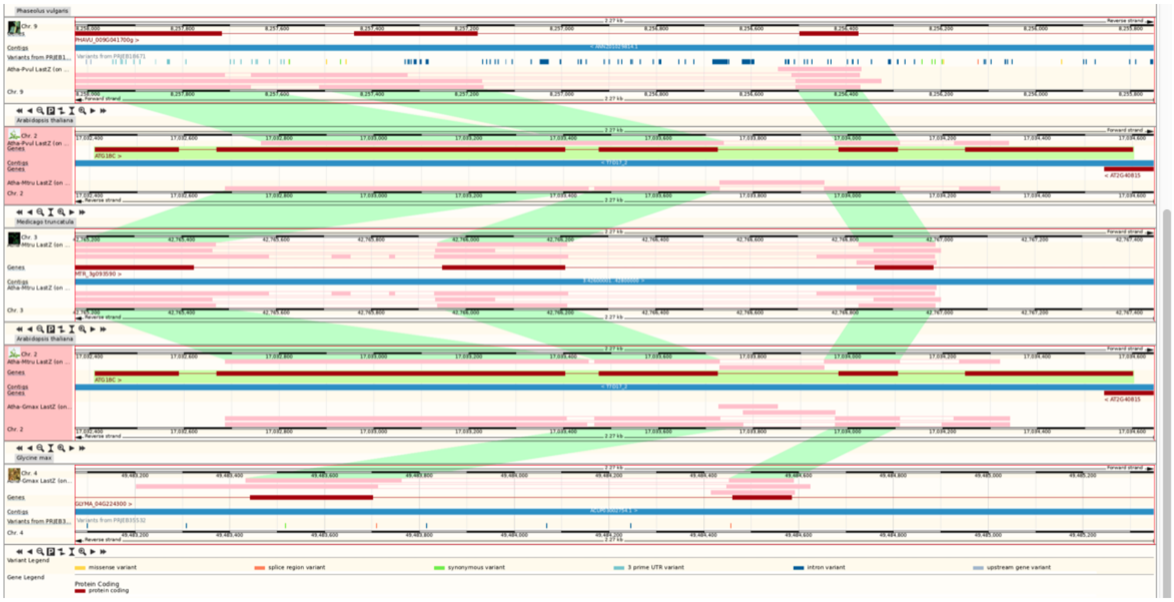

At3g56440

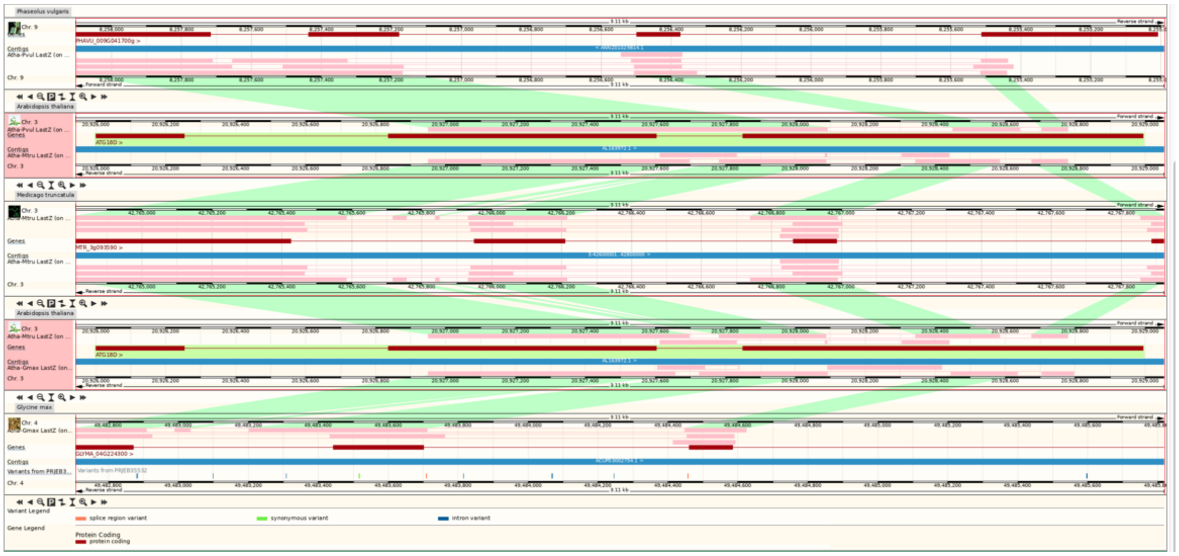

At5g05150

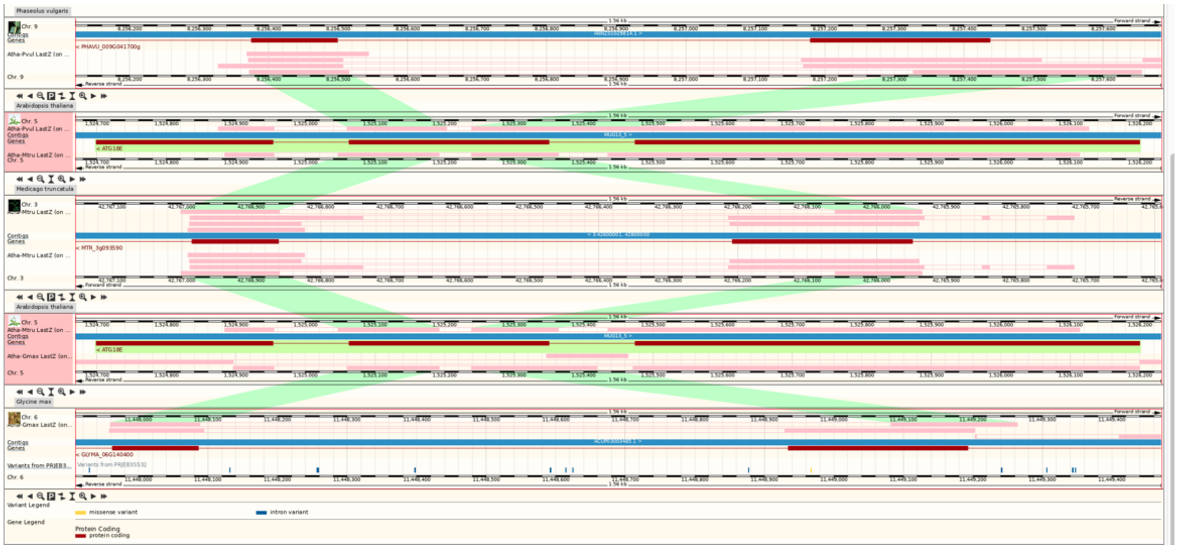

At5g54730

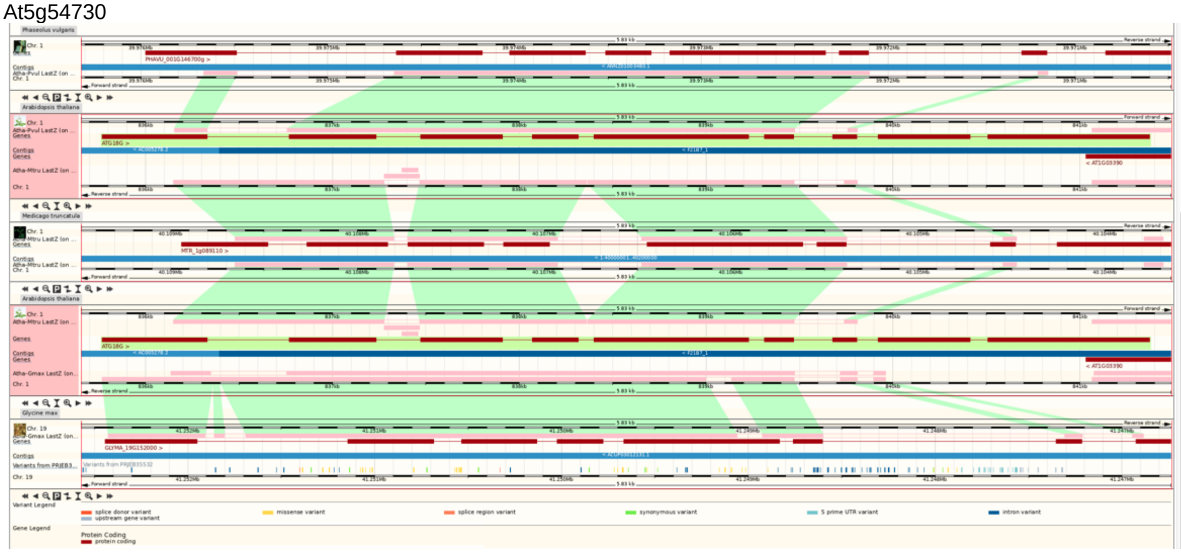

At1g54710

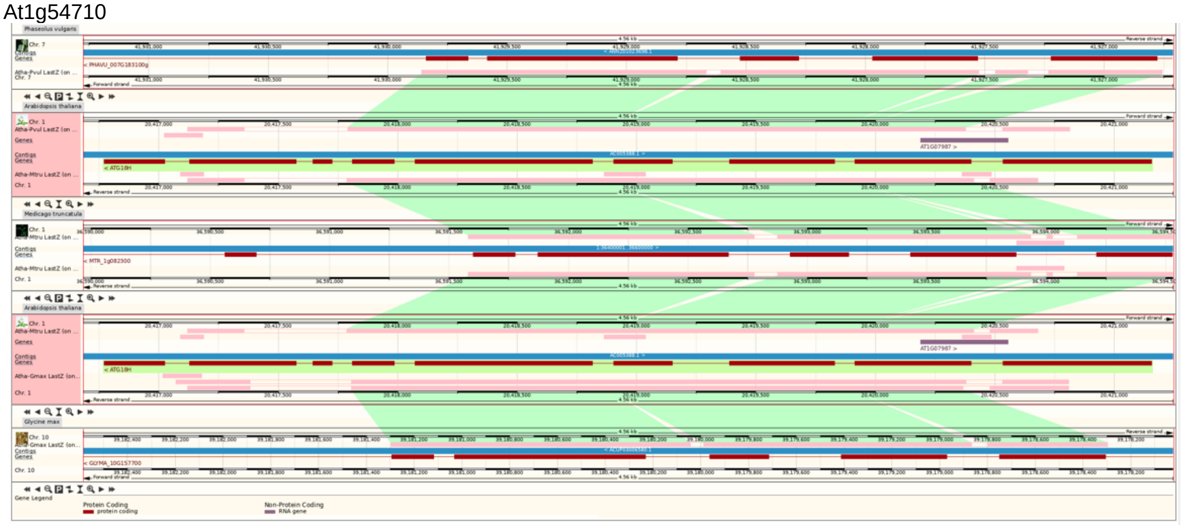

ATG101

At5g66930

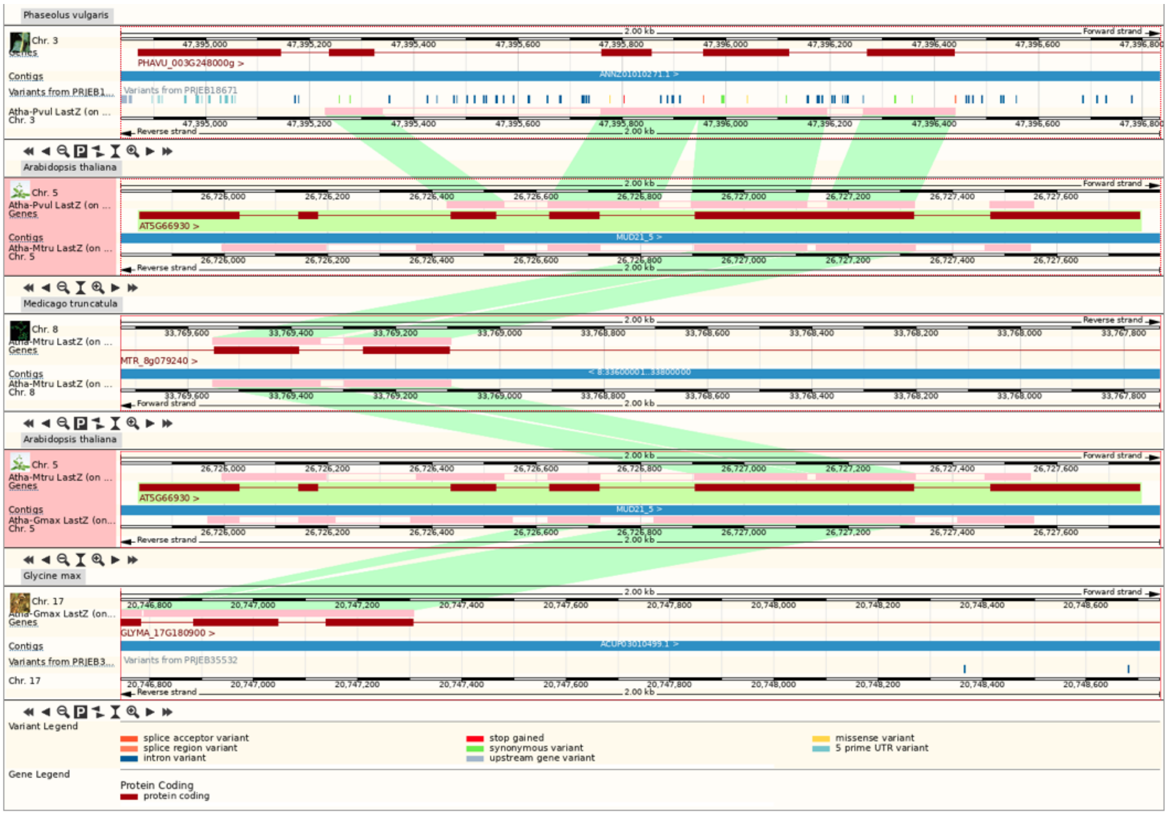

Phaseolus vulgaris
Chr. 3
Genes
PHAVU_003G248000g >
Contigs
Variants from PRJEB18671
Atha-Pvul LastZ (on ...
Forward strand
Reverse strand
2.00 kb
Arabidopsis thaliana
Chr. 5
AT5G66930 >
MUD21_5 >
Atha-Mtru LastZ (on ...
Medicago truncatula
Chr. 8
MTR_8g079240 >
Atha-Gmax LastZ (on...
Glycine max
Chr. 17
GLYMA_17G180900 >
Variants from PRJEB35532
Variant Legend
splice acceptor variant
splice region variant
intron variant
stop gained
synonymous variant
upstream gene variant
missense variant
5 prime UTR variant
Gene Legend
Protein Coding
protein coding

B. P. vulgaris Paralogous

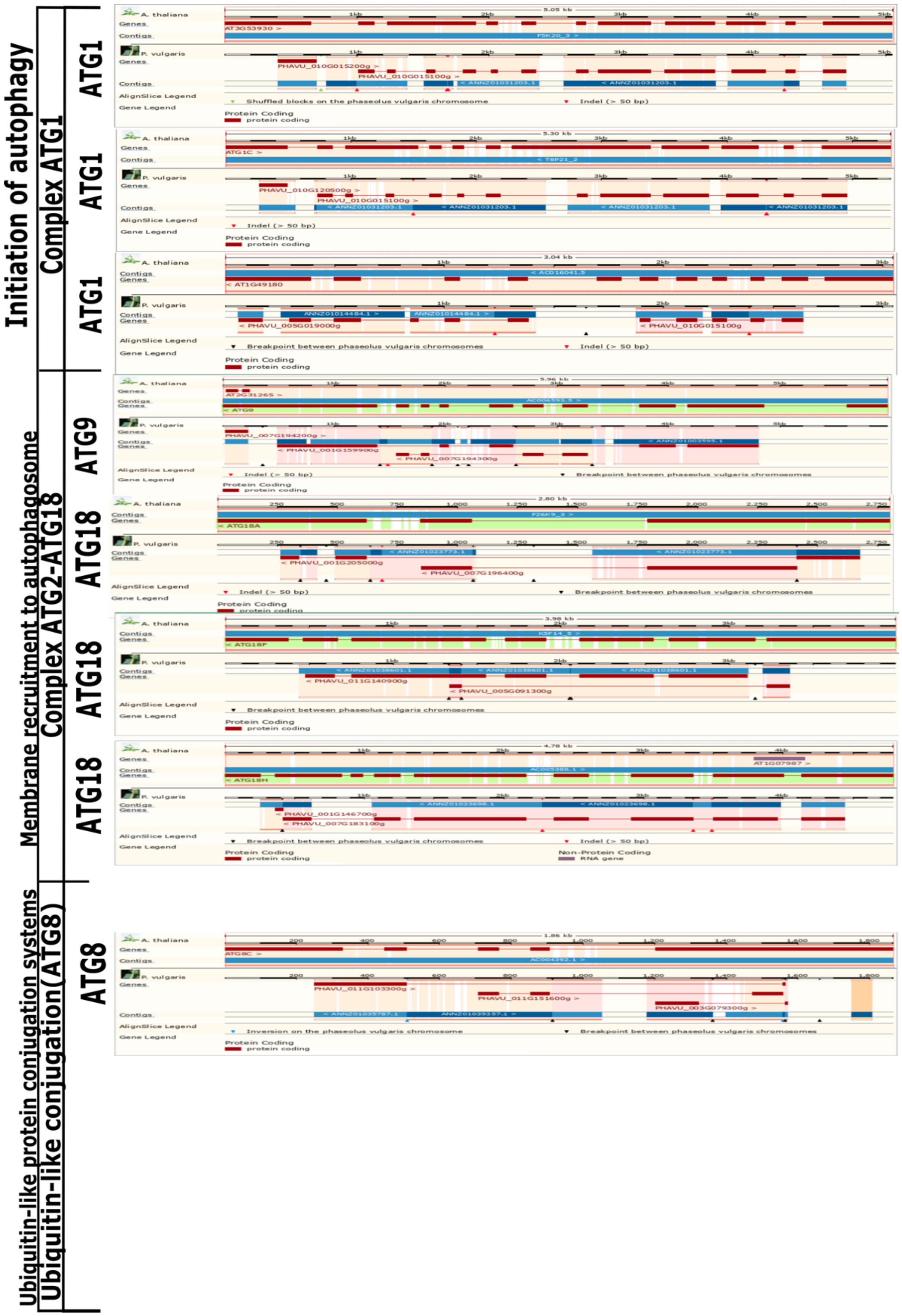

C. M. truncatula Paralogous

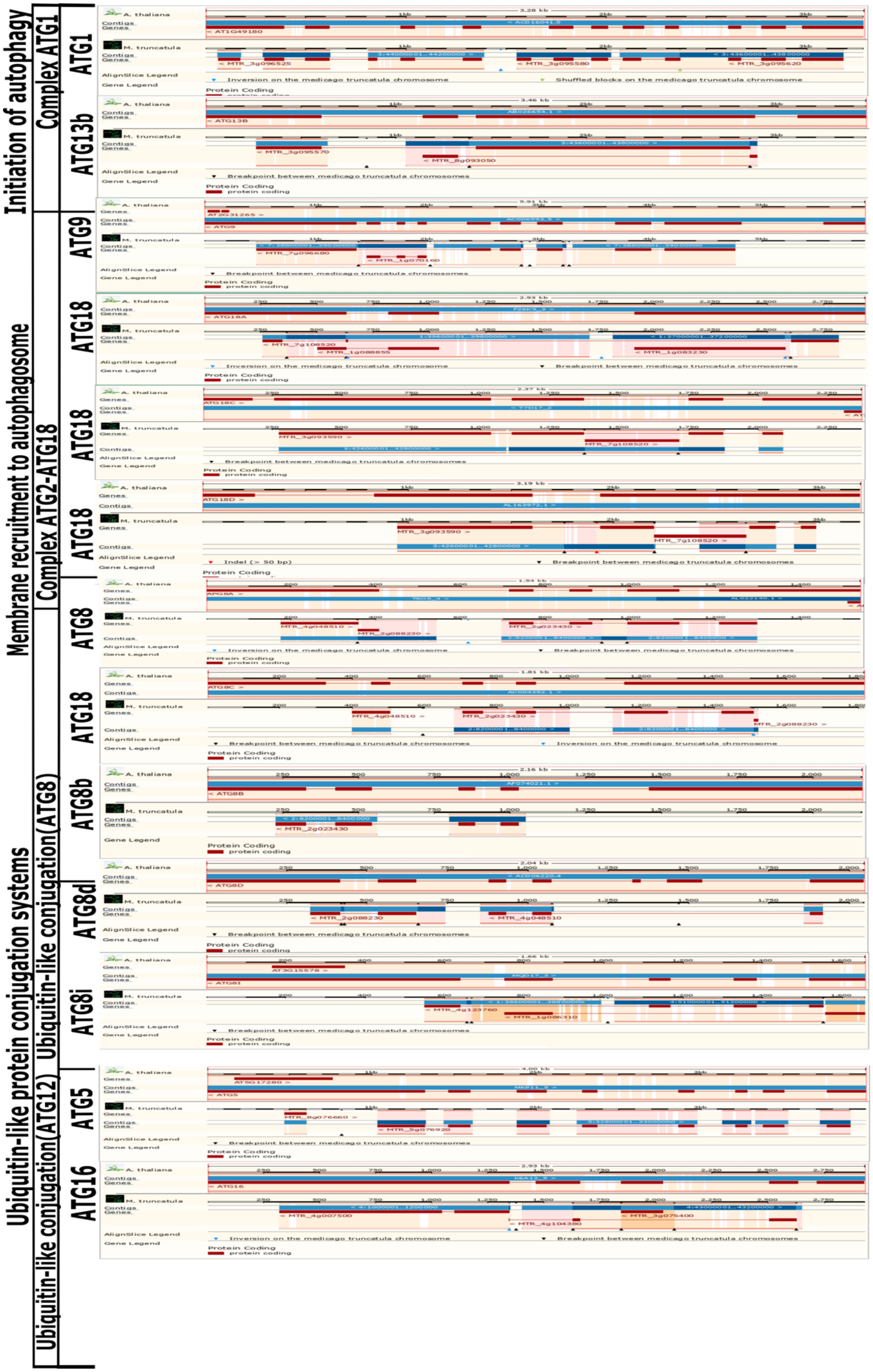

D. G.max Paralogous

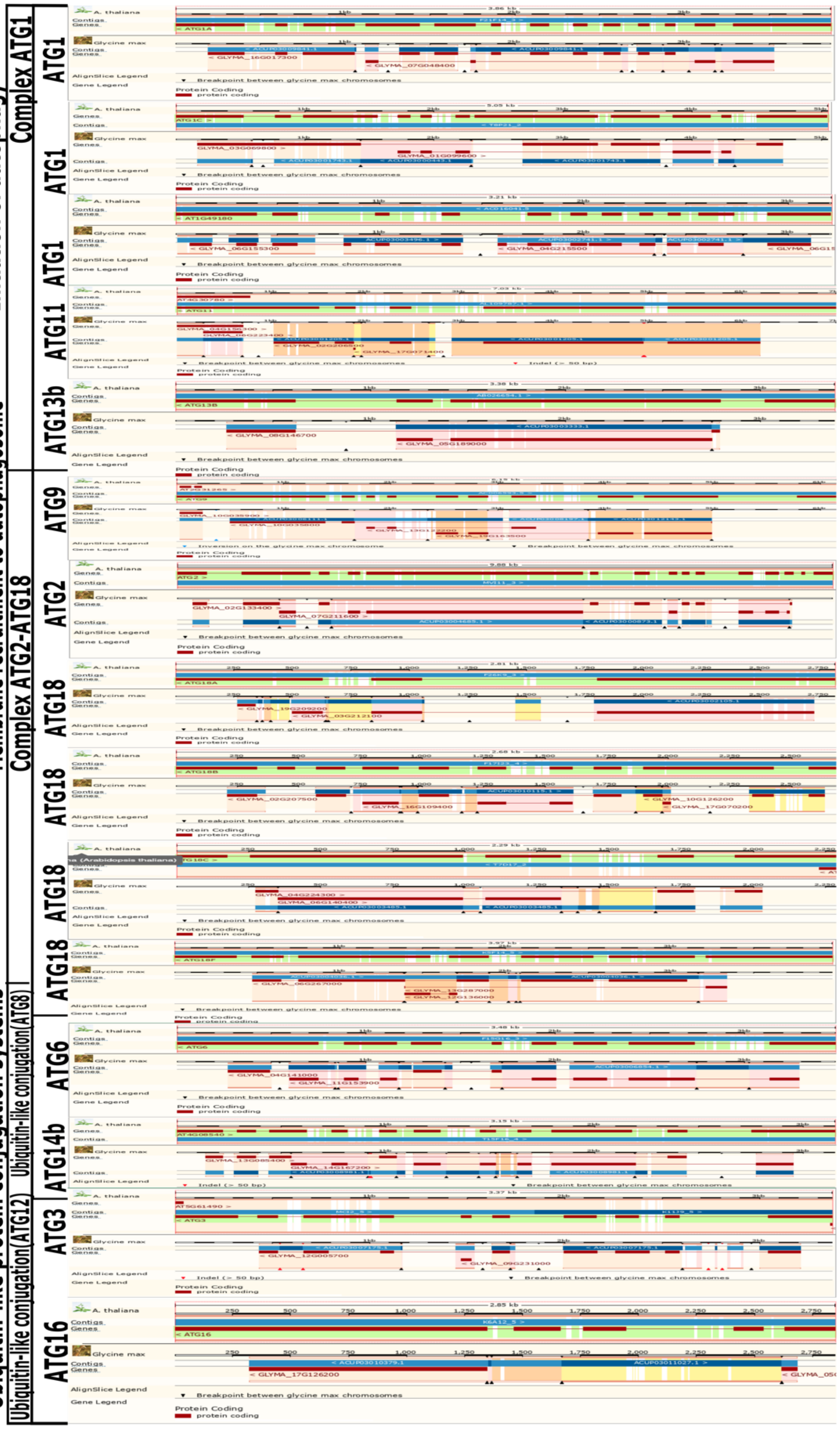

Initiation of autophagy
Complex ATG1
ATG1
ATG1
ATG1
ATG11
ATG13b
Membrane recruitment to autophagosome
Complex ATG2-ATG18
ATG9
ATG2
ATG18
ATG18
ATG18
ATG18
Ubiquitin-like protein conjugation systems
Ubiquitin-like conjugation (ATG8)
ATG6
ATG14b
Ubiquitin-like conjugation (ATG12)
ATG3
ATG16
