# Supplementary material for: Exploration of Autophagy Families in Legumes and Dissection of the ATG18 Family with a Special Focus on Phaseolus vulgaris
Source: Plants (Basel). 2021 Nov 29;10(12):2619. doi: 10.3390/plants10122619 (PMC8703869; doi:10.3390/plants10122619)
Supplement: Supplementary file 1 [file plants-10-02619-s001.zip › Supplementary tables .pdf]

Supplemental Table S1. List of identificators of Gen, transcript and Protein of each ATG genes in (A) *A. thaliana, (B) P. vulgaris, (C) M. truncatula and (D) G.max.*

A.1 List of identificators of Gen, gene localization and length of each ATG genes in *A. thaliana.*

Gene accession numbers	*Gene Length*	*Gene Chromosome Primary Identifier*	*Gene location*	*Gene location*
AT1G49180	2876	Chr1	18184654	18187529
AT2G37840	4499	Chr2	15851803	15856301
AT3G53930	4462	Chr3	19966352	19970813
AT3G61960	3517	Chr3	22941772	22945288
AT3G19190	9147	Chr3	6639711	6648857
AT5G61500	3069	Chr5	24733785	24736853
AT2G44140	4539	Chr2	18253497	18258035
AT3G59950	3412	Chr3	22144280	22147691
AT5G17290	2956	Chr5	5686945	5689900
AT3G61710	3118	Chr3	22839315	22842432
AT5G45900	3339	Chr5	18615222	18618560
AT4G21980	1446	Chr4	11655589	11657034
AT4G04620	1752	Chr4	2328449	2330200
AT1G62040	1790	Chr1	22932760	22934549
AT2G05630	1897	Chr2	2082986	2084882
AT2G45170	1454	Chr2	18624264	18625717
AT4G16520	1800	Chr4	9306663	9308462
AT3G60640	1324	Chr3	22415835	22417158
AT3G06420	1408	Chr3	1954990	1956397
AT3G15580	1201	Chr3	5273895	5275095
AT2G31260	5380	Chr2	13322021	13327400
AT3G07525	1315	Chr3	2399123	2400437
AT4G30790	4700	Chr4	14993086	14997785
AT1G54210	1390	Chr1	20241226	20242615
AT3G13970	1343	Chr3	4613506	4614848
AT3G49590	2806	Chr3	18377479	18380284
AT3G18770	3176	Chr3	6459884	6463059
AT1G77890	4038	Chr1	29292123	29296160
AT4G08540	2880	Chr4	5433645	5436524
AT5G50230	2512	Chr5	20448370	20450881
AT3G62770	2600	Chr3	23218633	23221232
AT4G30510	2456	Chr4	14905137	14907592
AT2G40810	2137	Chr2	17032464	17034600
AT3G56440	2988	Chr3	20926001	20928988
AT5G05150	1359	Chr5	1524841	1526199
AT5G54730	3553	Chr5	22233789	22237341
AT1G03380	5156	Chr1	835785	840940
AT1G54710	4266	Chr1	20416770	20421035
AT5G66930	1920	Chr5	26725839	26727758
AT1G50030	17555	Chr1	18522441	18539995
AT3G08850	8718	Chr3	2686583	2695300
AT2G22040	1823	Chr2	9374438	9376260

A.2 List of transcript accession and length of each ATG genes genes in *A. thaliana,*

Gene accession numbers	*Transcript accession numbers*	*Transcript Gene Length*
AT1G03380	AT1G03380.1	3732
AT1G49180	AT1G49180.1	1498
AT1G49180	AT1G49180.2	1376
AT1G50030	AT1G50030.1	8007
AT1G50030	AT1G50030.2	7926
AT1G54210	AT1G54210.1	631
AT1G54210	AT1G54210.2	570
AT1G54710	AT1G54710.1	3335
AT1G62040	AT1G62040.1	745
AT1G62040	AT1G62040.2	720
AT1G77890	AT1G77890.1	2275
AT1G77890	AT1G77890.2	2242
AT1G77890	AT1G77890.3	1818
AT2G05630	AT2G05630.1	695
AT2G05630	AT2G05630.2	827
AT2G22040	AT2G22040.1	1080
AT2G31260	AT2G31260.1	3239
AT2G37840	AT2G37840.1	2631
AT2G37840	AT2G37840.2	2916
AT2G40810	AT2G40810.1	1576
AT2G40810	AT2G40810.2	1556
AT2G44140	AT2G44140.1	3221
AT2G44140	AT2G44140.2	2911
AT2G45170	AT2G45170.1	683
AT2G45170	AT2G45170.2	733
AT3G06420	AT3G06420.1	712
AT3G07525	AT3G07525.1	889
AT3G07525	AT3G07525.2	837
AT3G08850	AT3G08850.1	4819
AT3G13970	AT3G13970.1	529
AT3G15580	AT3G15580.1	623
AT3G18770	AT3G18770.1	2343
AT3G19190	AT3G19190.1	5990
AT3G49590	AT3G49590.1	2324
AT3G49590	AT3G49590.2	2240
AT3G49590	AT3G49590.3	2440
AT3G53930	AT3G53930.1	2558
AT3G53930	AT3G53930.2	2561
AT3G56440	AT3G56440.1	1656
AT3G59950	AT3G59950.1	1905
AT3G59950	AT3G59950.2	2228
AT3G59950	AT3G59950.3	1265
AT3G60640	AT3G60640.1	643
AT3G61710	AT3G61710.1	1895

AT3G61710	AT3G61710.2	1989
AT3G61710	AT3G61710.3	1991
AT3G61960	AT3G61960.1	2366
AT3G61960	AT3G61960.2	2241
AT3G62770	AT3G62770.1	1625
AT3G62770	AT3G62770.3	1771
AT4G04620	AT4G04620.1	725
AT4G04620	AT4G04620.2	677
AT4G08540	AT4G08540.1	1735
AT4G16520	AT4G16520.1	713
AT4G16520	AT4G16520.2	892
AT4G21980	AT4G21980.1	798
AT4G21980	AT4G21980.2	894
AT4G30510	AT4G30510.1	1261
AT4G30510	AT4G30510.2	958
AT4G30790	AT4G30790.1	3744
AT5G01770	AT5G01770.1	4448
AT5G05150	AT5G05150.1	1125
AT5G17290	AT5G17290.1	1308
AT5G45900	AT5G45900.1	2300
AT5G50230	AT5G50230.1	1818
AT5G54730	AT5G54730.1	3017
AT5G61500	AT5G61500.1	1413
AT5G66930	AT5G66930.1	1091
AT5G66930	AT5G66930.2	998
AT5G66930	AT5G66930.3	959

A.3 List of identificators of Protein, length and uniprot accession of each ATG genes in *A. thaliana.*

Protein Name	Protein Length	Protein UniProt Accession
AT1G03380.1	959	Q8GUL1
AT1G49180.1	408	F4I1N8
AT1G49180.2	376	F4I1N7
AT1G50030.1	2481	A0A178WN52
AT1G50030.2	2454	F4I4X6
AT1G54210.1	96	A0A178VZU7
AT1G54210.2	52	Q67YB5
AT1G54210.3	96	A0A178VZU7
AT1G54710.1	927	Q8H1Q5
AT1G54710.2	806	A0A1P8AW11
AT1G62040.1	119	Q8S927
AT1G62040.2	94	F4HX35
AT1G77890.1	460	A0A178WBI6
AT1G77890.2	449	B3H442
AT1G77890.3	458	F4I8K9
AT1G77890.4	377	A0A1P8AWE4
AT1G77890.5	460	A0A178WBI6
AT2G05630.1	120	Q9SL04
AT2G05630.2	164	F4IHC1
AT2G22040.1	313	F4IIK6
AT2G31260.1	866	A0A178W047
AT2G37840.1	733	F4IRW0
AT2G37840.2	596	F4IRW0
AT2G37840.3	447	A0A178VZ48
AT2G40810.1	393	Q8GYD7
AT2G40810.2	393	Q8GYD7
AT2G40810.3	268	A0A1P8B0S0
AT2G44140.1	467	A0A178VZV2
AT2G44140.2	422	Q8S929
AT2G44140.3	467	A0A178VZV2
AT2G44140.4	384	A0A178VZ47
AT2G44140.5	389	A0A1P8B129
AT2G45170.1	122	A0A178VZA0
AT2G45170.2	122	A0A178VZA0
AT3G06420.1	119	Q8S925
AT3G07525.1	225	A0A178V6V0
AT3G07525.2	226	A0A178V865
AT3G08850.1	1344	A0A178V9S6
AT3G13970.1	94	Q9LVK3
AT3G13970.2	67	A0A1I9LNQ3
AT3G13970.3	67	A0A1I9LNQ3
AT3G13970.4	87	A0A1I9LNQ2
AT3G15580.1	115	Q9LRP7
AT3G18140.1	305	A0A178VME6
AT3G18140.2	224	F4J7K1
AT3G18770.1	625	A0A178VAK8
AT3G19190.1	1839	

AT3G19190.2	1892	F8S296
AT3G19190.3	1892	F8S296
AT3G49590.1	603	Q9SCK0
AT3G49590.2	603	Q9SCK0
AT3G49590.3	618	F4IXZ6
AT3G53930.1	711	F4JBP3
AT3G53930.2	712	A8MR56
AT3G53930.3	526	A0A1I9LQJ7
AT3G53930.4	527	A0A1I9LQJ6
AT3G53930.5	526	A0A1I9LQJ7
AT3G56440.1	391	Q0WPK3
AT3G56440.2	362	A0A1I9LQF3
AT3G56440.3	331	A0A1I9LQF2
AT3G59950.1	477	A0A178VJJ3
AT3G59950.2	360	F4J9I3
AT3G59950.3	267	Q9M1Y0
AT3G59950.4	391	A0A1I9LMQ9
AT3G59950.5	393	A0A1I9LMQ8
AT3G60640.1	121	Q9LZZ9
AT3G61710.1	517	Q9M367
AT3G61710.2	386	Q9M367
AT3G61710.3	318	Q9M367
AT3G61710.4	467	A0A1I9LT68
AT3G61960.1	626	Q94C95
AT3G61960.2	584	F4IX14
AT3G62770.1	425	A0A178V9Z7
AT3G62770.3	396	F4IZI7
AT4G04620.1	122	Q9XEB5
AT4G04620.2	122	Q9XEB5
AT4G04620.3	122	Q9XEB5
AT4G08540.1	473	Q8H1E1
AT4G16520.1	121	Q8VYK7
AT4G16520.2	121	Q8VYK7
AT4G16520.3	121	A0A1P8B738
AT4G21980.1	122	A0A178UVA3
AT4G21980.2	137	A8MS84
AT4G30510.1	366	Q8H1Q8
AT4G30510.2	312	F4JQB6
AT4G30790.1	1148	Q9SUG7
AT5G01770.1	1336	Q9LZW9
AT5G01770.2	1192	A0A1P8BAT5
AT5G01770.3	1332	A0A1P8BAT3
AT5G01770.4	1331	A0A1P8BAS5
AT5G01770.5	1329	A0A1P8BAR8
AT5G05150.1	374	Q9FHK8
AT5G17290.1	337	Q9FFI2
AT5G45900.1	697	Q94CD5
AT5G50230.1	509	Q6NNP0

AT5G54730.1	763	Q9FH32
AT5G54730.2	651	A0A1R7T3J2
AT5G61500.1	313	Q0WWQ1
AT5G61500.2	306	A0A1P8BCL3
AT5G66930.1	157	F4K265
AT5G66930.2	215	F4K265
AT5G66930.3	251	F4K264

B.1 List of identificators of Gen, gene localization and length of each ATG genes in *P.vulgaris.*

Gene accession numbers	***Gene Length***	***Gene Chromosome Primary Identifier***	***Gene location***	***Gene location***
Phvul.007G210800	1251	Chr07	33282212	33283462
Phvul.006G149640	1933	Chr06	25471813	25473745
Phvul.011G103300	2352	Chr11	11510662	11513013
Phvul.010G130300	2465	Chr10	41141057	41143521
Phvul.003G079300	2947	Chr03	12725555	12728501
Phvul.001G205000	3005	Chr01	46312575	46315579
Phvul.007G196400	3036	Chr07	31976845	31979880
Phvul.010G036300	3199	Chr10	5365166	5368364
Phvul.003G219600	3412	Chr03	44794445	44797856
Phvul.002G062200	3611	Chr02	7317826	7321436
Phvul.003G207100	3740	Chr03	43273987	43277726
Phvul.006G173700	4372	Chr06	27671872	27676243
Phvul.010G120500	4567	Chr10	40105482	40110048
Phvul.011G006500	4613	Chr11	472854	477466
Phvul.008G187800	4725	Chr08	52535454	52540178
Phvul.009G041700	4840	Chr09	8502323	8507162
Phvul.002G269600	5044	Chr02	44011076	44016119
Phvul.011G010700	5130	Chr11	817643	822772
Phvul.008G048900	5411	Chr08	4270236	4275646
Phvul.007G194300	5775	Chr07	31618092	31623866
Phvul.011G140900	6185	Chr11	36027897	36034081
Phvul.003G152800	6378	Chr03	36768348	36774725
Phvul.005G091300	6464	Chr05	23812386	23818849
Phvul.001G146700	6471	Chr01	39328927	39335397
Phvul.003G153800	6786	Chr03	36865951	36872736
Phvul.005G029900	7212	Chr05	2766598	2773809
Phvul.007G183100	7327	Chr07	30276041	30283367
Phvul.001G159900	7414	Chr01	41311908	41319321
Phvul.003G248000	7507	Chr03	48513916	48521422
Phvul.008G241000	7959	Chr08	58970503	58978461
Phvul.008G169200	9652	Chr08	47325764	47335415
Phvul.008G088100	10302	Chr08	8689853	8700154
Phvul.003G295800	11590	Chr03	53263303	53274892
Phvul.010G015100	14023	Chr10	2253323	2267345
Phvul.008G087800	15146	Chr08	8649887	8665032
Phvul.002G049900	31655	Chr02	4626523	4658177

B.2 List of transcript accession and length of each ATG genes in *P. vulgaris.*

Gene accession numbers	*Transcript accession numbers*	*Transcript Gene Length*
Phvul.001G146700	Phvul.001G146700.1	3938
Phvul.001G146700	Phvul.001G146700.2	3459
Phvul.001G146700	Phvul.001G146700.3	3989
Phvul.001G159900	Phvul.001G159900.1	3211
Phvul.001G159900	Phvul.001G159900.2	3108
Phvul.001G159900	Phvul.001G159900.3	3744
Phvul.001G159900	Phvul.001G159900.4	2972
Phvul.001G159900	Phvul.001G159900.5	3173
Phvul.001G159900	Phvul.001G159900.6	3457
Phvul.001G159900	Phvul.001G159900.7	2658
Phvul.001G205000	Phvul.001G205000.1	1718
Phvul.002G062200	Phvul.002G062200.1	762
Phvul.002G062200	Phvul.002G062200.2	888
Phvul.002G269600	Phvul.002G269600.1	2262
Phvul.003G079300	Phvul.003G079300.1	777
Phvul.003G079300	Phvul.003G079300.2	629
Phvul.003G152800	Phvul.003G152800.1	1735
Phvul.003G152800	Phvul.003G152800.2	1732
Phvul.003G153800	Phvul.003G153800.1	4725
Phvul.003G207100	Phvul.003G207100.1	2169
Phvul.003G207100	Phvul.003G207100.2	1997
Phvul.003G219600	Phvul.003G219600.1	743
Phvul.003G248000	Phvul.003G248000.1	1198
Phvul.003G295800	Phvul.003G295800.2	6545
Phvul.003G295800	Phvul.003G295800.3	6535
Phvul.003G295800	Phvul.003G295800.4	6413
Phvul.005G029900	Phvul.005G029900.1	1810
Phvul.005G091300	Phvul.005G091300.1	3077
Phvul.005G091300	Phvul.005G091300.2	3159
Phvul.005G091300	Phvul.005G091300.3	2997
Phvul.006G149640	Phvul.006G149640.1	944
Phvul.006G149640	Phvul.006G149640.2	837
Phvul.006G173700	Phvul.006G173700.1	1534
Phvul.007G183100	Phvul.007G183100.1	3428
Phvul.007G194300	Phvul.007G194300.1	2728
Phvul.007G196400	Phvul.007G196400.1	1651
Phvul.007G210800	Phvul.007G210800.1	654
Phvul.008G048900	Phvul.008G048900.1	2194
Phvul.008G048900	Phvul.008G048900.2	2140
Phvul.008G048900	Phvul.008G048900.3	2014
Phvul.008G087800	Phvul.008G087800.1	5007
Phvul.008G088100	Phvul.008G088100.1	4269
Phvul.008G088100	Phvul.008G088100.2	2714
Phvul.008G088100	Phvul.008G088100.3	3401

Phvul.008G169200	Phvul.008G169200.1	1930
Phvul.008G169200	Phvul.008G169200.2	2489
Phvul.008G187800	Phvul.008G187800.1	2667
Phvul.008G187800	Phvul.008G187800.3	2743
Phvul.008G187800	Phvul.008G187800.4	2619
Phvul.008G187800	Phvul.008G187800.5	2470
Phvul.008G241000	Phvul.008G241000.1	1336
Phvul.009G041700	Phvul.009G041700.1	2026
Phvul.010G015100	Phvul.010G015100.2	2578
Phvul.010G015100	Phvul.010G015100.3	2530
Phvul.010G015100	Phvul.010G015100.4	2576
Phvul.010G036300	Phvul.010G036300.1	1173
Phvul.010G120500	Phvul.010G120500.1	2185
Phvul.010G120500	Phvul.010G120500.2	2127
Phvul.010G130300	Phvul.010G130300.1	602
Phvul.011G006500	Phvul.011G006500.1	1341
Phvul.011G006500	Phvul.011G006500.2	1329
Phvul.011G010700	Phvul.011G010700.1	2459
Phvul.011G103300	Phvul.011G103300.1	848
Phvul.011G103300	Phvul.011G103300.2	863
Phvul.011G140900	Phvul.011G140900.1	3507

B.3 List of identificators of Protein, length, isoelectric point and, molecular weight of each ATG genes in *P. vulgaris.*

Protein accession numbers	Protein accession numbers	Protein length	Isoelectric point	Proteins Molecular Weight
	Phvul.010G120500.1	627	6.34	70430.22
Phvul.010G120500	Phvul.010G120500.2	477	5.59	53617.62
Phvul.010G015100	Phvul.010G015100.2	733	6.16	81386.04
	Phvul.010G015100.3	717	6.49	79717.22
	Phvul.010G015100.4	655	6.25	72675.94
Phvul.003G295800	Phvul.003G295800.2	1977	5.43	217499.88
	Phvul.003G295800.3	1977	5.43	217499.88
	Phvul.003G295800.4	1933	5.32	212448.85
Phvul.011G006500	Phvul.011G006500.1	314	4.73	35345.69
	Phvul.011G006500.2	310	4.73	34932.21
Phvul.008G048900	Phvul.008G048900.1	489	5.45	53395.03
	Phvul.008G048900.2	489	5.45	53395.03
	Phvul.008G048900.3	397	4.98	43725.09
Phvul.008G241000	Phvul.008G241000.1	349	4.79	39237.55
Phvul.005G029900	Phvul.005G029900.1	489	5.91	55623.84
Phvul.011G010700	Phvul.011G010700.1	700	5.67	77256.47
Phvul.003G079300	Phvul.003G079300.1	119	7.92	13755.77
	Phvul.003G079300.2	119	7.92	13755.77
Phvul.011G103300	Phvul.010G103300.1	120	8.78	13891.19
	Phvul.010G103300.2	120	8.78	13891.19
Phvul.003G219600	Phvul.003G219600.1	123	7.85	14165.28
Phvul.002G062200	Phvul.002G062200.1	131	7.85	15086.34

	Phvul.002G062200.2	131	7.85	14973.18
Phvul.007G210800	Phvul.007G210800.1	122	6.73	14135.17
Phvul.001G159900	Phvul.001G159900.1	857	6.35	98197.83
	Phvul.001G159900.2	857	6.35	98197.83
	Phvul.001G159900.3	857	6.35	98197.83
	Phvul.001G159900.4	857	6.35	98197.83
	Phvul.001G159900.5	857	6.35	98197.83
	Phvul.001G159900.6	857	6.35	98197.83
	Phvul.001G159900.7	733	6.64	84490.12
Phvul.007G194300	Phvul.007G194300.1	873	6.24	101816.55
Phvul.010G036300	Phvul.010G036300.1	239	5.75	27706.34
Phvul.003G153800	Phvul.003G153800.1	1153	5.69	130516.48
Phvul.010G130300	Phvul.010G130300.1	94	9.25	10536.18
Phvul.008G187800	Phvul.008G187800.1	593	8.71	20572.81
	Phvul.008G187800.3	593	8.71	20572.81
	Phvul.008G187800.4	593	8.71	20572.81
	Phvul.008G187800.5	590	8.89	65397.9
Phvul.002G269600	Phvul.002G269600.1	625	8.83	69262.82
Phvul.003G207100	Phvul.003G207100.1	514	6.1	56511.95
	Phvul.003G207100.2	514	6.1	56511.95
Phvul.007G196400	Phvul.007G196400.1	380	8.09	42023.77
Phvul.003G152800	Phvul.003G152800.1	359	8.86	38887.62
	Phvul.003G152800.2	358	8.86	38800.54
Phvul.009G041700	Phvul.009G041700.1	422	8.6	46963.54
Phvul.005G091300	Phvul.005G091300.1	889	6.53	97215.94
	Phvul.005G091300.2	889	6.53	97215.94
	Phvul.005G091300.3	865	6.79	94521.68
Phvul.001G146700	Phvul.001G146700.1	975	5.39	106417
	Phvul.001G146700.2	978	5.36	106730.35
	Phvul.001G146700.3	758	6.7	82592.35
Phvul.011G140900	Phvul.011G140900.1	925	646	100644.54
Phvul.007G183100	Phvul.007G183100.1	907	5.68	98293.52

C.1 List of identificators of Gen, gene localization and length of each ATG genes in *M. truncatula.*

Gene accession numbers	***Gene length***	***Chromosome***	***Gene location***	***Gene Localization***
Medtr0003s0540	4893	scaffold0003	305855	310747
Medtr1g070160	6744	chr1	30830518	30837261
Medtr1g082300	8290	chr1	36587909	36596198
Medtr1g083230	3467	chr1	37037962	37041428
Medtr1g086310	1194	chr1	38625116	38626309
Medtr1g088855	2398	chr1	39776324	39778721
Medtr1g089110	5803	chr1	40103141	40108943
Medtr2g016690	5275	chr2	5150008	5155282
Medtr2g023430	2567	chr2	8277496	8280062
Medtr2g082770	6818	chr2	34727678	34734495
Medtr3g018770	8740	chr3	5165817	5174556
Medtr3g075400	3315	chr3	34315394	34318708

Medtr3g093590	5282	chr3	42763022	42768303
Medtr3g095570	6584	chr3	43671041	43677624
Medtr3g095620	2509	chr3	43689826	43692334
Medtr4g019410	8113	chr4	6057862	6065974
Medtr4g036265	5057	chr4	13052245	13057301
Medtr4g037225	2178	chr4	13715596	13717773
Medtr4g048510	3431	chr4	17207135	17210565
Medtr4g101090	2798	chr4	41752327	41752327
Medtr4g086370	17746	chr4	33827034	33844779
Medtr4g101090	2798	chr4	41752327	41755124
Medtr4g104380	3492	chr4	43185561	43189052
Medtr4g123760	2598	chr4	51007802	51010399
Medtr4g130190	6124	chr4	54209571	54215694
Medtr4g130370	6952	chr4	54307709	54314660
Medtr5g005380	33530	chr5	389766	423295
Medtr5g061040	9168	chr5	25385477	25394644
Medtr5g068710	4017	chr5	29098584	29102600
Medtr5g076920	6495	chr5	32806624	32813118
Medtr7g072330	14075	chr7	26857318	26871392
Medtr7g081230	4703	chr7	30993699	30998401
Medtr7g096540	631	chr7	38739985	38740615
Medtr7g096680	6213	chr7	38799346	38805558
Medtr7g108520	3709	chr7	44206217	44209925
Medtr8g010140	2288	chr8	2577226	2579513
Medtr8g020500	3779	chr8	7198686	7202464
Medtr8g024100	6388	chr8	8817813	8824200
Medtr8g079240	5388	chr8	33765931	33771318
Medtr8g093050	4858	chr8	38885014	38889871

C.2 List of transcript accession and length of each ATG genes in *M. truncatula.*

Gene accession numbers	*Transcript accession numbers*	*Transcript Gene Length*
Medtr0003s0540	Medtr0003s0540.1	2551
Medtr1g070160	Medtr1g070160.1	3257
Medtr1g082300	Medtr1g082300.1	3440
Medtr1g082300	Medtr1g082300.2	3514
Medtr1g082300	Medtr1g082300.3	3426
Medtr1g083230	Medtr1g083230.1	1624
Medtr1g086310	Medtr1g086310.1	805
Medtr1g088855	Medtr1g088855.1	1065
Medtr1g089110	Medtr1g089110.1	3661
Medtr1g089110	Medtr1g089110.2	2907
Medtr2g016690	Medtr2g016690.1	996
Medtr2g023430	Medtr2g023430.1	851
Medtr2g082770	Medtr2g082770.1	3526
Medtr2g082770	Medtr2g082770.2	3938
Medtr2g082770	Medtr2g082770.3	3821
Medtr3g018770	Medtr3g018770.1	1612

Medtr3g075400	Medtr3g075400.1	2251
Medtr3g093590	Medtr3g093590.1	1828
Medtr3g093590	Medtr3g093590.2	1950
Medtr3g095570	Medtr3g095570.1	2485
Medtr3g095570	Medtr3g095570.2	4610
Medtr3g095570	Medtr3g095570.3	2231
Medtr3g095620	Medtr3g095620.1	1348
Medtr4g007500	Medtr4g007500.1	1527
Medtr4g019410	Medtr4g019410.1	2766
Medtr4g019410	Medtr4g019410.2	3215
Medtr4g036265	Medtr4g036265.1	1411
Medtr4g036265	Medtr4g036265.2	944
Medtr4g036265	Medtr4g036265.3	1545
Medtr4g037225	Medtr4g037225.1	647
Medtr4g037225	Medtr4g037225.2	810
Medtr4g048510	Medtr4g048510.1	772
Medtr4g086370	Medtr4g086370.1	6668
Medtr4g086370	Medtr4g086370.2	6736
Medtr4g086370	Medtr4g086370.3	6761
Medtr4g086370	Medtr4g086370.4	6829
Medtr4g101090	Medtr4g101090.1	1000
Medtr4g101090	Medtr4g101090.2	967
Medtr4g104380	Medtr4g104380.1	2025
Medtr4g123760	Medtr4g123760.1	935
Medtr4g123760	Medtr4g123760.2	1166
Medtr4g130190	Medtr4g130190.1	1667
Medtr4g130190	Medtr4g130190.2	1795
Medtr4g130190	Medtr4g130190.3	1714
Medtr4g130190	Medtr4g130190.4	1946
Medtr4g130190	Medtr4g130190.5	1973
Medtr4g130190	Medtr4g130190.6	2562
Medtr4g130190	Medtr4g130190.7	2230
Medtr4g130370	Medtr4g130370.1	4565
Medtr4g130370	Medtr4g130370.2	5095
Medtr4g130370	Medtr4g130370.3	5223
Medtr5g005380	Medtr5g005380.1	8192
Medtr5g061040	Medtr5g061040.1	1934
Medtr5g061040	Medtr5g061040.2	1982
Medtr5g061040	Medtr5g061040.3	2470
Medtr5g068710	Medtr5g068710.1	2613
Medtr5g068710	Medtr5g068710.2	2621
Medtr5g068710	Medtr5g068710.3	2872
Medtr5g068710	Medtr5g068710.4	2468
Medtr5g076920	Medtr5g076920.1	1395
Medtr5g076920	Medtr5g076920.2	1356
Medtr7g072330	Medtr7g072330.1	4865
Medtr7g072330	Medtr7g072330.2	4832
Medtr7g081230	Medtr7g081230.1	2079

Medtr7g081230	Medtr7g081230.2	1980
Medtr7g081230	Medtr7g081230.3	2044
Medtr7g081230	Medtr7g081230.4	1964
Medtr7g096540	Medtr7g096540.1	189
Medtr7g096680	Medtr7g096680.1	3073
Medtr7g108520	Medtr7g108520.1	1630
Medtr7g108520	Medtr7g108520.2	1579
Medtr7g108520	Medtr7g108520.3	1558
Medtr8g010140	Medtr8g010140.1	708
Medtr8g020500	Medtr8g020500.1	851
Medtr8g020500	Medtr8g020500.2	866
Medtr8g024100	Medtr8g024100.1	2674
Medtr8g079240	Medtr8g079240.1	1165
Medtr8g079240	Medtr8g079240.2	1465
Medtr8g093050	Medtr8g093050.1	1931
Medtr8g093050	Medtr8g093050.2	1996
Medtr8g093050	Medtr8g093050.3	1939

C.3 List of identificators of Protein and, proteins uniprot accession of each ATG genes in *M. truncatula.*

Proteins Name	***Proteins Length***	***Proteins UniProt Accession***
Medtr0003s0540.1	698	A0A072TJQ4
Medtr1g070160.1	866	A0A072VWT7
Medtr1g082300.1	908	G7I7G6
Medtr1g082300.2	907	A0A072VMK6
Medtr1g082300.3	913	A0A072VNS1
Medtr1g083230.1	385	G7I8N8
Medtr1g086310.1	121	G7IBT1
Medtr1g088855.1	354	A0A072VN14
Medtr1g089110.1	967	G7I2R8
Medtr1g089110.2	968	A0A072VPC8
Medtr2g016690.1	331	A0A072V4P2
Medtr2g023430.1	120	I3S7J4
Medtr2g082770.1	901	G7IHE9
Medtr2g082770.2	901	G7IHE9
Medtr2g082770.3	901	G7IHE9
Medtr3g018770.1	509	G7IW08
Medtr3g075400.1	509	A0A072V0W5
Medtr3g093590.1	415	A0A072V2C7
Medtr3g093590.2	415	A0A072V2C7
Medtr3g095570.1	633	G7JAX9
Medtr3g095570.2	535	A0A072V173
Medtr3g095570.3	544	A0A072V2J6
Medtr3g095620.1	290	G7JAY4
Medtr4g007500.1	364	A0A072UHF9
Medtr4g019410.1	737	A0A072UGS9
Medtr4g019410.2	567	A0A072UIQ0

Medtr4g036265.1	310	B7FJ97
Medtr4g036265.2	279	A0A072UK19
Medtr4g036265.3	310	B7FJ97
Medtr4g037225.1	120	I3S805
Medtr4g037225.2	120	I3S805
Medtr4g048510.1	120	I3SA41
Medtr4g086370.1	1975	G7JLA6
Medtr4g086370.2	1975	G7JLA6
Medtr4g086370.3	1975	G7JLA6
Medtr4g086370.4	1975	G7JLA6
Medtr4g101090.1	122	G7JIY2
Medtr4g101090.2	122	G7JIY2
Medtr4g104380.1	514	A0A072UPU7
Medtr4g123760.1	118	G7JQ24
Medtr4g123760.2	118	G7JQ24
Medtr4g130190.1	314	G7JFG8
Medtr4g130190.2	314	G7JFG8
Medtr4g130190.3	372	G7JED6
Medtr4g130190.4	335	A0A072US15
Medtr4g130190.5	277	A0A072V375
Medtr4g130190.6	335	A0A072US15
Medtr4g130190.7	268	A0A072UTC7
Medtr4g130370.1	1154	G7JFI4
Medtr4g130370.2	1080	A0A072UTD2
Medtr4g130370.3	953	A0A072USH3
Medtr5g005380.1	2471	G7K0Z4
Medtr5g061040.1	479	G7K937
Medtr5g061040.2	430	A0A072UFZ9
Medtr5g061040.3	388	A0A072UE35
Medtr5g068710.1	584	G7K663
Medtr5g068710.2	584	G7K663
Medtr5g068710.3	584	G7K663
Medtr5g068710.4	585	A0A072UQL3
Medtr5g076920.1	361	G7KFU3
Medtr5g076920.2	263	A0A072UQT7
Medtr7g072330.1	1369	G7KW32
Medtr7g072330.2	1358	A0A072U238
Medtr7g081230.1	487	G7KTD2
Medtr7g081230.2	454	A0A072UC37
Medtr7g081230.3	487	G7KTD2
Medtr7g081230.4	395	A0A072U1Z8
Medtr7g096540.1	62	A0A072U2I0
Medtr7g096680.1	893	A0A072UDM3
Medtr7g108520.1	418	G7KRU6
Medtr7g108520.2	418	G7KRU6
Medtr7g108520.3	362	A0A072U4W3
Medtr8g010140.1	235	A0A072TKT3
Medtr8g020500.1	95	G7L6T7

Medtr8g020500.2	124	A0A072TLU6
Medtr8g024100.1	696	A0A072TMB4
Medtr8g079240.1	218	G7LFM8
Medtr8g079240.2	194	A0A072TTJ1
Medtr8g093050.1	557	G7LD52
Medtr8g093050.2	583	G7LD68
Medtr8g093050.3	557	G7LD52

D.1 List of identificators of Gen, gene localization and length of each ATG genes in *G.max.*

Gene accession numbers	***Gene Chromosome Primary Identifier***	***Gene location***	***Gene location***	***Gene Length***
Glyma.01G099600	9884	Chr01	33115843	33125726
Glyma.01G241300	46877	Chr01	56488899	56535775
Glyma.02G008800	1510	Chr02	882915	884424
Glyma.02G133400	13046	Chr02	13780268	13793313
Glyma.02G207500	5395	Chr02	39248283	39253677
Glyma.02G220700	5189	Chr02	40856235	40861423
Glyma.02G240700	7081	Chr02	42910921	42918001
Glyma.03G069800	15530	Chr03	15703649	15719178
Glyma.03G097000	4731	Chr03	28464508	28469238
Glyma.03G148700	6682	Chr03	36433817	36440498
Glyma.03G162100	7529	Chr03	37703629	37711157
Glyma.03G212100	3188	Chr03	41817313	41820500
Glyma.04G141000	16604	Chr04	23665626	23682229
Glyma.04G215500	3047	Chr04	48694717	48697763
Glyma.04G224300	11333	Chr04	49475519	49486851
Glyma.05G043700	3405	Chr05	3901985	3905389
Glyma.05G189000	6042	Chr05	37485611	37491652
Glyma.06G140400	6246	Chr06	11446439	11452684
Glyma.06G150700	2332	Chr06	12289992	12292323
Glyma.06G267000	6203	Chr06	45497961	45504163
Glyma.06G306300	2241	Chr06	49529272	49531512
Glyma.07G038100	3134	Chr07	3134753	3137886
Glyma.07G048400	5895	Chr07	4096301	4102195
Glyma.07G203900	416	Chr07	37321516	37321931
Glyma.07G211600	12673	Chr07	38313562	38326234
Glyma.07G261000	3362	Chr07	43631706	43635067
Glyma.08G146700	5858	Chr08	11166509	11172366
Glyma.09G003900	2413	Chr09	316898	319310
Glyma.09G231000	3859	Chr09	45443954	45447812
Glyma.09G244800	4638	Chr09	46708330	46712967
Glyma.09G278500	13095	Chr09	49384135	49397229
Glyma.10G009300	1295	Chr10	896609	897903
Glyma.10G035800	7072	Chr10	3130966	3138037
Glyma.10G126200	1752	Chr10	33430366	33432117
Glyma.10G152500	3610	Chr10	38725865	38729474
Glyma.10G157700	7160	Chr10	39177037	39184196

Glyma.11G002600	35094	Chr11	135007	170100
Glyma.11G153900	7392	Chr11	12389260	12396651
Glyma.12G005700	4759	Chr12	433113	437871
Glyma.12G010000	4977	Chr12	712288	717264
Glyma.12G098400	2286	Chr12	8463842	8466127
Glyma.12G136000	7244	Chr12	15957977	15965220
Glyma.12G214600	6868	Chr12	37403821	37410688
Glyma.13G085400	9416	Chr13	19705658	19715073
Glyma.13G122200	6035	Chr13	23509326	23515360
Glyma.13G227200	6124	Chr13	33967115	33973238
Glyma.13G287000	6862	Chr13	38736311	38743172
Glyma.14G167200	12727	Chr14	41352672	41365398
Glyma.14G187000	5061	Chr14	45159106	45164166
Glyma.14G210200	10002	Chr14	47535839	47545840
Glyma.15G085200	4424	Chr15	6531153	6535576
Glyma.15G108200	2255	Chr15	8512403	8514657
Glyma.15G188600	303	Chr15	19737953	19738255
Glyma.16G007300	3408	Chr16	599220	602627
Glyma.16G017300	5617	Chr16	1519674	1525290
Glyma.16G109400	2132	Chr16	23481725	23483856
Glyma.17G013000	2453	Chr17	992098	994550
Glyma.17G070200	5982	Chr17	5495613	5501594
Glyma.17G071400	6706	Chr17	5578787	5585492
Glyma.17G126200	4150	Chr17	10054028	10058177
Glyma.17G140700	3375	Chr17	11428453	11431827
Glyma.17G180900	4228	Chr17	20745714	20749941
Glyma.18G210300	14655	Chr18	49597315	49611969
Glyma.18G248400	5038	Chr18	53539192	53544229
Glyma.19G152000	7065	Chr19	41245379	41252443
Glyma.19G163500	7538	Chr19	42435811	42443348
Glyma.19G209200	3329	Chr19	46393051	46396379
Glyma.20G230900	4132	Chr20	46480639	46484770
Glyma.20G235800	3099	Chr20	46825099	46828197
Glyma.U032100	802	scaffold_31	152782	153583

D.2 List of transcript accession and length of each ATG genes in *G. max.*

Gene accession numbers	***Transcript accession numbers***	***Transcript Gene Length***
Glyma.01G099600	Glyma.01G099600.1	2651
Glyma.01G099600	Glyma.01G099600.2	2566
Glyma.01G099600	Glyma.01G099600.3	2645
Glyma.01G241300	Glyma.01G241300.1	8150
Glyma.01G241300	Glyma.01G241300.2	7043
Glyma.02G008800	Glyma.02G008800.1	652
Glyma.02G008800	Glyma.02G008800.2	919
Glyma.02G133400	Glyma.02G133400.1	6651
Glyma.02G133400	Glyma.02G133400.2	6640

Glyma.02G207500	Glyma.02G207500.1	1876
Glyma.02G207500	Glyma.02G207500.2	1993
Glyma.02G207500	Glyma.02G207500.3	1939
Glyma.02G220700	Glyma.02G220700.1	2732
Glyma.02G240700	Glyma.02G240700.1	1352
Glyma.02G240700	Glyma.02G240700.2	1348
Glyma.02G240700	Glyma.02G240700.3	1324
Glyma.02G240700	Glyma.02G240700.4	1320
Glyma.02G240700	Glyma.02G240700.5	1407
Glyma.02G240700	Glyma.02G240700.6	1411
Glyma.03G069800	Glyma.03G069800.1	2678
Glyma.03G069800	Glyma.03G069800.10	1829
Glyma.03G069800	Glyma.03G069800.11	1823
Glyma.03G069800	Glyma.03G069800.2	2729
Glyma.03G069800	Glyma.03G069800.3	2732
Glyma.03G069800	Glyma.03G069800.4	2436
Glyma.03G069800	Glyma.03G069800.5	2433
Glyma.03G069800	Glyma.03G069800.6	2666
Glyma.03G069800	Glyma.03G069800.7	2666
Glyma.03G069800	Glyma.03G069800.8	2669
Glyma.03G069800	Glyma.03G069800.9	1832
Glyma.03G097000	Glyma.03G097000.1	1271
Glyma.03G097000	Glyma.03G097000.2	1166
Glyma.03G097000	Glyma.03G097000.3	1157
Glyma.03G097000	Glyma.03G097000.4	1545
Glyma.03G148700	Glyma.03G148700.1	4131
Glyma.03G162100	Glyma.03G162100.1	3286
Glyma.03G162100	Glyma.03G162100.2	3222
Glyma.03G162100	Glyma.03G162100.3	3049
Glyma.03G162100	Glyma.03G162100.4	3111
Glyma.03G162100	Glyma.03G162100.5	3082
Glyma.03G212100	Glyma.03G212100.1	1556
Glyma.03G212100	Glyma.03G212100.2	1543
Glyma.04G141000	Glyma.04G141000.1	1993
Glyma.04G141000	Glyma.04G141000.2	1946
Glyma.04G141000	Glyma.04G141000.3	1667
Glyma.04G141000	Glyma.04G141000.4	1614
Glyma.04G215500	Glyma.04G215500.1	1751
Glyma.04G215500	Glyma.04G215500.2	1582
Glyma.04G215500	Glyma.04G215500.3	2612
Glyma.04G215500	Glyma.04G215500.4	1817
Glyma.04G224300	Glyma.04G224300.1	4208
Glyma.04G224300	Glyma.04G224300.2	2373
Glyma.04G224300	Glyma.04G224300.3	2159
Glyma.05G043700	Glyma.05G043700.1	2473
Glyma.05G189000	Glyma.05G189000.1	2915
Glyma.05G189000	Glyma.05G189000.2	2604
Glyma.06G140400	Glyma.06G140400.1	2167

Glyma.06G140400	Glyma.06G140400.2	1925
Glyma.06G140400	Glyma.06G140400.3	1997
Glyma.06G150700	Glyma.06G150700.1	930
Glyma.06G267000	Glyma.06G267000.1	4001
Glyma.06G306300	Glyma.06G306300.1	786
Glyma.07G038100	Glyma.07G038100.1	819
Glyma.07G038100	Glyma.07G038100.2	993
Glyma.07G048400	Glyma.07G048400.1	2767
Glyma.07G048400	Glyma.07G048400.2	2335
Glyma.07G203900	Glyma.07G203900.1	416
Glyma.07G211600	Glyma.07G211600.1	7248
Glyma.07G211600	Glyma.07G211600.2	7076
Glyma.07G211600	Glyma.07G211600.3	6549

D.3 List of identificators of protein, length and uniprot accession of each ATG genes in *G. max.*

Proteins Name	***Proteins Length***	***Proteins UniProt Accession***
Glyma.01G099600.1.p	725	I1J6X2
Glyma.01G099600.2.p	725	I1J6X3
Glyma.01G099600.3.p	723	A0A0R0L8Z2
Glyma.01G241300.1.p	2468	I1JAW9
Glyma.01G241300.2.p	2196	A0A0R0LMJ1
Glyma.02G008800.1.p	122	B9A7M1
Glyma.02G008800.2.p	111	I1JBC1
Glyma.02G133400.1.p	1977	K7K841
Glyma.02G133400.2.p	1977	K7K841
Glyma.02G207500.1.p	368	K7K9T5
Glyma.02G207500.2.p	367	I1JGT6
Glyma.02G207500.3.p	314	I1JGT8
Glyma.02G220700.1.p	594	I1JH67
Glyma.02G240700.1.p	350	I1JHS0
Glyma.02G240700.2.p	253	K7KAG3
Glyma.02G240700.3.p	193	K7KAG7
Glyma.02G240700.4.p	193	K7KAG7
Glyma.02G240700.5.p	222	K7KAG5
Glyma.02G240700.6.p	222	K7KAG5
Glyma.03G069800.10.p	417	A0A0R0KFQ7
Glyma.03G069800.11.p	417	A0A0R0KFQ7
Glyma.03G069800.1.p	735	K7KDC2
Glyma.03G069800.2.p	733	A0A0R0KFT2
Glyma.03G069800.3.p	734	K7KDC3
Glyma.03G069800.4.p	555	A0A0R0KNA1
Glyma.03G069800.5.p	554	A0A0R0KQ39
Glyma.03G069800.6.p	731	A0A0R0KG83
Glyma.03G069800.7.p	731	A0A0R0KFR0
Glyma.03G069800.8.p	732	K7KDC4
Glyma.03G069800.9.p	418	A0A0R0KMM4
Glyma.03G097000.1.p	249	A0A0R0KR59

Glyma.03G097000.2.p	243	A0A0R0KPB6
Glyma.03G097000.3.p	240	C6TNN8
Glyma.03G097000.4.p	240	C6TNN8
Glyma.03G148700.1.p	979	I1JNP9
Glyma.03G162100.1.p	872	K7KFE6
Glyma.03G162100.2.p	872	K7KFE6
Glyma.03G162100.3.p	872	K7KFE6
Glyma.03G162100.4.p	872	K7KFE6
Glyma.03G162100.5.p	872	K7KFE6
Glyma.03G212100.1.p	423	I1JQJ8
Glyma.03G212100.2.p	401	I1JQJ9
Glyma.04G141000.1.p	509	I1JWG4
Glyma.04G141000.2.p	445	A0A0R0KHA3
Glyma.04G141000.3.p	434	A0A0R0KF84
Glyma.04G141000.4.p	466	A0A0R0KCW7
Glyma.04G215500.1.p	369	I1JY45
Glyma.04G215500.2.p	307	I1JY46
Glyma.04G215500.3.p	224	I1JY48
Glyma.04G215500.4.p	301	I1JY47
Glyma.04G224300.1.p	419	I1JYD8
Glyma.04G224300.2.p	419	I1JYD8
Glyma.04G224300.3.p	419	I1JYD8
Glyma.05G043700.1.p	514	I1K068
Glyma.05G189000.1.p	618	I1K4W4
Glyma.05G189000.2.p	618	I1K4W4
Glyma.06G140400.1.p	420	K7KUZ3
Glyma.06G140400.2.p	411	K7KUZ5
Glyma.06G140400.3.p	420	K7KUZ3
Glyma.06G150700.1.p	309	K7KV61
Glyma.06G267000.1.p	905	K7KXM5
Glyma.06G306300.1.p	120	C6T0R7
Glyma.07G038100.1.p	94	C6T5T5
Glyma.07G038100.2.p	71	A0A0R0IZ35
Glyma.07G048400.1.p	664	I1KHM7
Glyma.07G048400.2.p	571	A0A0R0IZG5
Glyma.07G203900.1.p	68	A0A0R0JCG3
Glyma.07G211600.1.p	1978	K7L2Z3
Glyma.07G211600.2.p	1978	K7L2Z3
Glyma.07G211600.3.p	1978	K7L2Z3

Supplemental Table S2 List of ATG18 proteins in *P. vulgaris. (A)* Molecular weight, theoretical isoelectric point, extinction coefficients, estimated half-life, instability index, aliphatic index, and grand average of hydropathicity (GRAVY). (B)Prediction of protein localization sites composition.

A

	# a.a.	MW	pI	a.a.Composition	(Asp + Glu)	(Arg + Lys)	Atomic composition	Formula:	#atoms	Extinction coefficents	Estimated half-life:	Instability index:	Aliphatic index:	GRAVY
PvATG18a	423	466689.77	6.6	Ala (A) 29 6.9% Arg (R) 20 4.7% Asn (N) 21 5.0% Asp (D) 20 4.7% Cys (C) 10 2.4% Gln (Q) 23 5.4% Glu (E) 20 4.7% Gly (G) 26 6.1% His (H) 11 2.6% Ile (I) 22 5.2% Leu (L) 33 7.8% Lys (K) 18 4.3% Met (M) 6 1.4% Phe (F) 24 5.7% Pro (P) 35 8.3% Ser (S) 43 10.2% Thr (T) 22 5.2% Trp (W) 4 0.9% Tyr (Y) 11 2.6% Val (V) 25 5.9% Pyl (O) 0 0.0% Sec (U) 0 0.0% (B) 0 0.0% (Z) 0 0.0% (X) 0 0.0%	40	38	Carbon C 2078 Hydrogen H 3211 Nitrogen N 571 Oxygen O 624 Sulfur S 16	C2078 H3211 N571 O624 S16	6500	Extinction coefficients are in units of M-1 cm-1, at 280 nm measured in water. Ext. coefficient 39015 Abs 0.1% (=1 g/l) 0.836, assuming all pairs of Cys residues form cystines Ext. coefficient 38390 Abs 0.1% (=1 g/l) 0.822, assuming all Cys residues are reduced	The N-terminal o the sequence considered is M (Met). The estimated half-lif is: 30 hours (mammalian reticulocytes, in vitro). >20 hours (yeast, in vivo). >10 hours (Escherichia coli in vivo).	The instability index (II) is computed to l 56.76 This classifies the protein as unstable.	74.7	-0.325
PvATG18c.I	422	46963.54	8.6	Ala (A) 23 5.5% Arg (R) 27 6.4% Asn (N) 28 6.6% Asp (D) 19 4.5% Cys (C) 11 2.6% Gln (Q) 18 4.3% Glu (E) 16 3.8% Gly (G) 31 7.3% His (H) 11 2.6% Ile (I) 25 5.9% Leu (L) 39 9.2% Lys (K) 13 3.1% Met (M) 12 2.8% Phe (F) 25 5.9% Pro (P) 19 4.5% Ser (S) 43 10.2% Thr (T) 21 5.0% Trp (W) 4 0.9% Tyr (Y) 10 2.4% Val (V) 27 6.4% Pyl (O) 0 0.0% Sec (U) 0 0.0% (B) 0 0.0% (Z) 0 0.0% (X) 0 0.0%	35	40	Carbon C 2074 Hydrogen H 3246 Nitrogen N 588 Oxygen O 613 Sulfur S 23	C2074 H3246 N588 O613 S23	6544	Extinction coefficients are in units of M-1 cm-1, at 280 nm measured in water.Ext. coefficient 37525Abs 0.1% (=1 g/l) 0.799, assuming all pairs of Cys residues form cystinesExt. coefficient 36900Abs 0.1% (=1 g/l) 0.786, assuming all Cys residues are reduced	The N-terminal o the sequence considered is M (Met). The estimated half-life is: 30 hours (mammali reticulocytes, in vitro). >20 hours (yeast in vivo). >10 hours (Escherichia coli, in vivo).	The instability index (II) is computed to be 37.21 This classifies the protein as stable.	83.15	-0.151
PvATG18c.II	380	42023.77	8.09	Ala (A) 30 7.9% Arg (R) 24 6.3% Asn (N) 12 3.2% Asp (D) 21 5.5% Cys (C) 8 2.1% Gln (Q) 16 4.2% Glu (E) 16 4.2% Gly (G) 30 7.9% His (H) 14 3.7% Ile (I) 17 4.5% Leu (L) 34 8.9% Lys (K) 15 3.9% Met (M) 8 2.1% Phe (F) 24 6.3% Pro (P) 16 4.2% Ser (S) 35 9.2% Thr (T) 20 5.3% Trp (W) 3 0.8% Tyr (Y) 10 2.6% Val (V) 27 7.1% Pyl (O) 0 0.0% Sec (U) 0 0.0% (B) 0 0.0% (Z) 0 0.0% (X) 0 0.0%	37	39	Carbon C 1869 Hydrogen H 2904 Nitrogen N 526 Oxygen O 548 Sulfur S 16	C1869 H2904 N526 O548 S16	5863	Extinction coefficients are in units of M-1 cm-1, at 280 nm measured in water. Ext. coefficient 31900 Abs 0.1% (=1 g/l) 0.759, assuming all pairs of Cys residues form cystines Ext. coefficient 31400 Abs 0.1% (=1 g/l) 0.747, assuming all Cys residues are reduced	The N-terminal of the sequence considered is M (Met). The estimated half-life is: 30 hours (mammalian reticulocytes, in vitro). >20 hours (yeast, in vivo). >10 hours (Escherichia coli, in vivo).	The instability index (II) is computed to be 46.02 This classifies the protein as unstable.	80.84	-0.154
PvATG18b	359	38887.62	8.86	Ala (A) 33 9.2% Arg (R) 18 5.0% Asn (N) 19 5.3% Asp (D) 13 3.6% Cys (C) 8 2.2% Gln (Q) 6 1.7% Glu (E) 11 3.1% Gly (G) 21 5.8% His (H) 10 2.8% Ile (I) 27 7.5% Leu (L) 40 11.1% Lys (K) 12 3.3% Met (M) 6 1.7% Phe (F) 19 5.3% Pro (P) 13 3.6% Ser (S) 50 13.9% Thr (T) 18 5.0% Trp (W) 1 0.3% Tyr (Y) 12 3.3% Val (V) 22 6.1% Pyl (O) 0 0.0% Sec (U) 0 0.0% (B) 0 0.0% (Z) 0 0.0% (X) 0 0.0%	24	30	Carbon C 1737 Hydrogen H 2749 Nitrogen N 471 Oxygen O 513 Sulfur S 14	C1737 H2749 N471 O513 S14	5484	Extinction coefficients are in units of M-1 cm-1, at 280 nm measured in water. Ext. coefficient 23880 Abs 0.1% (=1 g/l) 0.614, assuming all pairs of Cys residues form cystines Ext. coefficient 23380 Abs 0.1% (=1 g/l) 0.601, assuming all Cys residues are reduced	The N-terminal of the sequence considered is M (Met). The estimated half-life is: 30 hours (mammalian reticulocytes, in vitro). >20 hours (yeast, in vivo). >10 hours (Escherichia coli, in vivo).	he instability index (II) is computed to be 39.65 This classifies the protein as stable.	99.75	0.224
PvATG18f.I	925	100644.54	6.46	Ala (A) 59 6.4% Arg (R) 41 4.4% Asn (N) 55 5.9% Asp (D) 44 4.8% Cys (C) 14 1.5% Gln (Q) 26 2.8% Glu (E) 48 5.2% Gly (G) 81 8.8% His (H) 31 3.4% Ile (I) 57 6.2% Leu (L) 68 7.4% Lys (K) 43 4.6% Met (M) 19 2.1% Phe (F) 38 4.1% Pro (P) 40 4.3% Ser (S) 103 11.1% Thr (T) 46 5.0% Trp (W) 11 1.2% Tyr (Y) 24 2.6% Val (V) 77 8.3% Pyl (O) 0 0.0% Sec (U) 0 0.0% (B) 0 0.0% (Z) 0 0.0% (X) 0 0.0%	92	84	Carbon C 4439 Hydrogen H 6953 Nitrogen N 1245 Oxygen O 1364 Sulfur S 33	C4439 H6953 N1245 O1364 S33	14034	xtinction coefficients are in units of M-1 cm-1, at 280 nm measured in water. Ext. coefficient 97135 Abs 0.1% (=1 g/l) 0.965, assuming all pairs of Cys residues form cystines Ext. coefficient 96260 Abs 0.1% (=1 g/l) 0.956, assuming all Cys residues are reduced	The N-terminal of the sequence considered is M (Met). The estimated half-life is: 30 hours (mammalian reticulocytes, in vitro). >20 hours (yeast, in vivo). >10 hours (Escherichia coli, in vivo).	The instability index (II) is computed to be 41.01 This classifies the protein as unstable.	83.22	-0.202
PvATG18f.II	889	97215.94	6.53	Ala (A) 52 5.8% Arg (R) 42 4.7% Asn (N) 49 5.5% Asp (D) 46 5.2% Cys (C) 6 0.7% Gln (Q) 39 4.4% Glu (E) 41 4.6% Gly (G) 70 7.9% His (H) 31 3.5% Ile (I) 56 6.3% Leu (L) 61 6.9% Lys (K) 38 4.3% Met (M) 22 2.5% Phe (F) 32 3.6% Pro (P) 38 4.3% Ser (S) 118 13.3% Thr (T) 44 4.9% Trp (W) 10 1.1% Tyr (Y) 25 2.8% Val (V) 69 7.8% Pyl (O) 0 0.0% Sec (U) 0 0.0% (B) 0 0.0% (Z) 0 0.0% (X) 0 0.0%	87	80	Carbon C 4260 Hydrogen H 6685 Nitrogen N 1213 Oxygen O 1339 Sulfur S 28	C4260 H6685 N1213 O1339 S28	13525	Extinction coefficients are in units of M-1 cm-1, at 280 nm measured in water. Ext. coefficient 92625 Abs 0.1% (=1 g/l) 0.953, assuming all pairs of Cys residues form cystines Ext. coefficient 92250 Abs 0.1% (=1 g/l) 0.949, assuming all Cys residues are reduced	The N-terminal of the sequence considered is M (Met). The estimated half-life is: 30 hours (mammalian reticulocytes, in vitro). >20 hours (yeast, in vivo). >10 hours (Escherichia coli, in vivo).	The instability index (II) is computed to be 44.71 This classifies the protein as unstable.	79.69	-0.327
PvATG18g.I	975	106417	5.39	Ala (A) 47 4.8% Arg (R) 38 3.9% Asn (N) 52 5.3% Asp (D) 52 5.3% Cys (C) 21 2.2% Gln (Q) 35 3.6% Glu (E) 55 5.6%Gly (G) 74 7.6% His (H) 29 3.0% Ile (I) 53 5.4% Leu (L) 81 8.3% Lys (K) 38 3.9% Met (M) 15 1.5% Phe (F) 44 4.5%Pro (P) 48 4.9% Ser (S) 134 13.7% Thr (T) 52 5.3% Trp (W) 12 1.2% Tyr (Y) 24 2.5% Val (V) 71 7.3% Pyl (O) 0 0.0%Sec (U) 0 0.0% (B) 0 0.0% (Z) 0 0.0% (X) 0 0.0%	107	76	Carbon C 4676 Hydrogen H 7266 Nitrogen N 1284 Oxygen O 1487 Sulfur S 36	C4676 H7266 N1284 O1487 S36	14749	Extinction coefficients are in units of M-1 cm-1, at 280 nm measured in water. Ext. coefficient 103010 Abs 0.1% (=1 g/l) 0.968, assuming all pairs of Cys residues form cystines Ext. coefficient 101760 Abs 0.1% (=1 g/l) 0.956, assuming all Cys residues are reduced	The N-terminal of the sequence considered is M (Met). The estimated half-life is: 30 hours (mammalian reticulocytes, in vitro). >20 hours (yeast, in vivo). >10 hours (Escherichia coli, in vivo).	The instability index (II) is computed to be 44.41 This classifies the protein as unstable.	79.54	-0.256
PvATG18g.II	907	98293.52	5.68	Ala (A) 63 6.9% Arg (R) 34 3.7% Asn (N) 40 4.4% Asp (D) 47 5.2% Cys (C) 21 2.3% Gln (Q) 29 3.2% Glu (E) 48 5.3% Gly (G) 62 6.8% His (H) 29 3.2% Ile (I) 47 5.2% Leu (L) 66 7.3% Lys (K) 38 4.2% Met (M) 19 2.1% Phe (F) 37 4.1% Pro (P) 51 5.6% Ser (S) 128 14.1% Thr (T) 39 4.3% Trp (W) 13 1.4% Tyr (Y) 21 2.3% Val (V) 75 8.3% Pyl (O) 0 0.0% Sec (U) 0 0.0% (B) 0 0.0% (Z) 0 0.0% (X) 0 0.0%	95	72	Carbon C 4323 Hydrogen H 6729 Nitrogen N 1187 Oxygen O 1355 Sulfur S 40	C4323 H6729 N1187 O1355 S40	13634	Extinction coefficients are in units of M-1 cm-1, at 280 nm measured in water. Ext. coefficient 104040 Abs 0.1% (=1 g/l) 1.058, assuming all pairs of Cys residues form cystines Ext. coefficient 102790 Abs 0.1% (=1 g/l) 1.046, assuming all Cys residues are reduced	The N-terminal of the sequence considered is M (Met).The estimated half-life is: 30 hours (mammalian reticulocytes, in vitro). >20 hours (yeast, in vivo). >10 hours (Escherichia coli, in vivo).	The instability index (II) is computed to be 51.36This classifies the protein as unstable.	79.51	-0.177

Number of amino acids (# a.a.) Molecular weight (MW), Theoretical pI (pI), Amino acid composition (a.a. composition), Total number of negatively charged residues (Asp + Glu), Total number of positively charged residues (Arg + Lys), Total number of atoms(# atoms) andd Grand average of hydropathicity (GRAVY):

B

	Prediction of Protein Localization Sites	*Transmembranal localization*	*Transmebranal*	*Signal peptide*
PvATG18a	chloroplast stroma --- Certainty= 0.647(Affirmative) < succ><br>chloroplast thylakoid membrane --- Certainty= 0.470(Affirmative) < succ><br>cytoplasm --- Certainty= 0.450(Affirmative) < succ><br>chloroplast thylakoid space --- Certainty= 0.411(Affirmative) < succ>	Length: 423<br>Number of predicted TMHs: 0<br>Exp number of AAs in TMHs: 0.00940999999999999999<br>Exp number, first 60 AAs: 0<br>Total prob of N-in: 0.00332<br>TMHMM2.0 outside 1 423	no transmembranal	Signal peptide cleavage site predicted: none Sequence 167 RSVRLRR|DR 0.526 *ProP*<br><br>Propeptide cleavage sites predicted: Arg(R)/Lys(K): 1
PvATG18c.I	cytoplasm --- Certainty= 0.450(Affirmative) < succ><br>microbody (peroxisome) --- Certainty= 0.137(Affirmative) < succ><br>mitochondrial matrix space --- Certainty= 0.100(Affirmative) < succ><br>chloroplast thylakoid membrane --- Certainty= 0.100(Affirmative) < succ>	Length: 417<br>Number of predicted TMHs: 0<br>Exp number of AAs in TMHs: 0.880960000000000001<br>Exp number, first 60 AAs: 0.0004<br>Total prob of N-in: 0.02266<br>TMHMM2.0 outside 1 417	no transmembranal	Signal peptide cleavage site predicted: none<br><br>Propeptide cleavage sites predicted: Arg(R)/Lys(K): 0
PvATG18c.II	microbody (peroxisome) --- Certainty= 0.542(Affirmative) < succ><br>chloroplast stroma --- Certainty= 0.513(Affirmative) < succ><br>cytoplasm --- Certainty= 0.450(Affirmative) < succ><br>chloroplast thylakoid membrane --- Certainty= 0.269(Affirmative) < succ>	Length: 380<br>Number of predicted TMHs: 0<br>Exp number of AAs in TMHs: 0.550370000000000002<br>Exp number, first 60 AAs: 0.00096<br>Total prob of N-in: 0.02494<br>TMHMM2.0 outside 1 380	no transmembranal	Signal peptide cleavage site predicted: none<br><br>Propeptide cleavage sites predicted: Arg(R)/Lys(K): 0
PvATG18b	chloroplast thylakoid membrane --- Certainty= 0.650(Affirmative) < succ><br>mitochondrial inner membrane --- Certainty= 0.603(Affirmative) < succ><br>endoplasmic reticulum (membrane) --- Certainty= 0.600(Affirmative) < succ><br>chloroplast stroma --- Certainty= 0.426(Affirmative) < succ>	Length: 359<br>Number of predicted TMHs: 1<br>Exp number of AAs in TMHs: 18.64803<br>Exp number, first 60 AAs: 12.77989<br>Total prob of N-in: 0.75258<br>POSSIBLE N-term signal sequence<br>TMHMM2.0 inside 1 44<br>TMHMM2.0 TMhelix 45 67<br>TMHMM2.0 outside 68 359	Si transmembranal	Signal peptide cleavage site predicted: none<br><br>Propeptide cleavage sites predicted: Arg(R)/Lys(K): 0
PvATG18f.I	endoplasmic reticulum (membrane) --- Certainty= 0.820(Affirmative) < succ><br>plasma membrane --- Certainty= 0.190(Affirmative) < succ><br>microbody (peroxisome) --- Certainty= 0.142(Affirmative) < succ><br>endoplasmic reticulum (lumen) --- Certainty= 0.100(Affirmative) < succ>	Length: 925<br>Number of predicted TMHs: 1<br>Exp number of AAs in TMHs: 23.1784699999999999<br>Exp number, first 60 AAs: 22.6178<br>Total prob of N-in: 0.95521<br>POSSIBLE N-term signal sequence<br>TMHMM2.0 inside 1 11<br>TMHMM2.0 TMhelix 12 34<br>TMHMM2.0 outside 35 925	Si transmembranal	Signal peptide cleavage site predicted: between pos. 32 and 33: VLG-MS<br><br>Propeptide cleavage sites predicted: Arg(R)/Lys(K): 0
PvATG18f.II	endoplasmic reticulum (membrane) --- Certainty= 0.820(Affirmative) < succ><br>plasma membrane --- Certainty= 0.190(Affirmative) < succ><br>nucleus --- Certainty= 0.180(Affirmative) < succ><br>microbody (peroxisome) --- Certainty= 0.131(Affirmative) < succ>	Length: 889<br>Number of predicted TMHs: 1<br>Exp number of AAs in TMHs: 22.28907<br>Exp number, first 60 AAs: 19.59006<br>Total prob of N-in: 0.84906<br>POSSIBLE N-term signal sequence<br>TMHMM2.0 inside 1 6<br>TMHMM2.0 TMhelix 7 26<br>TMHMM2.0 outside 27 889	Si transmembranal	Signal peptide cleavage site predicted: between pos. 26 and 27: VMG-MR<br><br>Propeptide cleavage sites predicted: Arg(R)/Lys(K): 0
PvATG18g.I	outside --- Certainty= 0.370(Affirmative) < succ><br>endoplasmic reticulum (membrane) --- Certainty= 0.100(Affirmative) < succ><br>endoplasmic reticulum (lumen) --- Certainty= 0.100(Affirmative) < succ><br>Golgi body --- Certainty= 0.100(Affirmative) < succ>	Length: 975<br>Number of predicted TMHs: 0<br>Exp number of AAs in TMHs: 0.0865<br>Exp number, first 60 AAs: 0.0253<br>Total prob of N-in: 0.00386<br>TMHMM2.0 outside 1 975	no transmembranal	Signal peptide cleavage site predicted: none<br><br>Propeptide cleavage sites predicted: Arg(R)/Lys(K): 0
PvATG18g.II	chloroplast stroma --- Certainty= 0.524(Affirmative) < succ><br>cytoplasm --- Certainty= 0.450(Affirmative) < succ><br>microbody (peroxisome) --- Certainty= 0.316(Affirmative) < succ><br>chloroplast thylakoid membrane --- Certainty= 0.286(Affirmative) < succ>	Length: 907<br>Number of predicted TMHs: 0<br>Exp number of AAs in TMHs: 0.38325<br>Exp number, first 60 AAs: 0.00541<br>Total prob of N-in: 0.01864<br>TMHMM2.0 outside 1 907	no transmembranal	Signal peptide cleavage site predicted: none<br><br>Propeptide cleavage sites predicted: Arg(R)/Lys(K): 0

Supplementary table S3: List of oligos for RT-qPCR

Gene identifier	Gene		Oligonucleotide sequence
**Phvul.003G295800**	ATG2	F	5´-CAA CAC AAT GCT TGC ACG GTG A-3´
		R	5´-GTG CTA CCA TTG TTC AAA GGT GA-3´
**Phvul.007G210800**	ATG8i	F	5´-GCG ATC TGC CTG AGT TGG AG-3´
		R	5´-CAG TTT GAG GCA AGG TAT TCT TCA-3´
**Phvul.010G036300**	ATG9	F	5´-TGG GCA ACT ATT GCC GCT CAA-3´
		R	5´-CAT CCA TTC ACT CGT ACC ACA TGG-3´
**Phvul.007G194300**	ATG10	F	5´-CCA GGA CCC TTG AGT TGG CTT TA-3´
		R	5´-TCA GAA AGA GAT GTG CCA GCA TG-3´
